# Intermolecular Carbosilylation of α‐Olefins with C(sp^3^)−C(sp) Bond Formation Involving Silylium‐Ion Regeneration

**DOI:** 10.1002/anie.202203347

**Published:** 2022-04-19

**Authors:** Tao He, Zheng‐Wang Qu, Hendrik F. T. Klare, Stefan Grimme, Martin Oestreich

**Affiliations:** ^1^ Institut für Chemie Technische Universität Berlin Strasse des 17. Juni 115 10623 Berlin Germany; ^2^ Mulliken Center for Theoretical Chemistry Institut für Physikalische und Theoretische Chemie Rheinische Friedrich-Wilhelms-Universität Bonn Beringstraße 4 53115 Bonn Germany

**Keywords:** Alkenes, Carbosilylation, Cationic Reactions, Density Functional Calculations, Silylium Ions

## Abstract

A regioselective addition of alkynylsilanes across unactivated, terminal alkenes is reported. The reaction is initiated by the capture of a sterically unhindered silylium ion by a silylated phenylacetylene derivative to form a bis(silylated) ketene‐like carbocation. This in situ‐generated key intermediate is the actual catalyst that maintains the catalytic cycle by a series of electrophilic addition reactions of silylium ions and β‐silicon‐stabilized carbocations. The computed reaction mechanism is fully consistent with the experimental findings. This unprecedented two‐component carbosilylation establishes a C(sp^3^)−C(sp) bond and a C(sp^3^)−Si bond in atom‐economic fashion.

## Graphical abstract

Sterically unhindered silylium ions can initiate the silylalkynylation of terminal alkenes with silylated phenylacetylene derivatives in a two‐component reaction. These alkynylsilanes and the silylium‐ion initiator form bis(silylated) ketene‐like carbocations as the actual catalysts (gray box). This new carbosilylation enables the formation of a C(sp^3^)−C(sp) bond (see scheme).

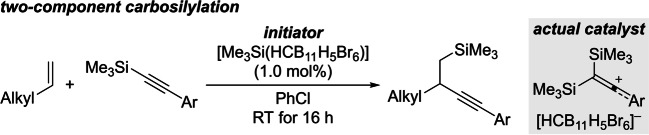

Regioselective difunctionalization of alkenes with formation of a carbon‐carbon and a carbon‐heteroatom bond in a single synthetic operation is currently experiencing renewed interest.^[1]^ These reactions are typically three‐component systems with one added to the unsaturated unit and the other subsequently coupled to the formed reactive intermediate. This can be achieved in several ways, and recently developed radical processes prevail for the carbosilylation of alkenes (Scheme 1, top).^[2]^ These methods are mainly applicable to the silyl(hetero)arylation and ‐carboxylation of styrene derivatives as is a nickel‐catalyzed silylacylation (not shown).^[3]^ Examples of the carbosilylation of unactivated alkenes are rare[^4^, ^5^] but a cationic two‐component silylallylation of terminal alkenes stands out (Scheme 1, center).[^6^, ^7^] This proton‐initiated transformation relies on *self‐regeneration of silylium ions*,[^8^, ^9^] making use of allylsilanes as the source of both the carbon nucleophile and the silicon electrophile. Inspired by this work, we envisaged a silylalkynylation of α‐olefins to enable for the first time C(sp^3^)−C(sp) bond formation as part of a carbosilylation reaction by using alkynyl‐^[10]^ instead of allylsilanes (Scheme 1, bottom).[^11^, ^12^]

**Scheme 1:**
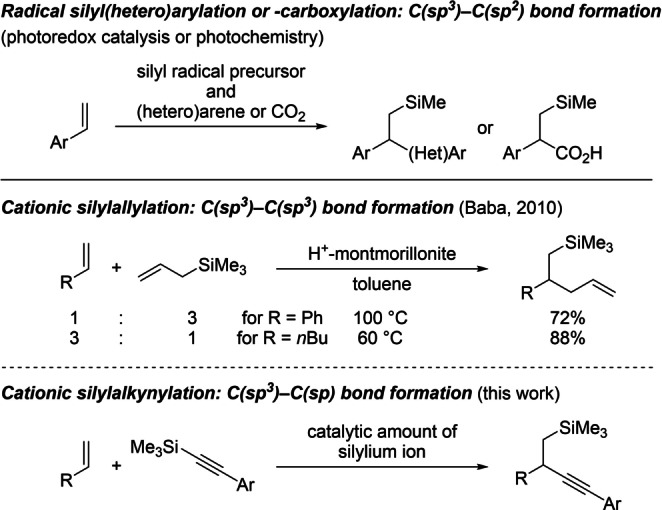
Approaches to carbosilylation of terminal alkenes with C(sp^3^)−C(sp^*n*^) bond formation (*n*=3–1).

We embarked on our investigation with unactivated alkene **1 a** and alkynylsilane **2 a** as model substrates in various ratios (Table 1). Using 1.0 mol % of [Me_3_Si(HCB_11_H_5_Br_6_)] as initiator, different arene solvents were screened. To our delight, the silylalkynylation product **3 aa** was already observed when running the reaction with equimolar quantities of the reactants in chlorobenzene at room temperature for 2 h (entry 1). The yield improved with more silylated phenylacetylene **2 a**, reaching an optimum with a two‐fold excess (entries 2–4). Comparable yields were obtained in other halogenated arene solvents such as 1,2‐dichlorobenzene and fluorobenzene (entries 5 and 6) whereas the yield was significantly lower in benzene and only 13 % in electron‐rich toluene, respectively (entries 7 and 8). Extending the reaction time to 16 h had a marginal effect, both at room temperature and 50 °C (entries 9 and 10).

**Table 1:** Optimization of the silylium‐ion‐initiated silylalkynylation of α‐olefins.^[a]^

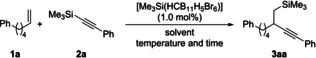					
Entry	Alkynylsilane (**2 a**; equiv)	Solvent	*T* [°C]	*t* [h]	Yield [%]^[b]^
1	1.0	PhCl	RT	2	38
2	1.2	PhCl	RT	2	50
3	2.0	PhCl	RT	2	59
4	3.0	PhCl	RT	2	59
5	2.0	1,2‐C_6_H_4_Cl_2_	RT	2	57
6	2.0	PhF	RT	2	50
7	2.0	benzene	RT	2	35
8	2.0	toluene	RT	2	13
9	2.0	PhCl	RT	16	64 (49)^[c]^
10	2.0	PhCl	50	16	63

[a] All reactions were performed with the α‐olefin **1 a** (0.10 mmol, 1.0 equiv) and the indicated amount of alkynylsilane **2 a** under argon atmosphere in the indicated solvent (0.25 mL, 0.4 M) at room temperature (with one exception). [b] Yields were determined by NMR spectroscopy using CH_2_Br_2_ as an internal standard. [c] The isolated yield of analytically pure product was obtained after flash chromatography on silica gel and is given in parentheses.

With the optimized protocol in hand (Table 1, entry 9), we then gauged the substrate scope (Schemes 2–5). A series of silylated phenylacetylene derivatives **2 a**–**n** were subjected to the general procedure (Scheme 2); the model reaction was performed on a 1.2‐mmol scale, and the yield was slightly higher than that obtained on the routinely employed 0.20‐mmol scale. Compared to the formation of the silylalkynylation product **3 aa**, the yields for methylated substrates were in the same range independent of the position at the aryl group (**3 ab**–**ae**). Good results were also seen for *n*‐propyl and *tert*‐butyl as well as trimethylsilyl substitution (**3 af**–**ah**). Lower yields were consistently found for halogenated derivatives (**3 ai**–**al**). Lewis basic, especially oxygen‐containing functional groups are not tolerated. The corresponding silylated alkynes bearing a naphthyl group reacted smoothly, affording **3 am** with the bulkier naphth‐1‐yl group in lower yield than less hindered **3 an**.

**Scheme 2:**
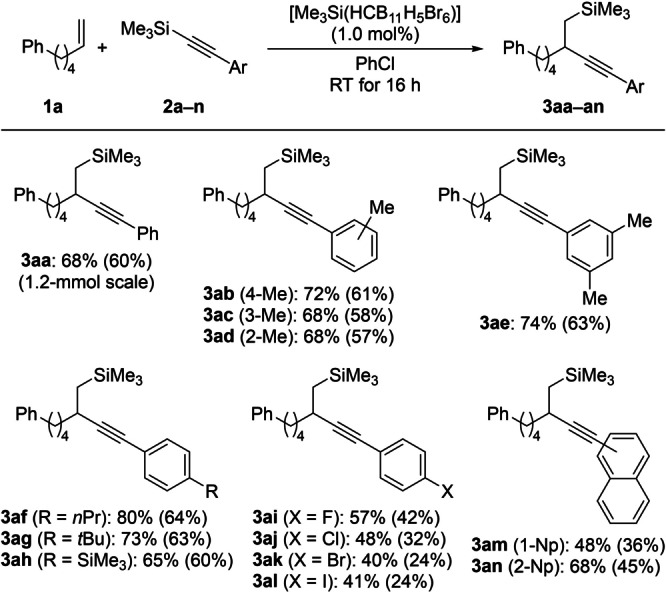
Scope I: Variation of the aryl group in the silylated phenylacetylene derivative. All reactions were performed with alkene **1 a** (0.20 mmol, 1.0 equiv), alkynylsilane **2** (0.40 mmol, 2.0 equiv), and [Me_3_Si(HCB_11_H_5_Br_6_)] (2.0 μmol, 1.0 mol %) under argon atmosphere in chlorobenzene (0.5 mL, 0.4 M) for 16 h. Yields were determined by ^1^H NMR spectroscopy using CH_2_Br_2_ as an internal standard. Yields of analytically pure material were obtained after flash chromatography on silica gel and are given in parentheses. Np=naphthyl.

Aside from **1 a** with a phenyl‐terminated long aliphatic chain, related α‐olefins **1 b**–**d** with shorter alkyl tethers were tested (Scheme 3, top). When alkene **1 b** was reacted with **2 a** following the standard protocol, the expected silylalkynylated product **3 ba** was found in only 16 % yield; instead, the cyclic carbosilylation product **4 ba** involving an intramolecular Friedel–Crafts alkylation was detected in 38 % yield. This result is in agreement with the Friedel–Crafts alkylation reported by us where a tetraorganosilane such as Ph–SiEt_3_ acts as a proton scavenger.^[13]^ We think that Ph−C≡C−SiMe_3_ fulfills the same role.^[14]^ Further shortening the carbon chain by another methylene unit did not impede the Friedel–Crafts reaction pathway. Alkene **1 c** failed to deliver the corresponding silylalkynylation product **3 ca**. Another intramolecular carbosilylation product **5 ca** formed in 36 % yield. This reaction outcome can be rationalized by an anti‐Markovnikov‐selective ring‐opening of an alkene‐coordinated silylium ion in an intramolecular Friedel–Crafts reaction. Subjecting less nucleophilic allylbenzene (**1 d**) to the standard setup was the next logical step. However, hardly any conversion was seen, and **1 d** was recovered in 87 %. Unlike Baba's silylallylation (cf. Scheme 1, center),^[6a]^ styrene oligomerized under our reaction conditions (not shown). Replacing the phenyl group in **1 d** by a cyclohexyl group was also unsuccessful (Scheme 3, bottom). Alkene **1 e** did not afford the silylalkynylation product **3 ea** but the spiro compound **6 ea** in low yield. The formation of **6 ea** can be explained by cationic cascades involving hydrogen shifts or an intramolecular C(sp^3^)−H insertion^[15]^ of a vinyl cation derived from the elusive silylalkynylation product **3 ea** in the superacidic medium. Additional α‐olefins **1 f**–**h** with no aryl group in the proximity of the double bond did participate in the silylalkynylation in good yields (Scheme 4).

**Scheme 3:**
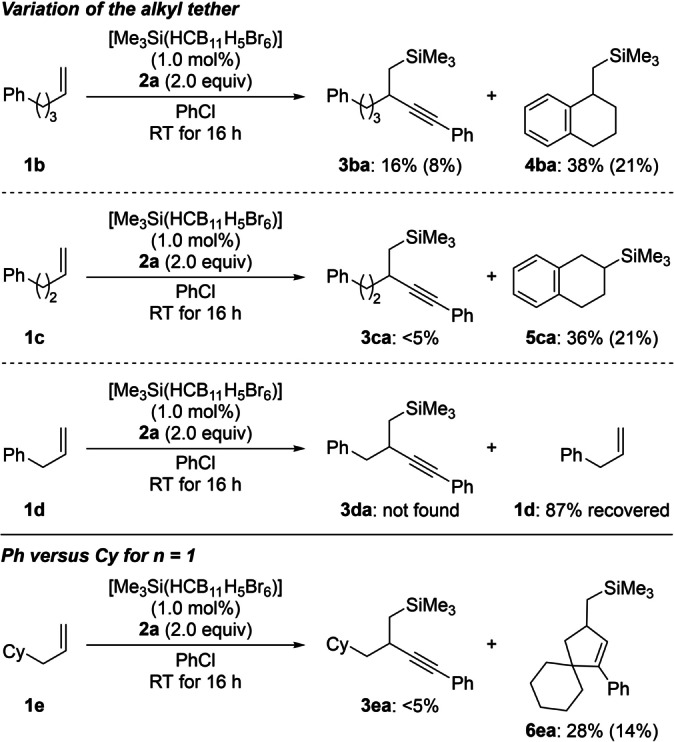
Scope II: Variation of the α‐olefin. Cy=cyclohexyl.

**Scheme 4:**
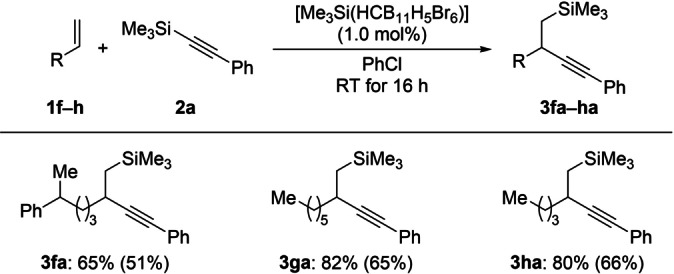
Scope III: Other α‐olefins.

The structure of the alkynylsilane **2** is crucial for the success of this carbosilylation (Scheme 5). Phenylacetylene bearing a trimethylsilyl group gave the best results, and **2 a** was used throughout this study. Increasing the steric demand of the silyl group lead to erosion of the yield for **2 o** (Et_3_Si) and **2 p** (Me_2_PhSi) and to no reaction for **2 q** (*i*Pr_3_Si). Scrambling of the silyl groups in cases where initiator and alkynylsilane are different, e.g. [Me_3_Si(HCB_11_H_5_Br_6_)] and **2 p** with Me_2_PhSi, could not be completely ruled out because reactions at higher initiator loadings (10 mol %) were messy. Substituents other than phenyl such as triisopropylsilyl as in **2 r** or *n*‐butyl as in **2 s** were not compatible with the method; enyne **2 t** only led to trace amounts of the product.

**Scheme 5:**
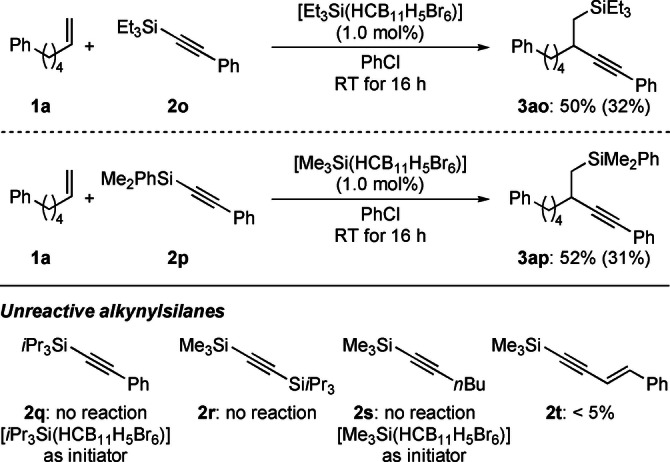
Scope IV: Variation of the alkynylsilane.

To gain mechanistic insight into this silylalkynylation reaction of unactivated alkenes, state‐of‐the‐art dispersion‐corrected DFT calculations were performed at the PW6B95‐D3/def2‐QZVP+COSMO‐RS//TPSS‐D3/def2‐TZVP+COSMO level of theory^[16]^ using [Me_3_Si(HCB_11_H_5_Br_6_)] as the initiator and α‐olefin **1 i** and alkynylsilane **2 a** as representative reactants in PhCl solution. The final free energies in kcal mol^−1^ at 298 K in 1 M concentration in PhCl are used in the discussion. The calculations show that the binding free energies of Me_3_Si^+^ and the available donor molecules increase from −9.0 kcal mol^−1^ for PhCl, −13.0 kcal mol^−1^ for [HCB_11_H_5_Br_6_]^−^, −13.5 kcal mol^−1^ for alkene **1 i** to −23.8 kcal mol^−1^ for alkyne **2 a**. As can be seen in Scheme 6 and Figure 1, the initial Me_3_Si^+^ transfer from [Me_3_Si(HCB_11_H_5_Br_6_)] to **2 a** is −10.8 kcal mol^−1^ exergonic over a low free energy barrier of 10.7 kcal mol^−1^ to form the stable cation **7 a** ^+^ along with the released weakly coordinating anion [HCB_11_H_5_Br_6_]^−^ in PhCl solution. The *C*_2_‐symmetric cation **7 a** ^+^ adopts a ketene‐like carbocation structure with the two geminal silyl groups attached through carbon‐silicon single bonds elongated by 0.11 Å. The necessary stabilization by the phenyl group is consistent with the inertness of alkyl‐substituted alkynes (see **2 s**; Scheme 5, bottom).

**Scheme 6:**
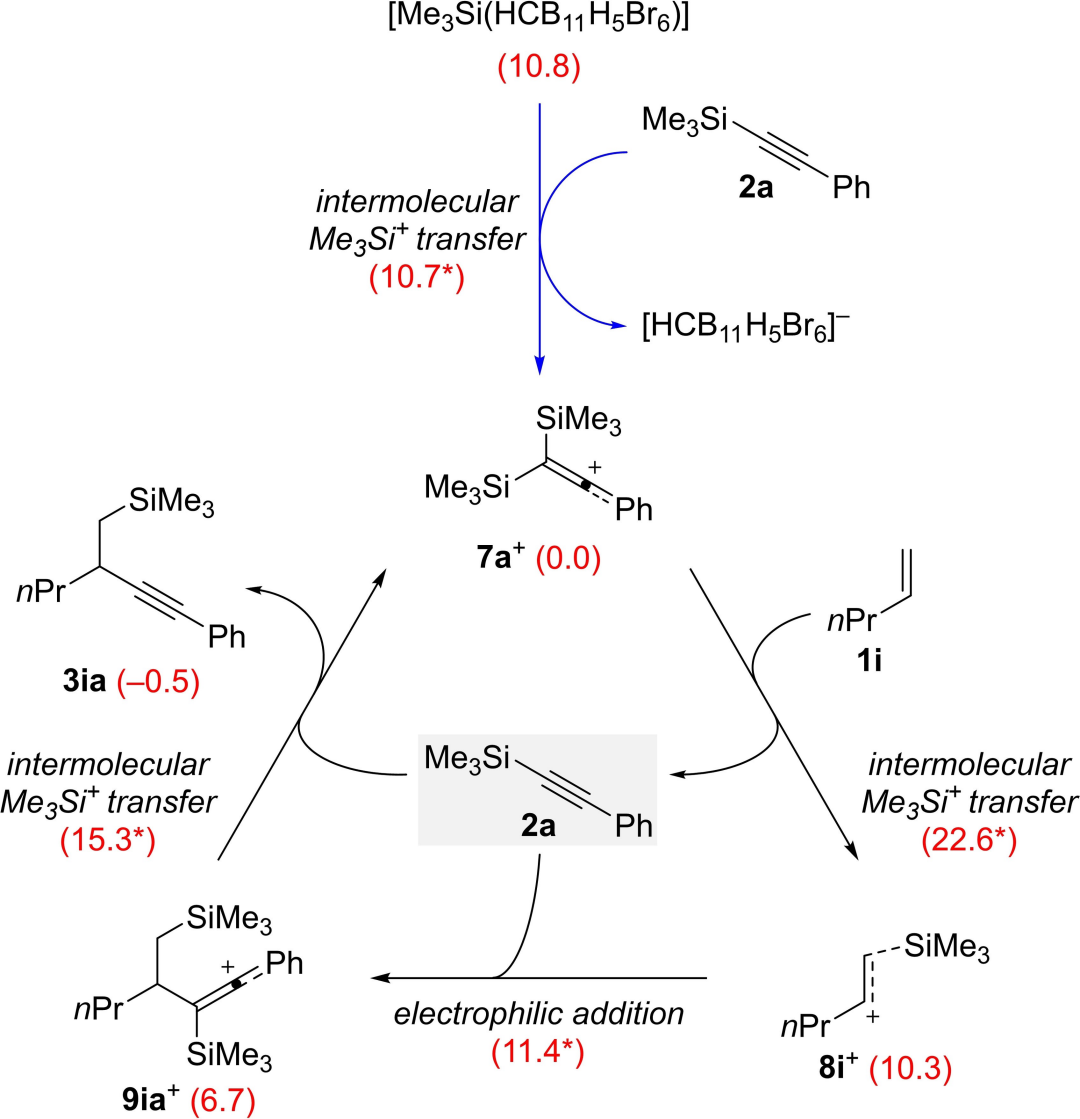
Initiation (highlighted in blue) and computed catalytic cycle of the silylalkynylation of pent‐1‐ene (**1 i**) with silylated phenylacetylene **2 a**. For each reaction step, the Gibbs free reaction energies and barriers (labeled with an asterisk) in kcal mol^−1^ were computed at the PW6B95‐D3 level of theory at 298 K and 1 M concentration in PhCl solution. The counteranion [HCB_11_H_5_Br_6_]^−^ is omitted for clarity when not acting as a stabilizing donor.

**Figure 1:**
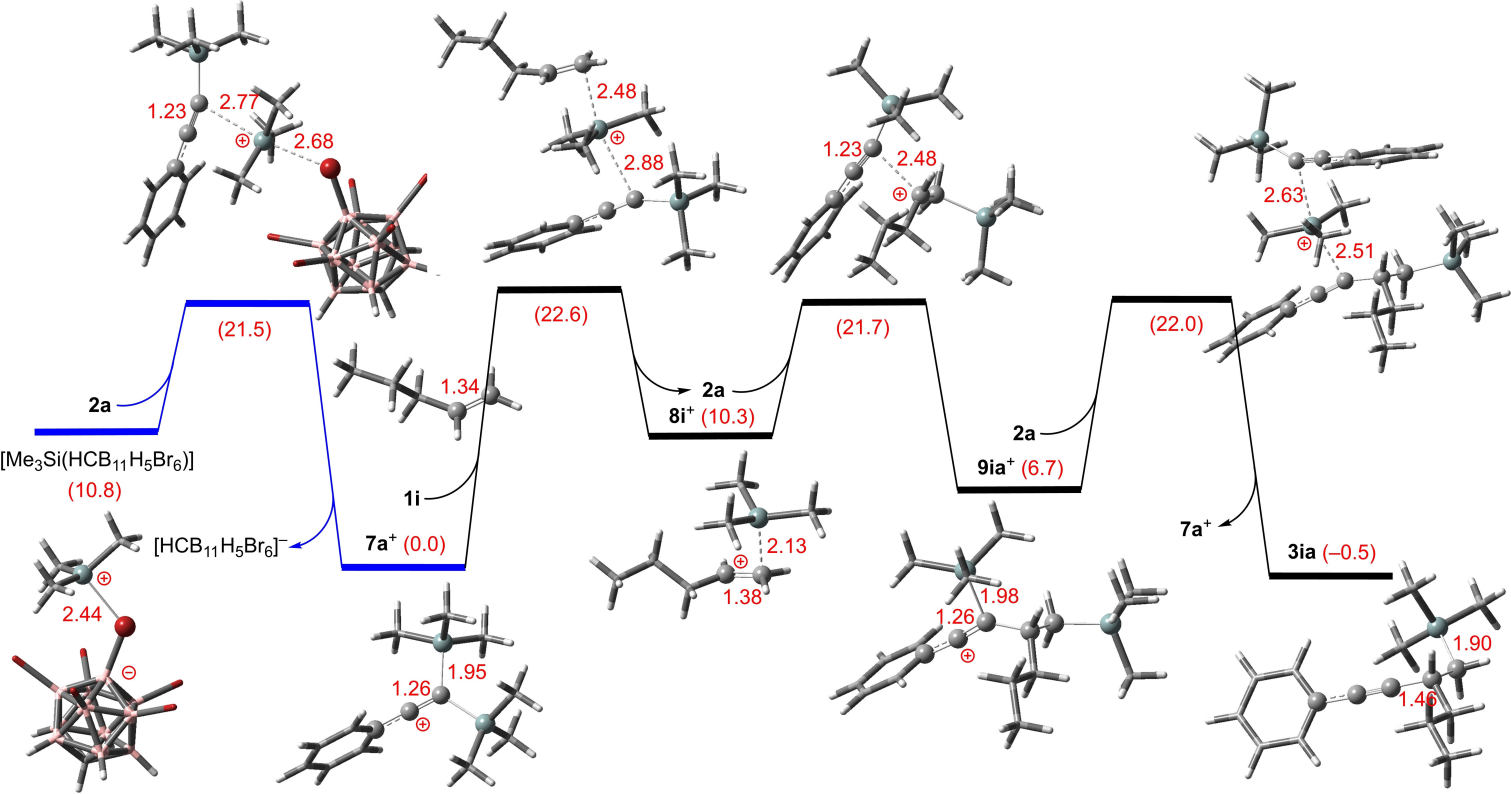
Gibbs free energy profile in kcal mol^−1^ at 298 K and 1 M concentration in PhCl solution of the silylalkynylation of pent‐1‐ene (**1 i**) with silylated phenylacetylene **2 a**. Crucial Br, Si and C atoms are highlighted as red, aqua, and gray balls in the ball‐and‐stick models with selected bond lengths indicated by red numbers in Å.

The subsequent Me_3_Si^+^ transfer from **7 a** ^+^ to the π‐bond of alkene **1 i** is 10.3 kcal mol^−1^ endergonic over a moderate barrier of 22.6 kcal mol^−1^ to form the transient cation **8 i** ^+^ with a longer carbon‐silicon bonding (2.13 Å) and slightly elongated carbon‐carbon double bond (by 0.04 Å). The computed Mulliken charges on the secondary and silylated carbon atoms as well as the Me_3_Si group are 0.40, 0.18, and 0.42 electrons, suggesting a dual reactivity of **8 i** ^+^ either as a silylium ion (for Me_3_Si^+^ transfer) or a carbocation. This notion is supported by the observed intramolecular Friedel–Crafts reactions of aryl groups tethered to the alkene (cf. Scheme 3). Importantly, nucleophilic addition of alkene **1 i** to cation **7 a** ^+^ is 21.9 kcal mol^−1^ endergonic over a sizable barrier of 24.9 kcal mol^−1^ and, hence, is kinetically less favorable by 2.3 kcal mol^−1^ than the Me_3_Si^+^ transfer from **7 a** ^+^ to **1 i** (see the Supporting Information for details).

Starting from cation **8 i** ^+^, the selective addition of the silyl‐substituted carbon atom of alkyne **2 a** to the secondary carbon atom of **8 i** ^+^ is −3.6 kcal mol^−1^ exergonic over a low free energy barrier of 11.4 kcal mol^−1^ (21.7 kcal mol^−1^ with respect to the stable cation **7 a** ^+^) to form the new cation **9 ia** ^+^. Again, **9 ia** ^+^ is stabilized by the phenyl group and adopts a ketene‐like structure with a carbon‐silicon single bond elongated by 0.14 Å. The computed Mulliken charges on the former alkyne carbon atoms and the Me_3_Si group are 0.34, 0.32, and 0.34 electrons, thereby indicating that **9 ia** ^+^ can both release Me_3_Si^+^ and react as a carbon electrophile. Further Me_3_Si^+^ transfer from **9 ia** ^+^ to the alkyne **2 a** is −7.2 kcal mol^−1^ exergonic over a low free energy barrier of 15.3 kcal mol^−1^ (22.0 kcal mol^−1^ with respect to the stable cation **7 a** ^+^) to regenerate the cation **7 a** ^+^ as the actual catalyst along with the final product **3 ia**. The overall catalytic silylalkynylation reaction is −0.5 kcal mol^−1^ exergonic over a barrier of 22.6 kcal mol^−1^ for the step from **7 a** ^+^ to **8 i** ^+^.^[17]^

In summary, we described here a new carbofunctionalization of unactivated alkenes for the formation of a C(sp^3^)−C(sp) and a C(sp^3^)−Si bond in one synthetic operation.^[12]^ This silylalkynylation makes use of silylated phenylacetylene derivatives as the source of the carbon nucleophile and the silicon electrophile^[18]^ and is as such atom‐economic. While initiated by 1.0 mol % of a small, counteranion‐stabilized silylium ion, the catalytic cycle is maintained by an in situ‐generated, bis(silylated) ketene‐like carbocation. The computed mechanism supports this mechanistic picture and also helps understanding the limitations of the methodology.

## Conflict of interest

The authors declare no conflict of interest.

## Supporting information

As a service to our authors and readers, this journal provides supporting information supplied by the authors. Such materials are peer reviewed and may be re‐organized for online delivery, but are not copy‐edited or typeset. Technical support issues arising from supporting information (other than missing files) should be addressed to the authors.

Supporting Information

## Data Availability

The data that support the findings of this study are available from the corresponding author upon reasonable request.
