# Supplementary material for: Intermolecular Carbosilylation of α‐Olefins with C(sp3)−C(sp) Bond Formation Involving Silylium‐Ion Regeneration
Source: Angew Chem Int Ed Engl. 2022 Apr 19;61(24):e202203347. doi: 10.1002/anie.202203347 (PMC9321976; doi:10.1002/anie.202203347)
Supplement: Supplementary file 1 — Supporting Information [file ANIE-61-0-s001.pdf]

Angewandte Chemie
Eine Zeitschrift der Gesellschaft Deutscher Chemiker

Supporting Information

Intermolecular Carbosilylation of α-Olefins with C(sp^3)–C(sp) Bond Formation Involving Silylium-Ion Regeneration

T. He, Z.-W. Qu, H. F. T. Klare, S. Grimme, M. Oestreich**

Table of Contents

**1**	**General Information**	**S3**
**2**	**Experimental Details for the Synthesis of Various Substituted Alkynylsilanes (GP 1–2) and Alkenes**	**S4**
2.1	Methods for the Synthesis of Substituted Alkynylsilanes (**GPs 1** and **2**)	S4
2.2	Characterization Data of Substituted Alkynylsilanes **2**	S5
2.3	Experimental Details for the Synthesis of Alkenes	S13
**3**	**Experimental Details for the Silylium-Ion-Initiated Silylalkynylation of Alkenes**	**S16**
3.1	General Procedure for the Silylium-Ion-Initiated Silylalkynylation of Alkenes (**GP 3**)	S16
3.2	Characterization Data of Products **3aa**–**ap**, **3ba**, and **3fa**–**ha**	S16
3.3	Characterization Data of Products **4ba**, **5ca**, and **6ea**	S27
**4**	**NMR Spectra**	**S29**
**5**	**Computational Data**	**S99**
**6**	**References**	**S130**

1 General Information

All reactions were performed in flame-dried glassware using an *MBraun* glovebox or conventional Schlenk techniques under a static pressure of argon (glovebox) or nitrogen (fume hood) unless otherwise stated. Standard solvents and reagents were obtained from commercial suppliers and used as received unless otherwise stated. Technical grade solvents for extraction and chromatography were distilled prior to use. Dichloromethane (CH_2Cl_2) and tetrahydrofurane (THF) were dried over calcium hydride and sodium, respectively, and freshly distilled prior to use. Dry benzene (C_6H_6), and *n*-pentane were obtained from an *MBraun* solvent purification system (SPS-800), degassed by three freeze-pump-thaw cycles, and stored in a glovebox over thermally activated 4 Å molecular sieves. Toluene (C_6H_7) was dried over CaH_2, distilled, degassed by three freeze-pump-thaw cycles, and stored in a glovebox over thermally activated 4 Å molecular sieves. Fluorobenzene (C_6H_5F), chlorobenzene (C_6H_5Cl), 1,2-dichlorobenzene (1,2-$C_6H_4Cl_2$) were dried over CaH_2, distilled, degassed by three freeze-pump-thaw cycles, and stored in a glovebox over thermally activated 4 Å molecular sieves. Trityl salt $[Ph_3C]^+[HCB_{11}H_5Br_5]^-$[S1] and silylium carborates $[Me_3Si][HCB_{11}H_5Br_6]$,[S2] $[Et_3Si][HCB_{11}H_5Br_6]$,[S1, S3] [*i*$Pr_3Si][HCB_{11}H_5Br_6]$[S4] were synthesized according to reported procedures. Analytical thin-layer chromatography (TLC) was performed on silica gel 60 F254 glass plates. Flash column chromatography was performed on silica gel 60 (40–63 µm, 230–400 mesh ASTM) by *VWR Chemicals* using the indicated solvents. ^{1}H, ^{13}C, ^{19}F, and ^{29}Si NMR spectra were recorded in $CDCl_3$ on *Bruker* AV500 instrument. Chemical shifts are reported in parts per million (ppm) and are referenced to the residual solvent resonance as the internal standard ($CHCl_3$: δ = 7.26 ppm for ^{1}H NMR and $CDCl_3$: δ = 77.16 ppm for ^{13}C NMR). ^{19}F and ^{29}Si NMR spectra are referenced in compliance with the unified scale for NMR chemical shifts as recommended by the IUPAC stating the chemical shift relative to CCl_3F and TMS, respectively.[S5] Data are reported as follows: chemical shift, multiplicity (s = singlet, d = doublet, t = triplet, q = quartet, m = multiplet), coupling constants (Hz), and integration. Infrared (IR) spectra were recorded on an *Agilent Technologies* Cary 630 FT-IR spectrometer equipped with an ATR unit or a *Jasco* FT/IR-4100 spectrometer, and the signals are reported in wavenumbers (cm^{-1}). High resolution mass spectra (HRMS) were obtained from the *Laboratory of Mass Spectrometry* at the *Institut für Chemie*, *Technische Universität Berlin*.

2 Experimental Details for the Synthesis of Various Substituted Alkynylsilanes (GPs 1 and 2) and Alkenes

2.1 Methods for the Synthesis of Substituted Alkynylsilanes (GP 1–2)

2.1.1 Method for the Synthesis of Substituted Alkynylsilanes (**GP 1**)

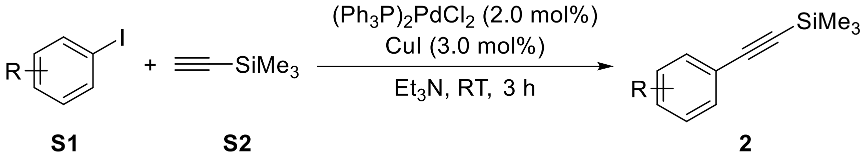

According to a reported procedure,[S6] a solution of the iodoarene **S1** (5.0 mmol, 1.0 equiv) and ethynyltrimethylsilane (**S2**, 589.3 mg, 6.0 mmol, 1.2 equiv) in Et_3N (10 mL) is added dropwise to a suspension of $(Ph_3P)_2PdCl_2$ (70.2 mg, 0.10 mmol, 2.0 mol%) and CuI (28.6 mg, 0.15 mmol, 3.0 mol%) in Et_3N (5 mL). The reaction mixture was maintained for 3 h at ambient temperature. The reaction was then quenched with saturated aqueous NH_4Cl solution (15 mL) and HCl (1.0 M, 10 mL). The resulting mixture is extracted with EtOAc (3 × 20 mL). The combined organic phases are dried over $MgSO_4$ and concentrated under reduced pressure. Purification of the residue by flash column chromatography on silica gel using cyclohexane afforded the aryl-substituted alkynylsilane **2** in analytically pure form.

2.1.2 Method for the Synthesis of Substituted Alkynylsilanes (**GP 2**)

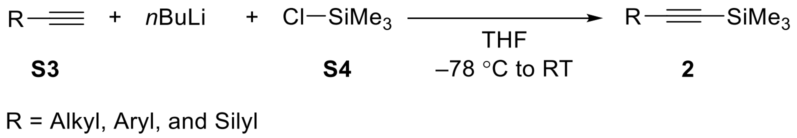

According to a reported procedure,[S7] *n*BuLi (2.2 mL of a 2.5 M solution in *n*-hexane, 5.5 mmol, 1.1 equiv) is added dropwise to a solution of alkyne **S3** (5.0 mmol, 1.0 equiv) in anhydrous THF (15 mL) at –78 °C. The mixture is stirred at this temperature for additional 30 min, and then chlorotrimethylsilane (**S4**, 762 μL, 6.0 mmol, 1.2 equiv) is added. The reaction mixture is allowed to warm to ambient temperature and stirred for additional 2 h. The reaction is quenched by the addition of saturated aqueous NH_4Cl solution (20 mL). The mixture is extracted with EtOAc (3 × 20 mL), dried over $MgSO_4$ and concentrated under reduced pressure. The residue is purified by flash column chromatography using *n*-pentane as eluent to afford the alkylnylsilane **2** in analytically pure form.

2.2 Characterization Data of Substituted Alkynylsilanes 2

2.2.1 Trimethyl(phenylethynyl)silane (**2a**)

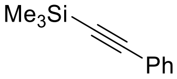

2a
$C_{11}H_{14}Si$
M = 174.32 g/mol

Prepared from iodobenzene (1.0 g, 5.0 mmol, 1.0 equiv) and ethynyltrimethylsilane (**S2**, 589.3 mg, 6.0 mmol, 1.2 equiv) according to **GP 1**. Flash column chromatography on silica gel using cyclohexane afforded **2a** as a pale yellow oil (787.1 mg, 90% yield). **R*f*** = 0.45 (cyclohexane). **^{1}H NMR** (500 MHz, $CDCl_3$, 298 K): δ = 7.48–7.45 (m, 2H), 7.33–7.27 (m, 3H), 0.25 (s, 9H) ppm. **^{13}C{^{1}H} NMR** (126 MHz, $CDCl_3$, 298 K): δ = 132.1, 128.6, 128.3, 123.3, 105.2, 94.2, 0.1 ppm. **^{1}H/^{29}Si HMQC NMR** (500/99 MHz, $CDCl_3$, 298 K, optimized for *J* = 7 Hz): δ = 0.25/–17.7 ppm. The NMR spectroscopic data are in accordance with those reported.[S7]

2.2.2 Trimethyl(*p*-tolylethynyl)silane (**2b**)

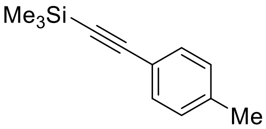

2b
$C_{12}H_{16}Si$
M = 188.35 g/mol

Prepared from 1-iodo-4-methylbenzene (1.1 g, 5.0 mmol, 1.0 equiv) and ethynyltrimethylsilane (**S2**, 589.3 mg, 6.0 mmol, 1.2 equiv) according to **GP 1**. Flash column chromatography on silica gel using cyclohexane afforded **2b** as a pale yellow oil (800.5 mg, 85% yield). **R*f*** = 0.45 (cyclohexane). **^{1}H NMR** (500 MHz, $CDCl_3$, 298 K): δ = 7.39–7.33 (m, 2H), 7.13–7.08 (m, 2H), 2.34 (s, 3H), 0.25 (s, 9H) ppm. **^{13}C{^{1}H} NMR** (126 MHz, $CDCl_3$, 298 K): δ = 138.8, 132.0, 129.1, 120.2, 105.5, 93.4, 21.6, 0.2 ppm. **^{1}H/^{29}Si HMQC NMR** (500/99 MHz, $CDCl_3$, 298 K, optimized for *J* = 7 Hz): δ = 0.25/–18.3 ppm. The NMR spectroscopic data are in accordance with those reported.[S7]

2.2.3 Trimethyl(*m*-tolylethynyl)silane (**2c**)

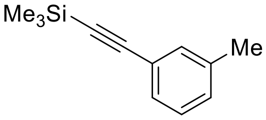

2c
$C_{12}H_{16}Si$
M = 188.35 g/mol

Prepared from 1-iodo-3-methylbenzene (1.1 g, 5.0 mmol, 1.0 equiv) and ethynyltrimethylsilane (**S2**, 589.3 mg, 6.0 mmol, 1.2 equiv) according to **GP 1**. Flash column chromatography on silica gel using cyclohexane afforded **2c** as a yellow oil (696.8 mg, 74% yield). **R*f*** = 0.45

(cyclohexane). **^{1}H NMR** (500 MHz, $CDCl_3$, 298 K): δ = 7.32–7.25 (m, 2H), 7.18 (dd, *J* = 7.7, 7.6 Hz, 1H), 7.12 (d, *J* = 7.7 Hz, 1H), 2.32 (s, 3H), 0.25 (s, 9H) ppm. **^{13}C{^{1}H} NMR** (126 MHz, $CDCl_3$, 298 K): δ = 138.0, 132.7, 129.5, 129.2, 128.2, 123.0, 105.5, 93.8, 21.3, 0.1 ppm. **^{1}H/^{29}Si HMQC NMR** (500/99 MHz, $CDCl_3$, 298 K, optimized for *J* = 7 Hz): δ = 0.25/–18.1 ppm. The NMR spectroscopic data are in accordance with those reported.[S8]

2.2.4 Trimethyl(*p*-tolylethynyl)silane (**2d**)

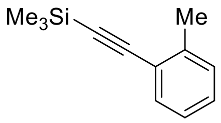

2d
$C_{12}H_{16}Si$
M = 188.35 g/mol

Prepared from 1-iodo-2-methylbenzene (1.1 g, 5.0 mmol, 1.0 equiv) and ethynyltrimethylsilane (**S2**, 589.3 mg, 6.0 mmol, 1.2 equiv) according to **GP 1**. Flash column chromatography on silica gel using cyclohexane afforded **2d** as a yellow oil (679.7 mg, 72% yield). **R_f** = 0.46 (cyclohexane). **^{1}H NMR** (500 MHz, $CDCl_3$, 298 K): δ = 7.43 (d, *J* = 7.6 Hz, 1H), 7.24–7.16 (m, 2H), 7.15–7.08 (m, 1H), 2.44 (s, 3H), 0.26 (s, 9H) ppm. **^{13}C{^{1}H} NMR** (126 MHz, $CDCl_3$, 298 K): δ = 140.7, 132.2, 129.5, 128.6, 125.6, 123.0, 104.2, 98.3, 20.8, 0.2 ppm. **^{1}H/^{29}Si HMQC NMR** (500/99 MHz, $CDCl_3$, 298 K, optimized for *J* = 7 Hz): δ = 0.26/–18.0 ppm. The NMR spectroscopic data are in accordance with those reported.[S9]

2.2.5 ((3,5-Dimethylphenyl)ethynyl)trimethylsilane (**2e**)

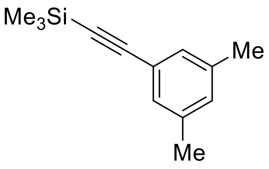

2e
$C_{13}H_{18}Si$
M = 202.37 g/mol

Prepared from 1-iodo-3,5-dimethylbenzene (1.2 g, 5.0 mmol, 1.0 equiv) and ethynyltrimethylsilane (**S2**, 589.3 mg, 6.0 mmol, 1.2 equiv) according to **GP 1**. Flash column chromatography on silica gel using cyclohexane afforded **2e** as a pale yellow oil (809.5 mg, 80% yield). **R_f** = 0.45 (cyclohexane). **^{1}H NMR** (500 MHz, $CDCl_3$, 298 K): δ = 7.11 (s, 2H), 6.95 (s, 1H), 2.28 (s, 6H), 0.25 (s, 9H) ppm. **^{13}C{^{1}H} NMR** (126 MHz, $CDCl_3$, 298 K): δ = 137.9, 130.5, 129.8, 122.8, 105.7, 93.4, 21.2, 0.2 ppm. **^{1}H/^{29}Si HMQC NMR** (500/99 MHz, $CDCl_3$, 298 K, optimized for *J* = 7 Hz): δ = 0.25/–17.9 ppm. The NMR spectroscopic data are in accordance with those reported.[S11]

2.2.6 Trimethyl((4-propylphenyl)ethynyl)silane (**2f**)

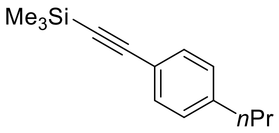

2f

$C_{14}H_{20}Si$

M = 216.40 g/mol

Prepared from 1-ethynyl-4-propylbenzene (721.0 mg, 5.0 mmol, 1.0 equiv) and chlorotrimethylsilane (**S2**, 1.1 g, 6.0 mmol, 2.0 equiv) according to **GP 2**. Flash column chromatography on silica gel using cyclohexane afforded **2f** as a colorless oil (1.0 g, 93% yield). **R_f** = 0.60 (*n*-hexane). **^{1}H NMR** (500 MHz, $CDCl_3$, 298 K): δ = 7.38 (d, *J* = 8.1 Hz, 2H), 7.10 (d, *J* = 8.1 Hz, 2H), 2.57 (t, *J* = 7.4 Hz, 2H), 1.62 (tq, *J* = 7.4 Hz, 2H), 0.92 (t, *J* = 7.4 Hz, 3H), 0.24 (s, 9H) ppm. **^{13}C{^{1}H} NMR** (126 MHz, $CDCl_3$, 298 K): δ = 143.5, 132.0, 128.5, 120.4, 105.5, 93.4, 38.1, 24.4, 13.8, 0.2 ppm. **^{1}H/^{29}Si HMQC NMR** (500/99 MHz, $CDCl_3$, 298 K, optimized for *J* = 7 Hz): δ = 0.24/–18.2 ppm. The NMR spectroscopic data are in accordance with those reported.[S10]

2.2.7 ((4-(*Tert*-butyl)phenyl)ethynyl)trimethylsilane (**2g**)

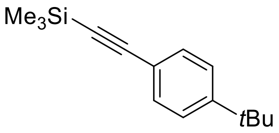

2g

$C_{15}H_{22}Si$

M = 230.43 g/mol

Prepared from 1-(*tert*-butyl)-4-iodobenzene (1.3 g, 5.0 mmol, 1.0 equiv) and ethynyltrimethylsilane (**S2**, 589.3 mg, 6.0 mmol, 1.2 equiv) according to **GP 1**. Flash column chromatography on silica gel using cyclohexane afforded **2g** as a yellow oil (1.0 g, 87% yield). **R_f** = 0.31 (*n*-hexane). **^{1}H NMR** (500 MHz, $CDCl_3$, 298 K): δ = 7.42–7.37 (m 2H), 7.33–7.29 (m, 2H), 1.30 (s, 9H), 0.24 (s, 9H) ppm. **^{13}C{^{1}H} NMR** (126 MHz, $CDCl_3$, 298 K): δ = 151.9, 131.8, 125.3, 120.2, 105.5, 93.4, 34.9, 31.3, 0.2 ppm. **^{1}H/^{29}Si HMQC NMR** (500/99 MHz, $CDCl_3$, 298 K, optimized for *J* = 7 Hz): δ = 0.24/–18.3 ppm. The NMR spectroscopic data are in accordance with those reported.[S8]

2.2.8 Trimethyl(4-((trimethylsilyl)ethynyl)phenyl)silane (**2h**)

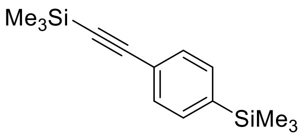

2h

$C_{14}H_{22}Si_2$

M = 246.50 g/mol

To a solution of ((4-bromophenyl)ethynyl)trimethylsilane (1.0 g, 4.0 mmol, 1.0 equiv, see section 2.2.11 for its preparation) in anhydrous THF (10 mL), *n*BuLi (4.8 mmol, 1.9 mL of a 2.5 M solution in *n*hexane, 1.2 equiv) was added dropwise at –78 °C. After stirring for additional 30 min at this temperature, chlorotrimethylsilane (**S4**, 869 mg, 8.0 mmol, 2.0 equiv) was added. The mixture was allowed to warm to room temperature and stirred for additional 2 h. The mixture was quenched with water (10 mL) and saturated aqueous NH_4Cl solution (10 mL). The organic phase was separated, and the aqueous layer was extracted with EtOAc (3 × 10 mL). The combined organic phases were dried over $MgSO_4$ and concentrated under reduced pressure. Purification of the residue by flash column chromatography on silica gel using cyclehexane afforded **2h** as a colorless oil (725.0 mg, 76%). **R*f*** = 0.45 (*n*-hexane). **^{1}H NMR** (500 MHz, $CDCl_3$, 298 K): δ = 7.46–7.41 (m, 4H), 0.25 (s, 9H), 0.25 (s, 9H) ppm. **^{13}C{^{1}H} NMR** (126 MHz, $CDCl_3$, 298 K): δ = 141.5, 133.2, 131.1, 123.5, 105.4, 94.6, 0.1, –1.1 ppm. **^{1}H/^{29}Si HMQC NMR** (500/99 MHz, $CDCl_3$, 298 K, optimized for J = 7 Hz): δ = 0.25/–4.1, 0.25/–17.9 ppm. **HRMS** (APCI): calculated for $C_{14}H_{22}Si_2^{•+}$ [M]$^{•+}$: 246.1260; found 246.1255.

2.2.9 ((4-Fluorophenyl)ethynyl)trimethylsilane (**2i**)

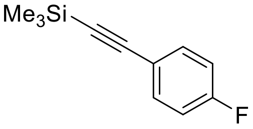

2i
$C_{11}H_{13}FSi$
M = 192.31 g/mol

Prepared from 1-ethynyl-4-fluorobenzene (600.6 mg, 5.0 mmol, 1.0 equiv) and chlorotrimethylsilane (**S2**, 1.1 g, 2.0 equiv) according to **GP 2**. Flash column chromatography on silica gel using cyclohexane afforded **2i** as a colorless oil (860 mg, 89% yield). **R*f*** = 0.60 (*n*-hexane). **^{1}H NMR** (500 MHz, $CDCl_3$, 298 K): δ = 7.48–7.41 (m, 2H), 7.02–6.95 (m, 2H), 0.24 (s, 9H) ppm. **^{13}C{^{1}H} NMR** (126 MHz, $CDCl_3$, 298 K): δ = 162.7 (d, $J_{C,F}$ = 250.3 Hz), 134.0 (d, $J_{C,F}$ = 8.2 Hz), 119.4 (d, $J_{C,F}$ = 3.9 Hz), 115.6 (d, $J_{C,F}$ = 22.1 Hz), 104.1, 94.0, 0.1 ppm. **^{1}H/^{29}Si HMQC NMR** (500/99 MHz, $CDCl_3$, 298 K, optimized for J = 7 Hz): δ = 0.24/–18.0 ppm. **^{19}F NMR** (471 MHz, $CDCl_3$, 298 K): δ = –110.5 ppm. The NMR spectroscopic data are in accordance with those reported.[S8]

2.2.10 ((4-Chlorophenyl)ethynyl)trimethylsilane (**2j**)

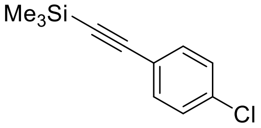

2j
$C_{11}H_{13}ClSi$
M = 208.76 g/mol

Prepared from 1-chloro-4-iodobenzene (1.2 g, 5.0 mmol, 1.0 equiv) and ethynyltrimethylsilane (**S2**, 589.3 mg, 1.2 equiv) according to **GP 1**. Flash column chromatography on silica gel using

cyclohexane afforded **2j** as a white solid (992.0 mg, 95% yield). **R*f*** = 0.45 (cyclohexane). **^{1}H NMR** (500 MHz, $CDCl_3$, 298 K): δ = 7.42–7.36 (m, 2H), 7.29–7.25 (m, 2H), 0.25 (s, 9H) ppm. **^{13}C{^{1}H} NMR** (126 MHz, $CDCl_3$, 298 K): δ = 134.6, 133.3, 128.7, 121.8, 104.0, 95.5, 0.0 ppm. **^{1}H/^{29}Si HMQC NMR** (500/99 MHz, $CDCl_3$, 298 K, optimized for *J* = 7 Hz): δ = 0.25/–17.9 ppm. The NMR spectroscopic data are in accordance with those reported.[S12]

2.2.11 ((4-Bromophenyl)ethynyl)trimethylsilane (**2k**)

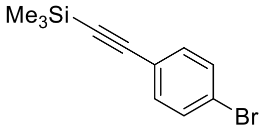

2k

$C_{11}H_{13}BrSi$

M = 253.21 g/mol

Prepared from 1-bromo-4-iodobenzene (1.4 g, 5.0 mmol, 1.0 equiv) and ethynyltrimethylsilane (**S2**, 589.3 mg, 1.2 equiv) according to **GP 1**. Flash column chromatography on silica gel using cyclohexane afforded **2k** as a white solid (1.21 g, 95% yield). **R*f*** = 0.50 (cyclohexane). **^{1}H NMR** (500 MHz, $CDCl_3$, 298 K): δ = 7.45–7.41 (m, 2H), 7.34–7.30 (m, 2H), 0.25 (s, 9H) ppm. **^{13}C{^{1}H} NMR** (126 MHz, $CDCl_3$, 298 K): δ = 133.5, 131.6, 122.9, 122.2, 104.0, 95.7, 0.0 ppm. **^{1}H/^{29}Si HMQC NMR** (500/99 MHz, $CDCl_3$, 298 K, optimized for *J* = 7 Hz): δ = 0.25/–17.7 ppm. The NMR spectroscopic data are in accordance with those reported.[S12]

2.2.12 ((4-Iodophenyl)ethynyl)trimethylsilane (**2l**)

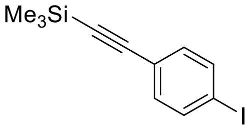

2l

$C_{11}H_{13}ISi$

M = 300.21 g/mol

To a solution of ((4-bromophenyl)ethynyl)trimethylsilane (**2k**, 1.2 g, 4.7 mmol, 1.0 equiv, see section 2.2.11 for its preparation) in anhydrous THF (10 mL), *n*BuLi (5.2 mmol, 2.1 mL of a 2.5 M solution in *n*-hexane, 1.1 equiv) was added dropwise at –78 °C. After stirring for additional 30 min at this temperature, a solution of iodine (3.0 g, 11.9 mmol, 2.5 equiv) in anhydrous THF (10 mL) was added dropwise. The mixture was allowed to warm to room temperature and stirred overnight. The mixture was quenched with water (10 mL) and aqueous $Na_2S_2O_3$ solution (1.0 M, 20 mL). The organic phase was separated, and the aqueous layer was extracted with EtOAc (3 × 15 mL). The combined organic phases were dried over $MgSO_4$ and concentrated under reduced pressure. Purification of the residue by flash column chromatography on silica gel using cyclohexane afforded **2l** as a purple solid, which was further recrystallized from *n*-hexane to obtain a white solid (564.4 mg, 40%). **R*f*** = 0.50 (cyclohexane). **^{1}H NMR** (500 MHz, $CDCl_3$, 298 K): δ = 7.66–7.61 (m, 2H), 7.20–7.16 (m, 2H), 0.24 (s, 9H) ppm. **^{13}C{^{1}H} NMR** (126 MHz, $CDCl_3$, 298 K): δ = 137.5, 133.6, 122.8, 104.1, 96.0, 94.6, 0.0 ppm. **^{1}H/^{29}Si HMQC NMR**

(500/99 MHz, $CDCl_3$, 298 K, optimized for *J* = 7 Hz): δ = 0.24/–17.7 ppm. The NMR spectroscopic data are in accordance with those reported.[S13]

2.2.13 Trimethyl(naphthalen-1-ylethynyl)silane (**2m**)

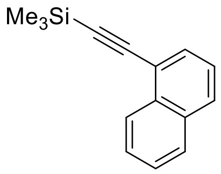

2m
$C_{15}H_{16}Si$
M = 224.38 g/mol

Prepared from 1-ethynylnaphthalene (761 mg, 5.0 mmol, 1.0 equiv) and chlorotrimethylsilane (**S4**, 1.1 g, 10 mmol, 2.0 equiv) according to **GP 2**. Flash column chromatography on silica gel using cyclohexane afforded **2m** as a pale yellow oil (816.0 mg, 73% yield). $\mathbf{R_f}$ = 0.26 (*n*-hexane). ^{1}**H NMR** (500 MHz, $CDCl_3$, 298 K): δ = 8.34 (d, *J* = 8.4 Hz, 1H), 7.86–7.80 (m, 2H), 7.70 (dd, *J* = 7.2, 1.2 Hz, 1H), 7.61–7.55 (m, 1H), 7.54–7.49 (m, 1H), 7.41 (dd, *J* = 8.2, 7.2 Hz, 1H), 0.34 (s, 9H) ppm. ^{13}**C{^{1}H} NMR** (126 MHz, $CDCl_3$, 298 K): δ = 133.6, 133.2, 131.0, 129.1, 128.4, 127.0, 126.5, 126.4, 125.3, 120.9, 103.2, 99.6, 0.3 ppm. ^{1}**H/^{29}Si HMQC NMR** (500/99 MHz, $CDCl_3$, 298 K, optimized for *J* = 7 Hz): δ = 0.34/–17.9 ppm. The NMR spectroscopic data are in accordance with those reported.[S14]

2.2.14 Trimethyl(naphthalen-2-ylethynyl)silane (**2n**)

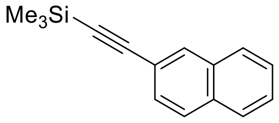

2n
$C_{15}H_{16}Si$
M = 224.38 g/mol

Prepared from 2-bromonaphthalene (828.3 mg, 4.0 mmol, 1.0 equiv) and trimethylsilylacetylene (**S2**, 589.3 mg, 8.0 mmol, 2.0 equiv) according to **GP 1** but at 40 °C for 12 h. Flash column chromatography on silica gel using cyclohexane afforded trimethyl-(naphthalen-2-ylethynyl)silane **2n** as a white solid (510.0 mg, 57% yield). $\mathbf{R_f}$ = 0.40 (*n*-hexane). ^{1}**H NMR** (500 MHz, $CDCl_3$, 298 K): δ = 8.00 (s, 1H), 7.83–7.74 (m, 3H), 7.53–7.46 (m, 3H), 0.29 (s, 9H) ppm. ^{13}**C{^{1}H} NMR** (126 MHz, $CDCl_3$, 298 K): δ = 133.0, 133.0, 132.1, 128.7, 128.0, 127.9, 127.9, 126.9, 126.4, 120.6, 105.6, 94.7, 0.2 ppm. ^{1}**H/^{29}Si HMQC NMR** (500/99 MHz, $CDCl_3$, 298 K, optimized for *J* = 7 Hz): δ = 0.29/–18.0 ppm. The NMR spectroscopic data are in accordance with those reported.[S15]

2.2.15 Triethyl(phenylethynyl)silane (**2o**)

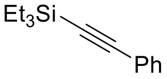

2o
$C_{14}H_{20}Si$
M = 216.40 g/mol

Prepared from ethynylbenzene (510.6 mg, 5.0 mmol, 1.0 equiv) and chlorotriethylsilane (1.5 g, 10 mmol, 2.0 equiv) according to **GP 2**. Flash column chromatography on silica gel using cyclohexane afforded **2o** as a pale yellow oil (1.0 g, 94% yield). **R*f*** = 0.60 (cyclohexane). **^{1}H NMR** (500 MHz, $CDCl_3$, 298 K): δ = 7.50–7.45 (m, 2H), 7.32–7.27 (m, 3H), 1.05 (t, *J* = 7.9 Hz, 9H), 0.68 (q, *J* = 7.9 Hz, 6H) ppm. **^{13}C{^{1}H} NMR** (126 MHz, $CDCl_3$, 298 K): δ = 132.2, 128.5, 128.3, 123.5, 106.5, 91.7, 7.6, 4.6 ppm. **^{1}H/^{29}Si HMQC NMR** (500/99 MHz, $CDCl_3$, 298 K, optimized for *J* = 7 Hz): δ = 1.05/–7.5, 0.68/–7.5 ppm. The NMR spectroscopic data are in accordance with those reported.[S16]

2.2.16 Dimethyl(phenyl)(phenylethynyl)silane (**2p**)

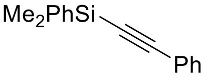

2p
$C_{16}H_{16}Si$
M = 236.39 g/mol

Prepared from phenylacetylene (510.6 mg, 5.0 mmol, 1.0 equiv) and chlorodimethyl(phenyl)-silane (1.0 g, 6.0 mmol, 1.2 equiv) according to **GP 2**. Flash column chromatography on silica gel using cyclohexane afforded **2p** as a colorless oil (945 mg, 80% yield). **R*f*** = 0.40 (cyclohexane). **^{1}H NMR** (500 MHz, $CDCl_3$, 298 K): δ = 7.73–7.68 (m, 2H), 7.53–7.49 (m, 2H), 7.42–7.38 (m, 3H), 7.36–7.28 (m, 3H), 0.50 (s, 6H) ppm. **^{13}C{^{1}H} NMR** (126 MHz, $CDCl_3$, 298 K): δ = 137.2, 133.9, 132.2, 129.6, 128.8, 128.4, 128.0, 123.1, 106.9, 92.2, –0.7 ppm. **^{1}H/^{29}Si HMQC NMR** (500/99 MHz, $CDCl_3$, 298 K, optimized for *J* = 7 Hz): δ = 0.50/–22.0 ppm. The NMR spectroscopic data are in accordance with those reported.[S18]

2.2.17 Triisopropyl(phenylethynyl)silane (**2q**)

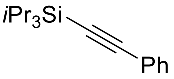

2q
$C_{17}H_{26}Si$
M = 258.48 g/mol

Prepared from ethynylbenzene (510.6 mg, 5.0 mmol, 1.0 equiv) and chlorotriisopropylsilane (1.9 g, 10 mmol, 2.0 equiv) according to **GP 2**. Flash column chromatography on silica gel using cyclohexane afforded **2q** as a colorless oil (1.2 g, 93% yield). **R*f*** = 0.65 (cyclohexane). **^{1}H NMR** (500 MHz, $CDCl_3$, 298 K): δ = 7.50–7.45 (m, 2H), 7.32–7.28 (m, 3H), 1.15 (s, 21 H) ppm. **^{13}C{^{1}H} NMR** (126 MHz, $CDCl_3$, 298 K): δ = 132.2, 128.4, 128.3, 123.7, 107.2, 90.6, 18.8,

11.5 ppm. **^{1}H/^{29}Si HMQC NMR** (500/99 MHz, $CDCl_3$, 298 K, optimized for *J* = 7 Hz): δ = 1.15/–2.2 ppm. The NMR spectroscopic data are in accordance with those reported.[S17]

2.2.18 Triisopropyl((trimethylsilyl)ethynyl)silane (**2r**)

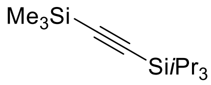

2r
$C_{14}H_{30}Si_2$
M = 254.56 g/mol

Prepared from ethynyltrimethylsilane (491.1 mg, 5.0 mmol, 1.0 equiv) and chlorotriisopropylsilane (1.0 g, 5.0 mmol, 1.0 equiv) according to **GP 2**. Flash column chromatography on silica gel using cyclohexane afforded **2r** as a colorless oil (1.1 g, 86% yield). $\mathbf{R}_f$ = 0.80 (cyclohexane). **^{1}H NMR** (500 MHz, $CDCl_3$, 298 K): δ = 1.10–1.03 (m, 21H), 0.17 (s, 9H) ppm. **^{13}C{^{1}H} NMR** (126 MHz, $CDCl_3$, 298 K): δ = 116.3, 110.3, 18.7, 11.2, 0.2 ppm. **^{1}H/^{29}Si HMQC NMR** (500/99 MHz, $CDCl_3$, 298 K, optimized for *J* = 7 Hz): δ = 1.06/–3.1, 0.17/–3.1 ppm. The NMR spectroscopic data are in accordance with those reported.[S12]

2.2.19 (*E*)-trimethyl(4-phenylbut-3-en-1-yn-1-yl)silane (**2t**)

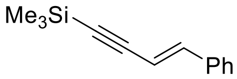

2t
$C_{13}H_{16}Si$
M = 200.36 g/mol

Prepared from (*E*)-(2-bromovinyl)benzene (915.3 mg, 5.0 mmol, 1.0 equiv) and ethynyltrimethylsilane (**S2**, 589.3 mg, 6.0 mmol, 1.2 equiv) according to **GP 1**. Flash column chromatography on silica gel using cyclohexane afforded **2t** as a yellow oil (901.3 g, 90% yield). $\mathbf{R}_f$ = 0.45 (cyclohexane). **^{1}H NMR** (500 MHz, $CDCl_3$, 298 K): δ = 7.39–7.35 (m, 2H), 7.35–7.26 (m, 3H), 7.01 (d, *J* = 16.4 Hz, 1H), 6.18 (d, *J* = 16.4 Hz, 1H), 0.23 (s, 9H) ppm. **^{13}C{^{1}H} NMR** (126 MHz, $CDCl_3$, 298 K): δ = 142.5, 136.3, 128.9, 128.9, 126.4, 108.2, 104.5, 97.0, 0.1 ppm. **^{1}H/^{29}Si HMQC NMR** (500/99 MHz, $CDCl_3$, 298 K, optimized for *J* = 7 Hz): δ = 0.23/–18.3 ppm. The NMR spectroscopic data are in accordance with those reported.[S19]

2.3 Experimental Details for the Synthesis of Alkenes

2.3.1 Experimental Details for the Synthesis of Hex-5-en-1-ylbenzene (**1a**)

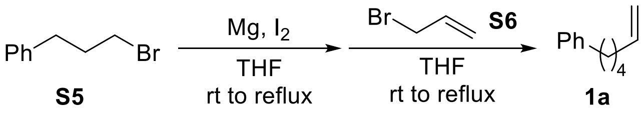

A solution of (3-bromopropyl)benzene (**S5**, 4.0 g, 20 mmol, 1.0 equiv) in anhydrous THF (20 mL) was slowly added to a suspension of magnesium turnings (624 mg, 26 mmol, 1.3 equiv) and iodine (catalytic amounts) in anhydrous THF (5 mL) at room temperature. After complete addition, the mixture was heated to refluxed for 2 h. The thus-obtained solution of (3-phenyl-propyl)magnesium bromide in THF was slowly added to a solution of allyl bromide (**S6**, 3.6 g, 30 mmol, 1.5 equiv) in anhydrous THF (10 mL) at room temperature. After complete addition, the reaction mixture was then stirred for 30 min at room temperature before heated at reflux overnight. The reaction mixture was then quenched by the addition of water (20 mL) and saturated aqueous NH_4Cl (20 mL). The organic phase was separated and the aqueous layer was extracted with EtOAc (3 × 20 mL). The combined organic phases were dried over $MgSO_4$ and concentrated under reduced pressure. Purification of the residue by flash column chromatography on silica gel using cyclohexane as eluent afforded hex-5-en-1-ylbenzene (**1a**) as a colorless oil (2.4 g, 75% yield).

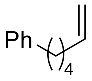

1a
$C_{12}H_{16}$
M = 160.26 g/mol

R*f* = 0.55 (cyclohexane). **^{1}H NMR** (500 MHz, $CDCl_3$, 298 K): δ = 7.31–7.26 (m, 2H), 7.21–7.15 (m, 3H), 5.86–5.76 (m, 1H), 5.03–4.97 (m, 1H), 4.97–4.92 (m, 1H), 2.62 (t, *J* = 7.6 Hz, 2H), 2.12–2.05 (m, 2H), 1.68–1.61 (m, 2H), 1.49–1.41 (m, 2H) ppm. **^{13}C{^{1}H} NMR** (126 MHz, $CDCl_3$, 298 K): δ = 142.8, 139.0, 128.5, 128.4, 125.7, 114.5, 36.0, 33.8, 31.1, 28.7 ppm. The NMR spectroscopic data are in accordance with those reported.[S20]

2.3.2 Experimental Details for the Synthesis of Pent-4-en-1-ylbenzene (**1b**)

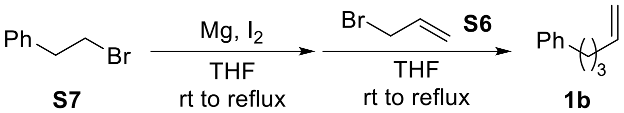

The synthetic procedure for pent-4-en-1-ylbenzene (**1b**) was adopted from that for hex-5-en-1-ylbenzene (**1a**): 57% yield starting from 15 mmol of (2-bromoethyl)benzene (**S7**).

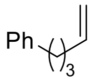

1b

$C_{11}H_{14}$

M = 146.23 g/mol

Colorless oil. **R*f*** = 0.58 (cyclohexane). **¹H NMR** (500 MHz, $CDCl_3$, 298 K): δ = 7.32–7.27 (m, 2H), 7.22–7.17 (m, 3H), 5.90–5.80 (m, 1H), 5.07–5.01 (m, 1H), 5.01–4.97 (m, 1H), 2.64 (t, *J* = 7.6 Hz, 2H), 2.15–2.08 (m, 2H), 1.78–1.70 (m, 2H) ppm. **^{13}C{^{1}H} NMR** (126 MHz, $CDCl_3$, 298 K): δ = 142.6, 138.8, 128.6, 128.4, 125.8, 114.8, 35.5, 33.4, 30.8 ppm. The NMR spectroscopic data are in accordance with those reported.[S20]

2.3.3 Experimental Details for the Synthesis of Hept-6-en-2-ylbenzene (**1f**)

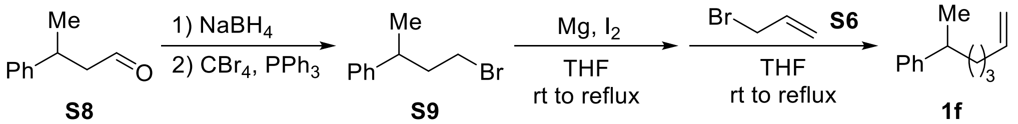

To a solution of 3-phenylbutanal (**S8**, 1.5 g, 10 mmol, 1.0 equiv) in methanol (15 mL), $NaBH_4$ (454 mg, 12 mmol, 1.2 equiv) was added portionwise at 0 °C. After complete addition, the reaction mixture was allowed to warm and stirred for additional 1 h. The reaction mixture was diluted by diethyl ether (20 mL) and then washed water (20 mL) and saturated brine (20 mL). The organic phase was separated and the aqueous layer was extracted with diethyl ether (2 × 20 mL). The combined organic phases were dried over $MgSO_4$ and concentrated under reduced pressure to afford crude 3-phenylbutan-1-ol in quantitive yield which was directly used next without further purification.

To a solution of 3-phenylbutan-1-ol (10 mmol, 1.0 equiv) and tetrabromomethane (3.6 g, 11 mmol, 1.1 equiv) in anhydrous THF (10 mL), a solution of triphenylphosphine (2.9 g, 11 mmol, 1.1 equiv) in anhydrous THF (15 mL) was added dropwise at 0 °C. The reaction mixture was allowed to warm and stirred for additional 2 h. The reaction mixture was quenched by the addition of water (20 mL) and diluted with diethyl ether (20 mL). The organic phase was separated and the aqueous layer was extracted with diethyl ether (3 × 10 mL). The combined organic phases were washed with brine (20 mL), dried over $MgSO_4$ and concentrated under reduced pressure. Purification of the residue by flash column chromatography on silica gel using cyclohexane as eluent afforded (4-bromobutan-2-yl)benzene (**S9**) as a colorless oil (1.5 g, 70% yield). The NMR spectroscopic data are in accordance with those reported.[S21]

The synthetic procedure for hept-6-en-2-ylbenzene (**1f**) from (4-bromobutan-2-yl)benzene (**S9**) was adopted from that for hex-5-en-1-ylbenzene (**1a**): 43% yield starting from 10 mmol of 3-phenylbutanal (**S8**) after 3 steps.

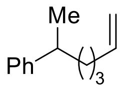

1f

$C_{13}H_{18}$

M = 174.29 g/mol

Colorless oil. **R*f*** = 0.60 (cyclohexane). **^{1}H NMR** (500 MHz, $CDCl_3$, 298 K): δ = 7.32–7.26 (m, 2H), 7.21–7.16 (m, 3H), 5.82–5.71 (m, 1H), 5.00–4.94 (m, 1H), 4.94–4.89 (m, 1H), 2.69 (qt, *J* = 7.1, 7.1 Hz, 1H), 2.07–1.98 (m, 2H), 1.65–1.52 (m, 2H), 1.41–1.22 (m, 2H), 1.24 (d, *J* = 7.0 Hz, 3H) ppm. **^{13}C{^{1}H} NMR** (126 MHz, $CDCl_3$, 298 K): δ = 147.9, 139.1, 128.4, 127.1, 125.9, 114.5, 40.0, 38.0, 34.0, 27.2, 22.4 ppm. The NMR spectroscopic data are in accordance with those reported.[S22]

3 Experimental Details for the Silylium-Ion-Initiated Silylalkynylation of Alkenes

3.1 General Procedure for the Silylium-Ion-Initiated Silylalkynylation of Alkenes (GP 3)

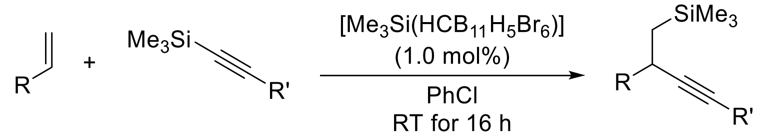

1 **2** **3**

In a glovebox, to a solution of the corresponding alkene (**1**, 0.20 mmol, 1.0 equiv) and alkynylsilane (**2**, 0.40 mmol, 2.0 equiv) in chlorobenzene (0.5 mL), silylium ion [$Me_3Si(HCB_{11}H_5Br_6)$] (1.4 mg, 2.0 µmmol, 1.0 mol%) is added and the mixture is stirred at room temperature for 16 h. The reaction mixture is then removed out from glovebox and quenched with diethyl ether (one drop). CH_2Br_2 (14.0 µL, 0.20 mmol, 1.0 equiv) is added as an internal standard to determine the yield by NMR spectroscopy. Purification by flash column chromatography on silica gel using *n*-pentane (and/or CH_2Cl_2/*n*-pentane) as eluent affords the corresponding silylalkynylation product **3** in analytically pure form.

3.2 Characterization Data of Products 3aa–ap, 3ba, and 3fa–ha

3.2.1 Trimethyl(6-phenyl-2-(phenylethynyl)hexyl)silane (**3aa**)

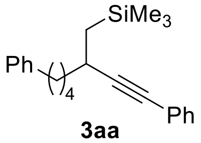

3aa

$C_{23}H_{30}Si$

M = 334.21 g/mol

Prepared from hex-5-en-1-ylbenzene (**1a**, 32.1 mg, 0.20 mmol, 1.0 equiv) and trimethyl-(phenylethynyl)silane (**2a**, 69.7 mg, 0.40 mmol, 2.0 equiv) according to **GP 3** (64% NMR yield). Purification by flash column chromatography on silica gel using *n*-pentane and then CH_2Cl_2/*n*-pentane (1:100) afforded **3aa** as a yellow oil (32.8 mg, 49% yield). **R*f*** = 0.21 (cyclohexane). **IR** (ATR): ṽ = 3060, 3025, 2930, 2855, 1693, 1599, 1491, 1452, 1247, 911, 840, 754, 694, 664 cm^{-1}. **^{1}H NMR** (500 MHz, $CDCl_3$, 298 K): δ = 7.38–7.34 (m, 2H), 7.31–7.24 (m, 5H), 7.23–7.15 (m, 3H), 2.65 (t, *J* = 7.6 Hz, 2H), 2.65–2.61 (m, 1H), 1.75–1.47 (m, 6H), 0.92 (dd, *J* = 14.4, 10.4 Hz, 1H), 0.80 (dd, *J* = 14.5, 4.7 Hz, 1H), 0.11 (s, 9H) ppm. **^{13}C{^{1}H} NMR** (126 MHz, $CDCl_3$, 298 K): δ = 142.9, 131.6, 128.6, 128.4, 128.3, 127.5, 125.7, 124.4, 95.2, 81.6, 39.2, 36.1, 31.5, 28.2, 27.2, 23.6, –0.65 ppm. **^{1}H/^{29}Si HMQC NMR** (500/99 MHz, $CDCl_3$, 298 K, optimized for *J* = 7 Hz): δ = 0.92/0.8, 0.80/0.8, 0.11/0.8 ppm. **HRMS** (APCI): calculated for $C_{23}H_{29}Si^{\bullet+}$ $[M–H]^{\bullet+}$: 333.2039; found 333.2036.

3.2.2 Trimethyl(6-phenyl-2-(*p*-tolylethynyl)hexyl)silane (**3ab**)

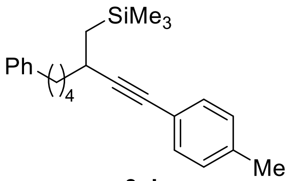

3ab
$C_{24}H_{32}Si$
M = 348.61 g/mol

Prepared from hex-5-en-1-ylbenzene (**1a**, 32.1 mg, 0.20 mmol, 1.0 equiv) and trimethyl(*p*-tolylethynyl)silane (**2b**, 75.2 mg, 0.40 mmol, 2.0 equiv) according to **GP 3** (72% NMR yield). Purification by flash column chromatography on silica gel using *n*-pentane and then CH_2Cl_2/*n*-pentane (1:100) afforded **3ab** as a yellow oil (42.5 mg, 61% yield). **R*f*** = 0.12 (cyclohexane). **IR** (ATR): ṽ = 3082, 3060, 3025, 2930, 2856, 1509, 1453, 1247, 1030, 925, 842, 816, 783, 747, 698 cm^{-1}. **^{1}H NMR** (500 MHz, $CDCl_3$, 298 K): δ = 7.32–7.26 (m, 4H), 7.24–7.17 (m, 3H), 7.12–7.08 (m, 2H), 2.67 (t, *J* = 7.5 Hz, 2H), 2.67–2.61 (m, 1H), 2.35 (s, 3H), 1.77–1.48 (m, 6H), 0.93 (dd, *J* = 14.4, 10.4 Hz, 1H), 0.81 (dd, *J* = 14.5, 4.7 Hz, 1H), 0.12 (s, 9H) ppm. **^{13}C{^{1}H} NMR** (126 MHz, $CDCl_3$, 298 K): δ = 142.9, 137.4, 131.4, 129.0, 128.6, 128.4, 125.7, 121.3, 94.4, 81.6, 39.2, 36.1, 31.5, 28.2, 27.2, 23.6, 21.5, –0.6 ppm. **^{1}H/^{29}Si HMQC NMR** (500/99 MHz, $CDCl_3$, 298 K, optimized for *J* = 7 Hz): δ = 0.93/0.6, 0.81/0.6, 0.12/0.6 ppm. **HRMS** (APCI): calculated for $C_{24}H_{31}Si^{\bullet+}$ $[M–H]^{\bullet+}$: 347.2195; found 347.2194.

3.2.3 Trimethyl(6-phenyl-2-(*m*-tolylethynyl)hexyl)silane (**3ac**)

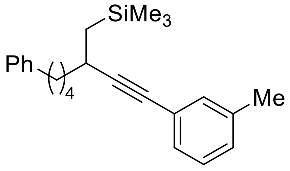

3ac
$C_{24}H_{32}Si$
M = 348.61 g/mol

Prepared from hex-5-en-1-ylbenzene (**1a**, 32.1 mg, 0.20 mmol, 1.0 equiv) and trimethyl(*m*-tolylethynyl)silane (**2c**, 75.2 mg, 0.40 mmol, 2.0 equiv) according to **GP 3** (68% NMR yield). Purification by flash column chromatography on silica gel using *n*-pentane and then CH_2Cl_2/*n*-pentane (1:100) afforded **3ac** as a yellow oil (40.4 mg, 58% yield). **R*f*** = 0.27 (cyclohexane). **IR** (ATR): ṽ = 3059, 3025, 2929, 2856, 1602, 1581, 1485, 1453, 1338, 1247, 1174, 1091, 1030, 938, 856, 838, 783, 746, 696 cm^{-1}. **^{1}H NMR** (500 MHz, $CDCl_3$, 298 K): δ = 7.32–7.27 (m, 2H), 7.24–7.17 (m, 6H), 7.12–7.07 (m, 1H), 2.67 (t, *J* = 7.4 H, 2H), 2.65–2.60 (m, 1H), 2.34 (s, 3H), 1.76–1.48 (m, 6H), 0.93 (dd, *J* = 14.5, 10.4 Hz, 1H), 0.81 (dd, *J* = 14.5, 4.7 Hz, 1H), 0.12 (s, 9H) ppm. **^{13}C{^{1}H} NMR** (126 MHz, $CDCl_3$, 298 K): δ = 142.9, 137.9, 132.1, 128.7, 128.6, 128.4, 128.4, 128.2, 125.7, 124.2, 94.8, 81.7, 39.2, 36.1, 31.5, 28.2, 27.2, 23.6, 21.4, –0.6 ppm.

^{1}H/^{29}Si HMQC NMR (500/99 MHz, $CDCl_3$, 298 K, optimized for *J* = 7 Hz): δ = 0.93/0.7, 0.81/0.7, 0.12/0.7 ppm. **HRMS** (APCI): calculated for $C_{24}H_{31}Si^{•+}$ [M–H]$^{•+}$: 347.2195; found 347.2187.

3.2.4 Trimethyl(6-phenyl-2-(*o*-tolylethynyl)hexyl)silane (**3ad**)

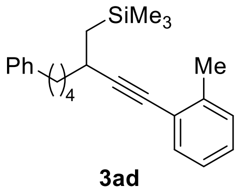

3ad
$C_{24}H_{32}Si$
M = 348.61 g/mol

Prepared from hex-5-en-1-ylbenzene (**1a**, 32.1 mg, 0.20 mmol, 1.0 equiv) and trimethyl(*o*-tolylethynyl)silane (**2d**, 75.2 mg, 0.40 mmol, 2.0 equiv) according to **GP 3** (68% NMR yield). Purification by flash column chromatography on silica gel using *n*-pentane and then CH_2Cl_2/*n*-pentane (1:100) afforded **3ad** as a yellow oil (39.7 mg, 57% yield). **R$_f$** = 0.26 (cyclohexane). **IR** (ATR): ṽ = 3061, 3024, 2929, 2855, 1601, 1485, 1453, 1337, 1246, 1195, 1157, 1115, 1043, 926, 840, 783, 754, 697, 656 cm^{-1}. **^{1}H NMR** (500 MHz, $CDCl_3$, 298 K): δ = 7.35 (d, *J* = 7.5 Hz, 1H), 7.31–7.26 (m, 2H), 7.23–7.16 (m, 5H), 7.14–7.09 (m, 1H), 2.73–2.65 (m, 1H), 2.66 (t, *J* = 7.4 Hz, 2H), 2.39 (s, 3H), 1.77–1.54 (m, 6H), 0.94 (dd, *J* = 14.5, 10.3 Hz, 1H), 0.81 (dd, *J* = 14.5, 4.8 Hz, 1H), 0.09 (s, 9H) ppm. **^{13}C{^{1}H} NMR** (126 MHz, $CDCl_3$, 298 K): δ = 142.9, 139.9, 131.9, 129.4, 128.5, 128.4, 127.5, 125.8, 125.5, 124.1, 99.2, 80.4, 39.4, 36.1, 31.5, 28.4, 27.3, 23.8, 21.0, –0.6 ppm. **^{1}H/^{29}Si HMQC NMR** (500/99 MHz, $CDCl_3$, 298 K, optimized for *J* = 7 Hz): δ = 0.96/0.6, 0.82/0.6, 0.12/0.6 ppm. **HRMS** (APCI): calculated for $C_{24}H_{33}Si^{•+}$ [M+H]$^{•+}$: 349.2352; found 349.2347.

3.2.5 (2-((3,5-Dimethylphenyl)ethynyl)-6-phenylhexyl)trimethylsilane (**3ae**)

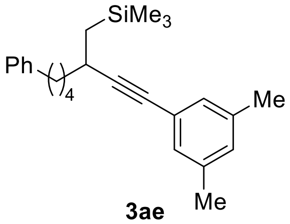

3ae
$C_{25}H_{34}Si$
M = 362.63 g/mol

Prepared from hex-5-en-1-ylbenzene (**1a**, 32.1 mg, 0.20 mmol, 1.0 equiv) and ((3,5-dimethylphenyl)ethynyl)trimethylsilane (**2e**, 81.0 mg, 0.40 mmol, 2.0 equiv) according to **GP 3** (74% NMR yield). Purification by flash column chromatography on silica gel using *n*-pentane and then CH_2Cl_2/*n*-pentane (1:100) afforded **3ae** as a yellow oil (45.7 mg, 63% yield). **R$_f$** = 0.24 (cyclohexane). **IR** (ATR): ṽ = 3059, 3026, 2929, 2856, 1719, 1690, 1598, 1495, 1453, 1308, 1247, 1032, 848, 747, 697 cm^{-1}. **^{1}H NMR** (500 MHz, $CDCl_3$, 298 K): δ = 7.31–7.26 (m, 2H), 7.23–7.16 (m, 3H), 7.01 (s, 2H), 6.91 (s, 1H), 2.66 (t, *J* = 7.6 Hz, 2H), 2.66–2.60 (m, 1H), 2.29 (s, 6H), 1.70–1.46 (m, 6H), 0.92 (dd, *J* = 14.4, 10.5 Hz, 1H), 0.80 (dd, *J* = 14.4, 4.7 Hz, 1H),

0.11 (s, 9H) ppm. **^{13}C{^{1}H} NMR** (126 MHz, $CDCl_3$, 298 K): δ = 142.9, 137.8, 129.4, 129.2, 128.6, 128.4, 125.7, 124.0, 94.4, 81.8, 39.2, 36.1, 31.5, 28.2, 27.3, 23.6, 21.3, –0.6 ppm. **^{1}H/^{29}Si HMQC NMR** (500/99 MHz, $CDCl_3$, 298 K, optimized for *J* = 7 Hz): δ = 0.92/0.6, 0.80/0.6, 0.11/0.6 ppm. **HRMS** (APCI): calculated for $C_{25}H_{33}Si^{•+}$ $[M–H]^{•+}$: 361.2352; found 361.2344.

3.2.6 Trimethyl(6-phenyl-2-((4-propylphenyl)ethynyl)hexyl)silane (**3af**)

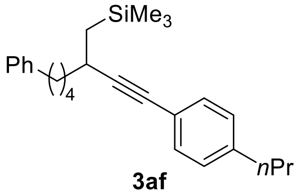

3af

$C_{26}H_{36}Si$

M = 376.66 g/mol

Prepared from hex-5-en-1-ylbenzene (**1a**, 32.1 mg, 0.20 mmol, 1.0 equiv) and trimethyl((4-propylphenyl)ethynyl)silane (**2f**, 86.6 mg, 0.40 mmol, 2.0 equiv) according to **GP 3** (80% NMR yield). Purification by flash column chromatography on silica gel using *n*-pentane and then CH_2Cl_2/*n*-pentane (1:100) afforded **3af** as a pale yellow oil (48.5 mg, 64% yield). **R_f** = 0.23 (*n*-hexane). **IR** (ATR): ṽ = 3061, 3025, 2952, 2929, 2856, 1603, 1509, 1454, 1410, 1338, 1246, 1199, 1181, 1029, 925, 839, 746, 697 cm^{-1}. **^{1}H NMR** (500 MHz, $CDCl_3$, 298 K): δ = 7.32–7.26 (m, 4H), 7.23–7.16 (m, 3H), 7.12–7.07 (m, 2H), 2.65 (t, *J* = 7.4 Hz, 2H), 2.65–2.60 (m, 1H), 2.57 (t, *J* = 7.4 Hz, 2H), 1.74–1.46 (m, 8H), 0.94 (t, *J* = 7.4 Hz, 3H), 0.91 (dd, *J* = 14.5, 10.5 Hz, 1H), 0.79 (dd, *J* = 14.5, 4.7 Hz, 1H), 0.10 (s, 9H) ppm. **^{13}C{^{1}H} NMR** (126 MHz, $CDCl_3$, 298 K): δ = 142.9, 142.2, 131.4, 128.6, 128.5, 128.4, 125.7, 121.5, 94.4, 81.6, 39.2, 38.0, 36.1, 31.5, 28.2, 27.3, 24.5, 23.6, 13.9, –0.6 ppm. **^{1}H/^{29}Si HMQC NMR** (500/99 MHz, $CDCl_3$, 298 K, optimized for *J* = 7 Hz): δ = 0.91/0.6, 0.79/0.6, 0.10/0.6 ppm. **HRMS** (APCI): calculated for $C_{26}H_{37}Si^{•+}$ $[M+H]^{•+}$: 377.2665; found 377.2655.

3.2.7 (2-((4-(*Tert*-butyl)phenyl)ethynyl)-6-phenylhexyl)trimethylsilane (**3ag**)

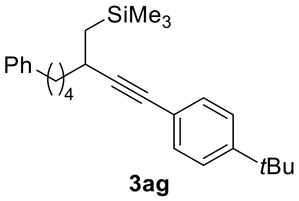

3ag

$C_{27}H_{38}Si$

M = 390.69 g/mol

Prepared from hex-5-en-1-ylbenzene (**1a**, 32.1 mg, 0.20 mmol, 1.0 equiv) and ((4-(*tert*-butyl)phenyl)ethynyl)trimethylsilane (**2g**, 92.2 mg, 0.40 mmol, 2.0 equiv) according to **GP 3** (73% NMR yield). Purification by flash column chromatography on silica gel using *n*-pentane and then CH_2Cl_2/*n*-pentane (1:100) afforded **3ag** as a pale yellow oil (49.0 mg, 63% yield). **R_f** = 0.23 (*n*-hexane). **IR** (ATR): ṽ = 3061, 3026, 2953, 2932, 2859, 1718, 1689, 1606, 1497, 1455, 1409, 1363, 1316, 1285, 1267, 1247, 1189, 1110, 1018, 925, 835, 778, 747, 698 cm^{-1}. **^{1}H NMR**

(500 MHz, $CDCl_3$, 298 K): δ = 7.31 (s, 4H), 7.30–7.26 (m, 2H), 7.23–7.16 (m, 3H), 2.65 (t, *J* = 7.6 Hz, 2H), 2.67–2.61 (m, 1H), 1.75–1.46 (m, 6H), 1.32 (s, 9H), 0.91 (dd, *J* = 14.5, 10.5 Hz, 1H), 0.79 (dd, *J* = 14.5, 4.7 Hz, 1H), 0.11 (s, 9H) ppm. **$^{13}C\{^1H\}$ NMR** (126 MHz, $CDCl_3$, 298 K): δ = 150.6, 142.9, 131.2, 128.6, 128.4, 125.7, 125.3, 121.4, 94.4, 81.6, 39.3, 36.1, 34.8, 31.5, 31.4, 28.2, 27.3, 23.6, –0.6 ppm. **$^1H/^{29}Si$ HMQC NMR** (500/99 MHz, $CDCl_3$, 298 K, optimized for *J* = 7 Hz): δ = 0.91/0.6, 0.79/0.6, 0.11/0.6 ppm. **HRMS** (APCI): calculated for $C_{27}H_{37}Si^{•+}$ [M–H]$^{•+}$: 389.2665; found 389.2666.

3.2.8 Trimethyl(6-phenyl-2-((4-(trimethylsilyl)phenyl)ethynyl)hexyl)silane (**3ah**)

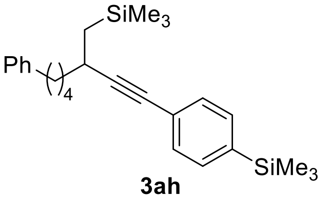

3ah

$C_{26}H_{38}Si_2$

M = 406.76 g/mol

Prepared from hex-5-en-1-ylbenzene (**1a**, 32.1 mg, 0.20 mmol, 1.0 equiv) and trimethyl(4-((trimethylsilyl)ethynyl)phenyl)silane (**2h**, 98.6 mg, 0.40 mmol, 2.0 equiv) according to **GP 3** (65% NMR yield). Purification by flash column chromatography on silica gel using *n*-pentane and then CH_2Cl_2/*n*-pentane (1:100) afforded **3ah** as a pale yellow oil (49.2 mg, 60% yield). **R_f** = 0.23 (*n*-hexane). **IR** (ATR): ṽ = 3062, 3024, 2950, 2931, 2855, 1693, 1595, 1495, 1453, 1407, 1389, 1337, 1290, 1247, 1188, 1108, 1029, 837, 755, 717, 697 cm^{-1}. **^1H NMR** (500 MHz, $CDCl_3$, 298 K): δ = 7.47–7.42 (m, 2H), 7.37–7.33 (m, 2H), 7.31–7.26 (m, 2H), 7.23–7.16 (m, 3H), 2.70–2.59 (m, 3H), 1.78–1.44 (m, 6H), 0.92 (dd, *J* = 14.4, 10.5 Hz, 1H), 0.80 (dd, *J* = 14.5, 4.7 Hz, 1H), 0.27 (s, 9H), 0.11 (s, 9H) ppm. **$^{13}C\{^1H\}$ NMR** (126 MHz, $CDCl_3$, 298 K): δ = 142.9, 139.9, 133.2, 130.7, 128.6, 128.4, 125.7, 124.7, 95.7, 81.7, 39.2, 36.1, 31.5, 28.2, 27.3, 23.6, –0.6, –1.1 ppm. **$^1H/^{29}Si$ HMQC NMR** (500/99 MHz, $CDCl_3$, 298 K, optimized for *J* = 7 Hz): δ = 0.92/0.6, 0.80/0.6, 0.11/0.6, 0.27/–4.1 ppm. **HRMS** (APCI): calculated for $C_{26}H_{37}Si_2^{•+}$ [M–H]$^{•+}$: 405.2434; found 405.2430.

3.2.9 (2-((4-Fluorophenyl)ethynyl)-6-phenylhexyl)trimethylsilane (**3ai**)

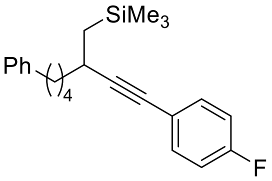

3ai

$C_{23}H_{29}FSi$

M = 352.57 g/mol

Prepared from hex-5-en-1-ylbenzene (**1a**, 32.1 mg, 0.20 mmol, 1.0 equiv) and ((4-fluorophenyl)ethynyl)trimethylsilane (**2i**, 76.9 mg, 0.40 mmol, 2.0 equiv) according to **GP 3** (57% NMR yield). Purification by flash column chromatography on silica gel using *n*-pentane and

then CH_2Cl_2/*n*-pentane (1:100) afforded **3ai** as a pale yellow oil (29.5 mg, 42% yield). **R*f*** = 0.15 (*n*-hexane). **IR** (ATR): ṽ = 3061, 3026, 2931, 2856, 1694, 1601, 1506, 1453, 1416, 1337, 1293, 1247, 1230, 1155, 1091, 1014, 836, 747, 698 cm^{-1}. **^{1}H NMR** (500 MHz, $CDCl_3$, 298 K): δ = 7.35–7.30 (m, 2H), 7.30–7.25 (m, 2H), 7.22–7.16 (m, 3H), 7.00–6.94 (m, 2H), 2.65 (t, *J* = 7.6 Hz, 2H), 2.65–2.59 (m, 1H), 1.75–1.45 (m, 6H), 0.91 (dd, *J* = 14.5, 10.4 Hz, 1H), 0.79 (dd, *J* = 14.5, 4.7 Hz, 1H), 0.10 (s, 9H) ppm. **^{13}C{^{1}H} NMR** (126 MHz, $CDCl_3$, 298 K): δ = 162.1 (d, $J_{C,F}$ = 248.3 Hz), 142.8, 133.3 (d, $J_{C,F}$ = 8.3 Hz), 128.6, 128.4, 125.8, 120.4 (d, $J_{C,F}$ = 3.3 Hz), 115.5 (d, $J_{C,F}$ = 21.9 Hz), 94.8, 80.5, 39.1, 36.1, 31.4, 28.1, 27.2, 23.5, –0.7 ppm. **^{1}H/^{29}Si HMQC NMR** (500/99 MHz, $CDCl_3$, 298 K, optimized for *J* = 7 Hz): δ = 0.91/0.6, 0.79/0.6, 0.10/0.6 ppm. **HRMS** (APCI): calculated for $C_{23}H_{28}FSi^{•+}$ [M–H]$^{•+}$: 351.1944; found 351.1939.

3.2.10 (2-((4-Chlorophenyl)ethynyl)-6-phenylhexyl)trimethylsilane (**3aj**)

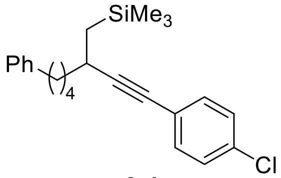

3aj
$C_{23}H_{29}ClSi$
M = 369.02 g/mol

Prepared from hex-5-en-1-ylbenzene (**1a**, 32.1 mg, 0.20 mmol, 1.0 equiv) and ((4-chlorophenyl)ethynyl)trimethylsilane (**2j**, 83.5 mg, 0.40 mmol, 2.0 equiv) according to **GP 3** (48% NMR yield). Purification by flash column chromatography on silica gel using *n*-pentane and then CH_2Cl_2/*n*-pentane (1:100) afforded **3aj** as a pale yellow oil (23.2 mg, 32% yield). **R*f*** = 0.23 (cyclohexane). **IR** (ATR): ṽ = 3060, 3026, 2932, 2856, 1489, 1454, 1399, 1338, 1247, 1091, 1029, 1014, 842, 748, 698 cm^{-1}. **^{1}H NMR** (500 MHz, $CDCl_3$, 298 K): δ = 7.31–7.22 (m, 6H), 7.21–7.16 (m, 3H), 2.65 (t, *J* = 7.6 Hz, 2H), 2.65–2.58 (m, 1H), 1.74–1.44 (m, 6H), 0.90 (dd, *J* = 14.5, 10.4 Hz, 1H), 0.79 (dd, *J* = 14.5, 4.8 Hz, 1H), 0.09 (s, 9H) ppm. **^{13}C{^{1}H} NMR** (126 MHz, $CDCl_3$, 298 K): δ = 142.8, 133.4, 132.8, 128.6, 128.6, 128.4, 125.8, 122.8, 96.3, 80.5, 39.0, 36.1, 31.4, 28.2, 27.2, 23.5, –0.7 ppm. **^{1}H/^{29}Si HMQC NMR** (500/99 MHz, $CDCl_3$, 298 K, optimized for *J* = 7 Hz): δ = 0.90/0.6, 0.79/0.6, 0.09/0.6 ppm. **HRMS** (APCI): calculated for $C_{23}H_{28}ClSi^{•+}$ [M–H]$^{•+}$: 367.1649; found 367.1646.

3.2.11 (2-((4-Bromophenyl)ethynyl)-6-phenylhexyl)trimethylsilane (**3ak**)

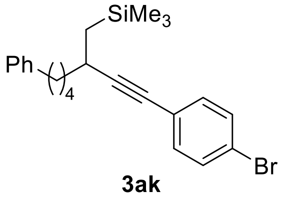

3ak
$C_{23}H_{29}BrSi$
M = 413.47 g/mol

Prepared from hex-5-en-1-ylbenzene (**1a**, 32.1 mg, 0.20 mmol, 1.0 equiv) and ((4-bromophenyl)ethynyl)trimethylsilane (**2k**, 101.3 mg, 0.40 mmol, 2.0 equiv) according to **GP 3** (40% NMR yield). Purification by flash column chromatography on silica gel using *n*-pentane and then CH_2Cl_2/*n*-pentane (1:100) afforded **3ak** as a pale yellow oil (20.0 mg, 24% yield). $\mathbf{R_f}$ = 0.26 (cyclohexane). **IR** (ATR): ṽ = 3060, 3025, 2930, 2856, 1716, 1586, 1485, 1453, 1247, 1175, 1094, 1070, 1030, 1010, 842, 747, 698 cm^{-1}. ^{1}**H NMR** (500 MHz, $CDCl_3$, 298 K): δ = 7.42–7.38 (m, 2H), 7.30–7.25 (m, 2H), 7.22–7.16 (m, 5H), 2,.64 (t, *J* = 7.6 Hz, 2H), 2.64–2.57 (m, 1H), 1.74–1.44 (m, 6H), 0.90 (dd, *J* = 14.5, 10.4 Hz, 1H), 0.78 (dd, *J* = 14.5, 4.7 Hz, 1H), 0.08 (s, 9H) ppm. ^{13}**C{**^{1}**H} NMR** (126 MHz, $CDCl_3$, 298 K): δ = 142.8, 133.0, 131.5, 128.6, 128.4, 125.8, 123.3, 121.6, 96.5, 80.6, 39.0, 36.0, 31.4, 28.2, 27.2, 23.4, –0.7 ppm. ^{1}**H/**^{29}**Si HMQC NMR** (500/99 MHz, $CDCl_3$, 298 K, optimized for *J* = 7 Hz): δ = 0.90/0.6, 0.78/0.6, 0.08/0.6 ppm. **HRMS** (APCI): calculated for $C_{23}H_{28}BrSi^{\bullet+}$ $[M–H]^{\bullet+}$: 411.1144, 413.1123; found 413.1113.

3.2.12 (2-((4-Iodophenyl)ethynyl)-6-phenylhexyl)trimethylsilane (**3al**)

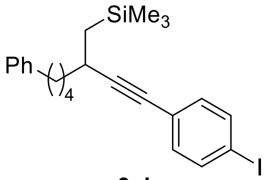

3al
$C_{23}H_{29}ISi$
M = 460.47 g/mol

Prepared from hex-5-en-1-ylbenzene (**1a**, 32.1 mg, 0.20 mmol, 1.0 equiv) and ((4-iodophenyl)ethynyl)trimethylsilane (**2l**, 120.1 mg, 0.40 mmol, 2.0 equiv) according to **GP 3** (41% NMR yield). Purification by flash column chromatography on silica gel using *n*-pentane and then CH_2Cl_2/*n*-pentane (1:100) afforded **3al** as a pale yellow oil (22.0 mg, 24% yield). $\mathbf{R_f}$ = 0.30 (cyclohexane). **IR** (ATR): ṽ = 3082, 3060, 3024, 2930, 2855, 1482, 1453, 1389, 1247, 1058, 1006, 841, 820, 746, 698 cm^{-1}. ^{1}**H NMR** (500 MHz, $CDCl_3$, 298 K): δ = 7.63–7.59 (m, 2H), 7.31–7.26 (m, 2H), 7.22–7.17 (m, 3H), 7.09–7.05 (m, 2H), 2.64 (t, *J* = 7.6 Hz, 2H), 2.64–2.57 (m, 1H), 1.74–1.45 (m, 6H), 0.90 (dd, *J* = 14.4, 10.4 Hz, 1H), 0.78 (dd, *J* = 14.4, 4.7 Hz, 1H), 0.08 (s, 9H) ppm. ^{13}**C{**^{1}**H} NMR** (126 MHz, $CDCl_3$, 298 K): δ = 142.8, 137.4, 133.2, 128.6, 128.4, 125.8, 123.9, 96.8, 93.1, 80.7, 39.0, 36.0, 31.4, 28.3, 27.2, 23.4, –0.7 ppm. ^{1}**H/**^{29}**Si HMQC NMR** (500/99 MHz, $CDCl_3$, 298 K, optimized for *J* = 7 Hz): δ = 0.90/0.6, 0.78/0.6, 0.08/0.6 ppm. **HRMS** (APCI): calculated for $C_{23}H_{30}ISi^{\bullet+}$ $[M+H]^{\bullet+}$: 461.1161; found 461.1148.

3.2.13 Trimethyl(2-(naphthalen-1-ylethynyl)-6-phenylhexyl)silane (**3am**)

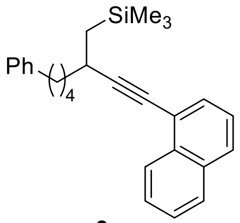

3am
$C_{27}H_{32}Si$
M = 384.64 g/mol

Prepared from hex-5-en-1-ylbenzene (**1a**, 32.1 mg, 0.20 mmol, 1.0 equiv) and trimethyl(naphthalen-1-ylethynyl)silane (**2m**, 89.8 mg, 0.40 mmol, 2.0 equiv) according to **GP 3** (48% NMR yield). Purification by flash column chromatography on silica gel using *n*-pentane and then CH_2Cl_2/*n*-pentane (1:50) afforded **3am** as a pale yellow oil (27.8 mg, 36% yield). **R*f*** = 0.13 (*n*-hexane). **IR** (ATR): ṽ = 3058, 3025, 2930, 2855, 1602, 1584, 1495, 1453, 1394, 1333, 1246, 1016, 903, 853, 798, 772, 746, 697 cm^{-1}. **^{1}H NMR** (500 MHz, $CDCl_3$, 298 K): δ = 8.34 (d, *J* = 8.2 Hz, 1H), 7.84 (d, *J* = 7.6 Hz, 1H), 7.78 (d, *J* = 8.2 Hz, 1H), 7.60 (dd, *J* = 7.1, 1.0 Hz, 1H), 7.58–7.49 (m, 2H), 7.41 (dd, *J* = 8.2, 7.2 Hz, 1H), 7.30–7.25 (m, 2H), 7.24–7.15 (m, 3H), 2.86–2.77 (m, 1H), 2.68 (t, *J* = 7.5 Hz, 2H), 1.80–1.59 (m, 6H), 1.05 (dd, *J* = 14.5, 10.3 Hz, 1H), 0.90 (dd, *J* = 14.5, 4.8 Hz, 1H), 0.14 (s, 9H) ppm. **^{13}C{^{1}H} NMR** (126 MHz, $CDCl_3$, 298 K): δ = 142.9, 133.6, 133.4, 130.0, 128.6, 128.4, 128.3, 127.9, 126.5, 126.5, 126.3, 125.8, 125.4, 122.1, 100.3, 79.6, 39.3, 36.2, 31.5, 28.6, 27.4, 23.8, –0.6 ppm. **^{1}H/^{29}Si HMQC NMR** (500/99 MHz, $CDCl_3$, 298 K, optimized for *J* = 7 Hz): δ = 1.05/0.7, 0.90/0.7, 0.14/0.7 ppm. **HRMS** (APCI): calculated for $C_{27}H_{33}Si^{\bullet+}$ $[M+H]^{\bullet+}$: 385.2352; found 385.2344.

3.2.14 Trimethyl(2-(naphthalen-2-ylethynyl)-6-phenylhexyl)silane (**3an**)

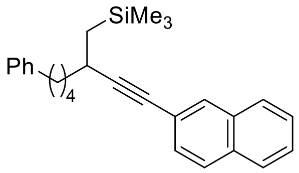

3an
$C_{27}H_{32}Si$
M = 384.64 g/mol

Prepared from hex-5-en-1-ylbenzene (**1a**, 32.1 mg, 0.20 mmol, 1.0 equiv) and trimethyl(naphthalen-2-ylethynyl)silane (**2n**, 89.8 mg, 0.40 mmol, 2.0 equiv) according to **GP 3** (68% NMR yield). Purification by flash column chromatography on silica gel using *n*-pentane and then CH_2Cl_2/*n*-pentane (1:50) afforded **3an** as a pale yellow oil (34.6 mg, 45% yield). **R*f*** = 0.20 (cyclohexane). **IR** (ATR): ṽ = 3056, 3023, 2929, 2854, 2220, 1625, 1597, 1496, 1453, 1413, 1364, 1344, 1245, 1199, 1181, 948, 925, 889, 853, 833, 816, 786, 744, 697 cm^{-1}. **^{1}H NMR** (500 MHz, $CDCl_3$, 298 K): δ = 7.88 (s, 1H), 7.83–7.79 (m, 2H), 7.76 (d, *J* = 8.6 Hz, 1H), 7.51–7.41 (m, 3H), 7.32–7.27 (m, 2H), 7.25–7.17 (m, 3H), 2.74–2.68 (m, 1H), 2.68 (t, *J* = 7.5 Hz, 2H), 1.79–1.54 (m, 6H), 0.97 (dd, *J* = 14.4, 10.4 Hz, 1H), 0.84 (dd, *J* = 4.7Hz, 1H), 0.14 (s,

9H) ppm. **^{13}C{^{1}H} NMR** (126 MHz, $CDCl_3$, 298 K): δ = 142.9, 133.2, 132.6, 130.9, 128.8, 128.6, 128.4, 127.9, 127.8, 127.7, 126.5, 126.3, 125.8, 121.7, 95.7, 81.9, 39.2, 36.1, 31.5, 28.3, 27.3, 23.6, –0.6 ppm. **^{1}H/^{29}Si HMQC NMR** (500/99 MHz, $CDCl_3$, 298 K, optimized for *J* = 7 Hz): δ = 0.97/0.7, 0.84/0.7, 0.14/0.7 ppm. **HRMS** (APCI): calculated for $C_{27}H_{33}Si^{•+}$ $[M+H]^{•+}$: 385.2352; found 385.2346.

3.2.15 Trimethyl(5-phenyl-2-(phenylethynyl)pentyl)silane (**3ba**)

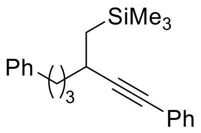

3ba
$C_{22}H_{28}Si$
M = 320.55 g/mol

Prepared from pent-4-en-1-ylbenzene (**1b**, 29.2 mg, 0.20 mmol, 1.0 equiv) and trimethyl(phenylethynyl)silane (**2a**, 69.6 mg, 0.40 mmol, 2.0 equiv) according to **GP 3** (16% NMR yield). Purification by flash column chromatography on silica gel using CH_2Cl_2/*n*-pentane (1:100) afforded **3ba** as a pale yellow oil (5.1 mg, 8% yield). **R*f*** = 0.22 (cyclohexane). **IR** (ATR): ṽ = 3083, 3058, 3026, 2943, 2857, 1600, 1491, 1453, 1339, 1247, 1029, 912, 841, 792, 754, 695, 661 cm^{-1}. **^{1}H NMR** (500 MHz, $CDCl_3$, 298 K): δ = 7.39–7.36 (m, 2H), 7.31–7.26 (m, 5H), 7.23–7.16 (m, 3H), 2.72–2.61 (m, 3H), 1.97–1.86 (m, 1H), 1.86–1.74 (m, 1H), 1.66–1.54 (m, 2H), 0.91 (dd, *J* = 14.5, 10.5 Hz, 1H), 0.79 (dd, *J* = 14.5, 4.7 Hz, 1H), 0.09 (s, 9H) ppm. **^{13}C{^{1}H} NMR** (126 MHz, $CDCl_3$, 298 K): δ = 142.7, 131.6, 128.6, 128.4, 128.3, 127.5, 125.8, 124.4, 95.1, 81.7, 38.8, 35.9, 29.3, 28.1, 23.5, –0.7 ppm. **^{1}H/^{29}Si HMQC NMR** (500/99 MHz, $CDCl_3$, 298 K, optimized for *J* = 7 Hz): δ = 0.91/0.6, 0.79/0.6, 0.09/0.6 ppm. **HRMS** (APCI): calculated for $C_{22}H_{27}Si^{•+}$ $[M–H]^{•+}$: 319.1882; found 319.1879.

3.2.16 Trimethyl(6-phenyl-2-(phenylethynyl)heptyl)silane (**3fa**)

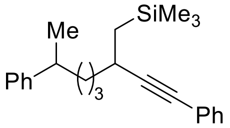

3fa
$C_{24}H_{32}Si$
M = 348.61 g/mol

Prepared from hept-6-en-2-ylbenzene (**1f**, 34.9 mg, 0.20 mmol, 1.0 equiv) and trimethyl(phenylethynyl)silane (**2a**, 69.7 mg, 0.40 mmol, 2.0 equiv) according to **GP 3** (65% NMR yield). Purification by flash column chromatography on silica gel using *n*-pentane and then CH_2Cl_2/*n*-pentane (1:100) afforded **3fa** as a pale yellow oil (35.4 mg, 51% yield). **R*f*** = 0.23 (cyclohexane). **IR** (ATR): ṽ = 3080, 3060, 3026, 2953, 2927, 2870, 1599, 1491, 1452, 1247, 909, 841, 756, 698 cm^{-1}. **^{1}H NMR** (500 MHz, $CDCl_3$, 298 K): δ = 7.34–7.25 (m, 7H), 7.23–7.15 (m, 3H), 2.72 (qt, *J* = 7.1, 7.0 Hz, 1H), 2.64–2.55 (m, 1H), 1.71–1.41 (m, 6H), 1.26, 1.26 (d, *J* = 7.0 Hz, 3H), 0.92–0.83 (m, 1H), 0.79–0.72 (m, 1H), 0.08, 0.08 (s, 9H) ppm. **^{13}C{^{1}H} NMR**

(126 MHz, $CDCl_3$, 298 K): δ = 148.0, 147.9, 131.6, 131.5, 128.4, 128.3, 127.5, 127.2, 127.1, 125.9, 124.4, 124.4, 95.2, 95.2, 81.6, 81.5, 40.1, 40.0, 39.4, 39.3, 38.3, 28.2, 28.1, 25.7, 25.6, 23.6, 23.5, 22.6, 22.4 –0.7 ppm. **$^{1}H/^{29}Si$ HMQC NMR** (500/99 MHz, $CDCl_3$, 298 K, optimized for *J* = 7 Hz): δ = 0.87/0.6, 0.75/0.6, 0.08/0.6 ppm. **HRMS** (APCI): calculated for $C_{24}H_{33}Si^{•+}$ $[M+H]^{•+}$: 349.2352; found 349.2344.

3.2.17 Trimethyl(2-(phenylethynyl)octyl)silane (**3ga**)

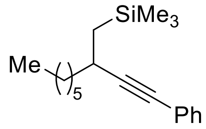

3ga
$C_{19}H_{30}Si$
M = 286.21 g/mol

Prepared from oct-1-ene (**1g**, 22.5 mg, 0.20 mmol, 1.0 equiv) and trimethyl(phenylethynyl)silane (**2a**, 69.7 mg, 0.40 mmol, 2.0 equiv) according to **GP 3** (82% NMR yield). Purification by flash column chromatography on silica gel using *n*-pentane afforded **3ga** as a colorless oil (37.2 mg, 65% yield). **R_f** = 0.62 (cyclohexane). **IR** (ATR): ṽ = 3057, 3031, 2953, 2926, 2855, 1490, 1459, 1247, 910, 841, 754, 690, 663 cm^{-1}. **^{1}H NMR** (500 MHz, $CDCl_3$, 298 K): δ = 7.40–7.36 (m, 2H), 7.30–7.22 (m, 3H), 2.68–2.58 (m, 1H), 1.61–1.49 (m, 3H), 1.49–1.40 (m, 1H), 1.39–1.26 (m, 6H), 0.95–0.87 (m, 4H), 0.81 (dd, *J* = 14.5, 4.8 Hz, 1H), 0.10 (s, 9H) ppm. **$^{13}C\{^{1}H\}$ NMR** (126 MHz, $CDCl_3$, 298 K): δ = 131.6, 128.3, 127.5, 124.5, 95.5, 81.4, 39.3, 32.0, 29.3, 28.2, 27.5, 23.5, 22.8, 14.2, –0.6 ppm. **$^{1}H/^{29}Si$ HMQC NMR** (500/99 MHz, $CDCl_3$, 298 K, optimized for *J* = 7 Hz): δ = 0.91/0.6, 0.81/0.6, 0.10/0.6 ppm. **HRMS** (APCI): calculated for $C_{19}H_{29}Si^{•+}$ $[M–H]^{•+}$: 285.2039; found 285.2038.

3.2.18 Trimethyl(2-(phenylethynyl)hexyl)silane (**3ha**)

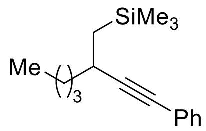

3ha
$C_{17}H_{26}Si$
M = 258.48 g/mol

Prepared from hex-1-ene (**1h**, 16.8 mg, 0.20 mmol, 1.0 equiv) and trimethyl(phenylethynyl)silane (**2a**, 69.7 mg, 0.40 mmol, 2.0 equiv) according to **GP 3** (80% NMR yield). Purification by flash column chromatography on silica gel using *n*-pentane afforded **3ha** as a colorless oil (34.4 mg, 66% yield). **R_f** = 0.50 (cyclohexane). **IR** (ATR): ṽ = 3057, 3030, 2953, 2927, 2858, 1598, 1489, 1458, 1442, 1338, 1247, 1193, 1069, 1028, 946, 909, 838, 754, 690, 660 cm^{-1}. **^{1}H NMR** (500 MHz, $CDCl_3$, 298 K): δ = 7.41–7.37 (m, 2H), 7.31–7.23 (m, 3H), 2.68–2.60 (m, 1H), 1.62–1.50 (m, 3H), 1.50–1.30 (m, 3H), 0.94 (t, *J* = 7.3 Hz, 3H), 0.92 (dd, *J* = 14.4, 10.4 Hz, 1H), 0.81 (dd, *J* = 14.4, 4.7 Hz, 1H), 0.11 (s, 9H) ppm. **$^{13}C\{^{1}H\}$ NMR** (126 MHz, $CDCl_3$, 298 K): δ = 131.6, 128.3, 127.5, 124.5, 95.4, 81.4, 39.0, 29.8, 28.2,

23.5, 22.7, 14.3, –0.6 ppm. **^{1}H/^{29}Si HMQC NMR** (500/99 MHz, $CDCl_3$, 298 K, optimized for *J* = 7 Hz): δ = 0.92/0.5, 0.81/0.5 ppm. **HRMS** (APCI): calculated for $C_{17}H_{27}Si^{•+}$ $[M+H]^{•+}$: 259.1882; found 259.1876.

3.2.19 Triethyl(6-phenyl-2-(phenylethynyl)hexyl)silane (**3ao**)

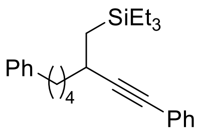

3ao
$C_{26}H_{36}Si$
M = 376.66 g/mol

Prepared from hex-5-en-1-ylbenzene (**1a**, 32.1 mg, 0.20 mmol, 1.0 equiv) and triethyl(phenylethynyl)silane (**2o**, 86.6 mg, 0.40 mmol, 2.0 equiv) according to **GP 3** using $[Et_3Si(HCB_{11}H_5Br_6)]$ (1.5 mg, 2.0 µmmol, 1.0 mol%) as initiator (50% NMR yield). Purification by flash column chromatography on silica gel using *n*-pentane and then CH_2Cl_2/*n*-pentane (1:100) afforded **3ao** as a pale yellow oil (24.0 mg, 32% yield). **R*f*** = 0.24 (cyclohexane). **IR** (ATR): ṽ = 3060, 3025, 2931, 2909, 2872, 1599, 1491, 1454, 1237, 1014, 786, 753, 727, 694 cm^{-1}. **^{1}H NMR** (500 MHz, $CDCl_3$, 298 K): δ = 7.38–7.34 (m, 2H), 7.31–7.25 (m, 5H), 7.22–7.15 (m, 3H), 2.65 (t, *J* = 7.4 Hz, 2H), 2.65–2.58 (m, 1H), 1.73–1.47 (m, 6H), 0.97 (t, *J* = 8.1 Hz, 9H), 0.94 (dd, *J* = 14.6, 10.7 Hz, 1H), 0.78 (dd, *J* = 14.6, 4.4 Hz, 1H), 0.69–0.58 (m, 6H) ppm. **^{13}C{^{1}H} NMR** (126 MHz, $CDCl_3$, 298 K): δ = 142.9, 131.5, 128.6, 128.4, 128.3, 127.5, 125.7, 124.4, 95.3, 81.3, 39.5, 36.1, 31.5, 28.0, 27.3, 18.3, 7.7, 3.9 ppm. **^{1}H/^{29}Si HMQC NMR** (500/99 MHz, $CDCl_3$, 298 K, optimized for *J* = 7 Hz): δ = 0.97/6.1, 0.94/6.1, 0.78/6.1, 0.64/6.1 ppm. **HRMS** (APCI): calculated for $C_{26}H_{37}Si^{•+}$ $[M+H]^{•+}$: 377.2665; found 377.2659.

3.2.20 Dimethyl(phenyl)(6-phenyl-2-(phenylethynyl)hexyl)silane (**3ap**)

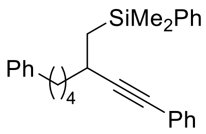

3ap
$C_{28}H_{32}Si$
M = 396.65 g/mol

Prepared from hex-5-en-1-ylbenzene (**1a**, 32.1 mg, 0.20 mmol, 1.0 equiv) and dimethyl(phenyl)(phenylethynyl)silane (**2a**, 94.6 mg, 0.40 mmol, 2.0 equiv) according to **GP 3** using $[Me_3Si(HCB_{11}H_5Br_6)]$ (1.4 mg, 2.0 µmmol, 1.0 mol%) (52% NMR yield). Purification by flash column chromatography on silica gel using *n*-pentane and then CH_2Cl_2/*n*-pentane (1:100) afforded **3ap** as a pale yellow oil (24.5 mg, 31% yield). **R*f*** = 0.08 (cyclohexane). **IR** (ATR): ṽ = 3064, 3023, 2930, 2855, 1598, 1519, 1488, 1442, 1427, 1337, 1248, 1112, 1069, 1028, 965, 912, 835, 805, 755, 729, 697 cm^{-1}. **^{1}H NMR** (500 MHz, $CDCl_3$, 298 K): δ = 7.58–7.54 (m, 2H), 7.37–7.33 (m, 3H), 7.33–7.24 (m, 7H), 7.20–7.15 (m, 3H), 2.67–2.61 (m, 1H), 2.61 (t, *J* = 7.4 Hz, 2H), 1.70–1.53 (m, 5H), 1.52–1.44 (m, 1H), 1.16 (dd, *J* = 14.6, 10.4 Hz, 1H), 1.04 (dd, *J* =

14.6, 4.6 Hz, 1H), 0.41 (s, 3H), 0.40 (s, 3H) ppm. **$^{13}C\{^1H\}$ NMR** (126 MHz, $CDCl_3$, 298 K): δ = 142.9, 139.6, 133.8, 131.6, 129.0, 128.6, 128.4, 128.3, 127.9, 127.5, 125.7, 124.3, 95.0, 81.9, 39.1, 36.0, 31.4, 28.1, 27.1, 22.8, –1.7, –2.3 ppm. **$^1H/^{29}Si$ HMQC NMR** (500/99 MHz, $CDCl_3$, 298 K, optimized for *J* = 7 Hz): δ = 1.16/–3.9, 1.04/–3.9, 0.41/–3.9, 0.40/–3.9 ppm. **HRMS** (APCI): calculated for $C_{28}H_{33}Si^{•+}$ [M+H]$^{•+}$: 397.2352; found 397.2341.

3.3 Characterization Data of Products 4ba, 5ca, and 6ea

3.3.1 Trimethyl((1,2,3,4-tetrahydronaphthalen-1-yl)methyl)silane (**4ba**)

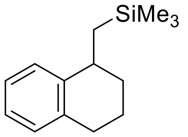

4ba
$C_{14}H_{22}Si$
M = 218.42 g/mol

Prepared from pent-4-en-1-ylbenzene (**1b**, 29.2 mg, 0.20 mmol, 1.0 equiv) and trimethyl(phenylethynyl)silane (**2a**, 69.6 mg, 0.40 mmol, 2.0 equiv) according to **GP 3** (38% NMR yield). Purification by flash column chromatography on silica gel using *n*-pentane afforded **4ba** as a colorless oil (9.1 mg, 21% yield). **R_f** = 0.58 (cyclohexane). **IR** (ATR): ṽ = 3064, 3019, 2947, 2900, 1487, 1451, 1415, 1248, 1211, 909, 861, 838, 753, 690 cm^{-1}. **^1H NMR** (500 MHz, $CDCl_3$, 298 K): δ = 7.16 (d, *J* = 7.5 Hz, 1H), 7.14–7.09 (m, 1H), 7.09–7.02 (m, 2H), 3.00–2.92 (m, 1H), 2.83–2.68 (m, 2H), 1.97–1.83 (m, 2H), 1.75–1.66 (m, 1H), 1.64–1.57 (m, 1H), 1.04 (dd, *J* = 15.0, 3.6 Hz, 1H), 0.94 (dd, *J* = 15.0, 10.8 Hz, 1H), 0.07 (s, 9H) ppm. **$^{13}C\{^1H\}$ NMR** (126 MHz, $CDCl_3$, 298 K): δ = 144.2, 136.6, 129.1, 128.5, 125.7, 125.3, 34.3, 30.7, 29.9, 26.1, 20.1, –0.4 ppm. **$^1H/^{29}Si$ HMQC NMR** (500/99 MHz, $CDCl_3$, 298 K, optimized for *J* = 7 Hz): δ = 1.04/0.3, 0.94/0.3, 0.07/0.3 ppm. **HRMS** (APCI): calculated for $C_{14}H_{21}Si^{•+}$ [M–H]$^{•+}$: 217.1413; found 217.1410.

3.3.2 Trimethyl(1,2,3,4-tetrahydronaphthalen-2-yl)silane (**5ca**)

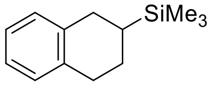

5ca
$C_{13}H_{20}Si$
M = 204.39 g/mol

Prepared from but-3-en-1-ylbenzene (**1c**, 26.4 mg, 0.20 mmol, 1.0 equiv) and trimethyl(phenylethynyl)silane (**2a**, 69.6 mg, 0.40 mmol, 2.0 equiv) according to **GP 3** (36% NMR yield). Purification by flash column chromatography on silica gel using *n*-pentane afforded **5ca** as a colorless oil (8.5 mg, 21% yield). **R_f** = 0.50 (cyclohexane). **IR** (ATR): ṽ = 3078, 3059, 3016, 2952, 2914, 2873, 2851, 2833, 1493, 1450, 1433, 1248, 1040, 921, 885, 833, 786, 742, 688 cm^{-1}. **^1H NMR** (500 MHz, $CDCl_3$, 298 K): δ = 7.15–7.04 (m, 4H), 2.88–2.72 (m, 3H), 2.70–2.59 (m, 1H), 2.02–1.93 (m, 1H), 1.56–1.44 (m, 1H), 0.97 (tdd, *J* = 12.7, 4.9, 2.6

Hz, 1H), 0.05 (s, 9H) ppm. **^{13}C{^{1}H} NMR** (126 MHz, $CDCl_3$, 298 K): δ = 137.9, 137.4, 129.3, 128.9, 125.5, 125.5, 30.5, 30.4, 24.2, 22.3, –3.4 ppm. **^{1}H/^{29}Si HMQC NMR** (500/99 MHz, $CDCl_3$, 298 K, optimized for *J* = 7 Hz): δ = 2.65/3.0, 1.50/3.0, 0.05/3.0 ppm. **HRMS** (APCI): calculated for $C_{13}H_{19}Si^{•+}$ [M–H]$^{•+}$: 203.1256; found 203.1253.

3.3.3 Trimethyl((4-phenylspiro[4.5]dec-3-en-2-yl)methyl)silane (**6ea**)

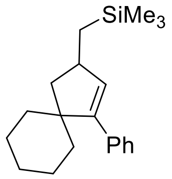

6ea
$C_{20}H_{30}Si$
M = 298.55 g/mol

Prepared from allylcyclohexane (**1e**, 24.8 mg, 0.20 mmol, 1.0 equiv) and trimethyl(phenylethynyl)silane (**2a**, 69.6 mg, 0.40 mmol, 2.0 equiv) according to **GP 3** (28% NMR yield). Purification by flash column chromatography on silica gel using *n*-pentane afforded **6ea** as a colorless oil (8.5 mg, 14% yield). **R*f*** = 0.57 (cyclohexane). **IR** (ATR): ṽ = 3053, 3025, 2927, 2855, 1509, 1493, 1247, 1029, 838, 805, 761, 698 cm^{-1}. **^{1}H NMR** (500 MHz, $CDCl_3$, 298 K): δ = 7.31–7.27 (m, 2H), 7.26–7.21 (m, 3H), 5.52 (d, *J* = 1.9 Hz, 1H), 2.85–2.76 (m, 1H), 2.37 (dd, *J* = 12.6, 7.6 Hz, 1H), 1.66–1.31 (m, 10 H), 1.28 (dd, *J* = 12.5, 7.5 Hz, 1H), 0.81 (dd, *J* = 14.5, 6.9 Hz, 1H), 0.67 (dd, *J* = 14.5, 8.2 Hz, 1H), 0.05 (s, 9H) ppm. **^{13}C{^{1}H} NMR** (126 MHz, $CDCl_3$, 298 K): δ = 151.5, 138.7, 135.0, 128.8, 127.8, 126.6, 52.0, 45.5, 38.6, 37.6, 34.1, 26.0, 25.0, 23.9, 23.2, –0.5 ppm. **^{1}H/^{29}Si HMQC NMR** (500/99 MHz, $CDCl_3$, 298 K, optimized for *J* = 7 Hz): δ = 0.81/0.2, 0.67/0.2, 0.05/0.2 ppm. **HRMS** (APCI): calculated for $C_{20}H_{29}Si^{•+}$ [M–H]$^{•+}$: 297.2039; found 297.2031.

4 NMR Spectra

Figure S1. **^{1}H NMR** spectrum (500 MHz, $CDCl_3$, 298 K) of **3aa** from the reaction of alkene (**1a**) and alkynylsilane (**2a**).

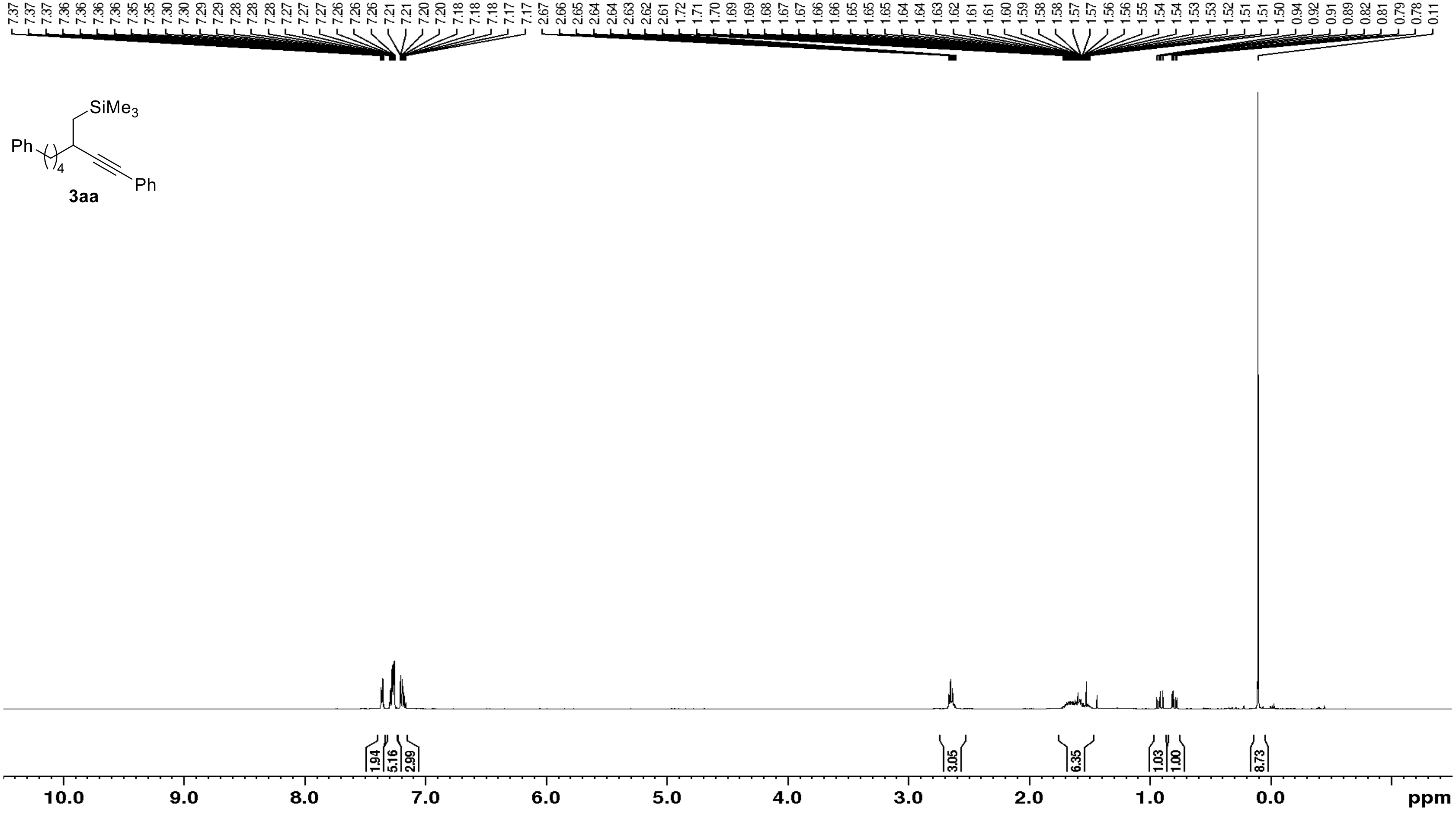

Figure S2. $^{13}C\{^{1}H\}$ **NMR** spectrum (126 MHz, $CDCl_3$, 298 K) of **3aa** from the reaction of alkene (**1a**) and alkynylsilane (**2a**).

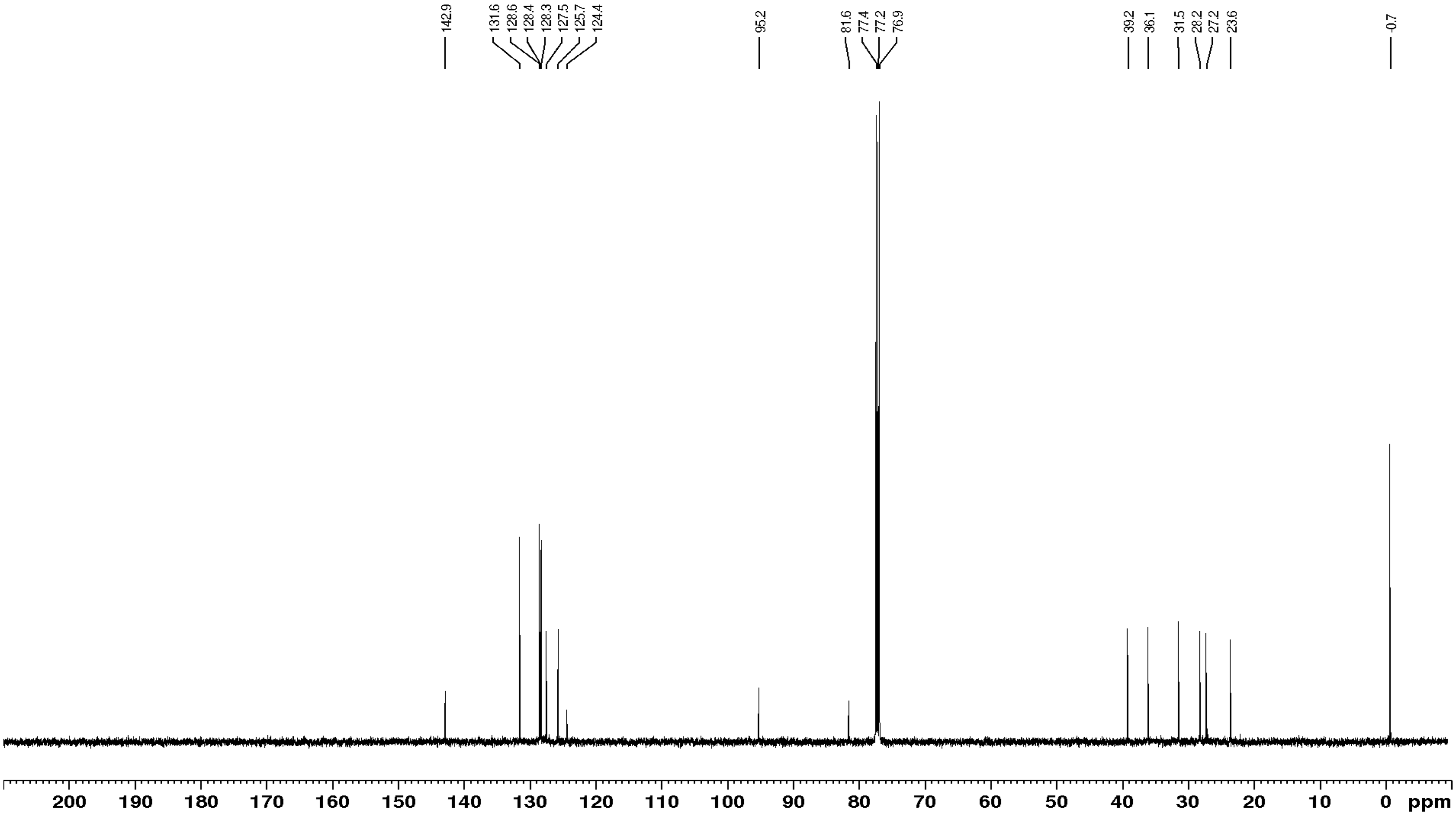

Figure S3. **$^{1}H/^{29}Si$ HMQC** NMR spectrum (500/99 MHz, $CDCl_3$, 298 K, optimized for *J* = 7 Hz) of **3aa** from the reaction of alkene (**1a**) and alkynylsilane (**2a**).

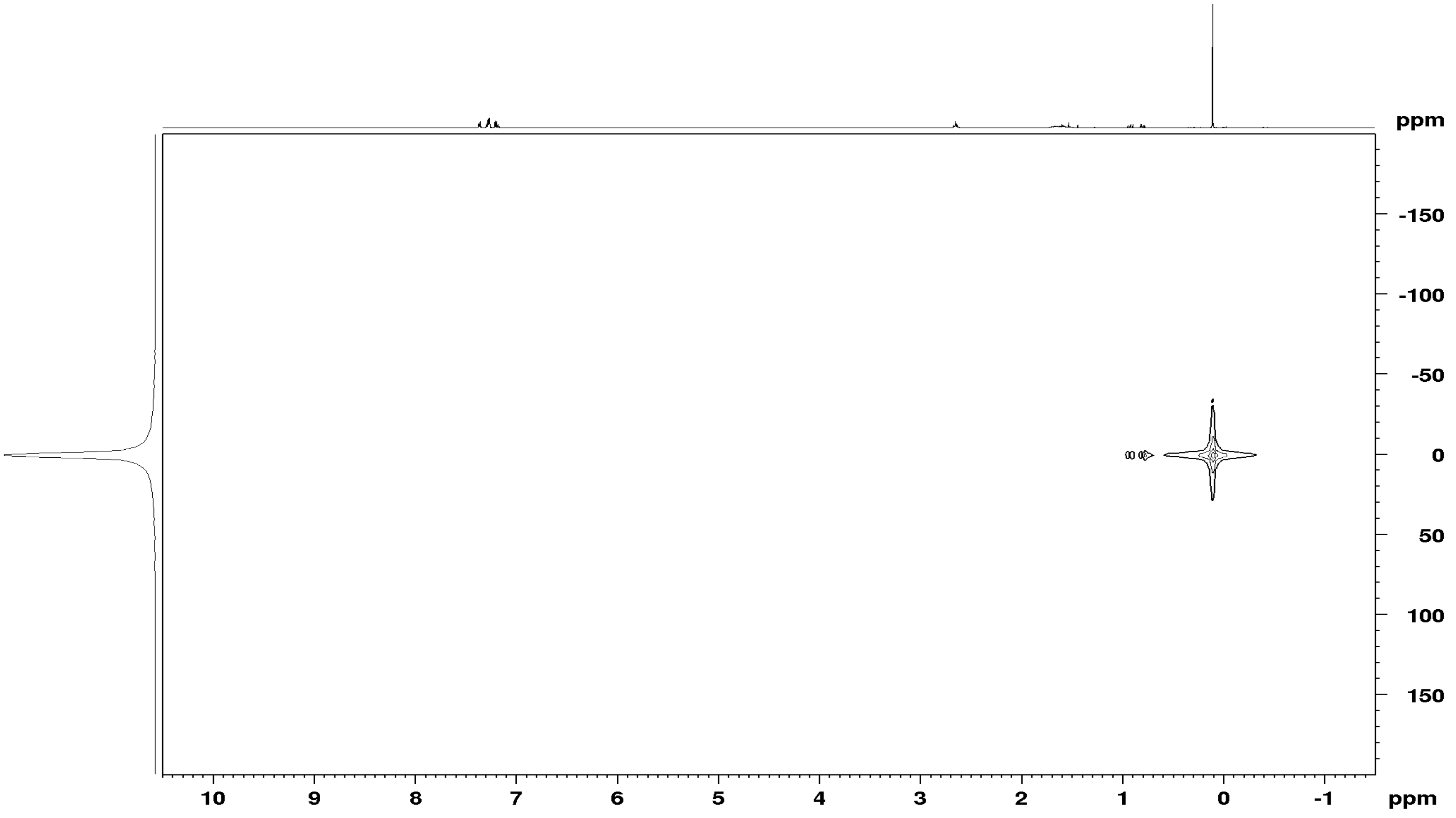

Figure S4. **^{1}H NMR** spectrum (500 MHz, $CDCl_3$, 298 K) of **3ab** from the reaction of alkene (**1a**) and alkynylsilane (**2b**).

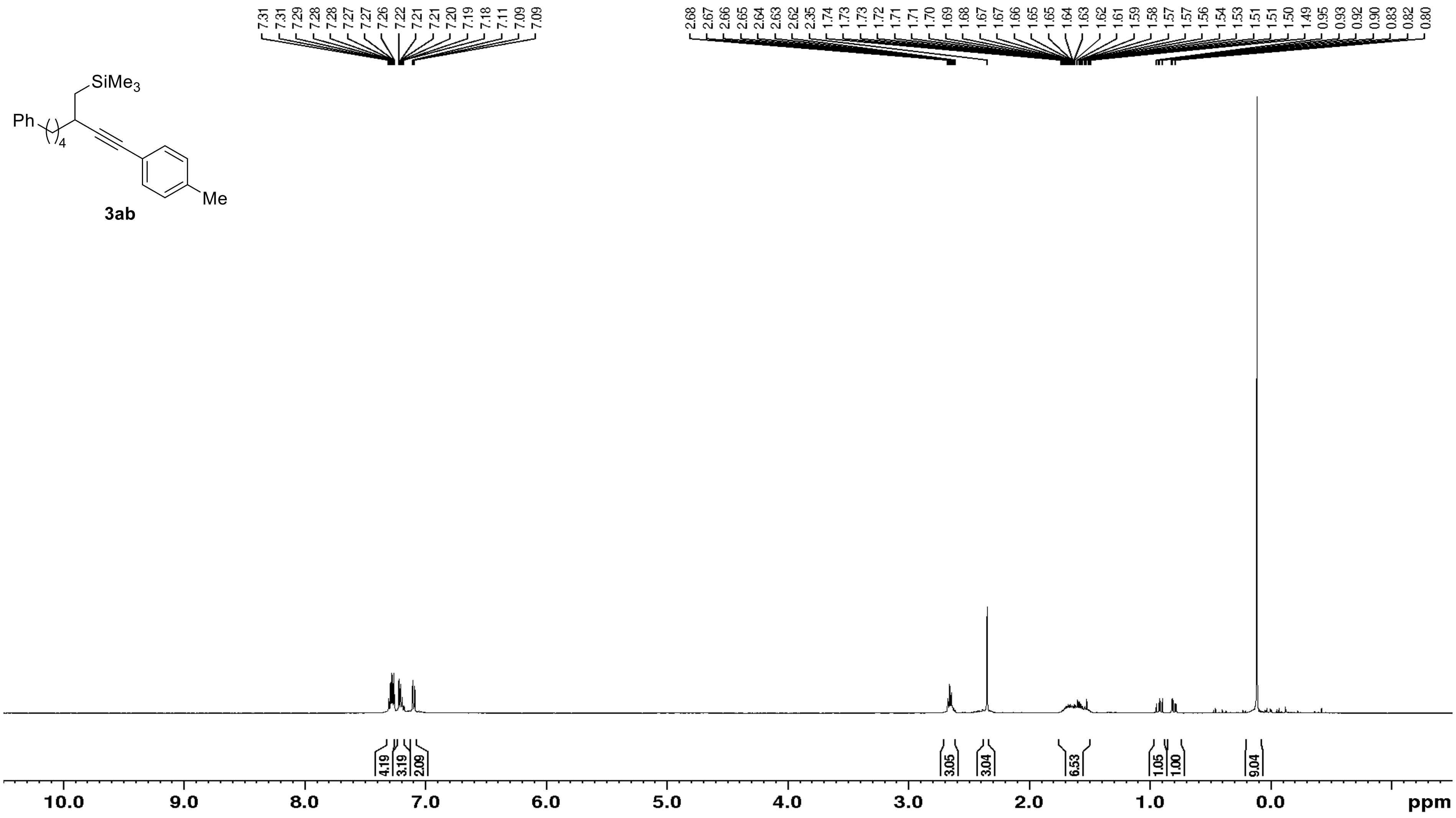

Figure S5. **$^{13}C\{^1H\}$ NMR** spectrum (126 MHz, $CDCl_3$, 298 K) of **3ab** from the reaction of alkene (**1a**) and alkynylsilane (**2b**).

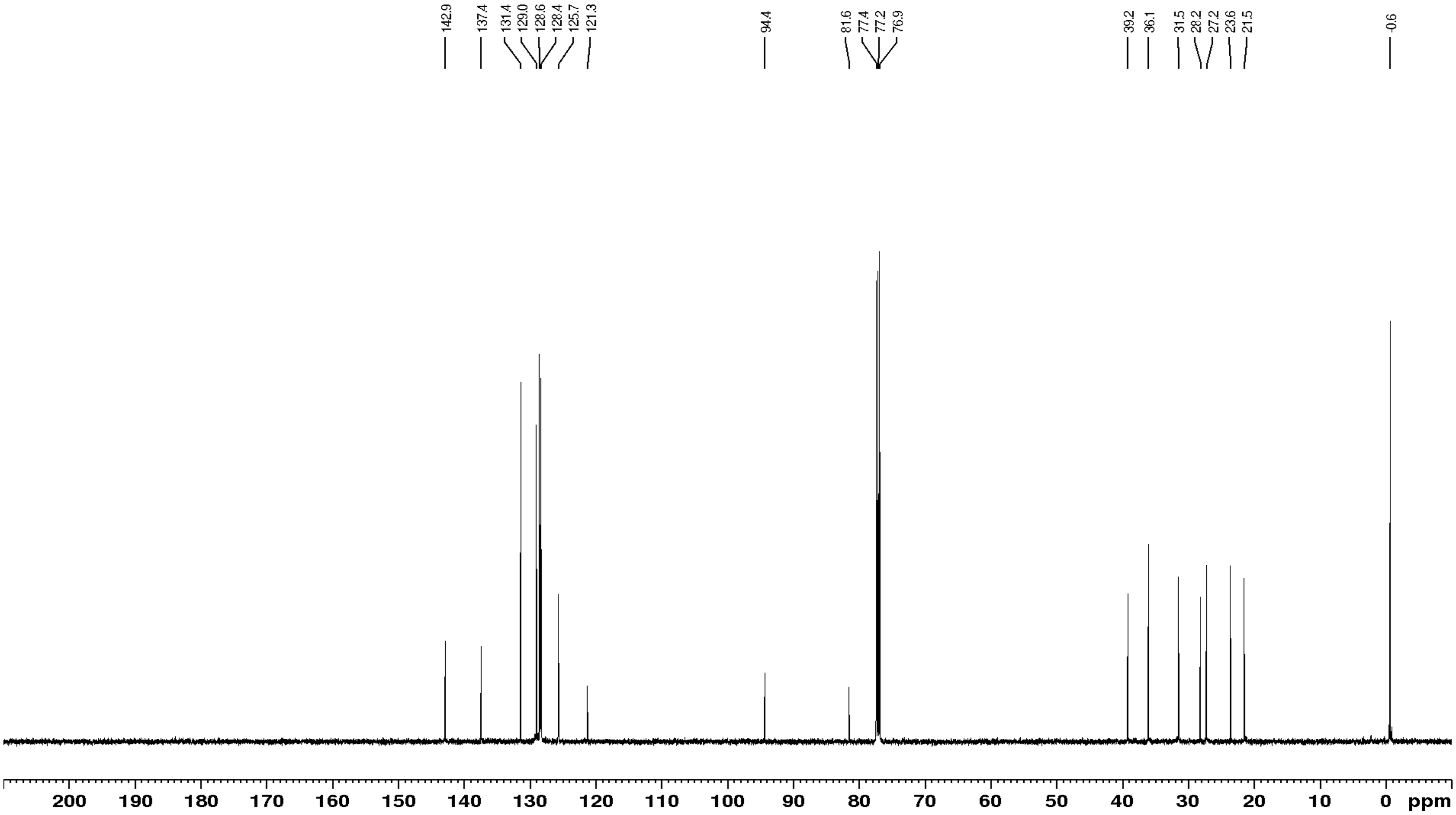

Figure S6. **^{1}H/^{29}Si HMQC** NMR spectrum (500/99 MHz, $CDCl_3$, 298 K, optimized for J = 7 Hz) of **3ab** from the reaction of alkene (**1a**) and alkynylsilane (**2b**).

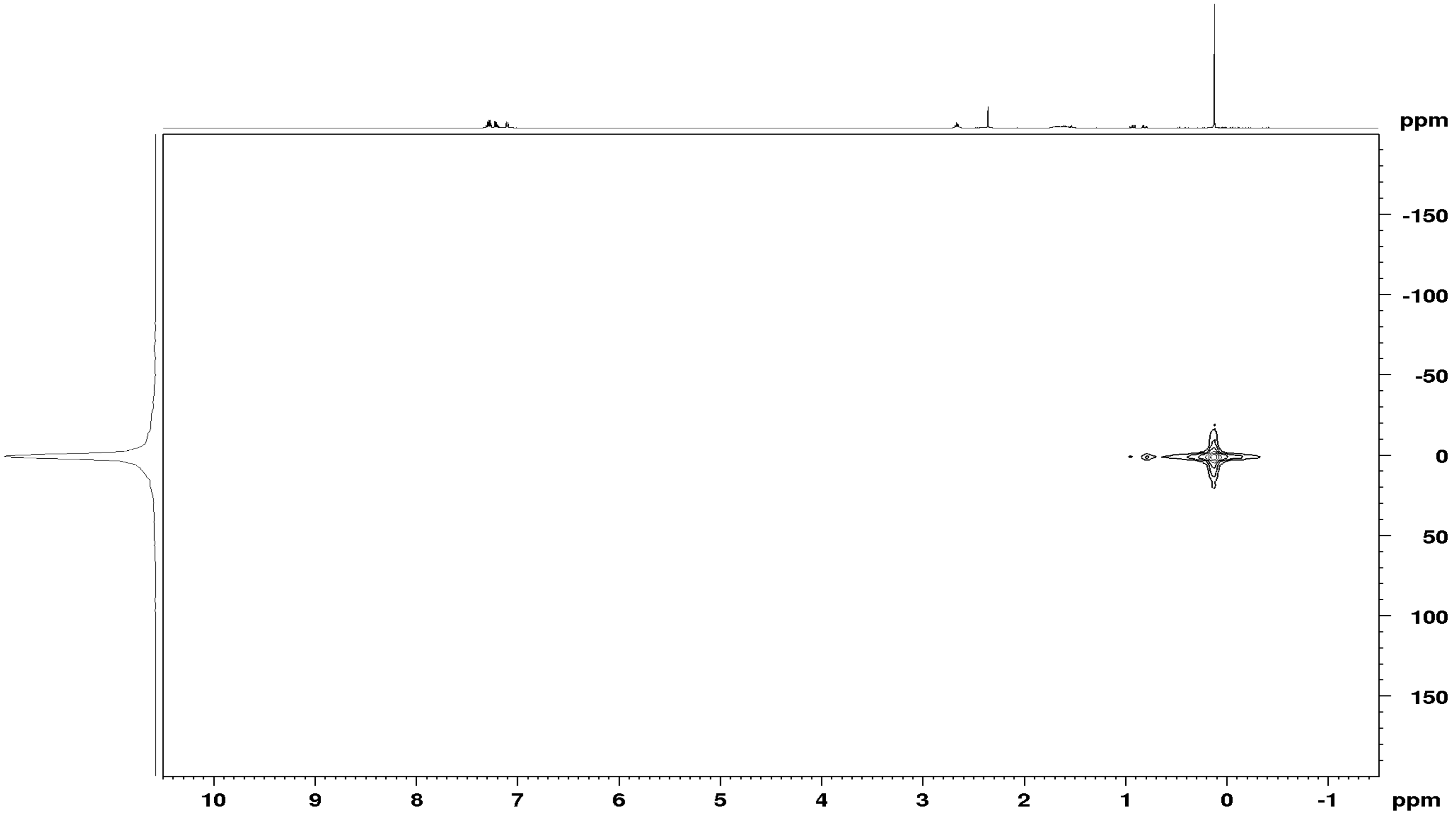

Figure S7. **^{1}H NMR** spectrum (500 MHz, $CDCl_3$, 298 K) of **3ac** from the reaction of alkene (**1a**) and alkynylsilane (**2c**).

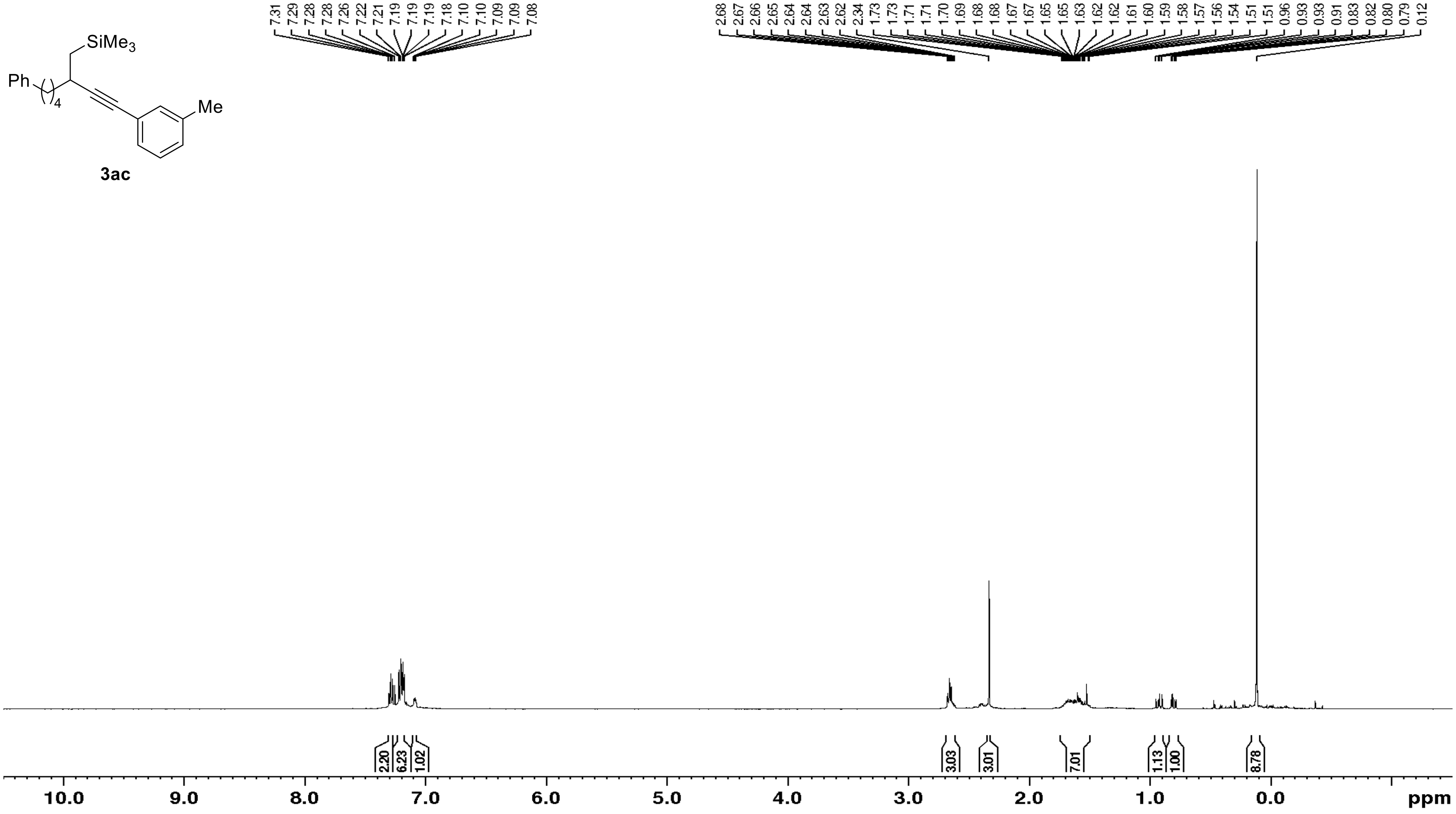

Figure S8. **$^{13}C\{^1H\}$ NMR** spectrum (126 MHz, $CDCl_3$, 298 K) of **3ac** from the reaction of alkene (**1a**) and alkynylsilane (**2c**).

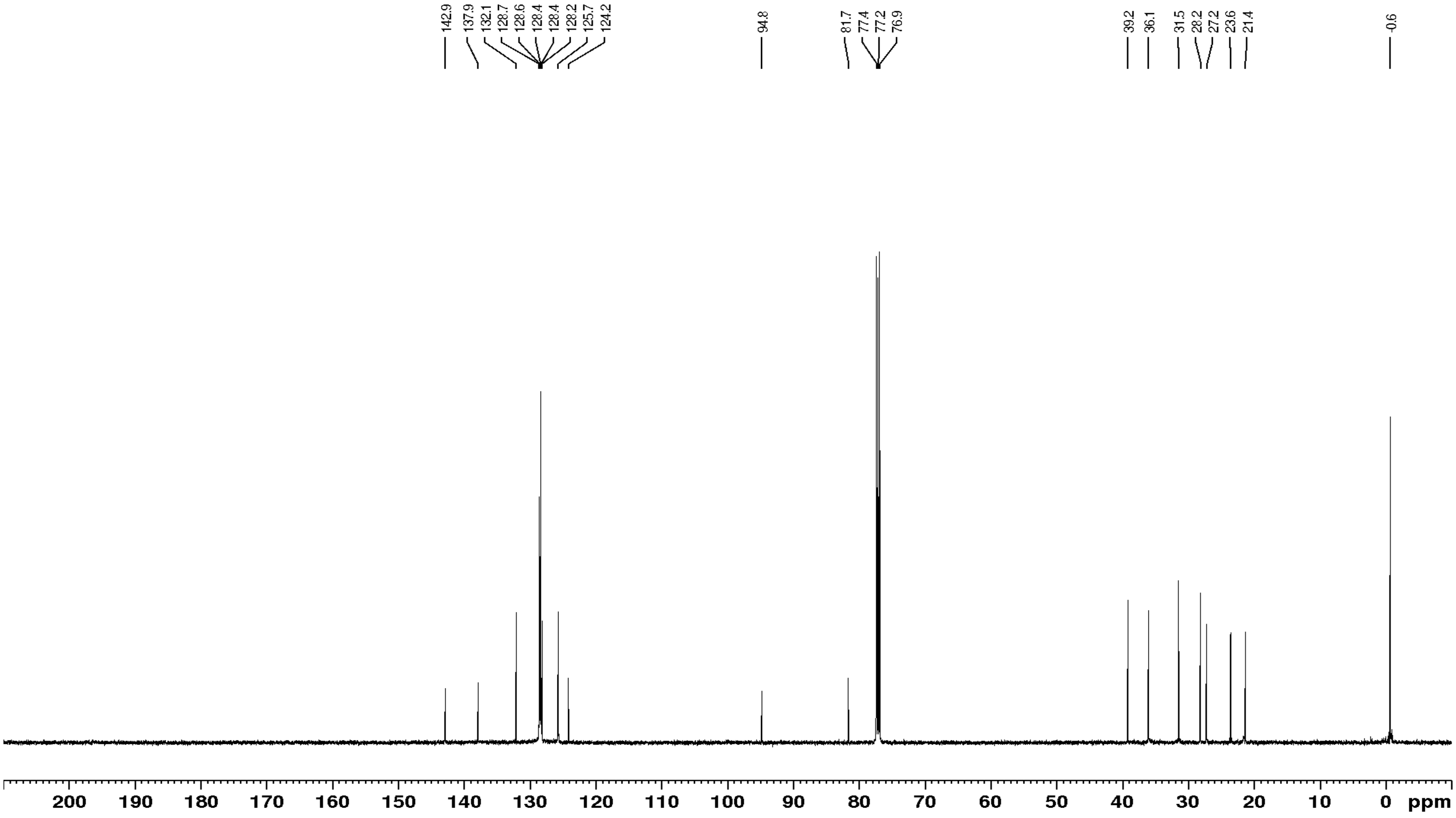

Figure S9. **$^{1}H/^{29}Si$ HMQC** NMR spectrum (500/99 MHz, $CDCl_3$, 298 K, optimized for J = 7 Hz) of **3ac** from the reaction of alkene (**1a**) and alkynylsilane (**2c**).

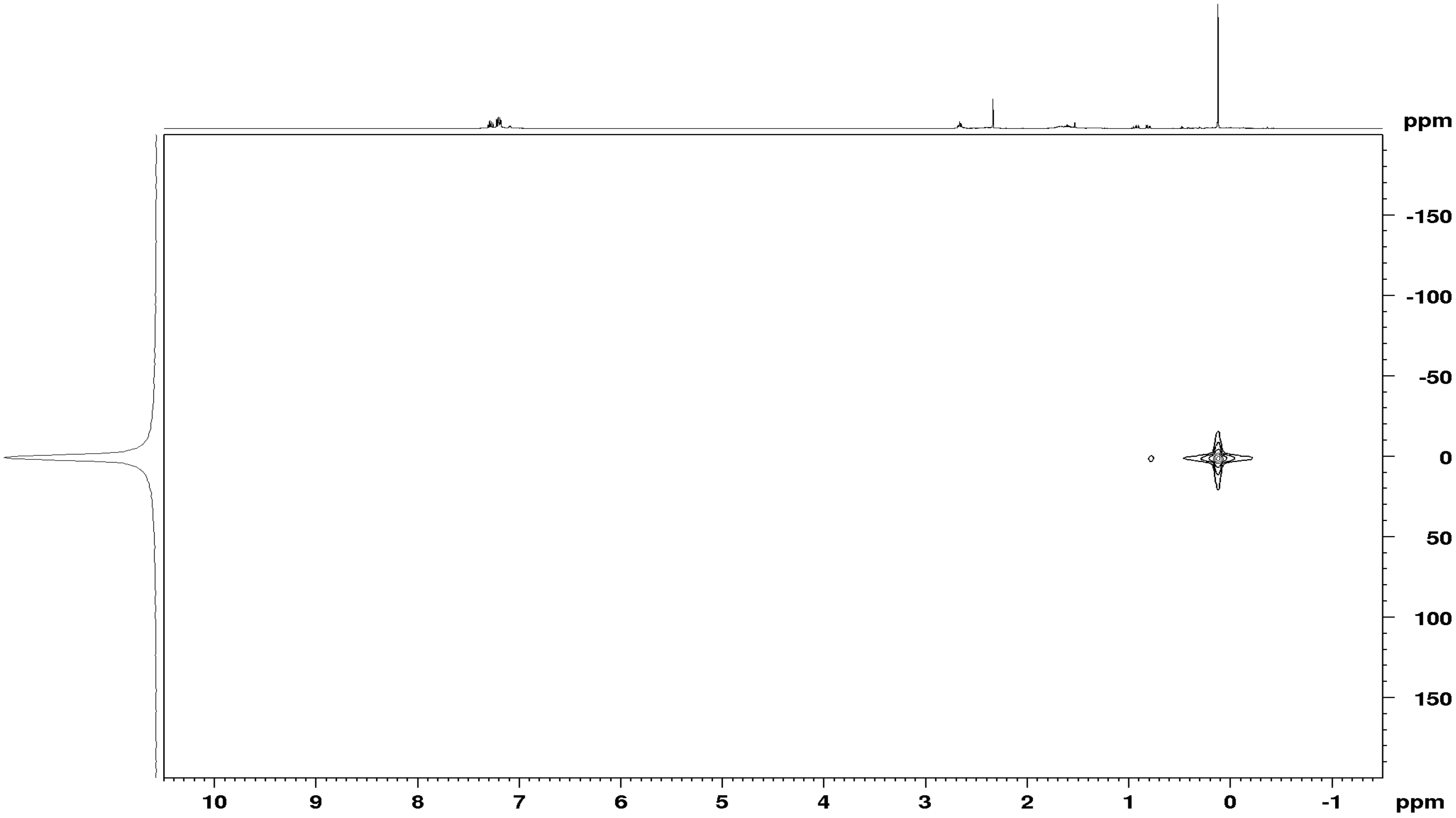

Figure S10. **^{1}H NMR** spectrum (500 MHz, $CDCl_3$, 298 K) of **3ad** from the reaction of alkene (**1a**) and alkynylsilane (**2d**).

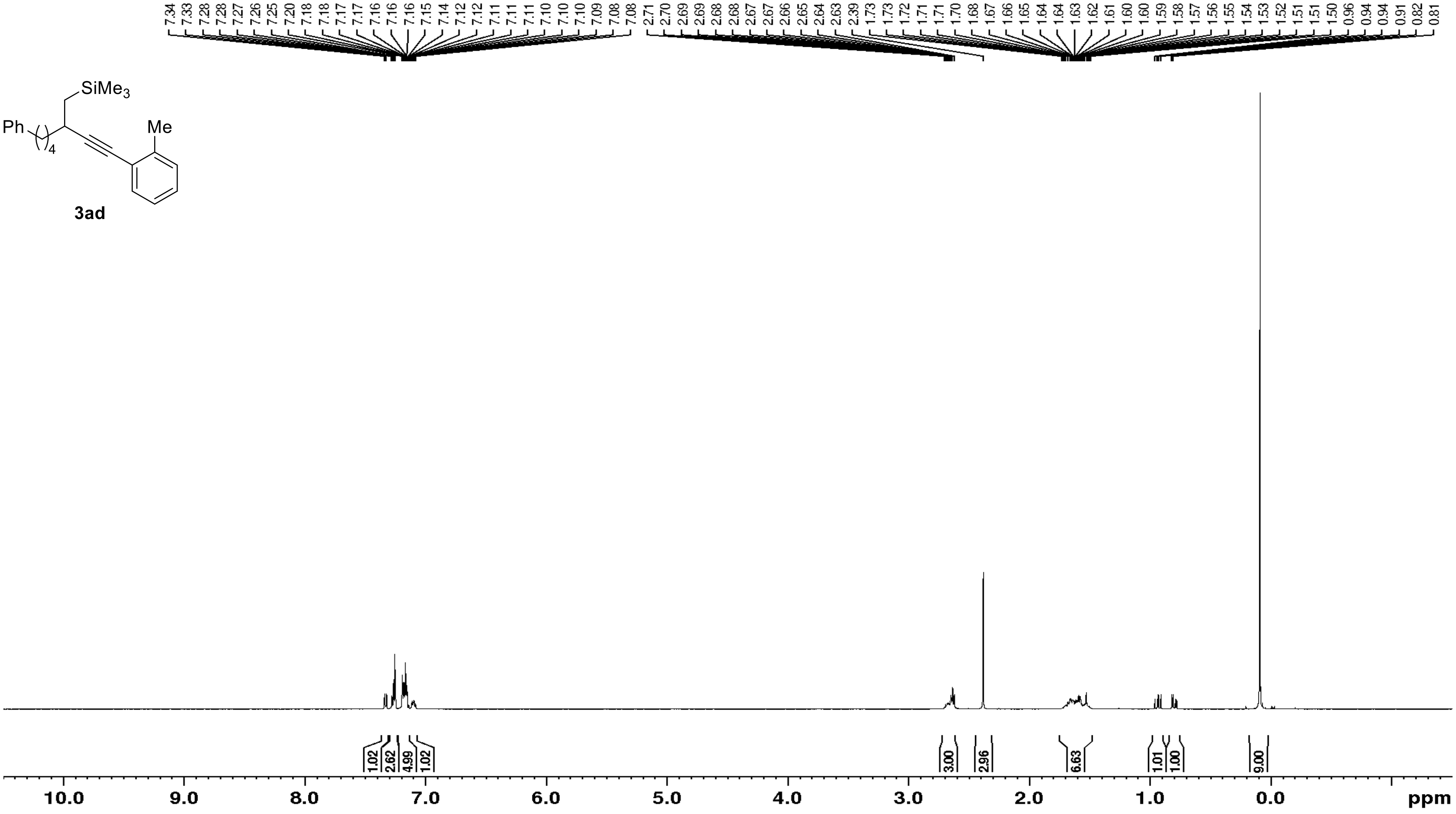

Figure S11. **$^{13}C\{^1H\}$ NMR** spectrum (126 MHz, $CDCl_3$, 298 K) of **3ad** from the reaction of alkene (**1a**) and alkynylsilane (**2d**).

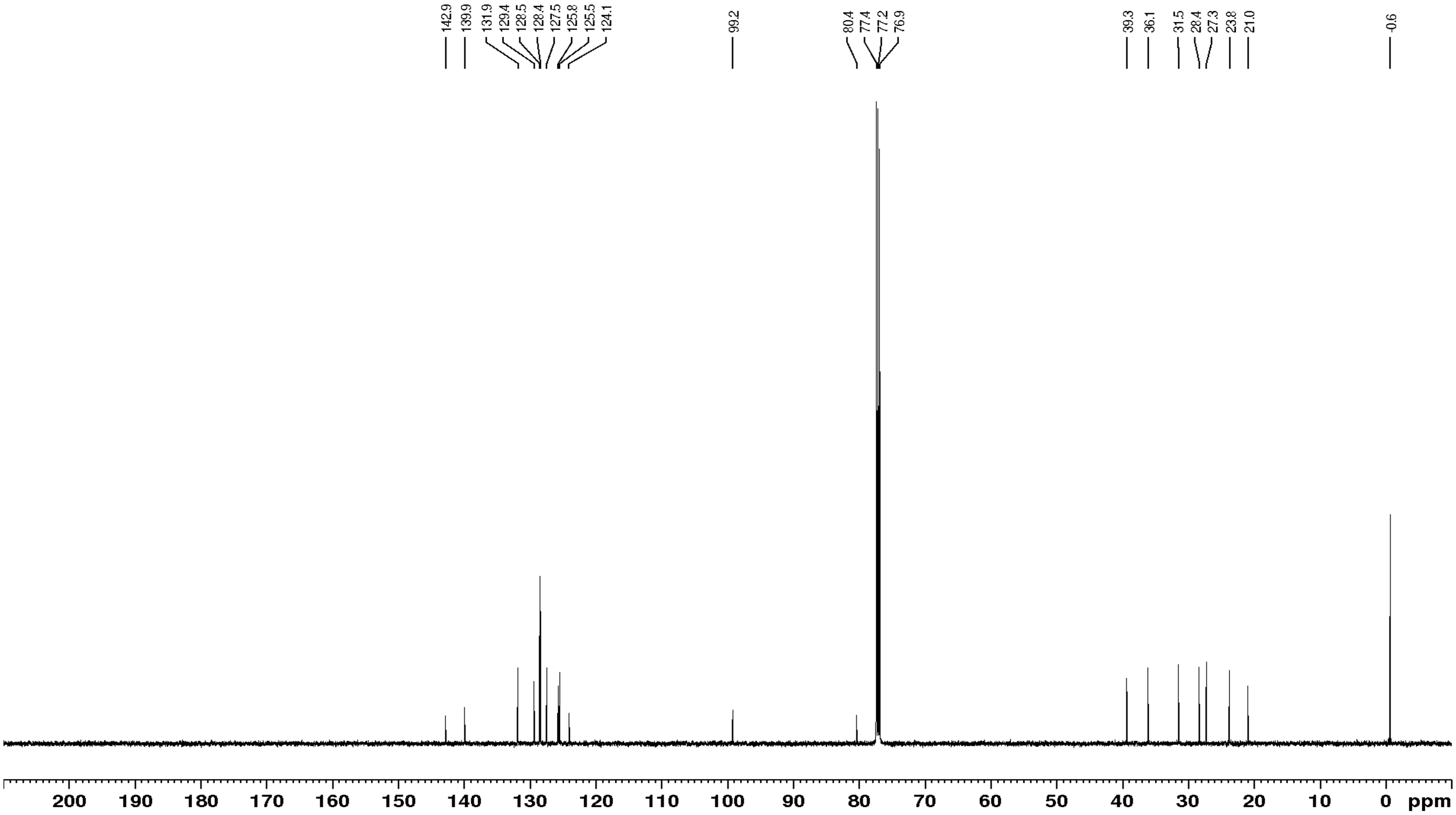

Figure S12. **$^{1}H/^{29}Si$ HMQC** NMR spectrum (500/99 MHz, $CDCl_3$, 298 K, optimized for J = 7 Hz) of **3ad** from the reaction of alkene (**1a**) and alkynylsilane (**2d**).

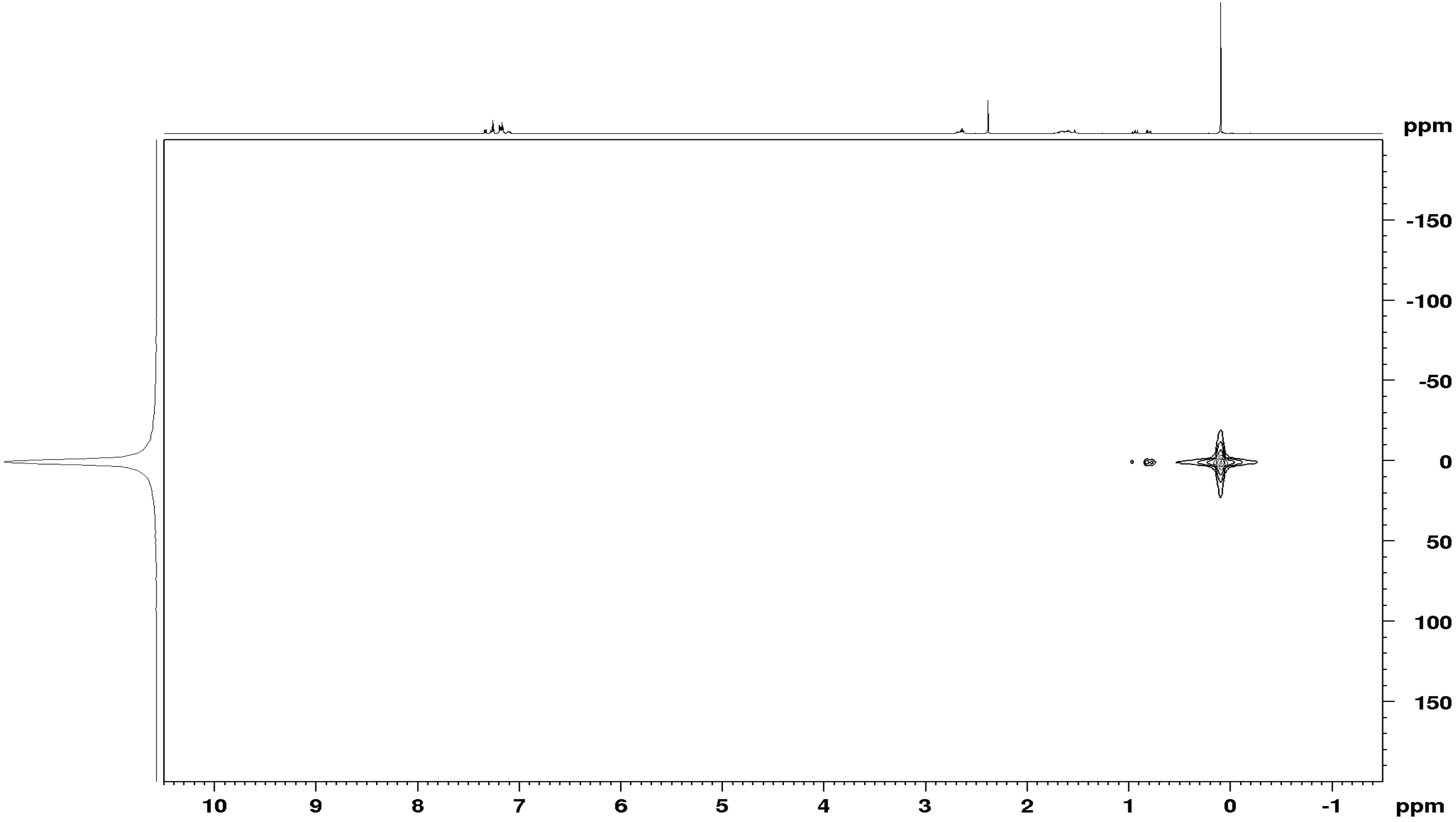

Figure S13. **^{1}H NMR** spectrum (500 MHz, $CDCl_3$, 298 K) of **3ae** from the reaction of alkene (**1a**) and alkynylsilane (**2e**).

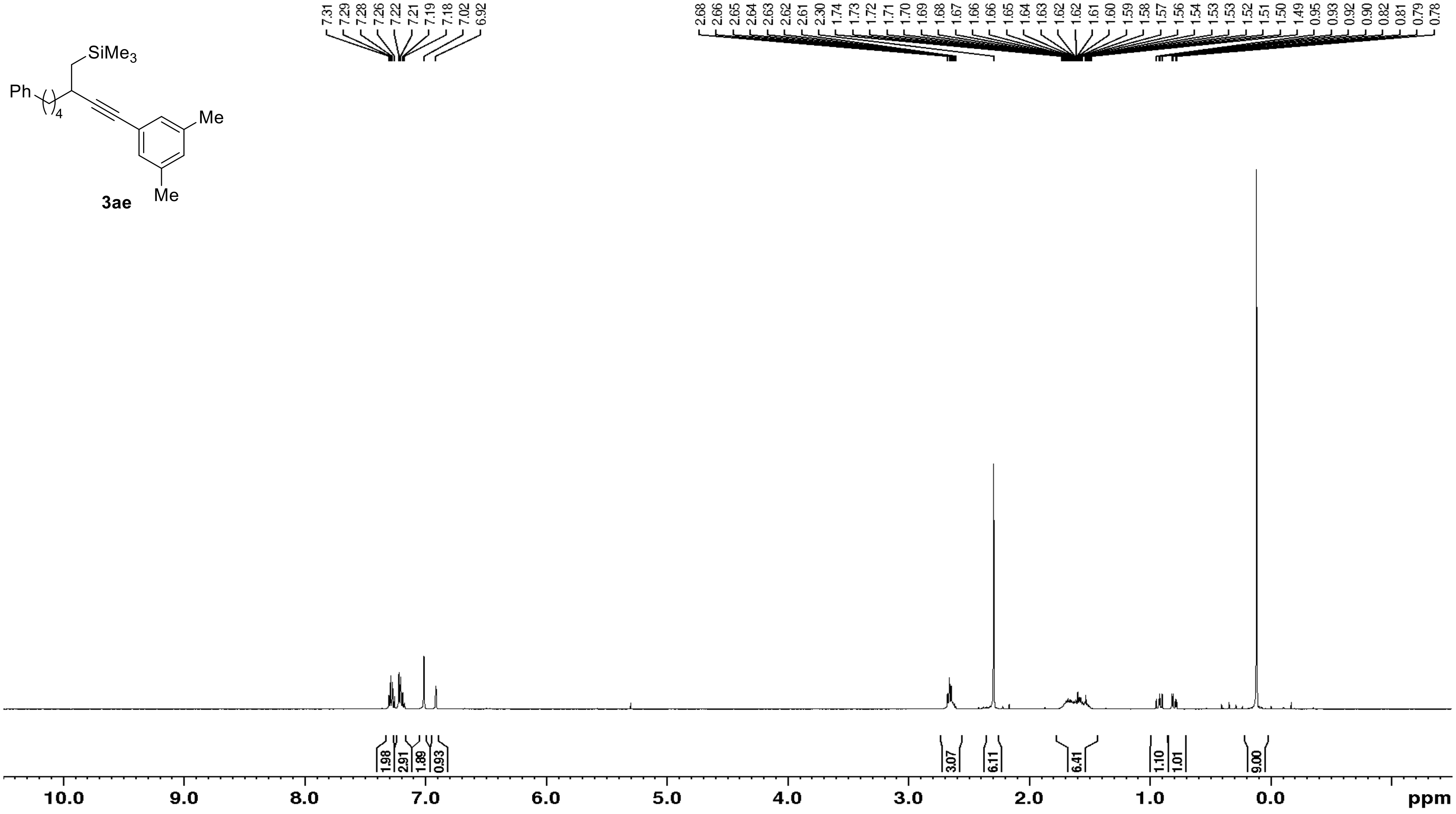

Figure S14. **$^{13}C\{^{1}H\}$ NMR** spectrum (126 MHz, $CDCl_3$, 298 K) of **3ae** from the reaction of alkene (**1a**) and alkynylsilane (**2e**).

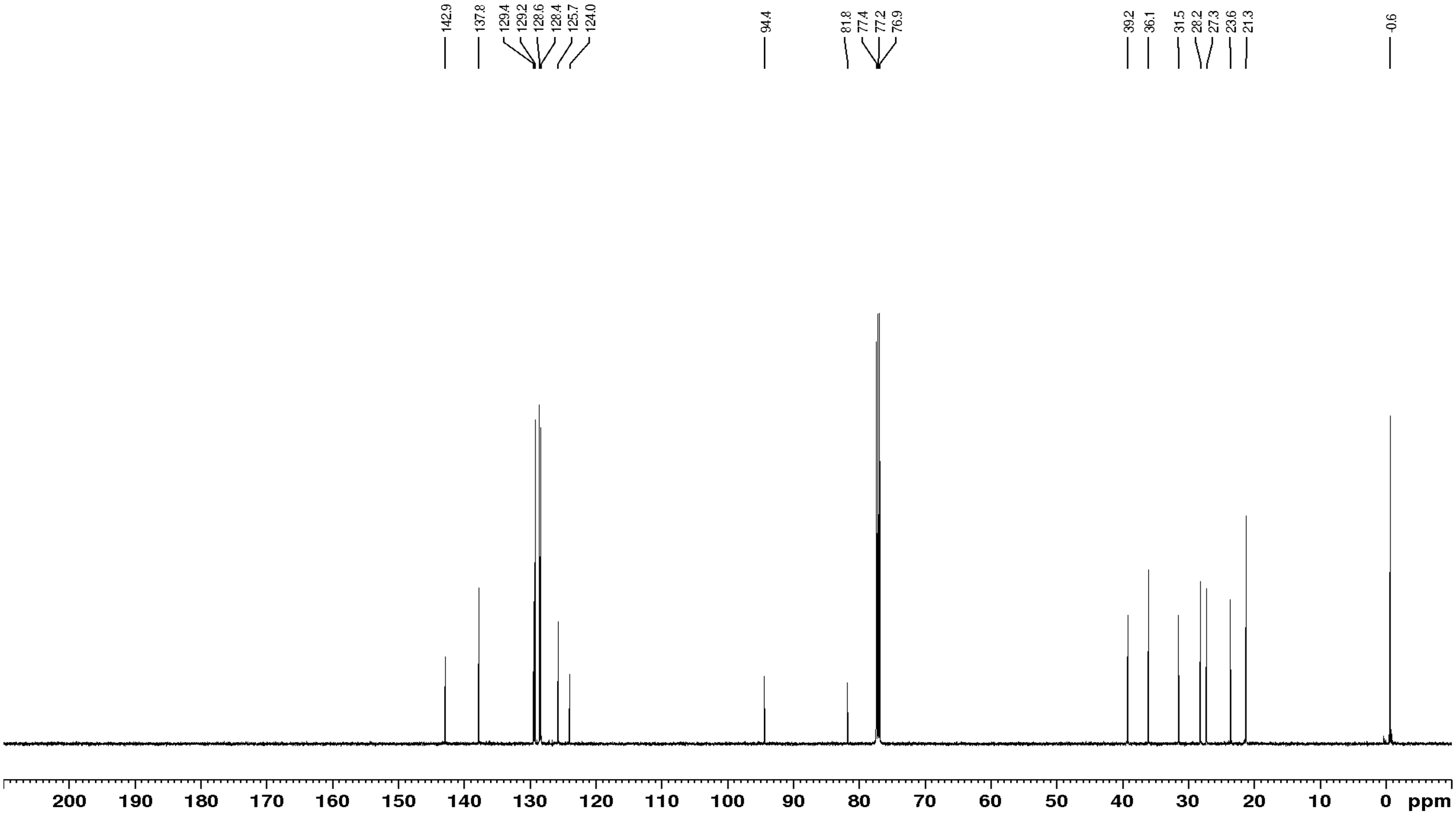

Figure S15. **$^{1}H/^{29}Si$ HMQC** NMR spectrum (500/99 MHz, $CDCl_3$, 298 K, optimized for J = 7 Hz) of **3ae** from the reaction of alkene (**1a**) and alkynylsilane (**2e**).

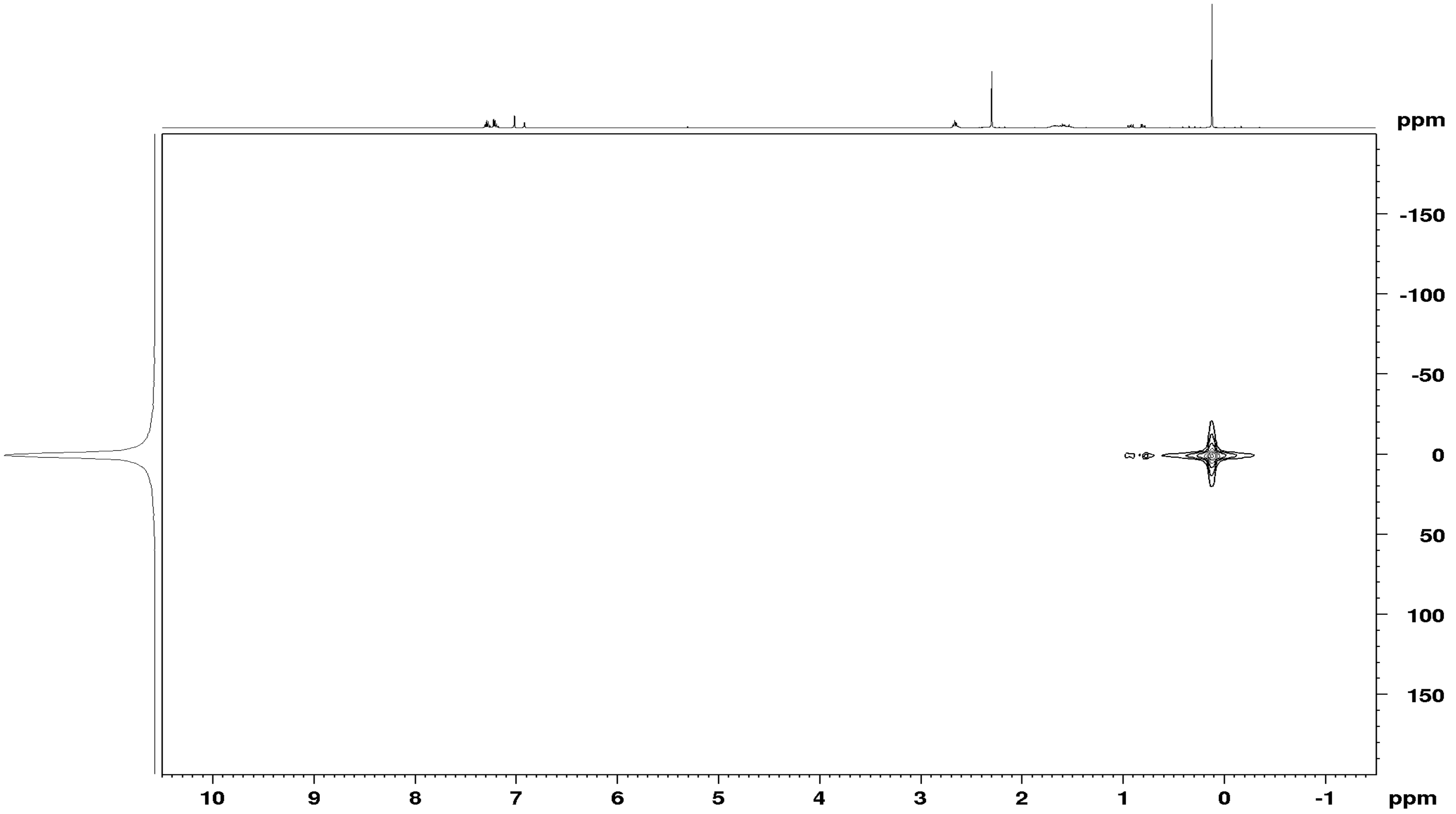

Figure S16. **^{1}H NMR** spectrum (500 MHz, $CDCl_3$, 298 K) of **3af** from the reaction of alkene (**1a**) and alkynylsilane (**2f**).

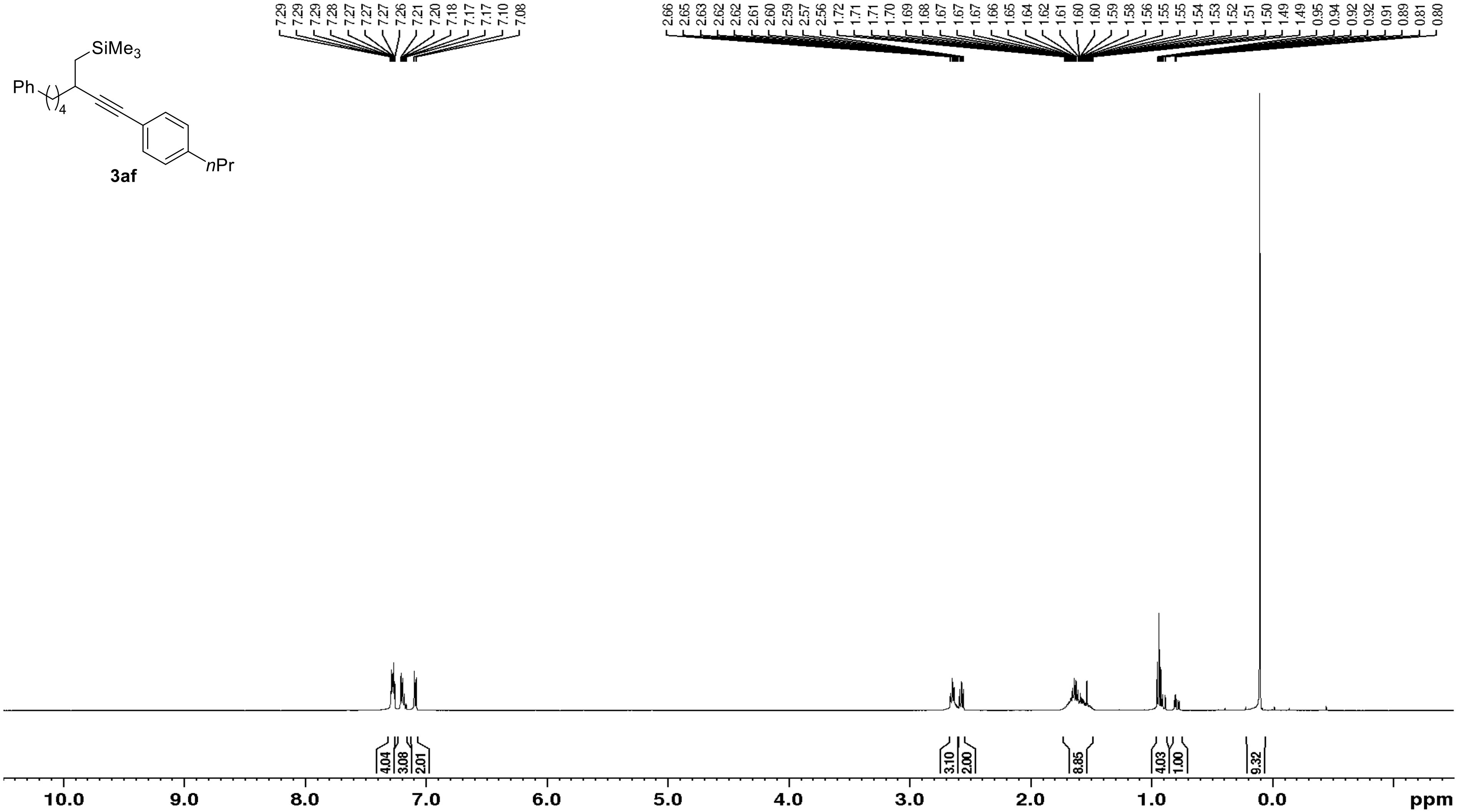

Figure S17. **$^{13}C\{^{1}H\}$ NMR** spectrum (126 MHz, $CDCl_3$, 298 K) of **3af** from the reaction of alkene (**1a**) and alkynylsilane (**2f**).

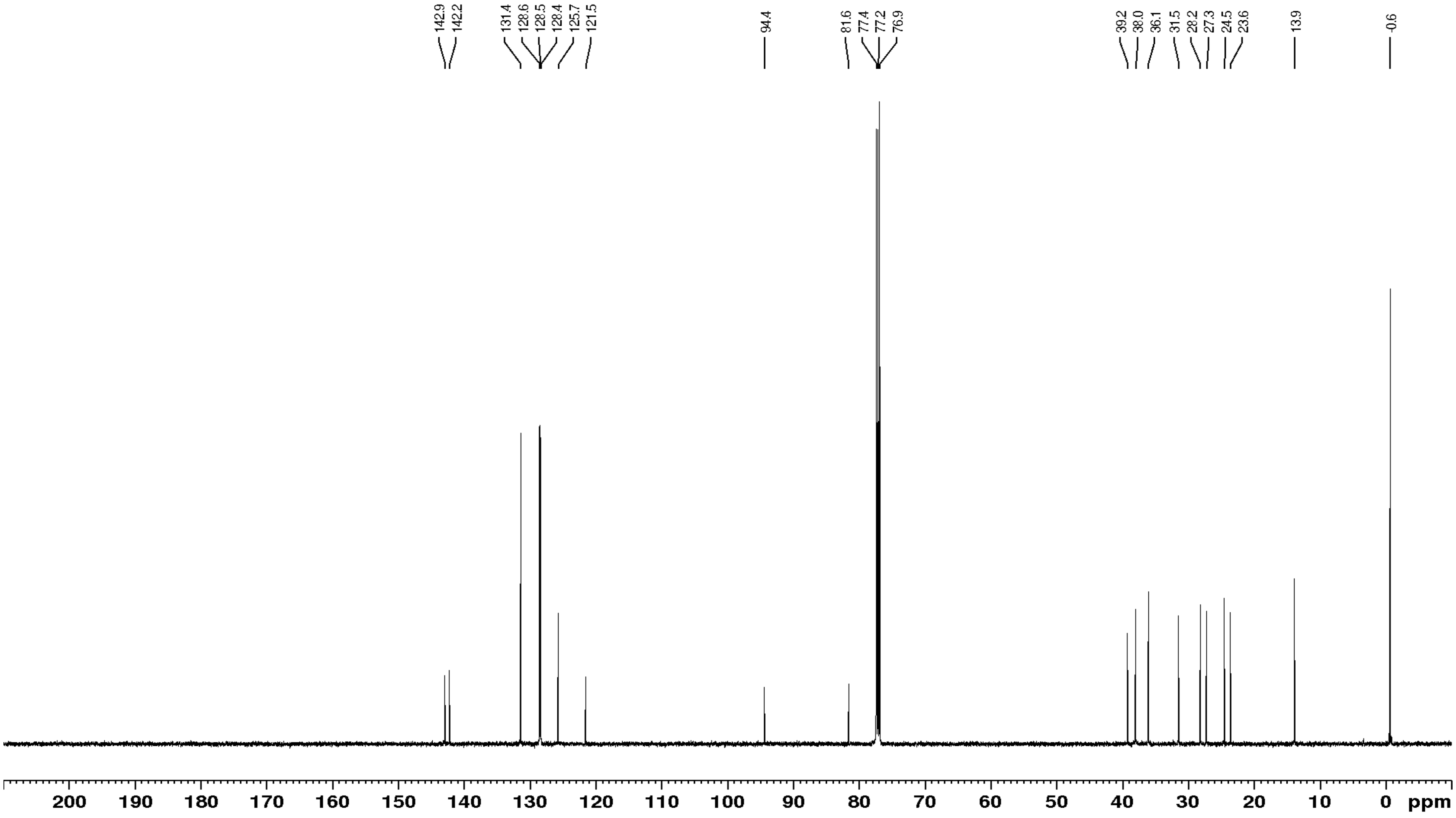

Figure S18. **$^1H/^{29}Si$ HMQC** NMR spectrum (500/99 MHz, $CDCl_3$, 298 K, optimized for *J* = 7 Hz) of **3af** from the reaction of alkene (**1a**) and alkynylsilane (**2f**).

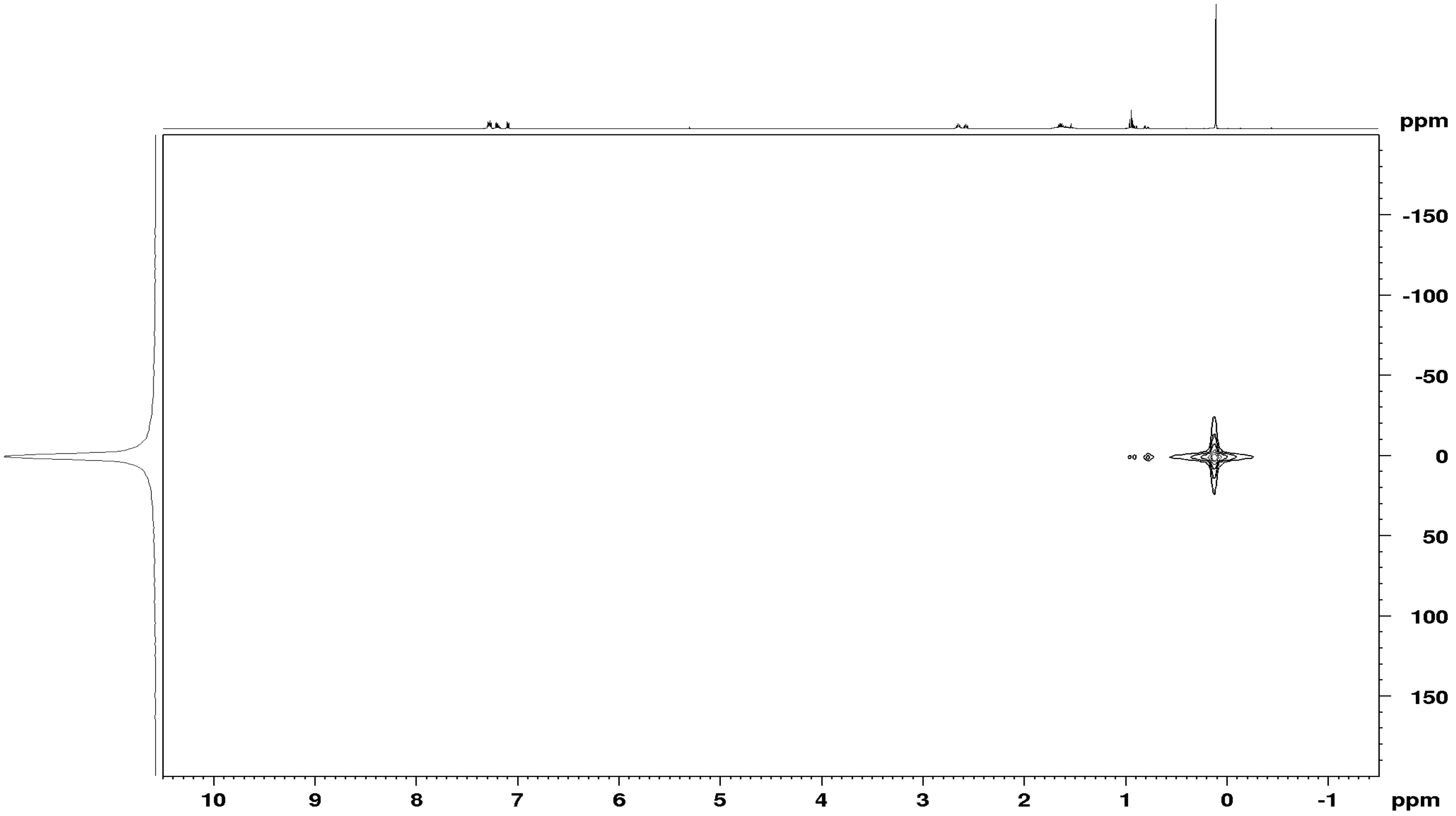

Figure S19. **^{1}H NMR** spectrum (500 MHz, $CDCl_3$, 298 K) of **3ag** from the reaction of alkene (**1a**) and alkynylsilane (**2g**).

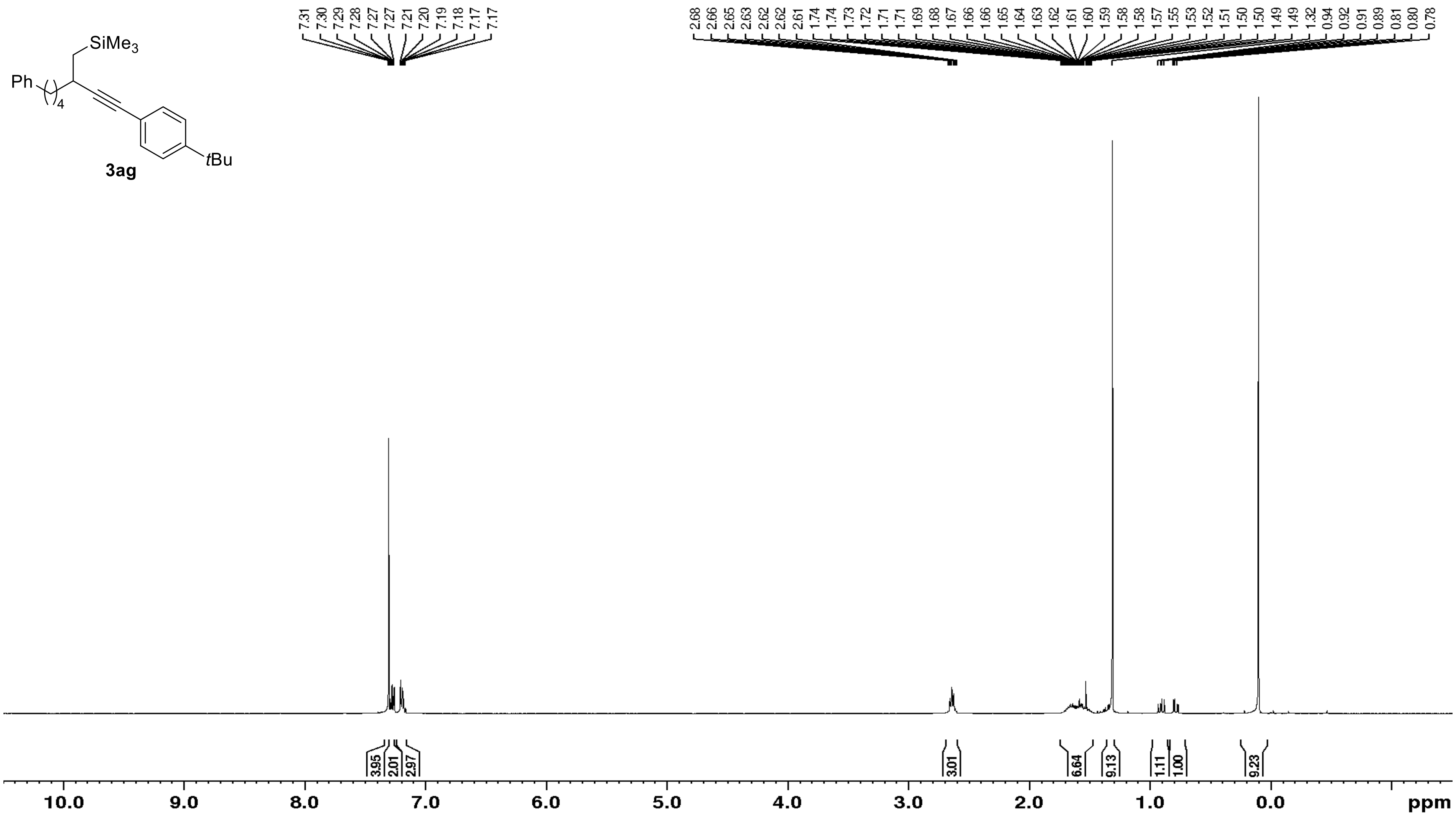

Figure S20. **$^{13}C\{^1H\}$ NMR** spectrum (126 MHz, $CDCl_3$, 298 K) of **3ag** from the reaction of alkene (**1a**) and alkynylsilane (**2g**).

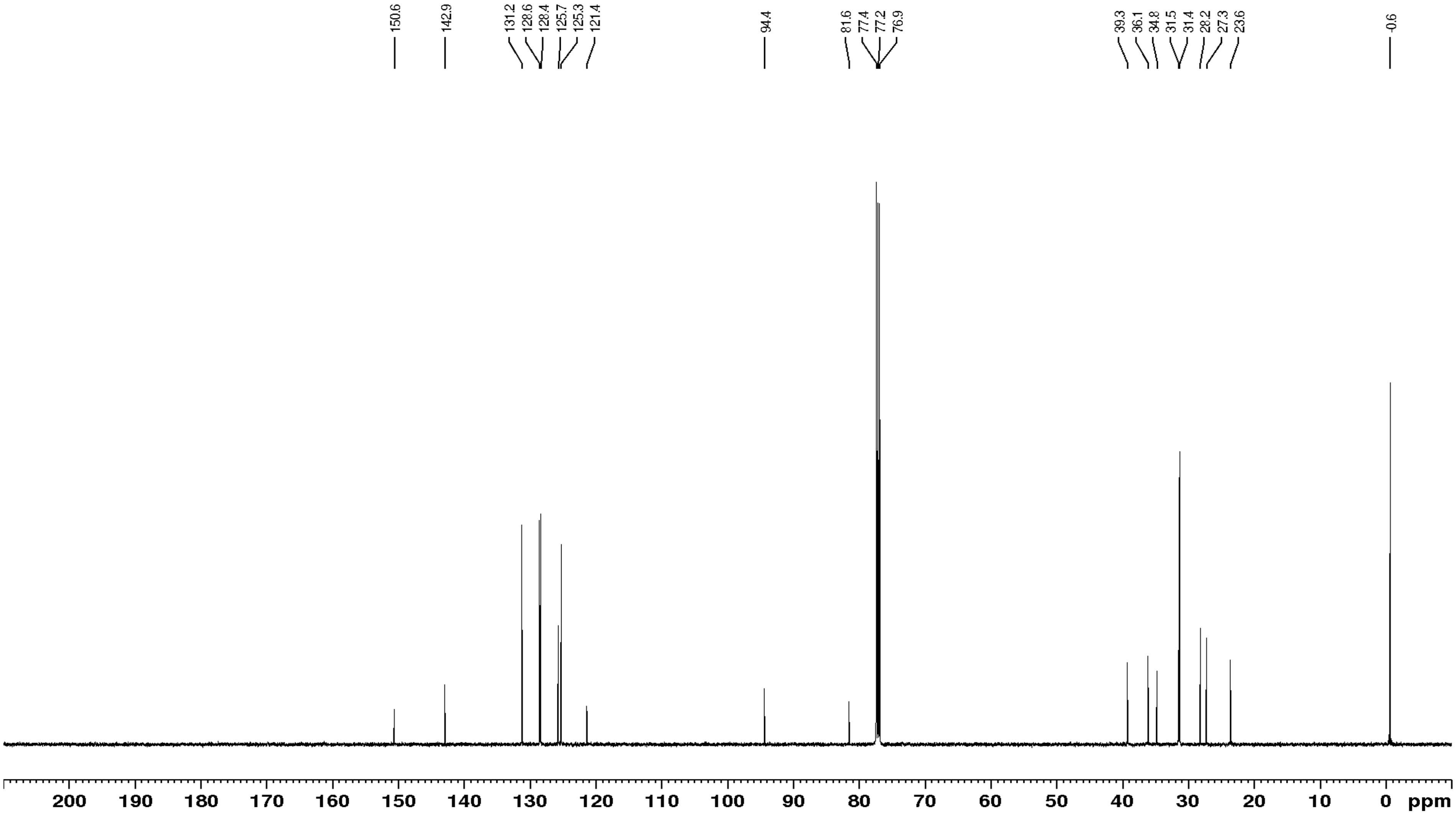

Figure S21. **^{1}H/^{29}Si HMQC** NMR spectrum (500/99 MHz, $CDCl_3$, 298 K, optimized for *J* = 7 Hz) of **3ag** from the reaction of alkene (**1a**) and alkynylsilane (**2g**).

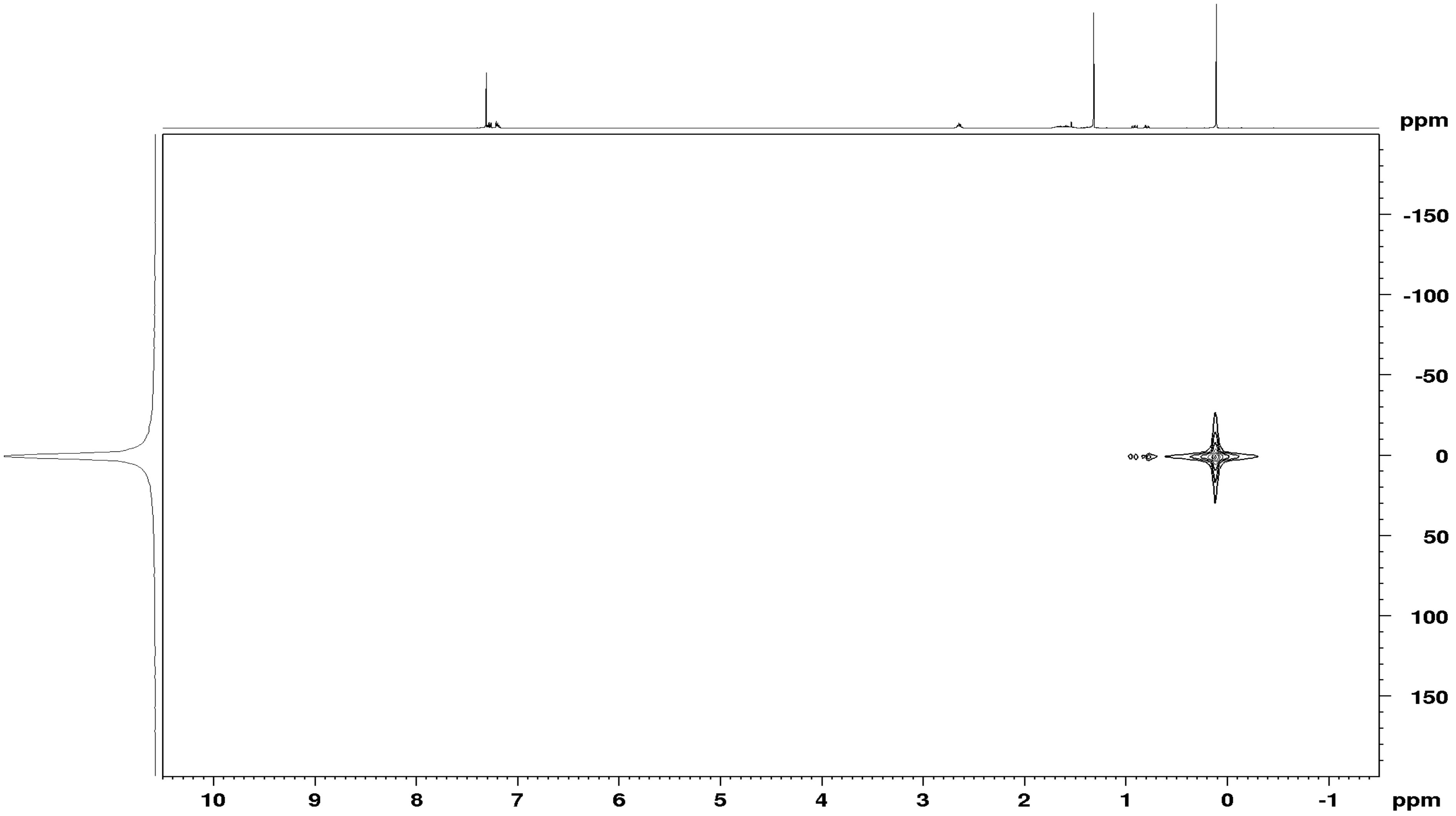

Figure S22. **^{1}H NMR** spectrum (500 MHz, $CDCl_3$, 298 K) of **3ah** from the reaction of alkene (**1a**) and alkynylsilane (**2h**).

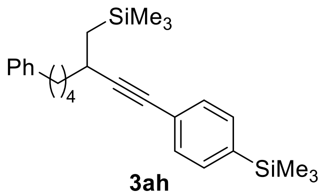

3ah

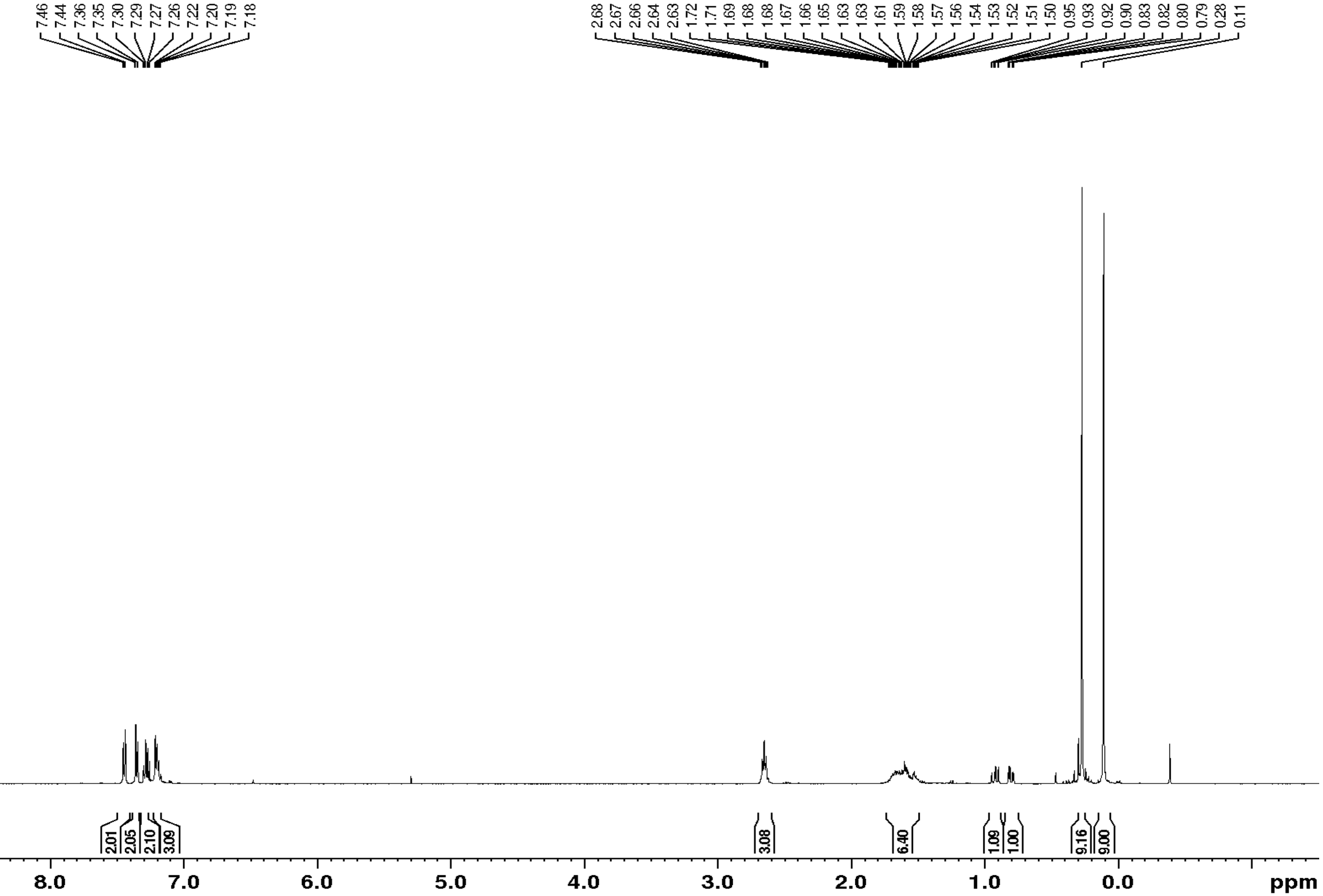

Figure S23. **$^{13}C\{^{1}H\}$ NMR** spectrum (126 MHz, $CDCl_3$, 298 K) of **3ah** from the reaction of alkene (**1a**) and alkynylsilane (**2h**).

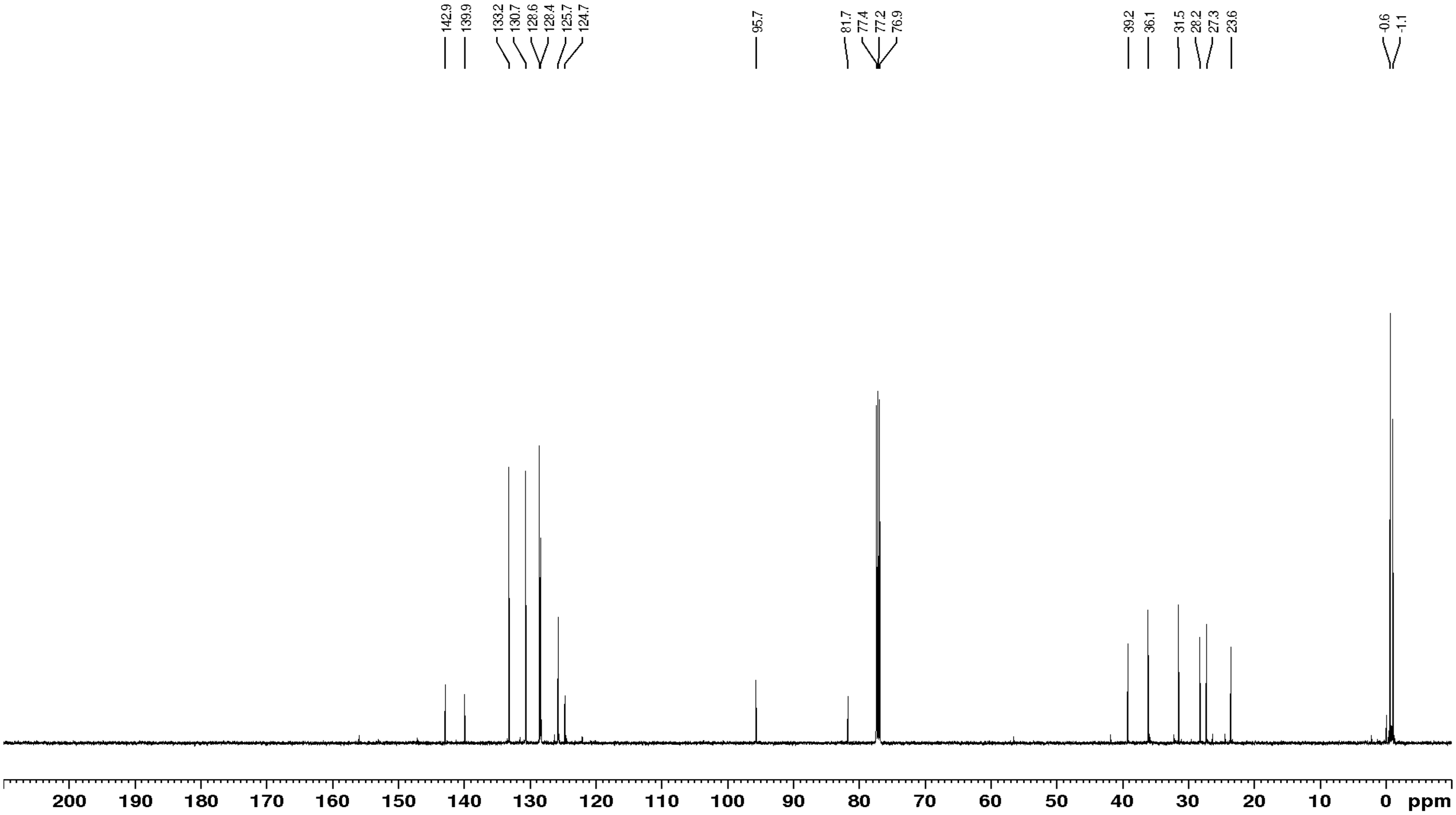

Figure S24. **$^1H/^{29}Si$ HMQC** NMR spectrum (500/99 MHz, $CDCl_3$, 298 K, optimized for *J* = 7 Hz) of **3ah** from the reaction of alkene (**1a**) and alkynylsilane (**2h**).

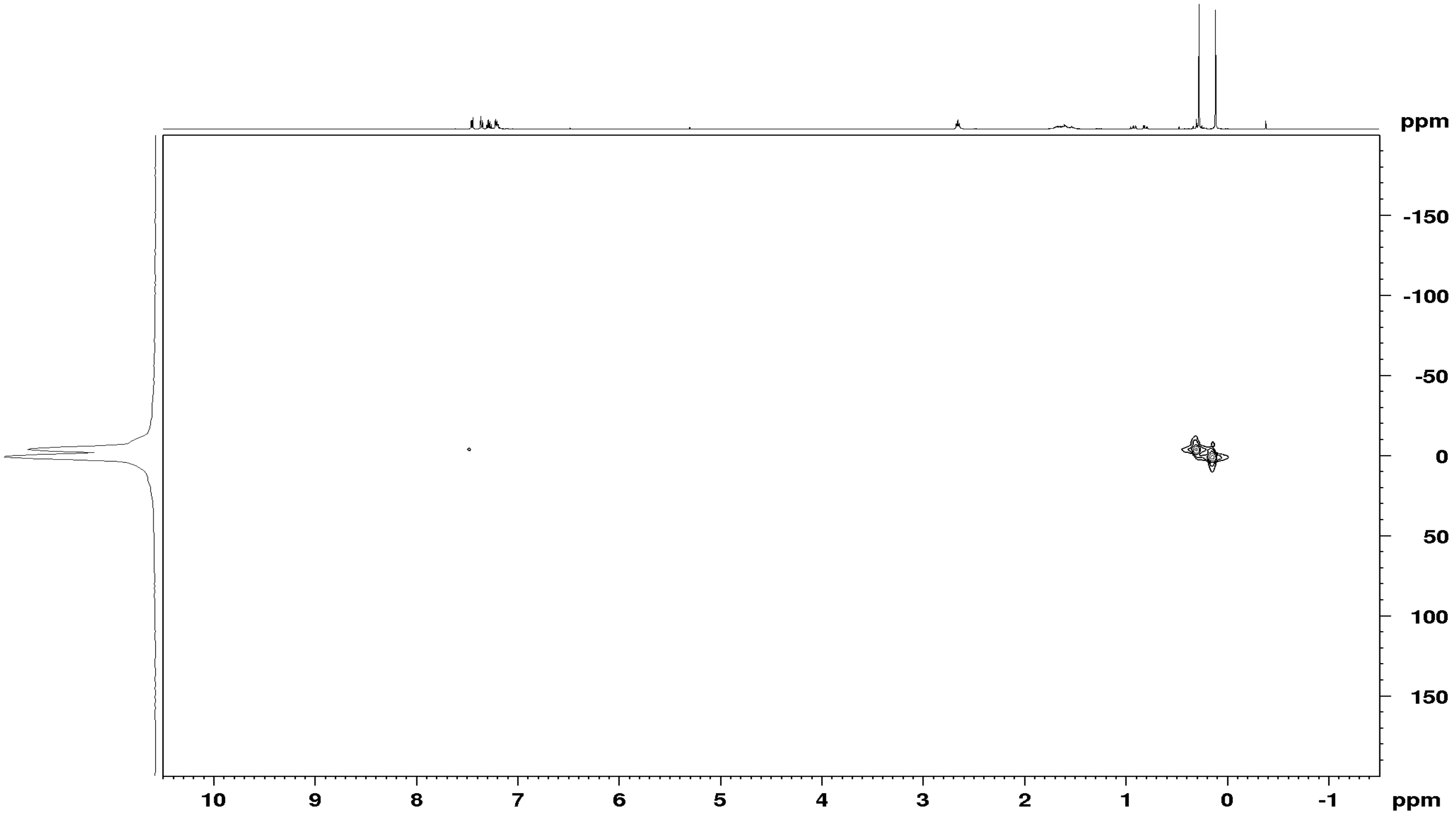

Figure S25. **^{1}H NMR** spectrum (500 MHz, $CDCl_3$, 298 K) of **3ai** from the reaction of alkene (**1a**) and alkynylsilane (**2i**).

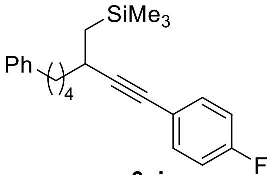

3ai

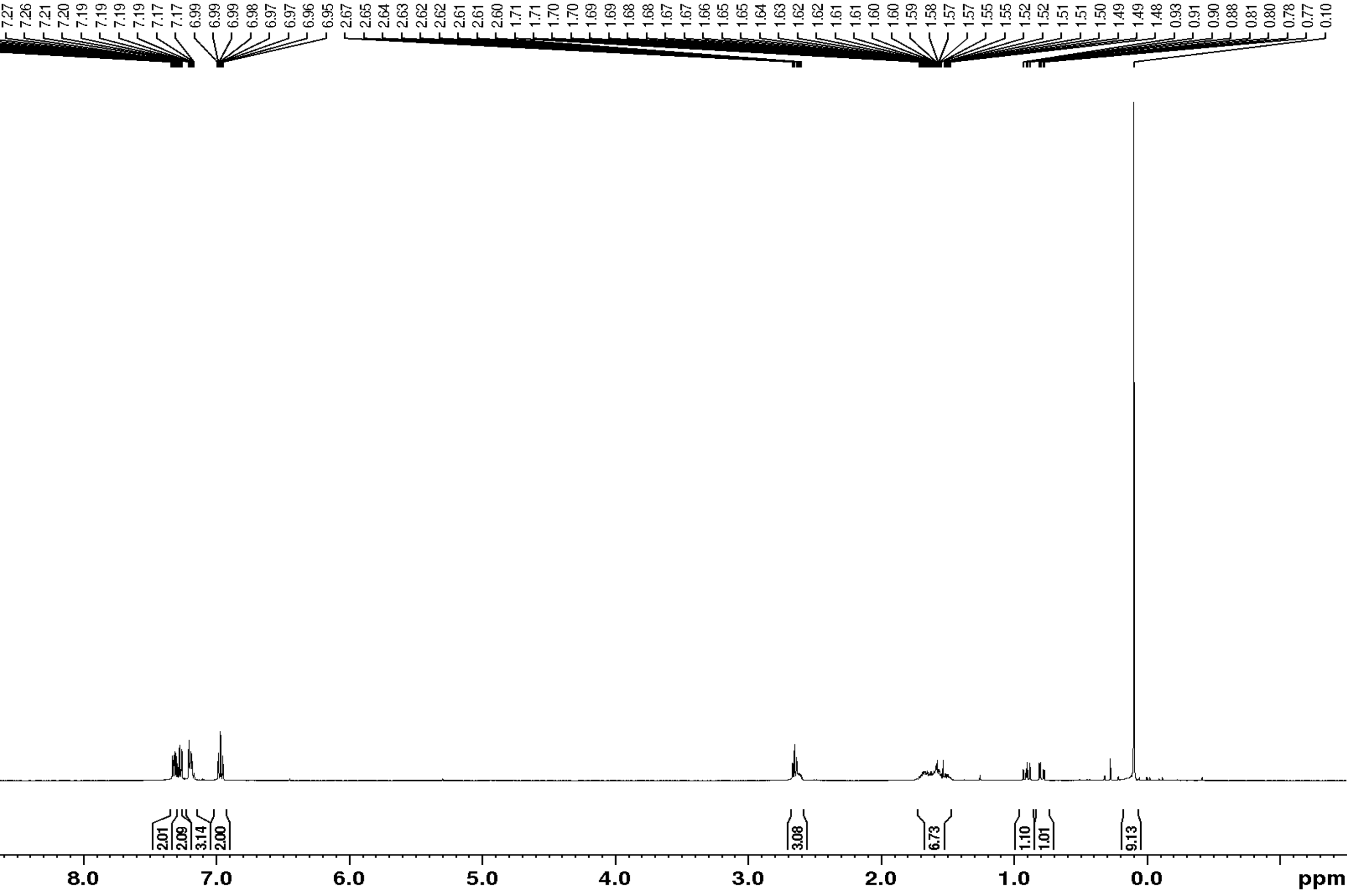

Figure S26. **$^{13}C\{^1H\}$ NMR** spectrum (126 MHz, $CDCl_3$, 298 K) of **3ai** from the reaction of alkene (**1a**) and alkynylsilane (**2i**).

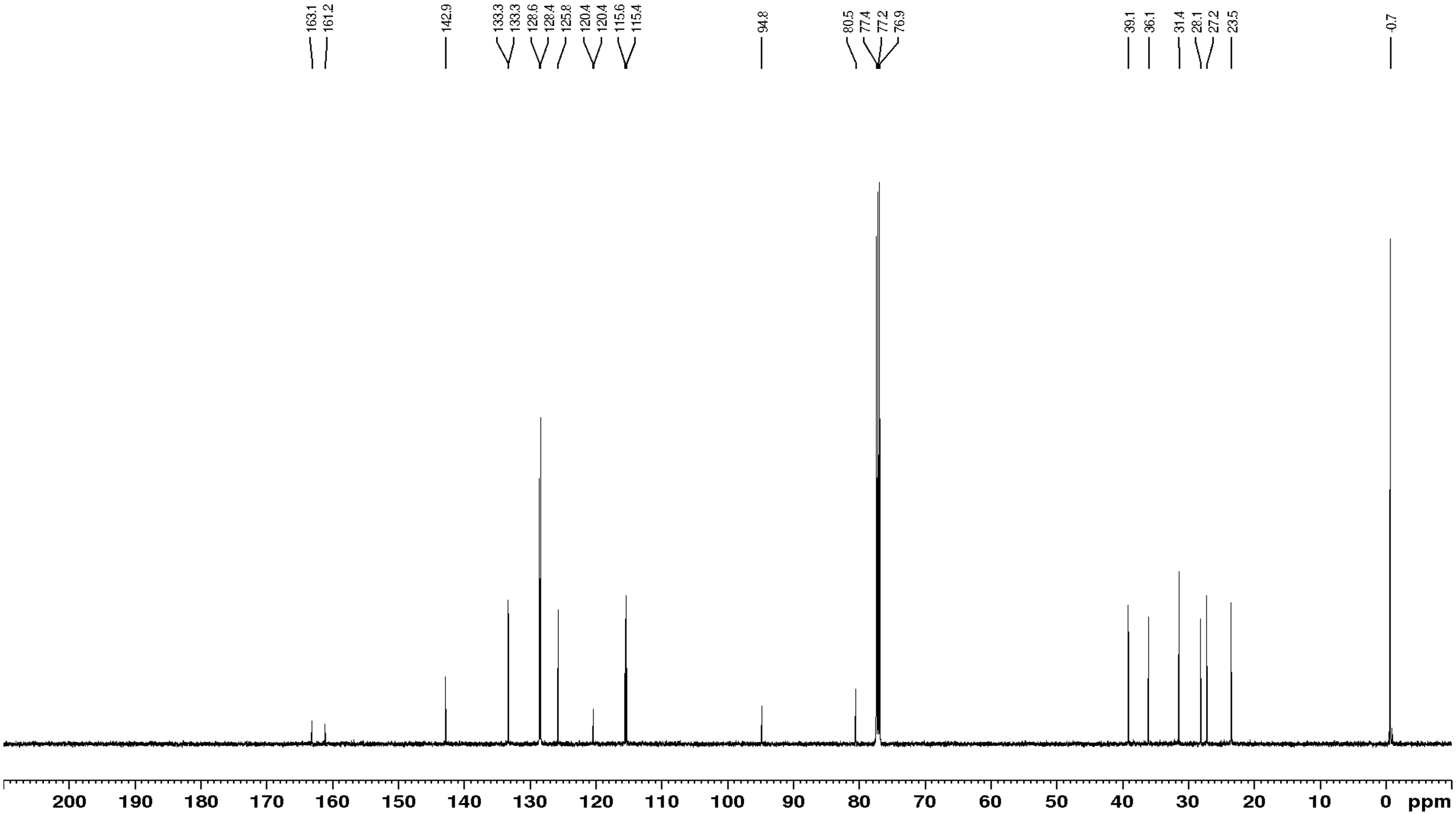

Figure S27. **$^{1}H/^{29}Si$ HMQC** NMR spectrum (500/99 MHz, $CDCl_3$, 298 K, optimized for *J* = 7 Hz) of **3ai** from the reaction of alkene (**1a**) and alkynylsilane (**2i**).

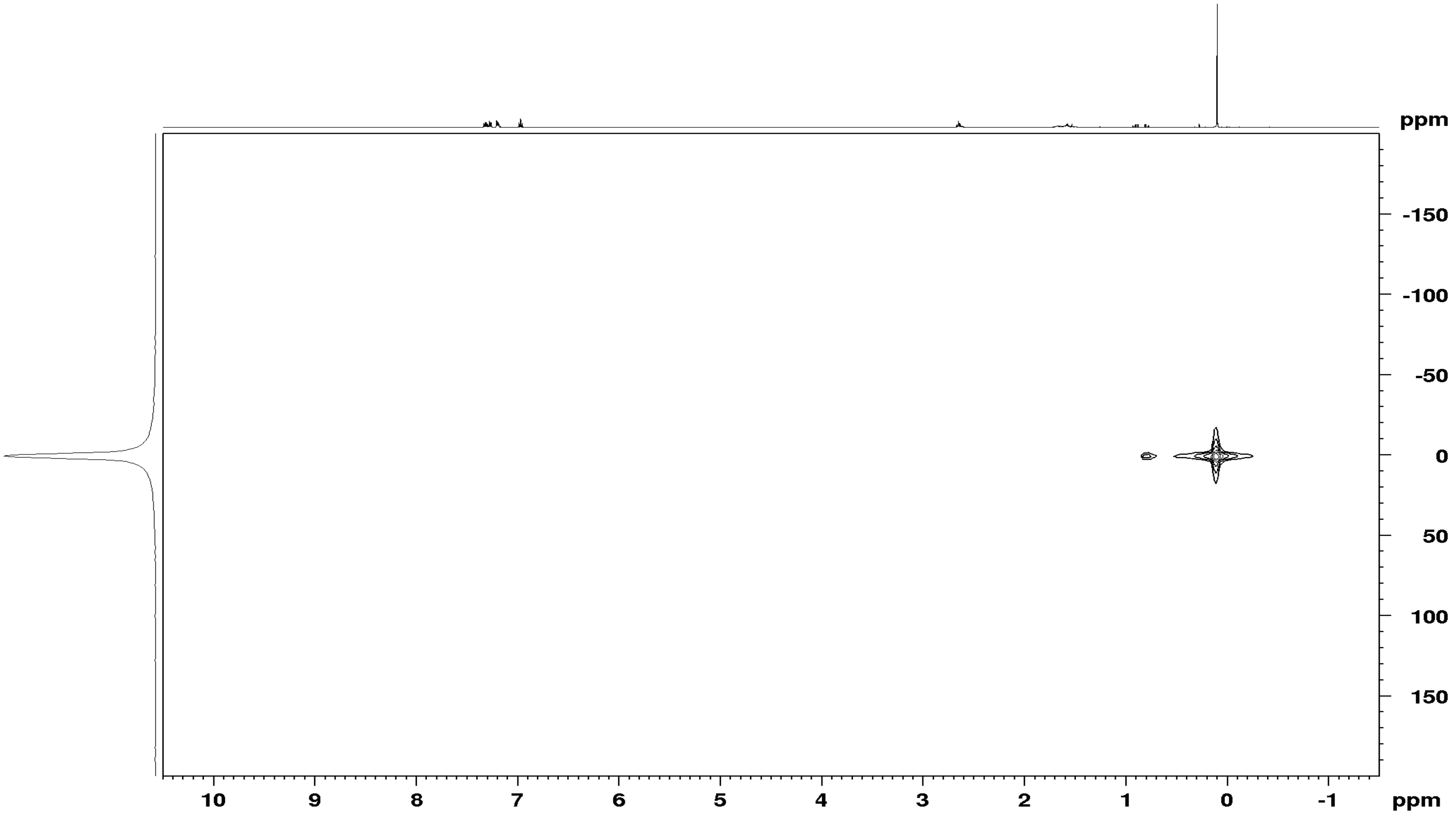

Figure S28. [19]**F NMR** spectrum (471 MHz, $CDCl_3$, 298 K) of **3ai** from the reaction of alkene (**1a**) and alkynylsilane (**2i**).

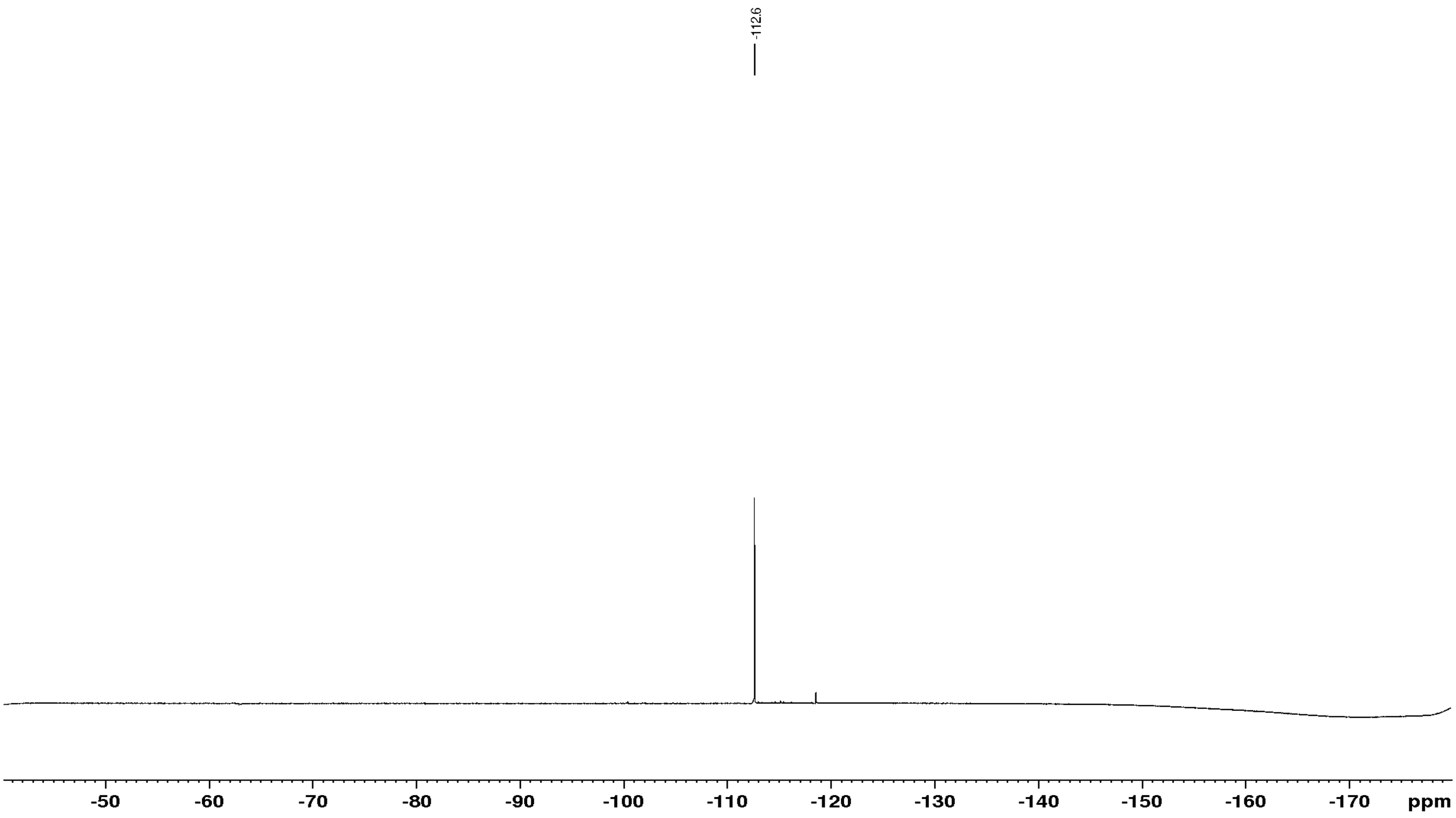

Figure S29. **^{1}H NMR** spectrum (500 MHz, $CDCl_3$, 298 K) of **3aj** from the reaction of alkene (**1a**) and alkynylsilane (**2j**).

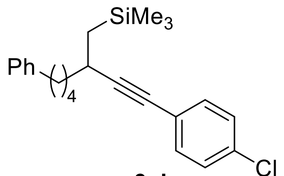

3aj

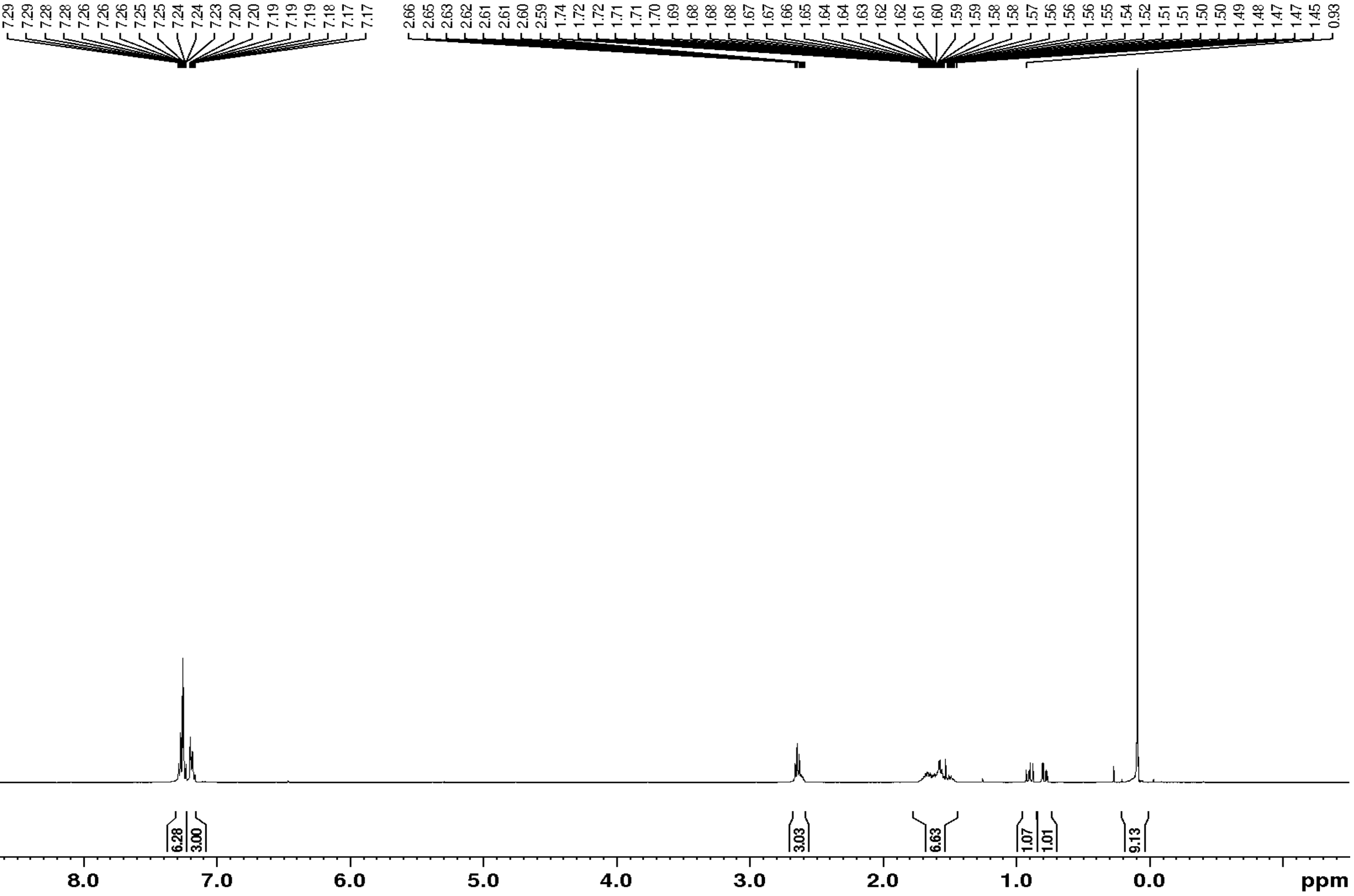

Figure S30. **$^{13}C\{^{1}H\}$ NMR** spectrum (126 MHz, $CDCl_3$, 298 K) of **3aj** from the reaction of alkene (**1a**) and alkynylsilane (**2j**).

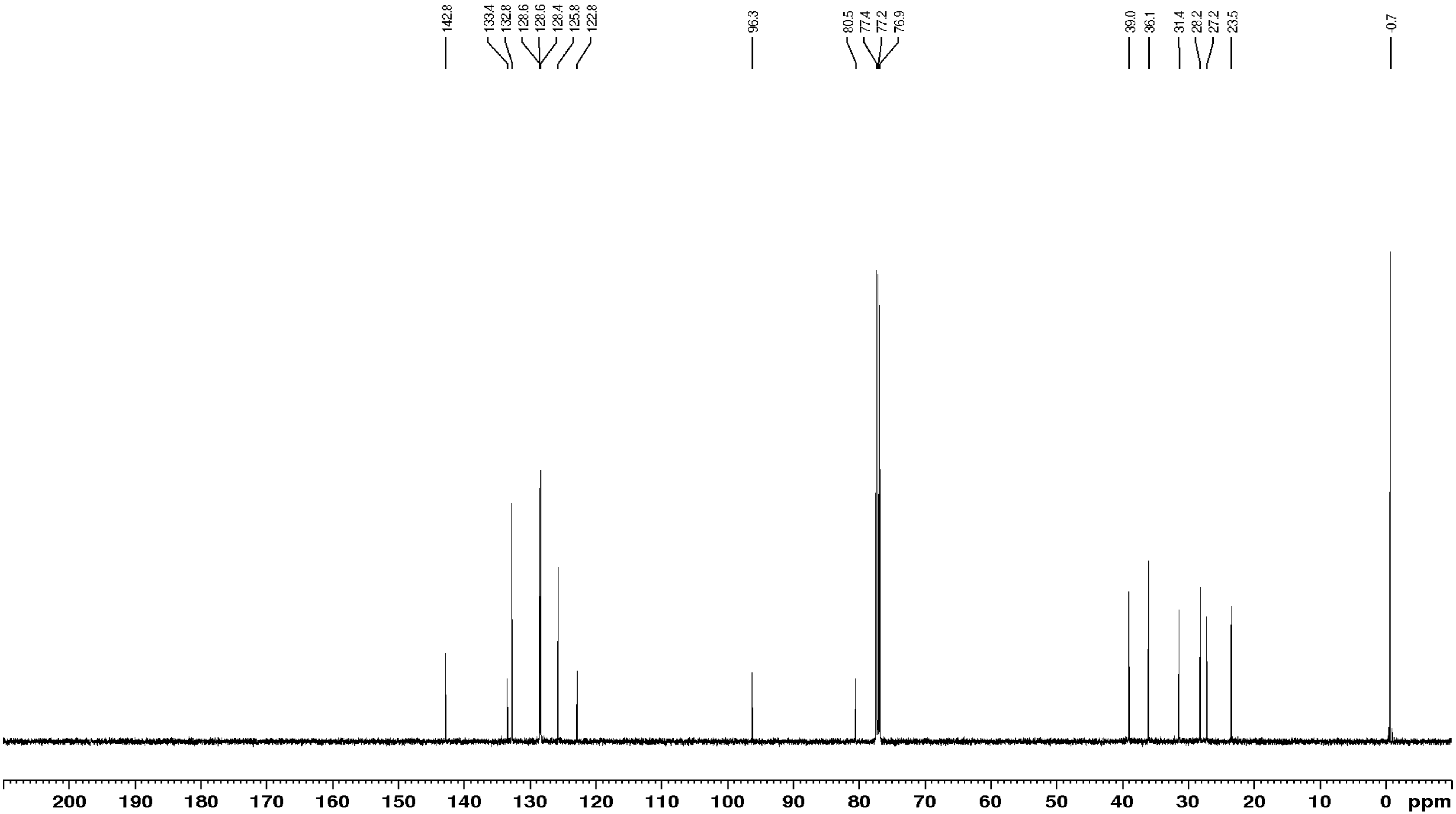

Figure S31. **$^{1}H/^{29}Si$ HMQC** NMR spectrum (500/99 MHz, $CDCl_3$, 298 K, optimized for *J* = 7 Hz) of **3aj** from the reaction of alkene (**1a**) and alkynylsilane (**2j**).

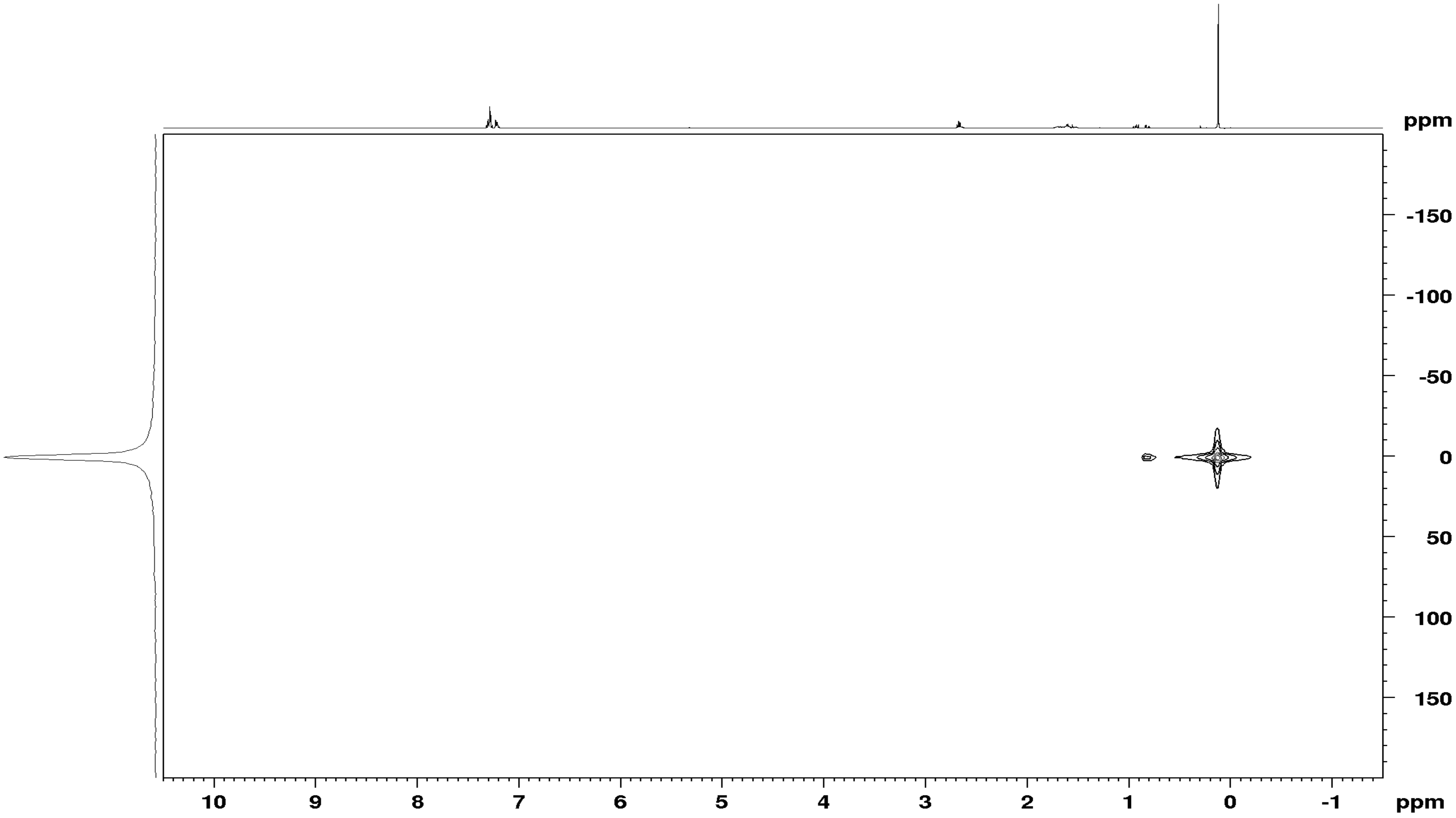

Figure S32. **^{1}H NMR** spectrum (500 MHz, $CDCl_3$, 298 K) of **3ak** from the reaction of alkene (**1a**) and alkynylsilane (**2k**).

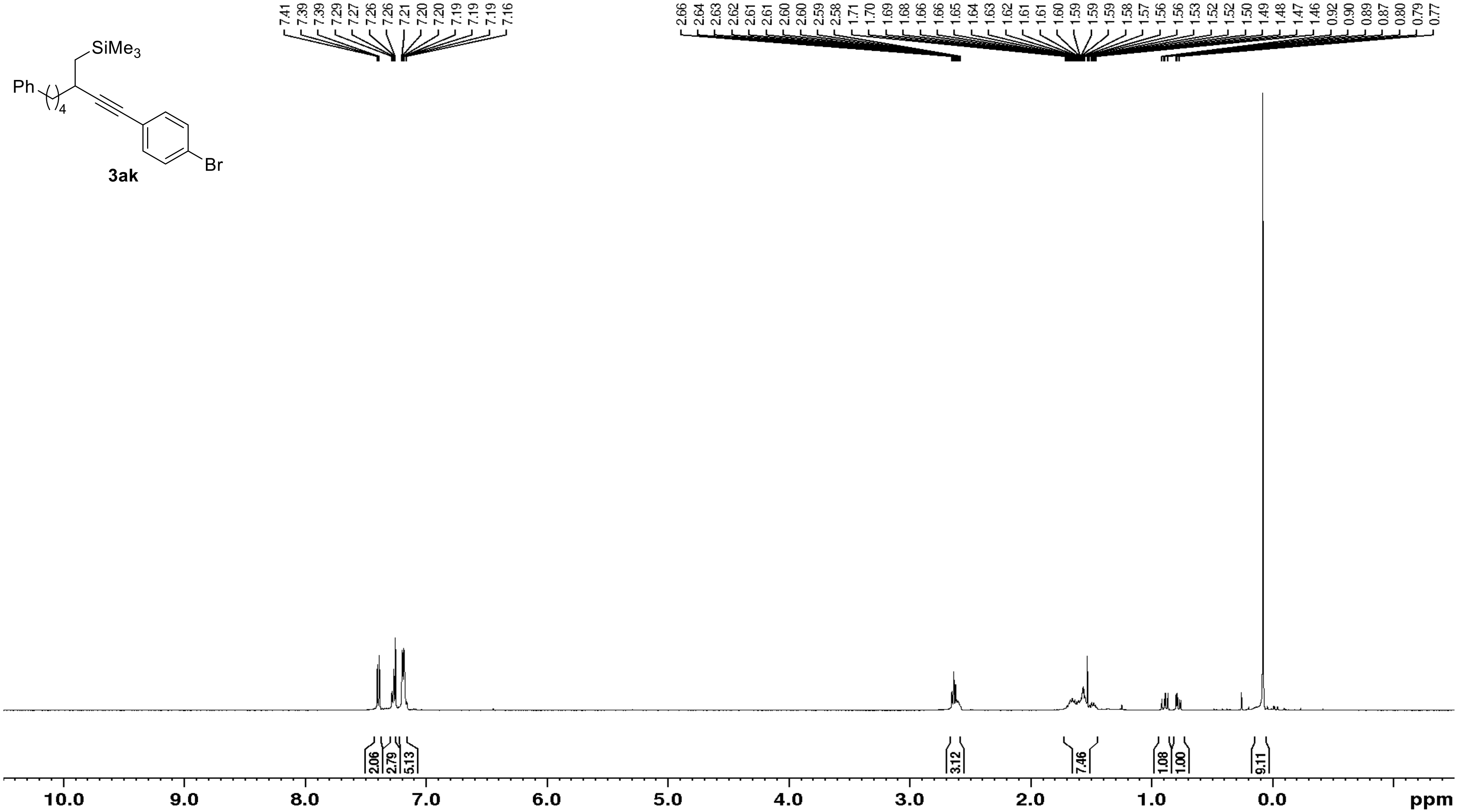

Figure S33. **$^{13}C\{^{1}H\}$ NMR** spectrum (126 MHz, $CDCl_3$, 298 K) of **3ak** from the reaction of alkene (**1a**) and alkynylsilane (**2k**).

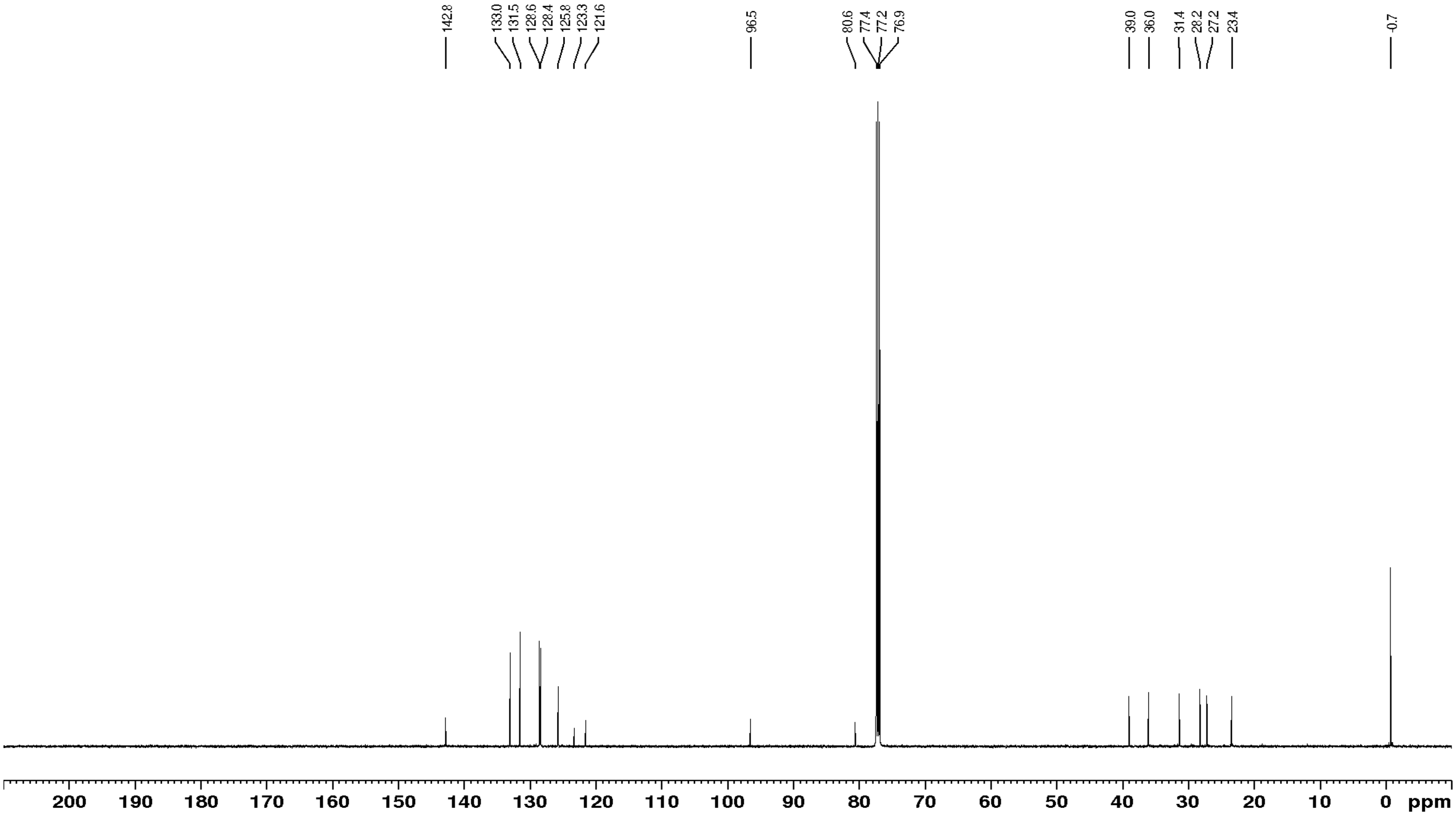

Figure S34. **$^{1}H/^{29}Si$ HMQC** NMR spectrum (500/99 MHz, $CDCl_3$, 298 K, optimized for *J* = 7 Hz) of **3ak** from the reaction of alkene (**1a**) and alkynylsilane (**2k**).

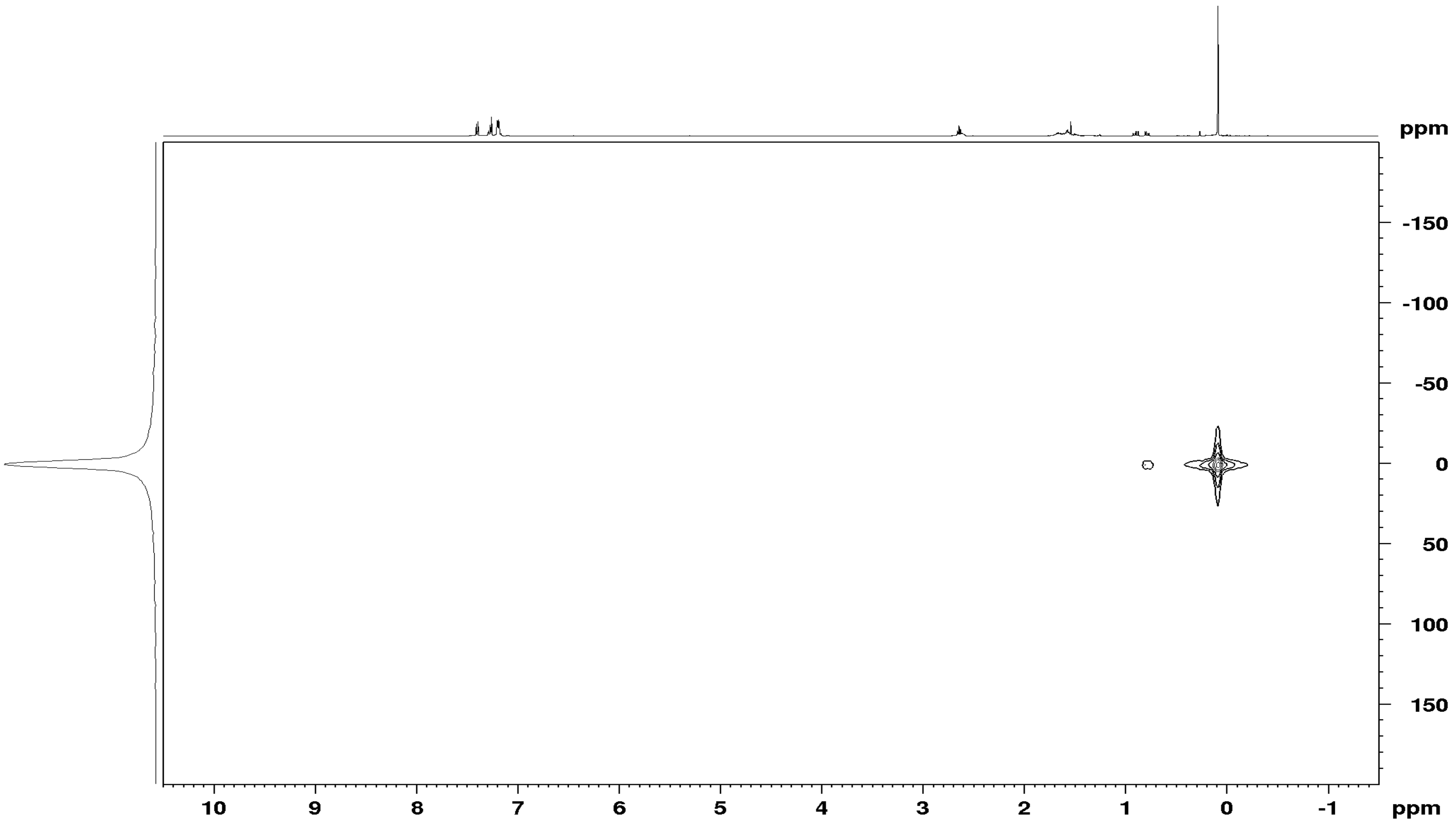

Figure S35. **¹H NMR** spectrum (500 MHz, $CDCl_3$, 298 K) of **3al** from the reaction of alkene (**1a**) and alkynylsilane (**2l**).

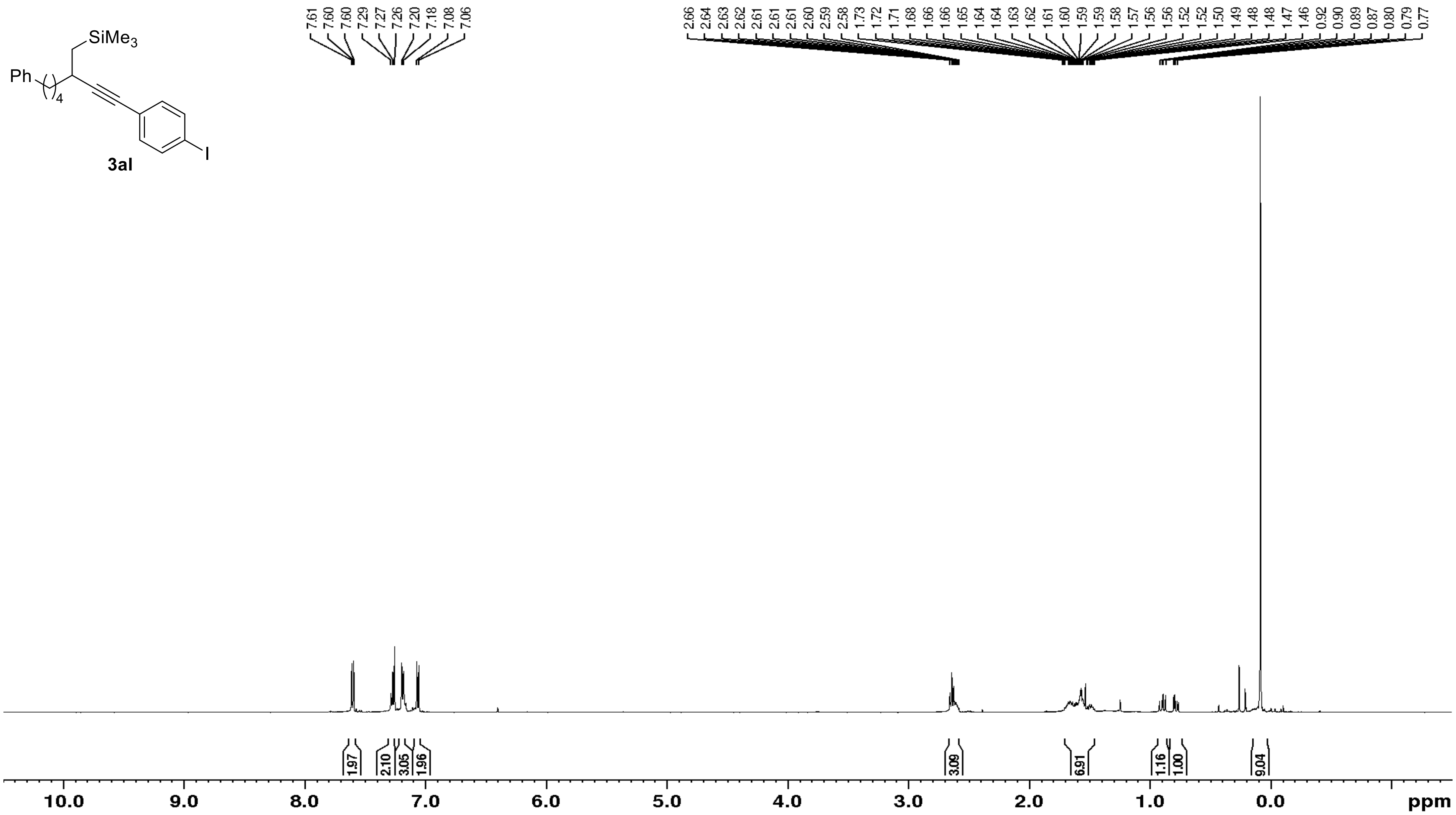

Figure S36. **$^{13}C\{^1H\}$ NMR** spectrum (126 MHz, $CDCl_3$, 298 K) of **3al** from the reaction of alkene (**1a**) and alkynylsilane (**2l**).

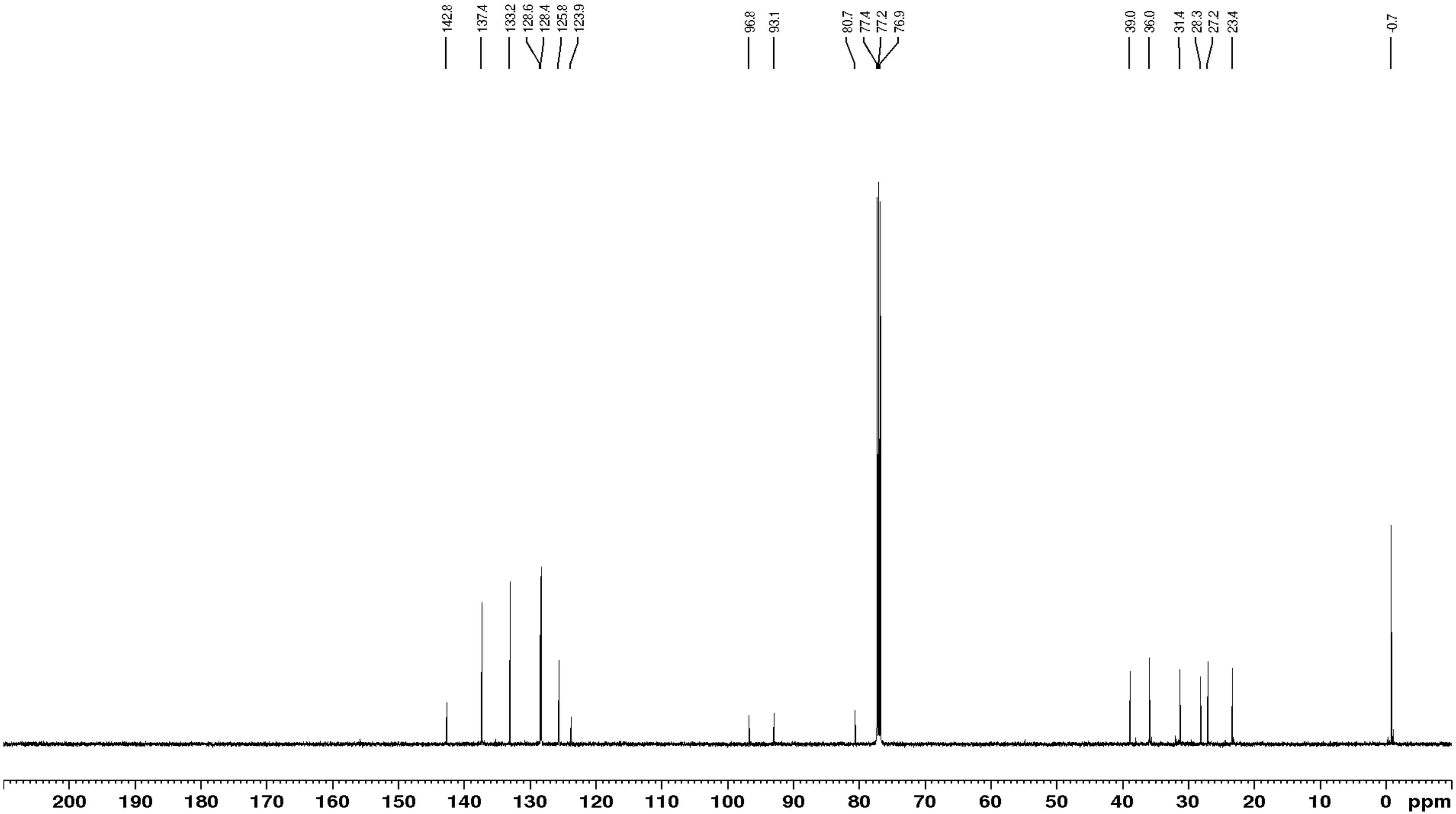

Figure S37. **$^{1}H/^{29}Si$ HMQC** NMR spectrum (500/99 MHz, $CDCl_3$, 298 K, optimized for J = 7 Hz) of **3al** from the reaction of alkene (**1a**) and alkynylsilane (**2l**).

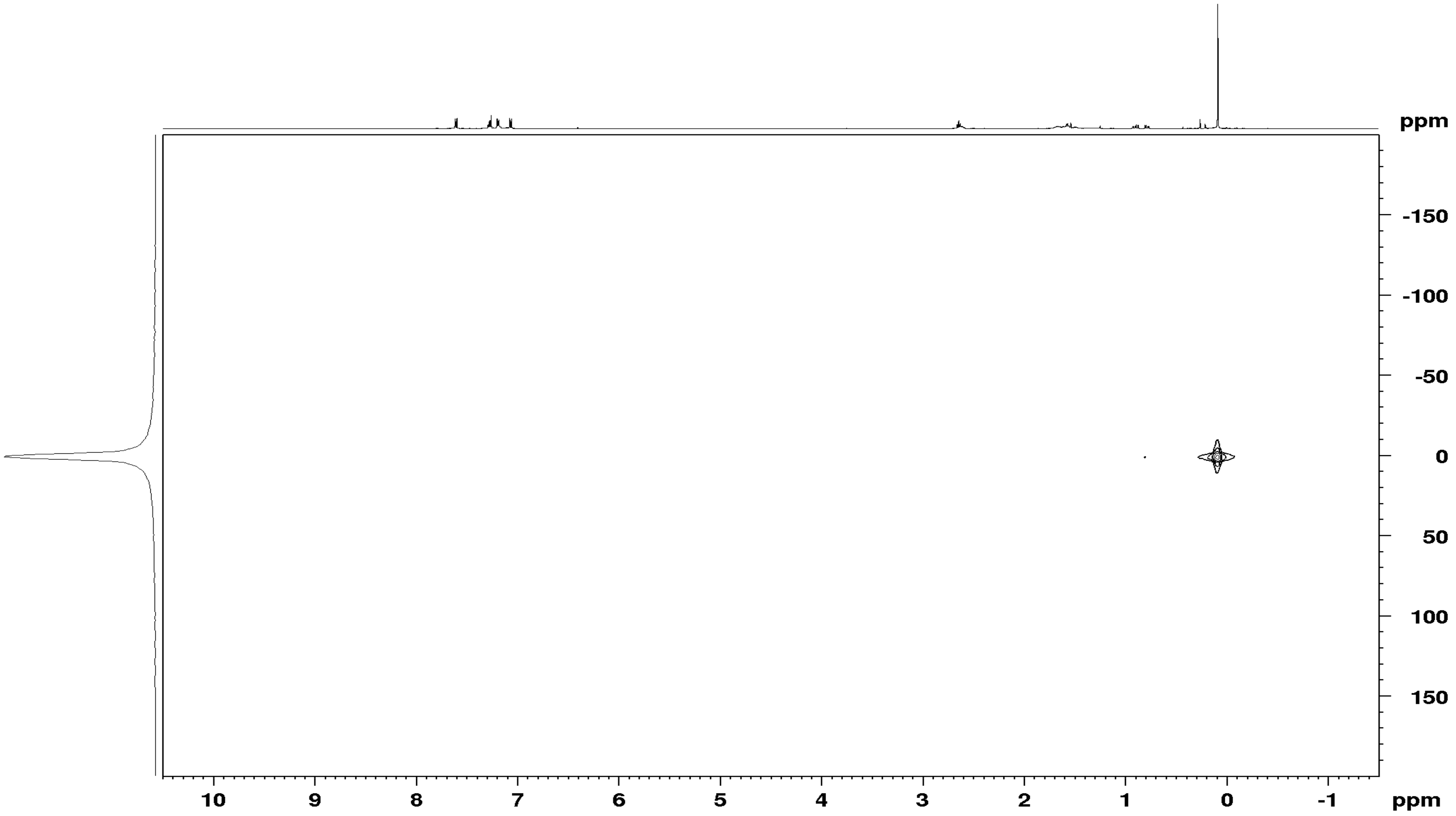

Figure S38. **^{1}H NMR** spectrum (500 MHz, $CDCl_3$, 298 K) of **3am** from the reaction of alkene (**1a**) and alkynylsilane (**2m**).

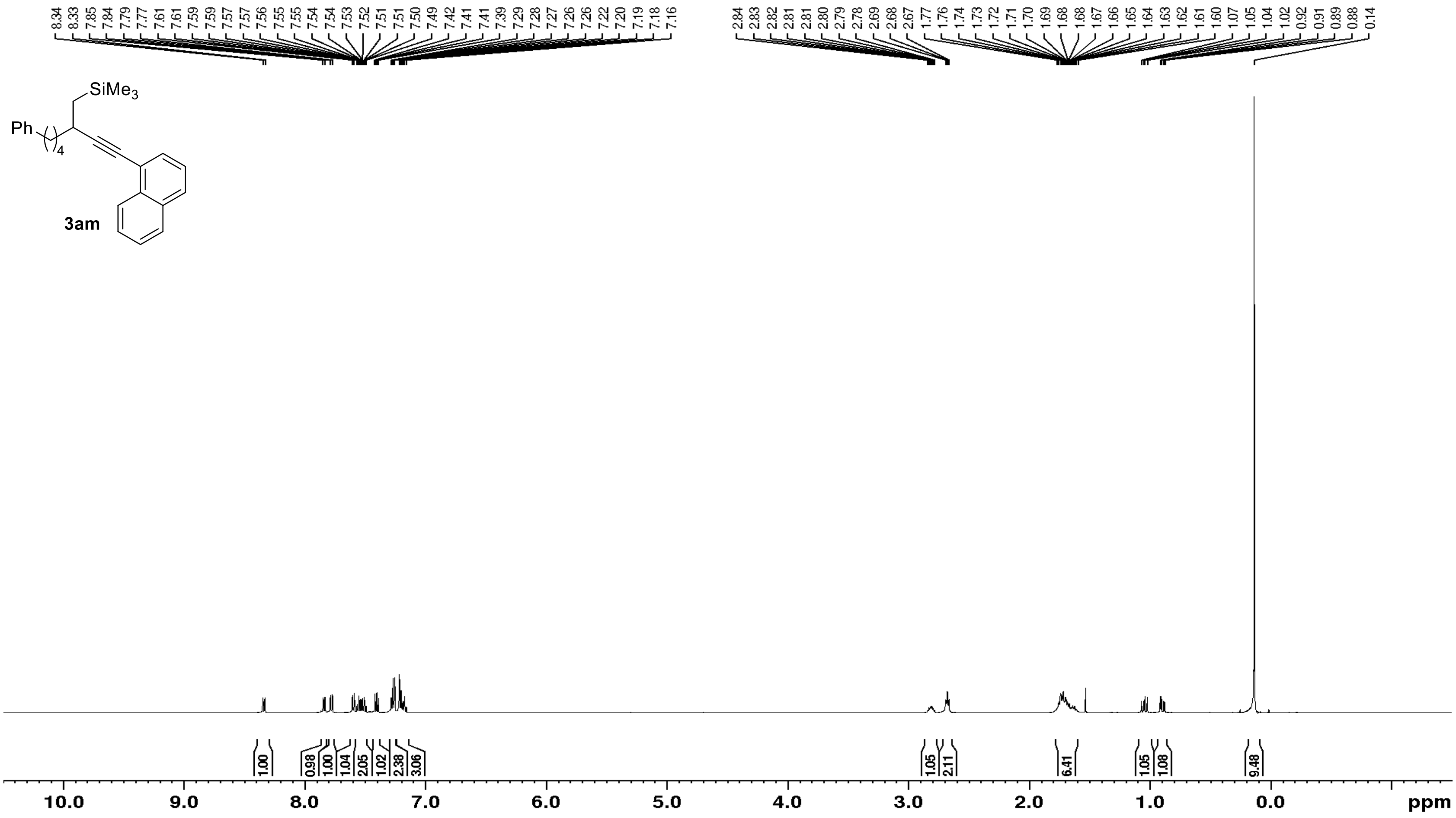

Figure S39. $^{13}C\{^{1}H\}$ **NMR** spectrum (126 MHz, $CDCl_3$, 298 K) of **3am** from the reaction of alkene (**1a**) and alkynylsilane (**2m**).

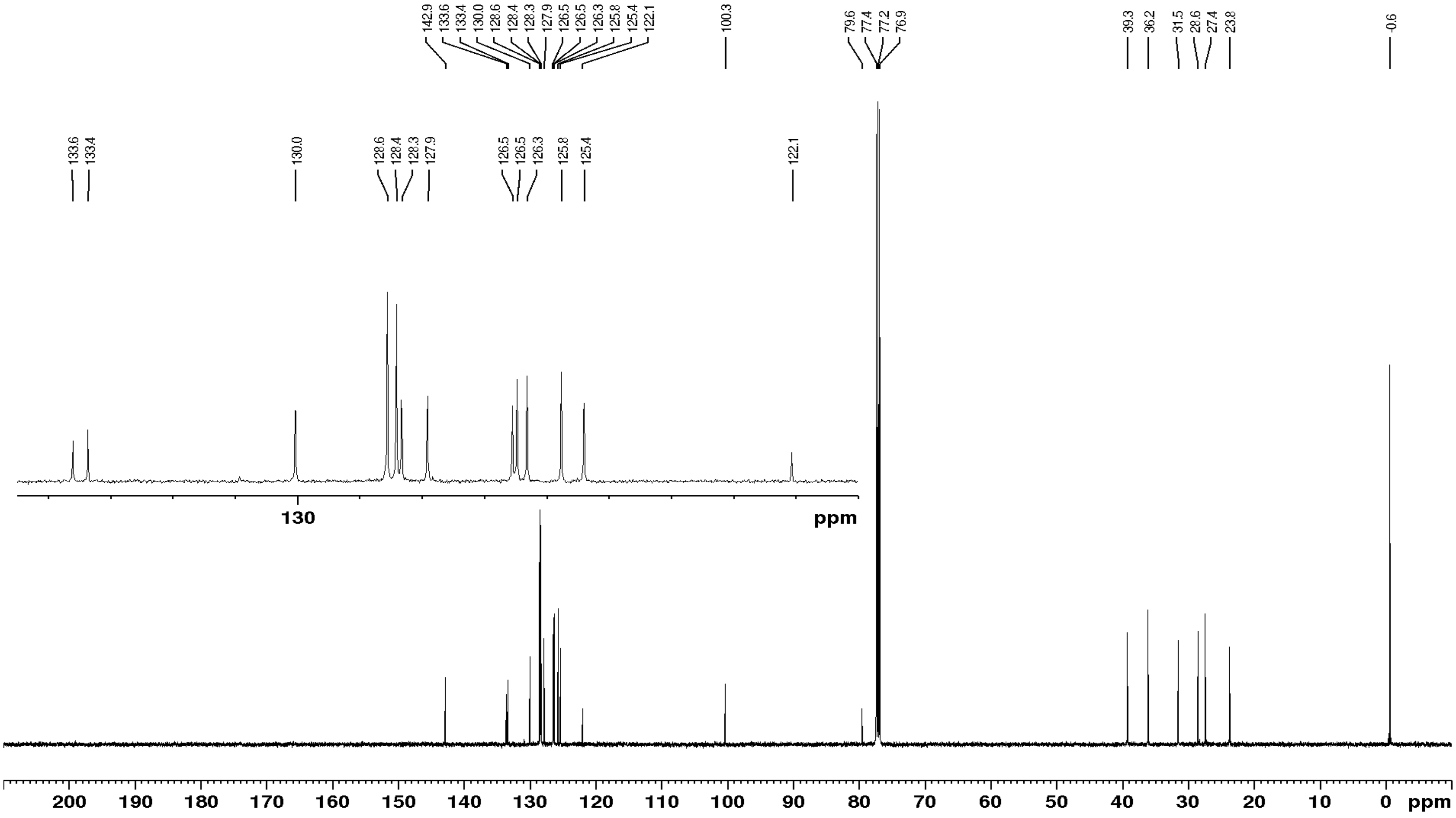

Figure S40. ^{1}**H/**^{29}**Si HMQC** NMR spectrum (500/99 MHz, $CDCl_3$, 298 K, optimized for *J* = 7 Hz) of **3am** from the reaction of alkene (**1a**) and alkynylsilane (**2m**).

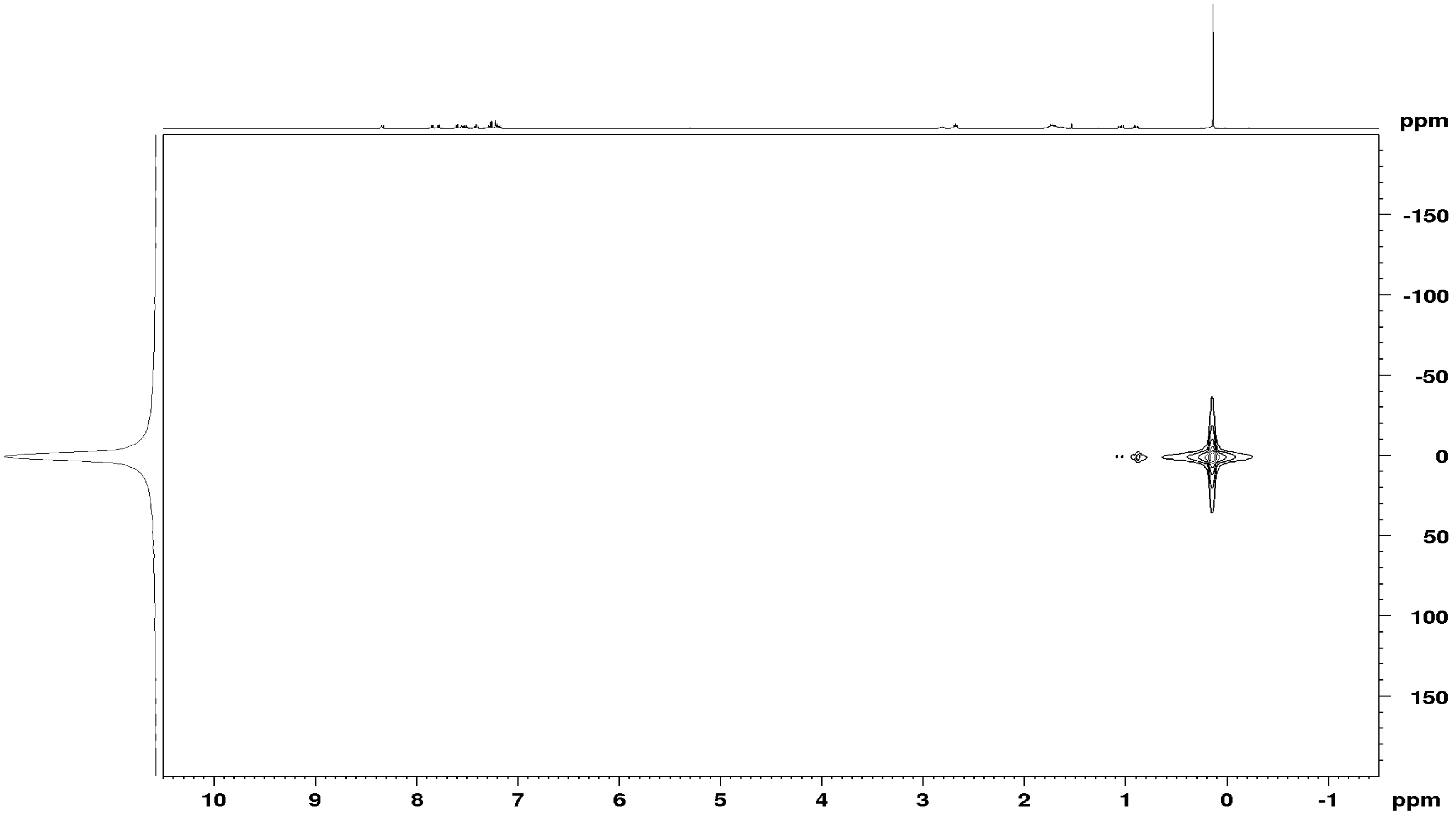

Figure S41. ^{1}**H NMR** spectrum (500 MHz, $CDCl_3$, 298 K) of **3an** from the reaction of alkene (**1a**) and alkynylsilane (**2n**).

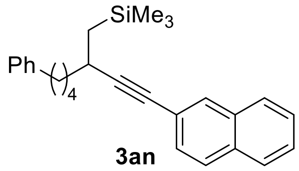

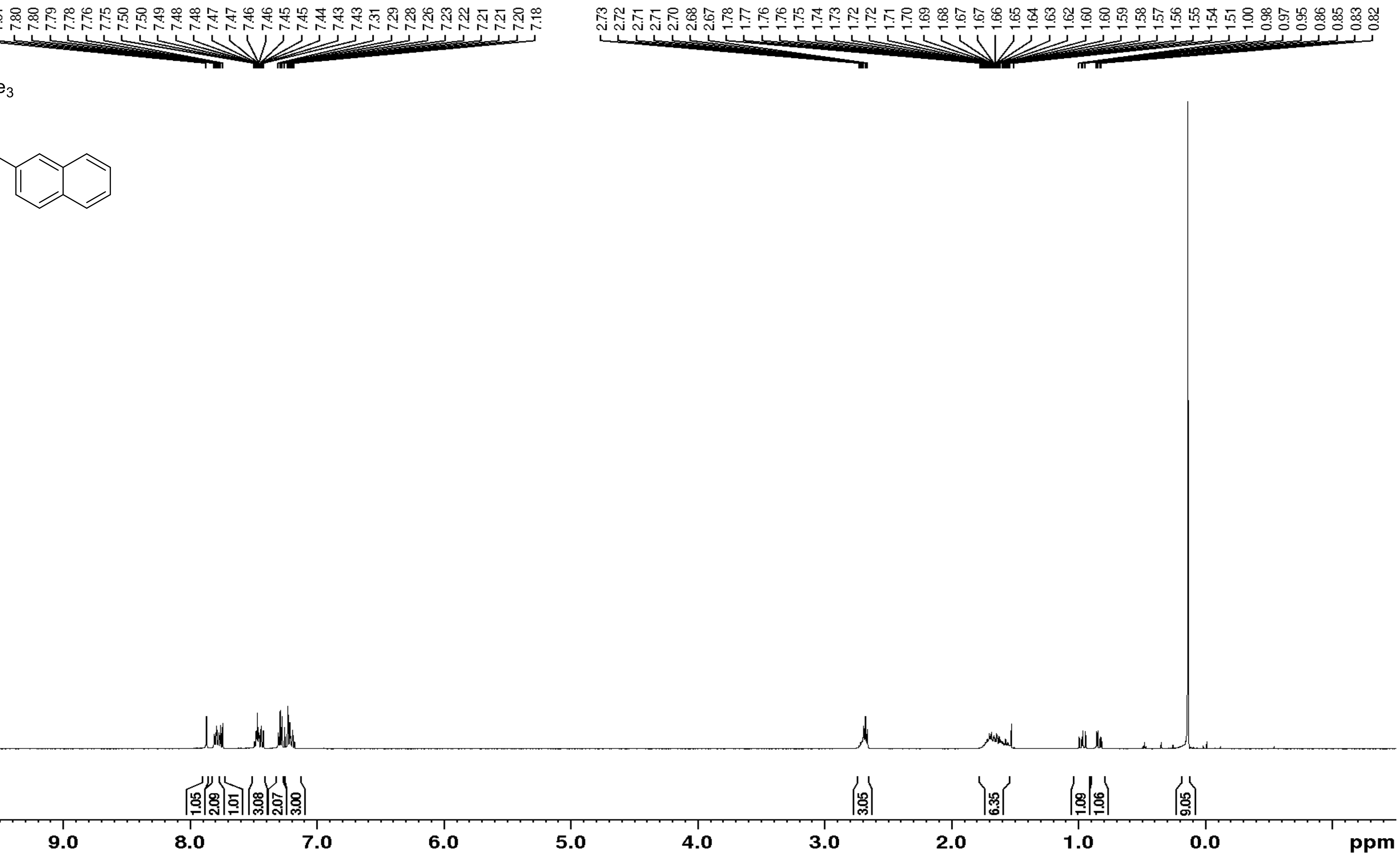

Figure S42. **$^{13}C\{^{1}H\}$ NMR** spectrum (126 MHz, $CDCl_3$, 298 K) of **3an** from the reaction of alkene (**1a**) and alkynylsilane (**2n**).

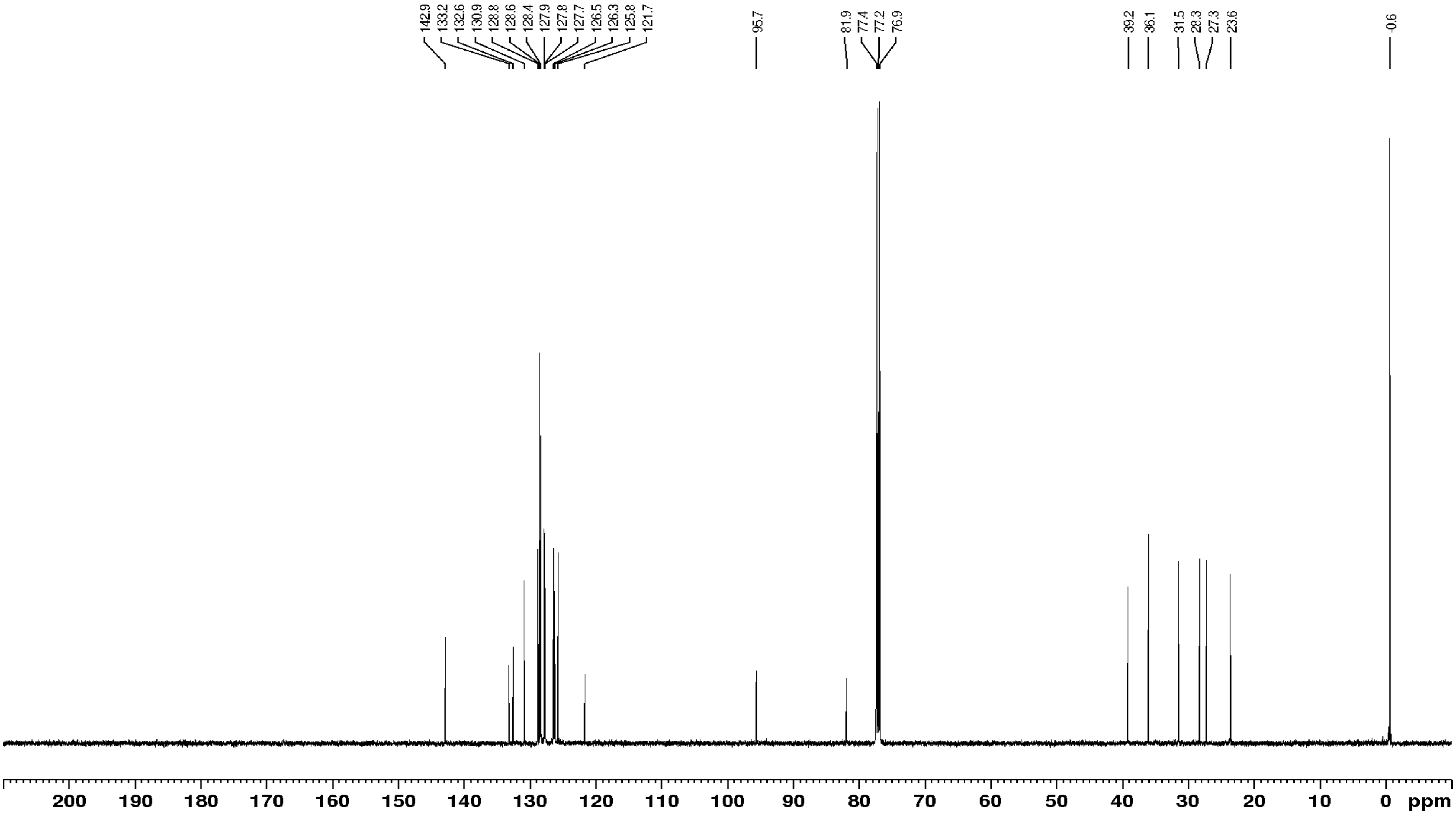

Figure S43. **$^1H/^{29}Si$ HMQC** NMR spectrum (500/99 MHz, $CDCl_3$, 298 K, optimized for J = 7 Hz) of **3an** from the reaction of alkene (**1a**) and alkynylsilane (**2n**).

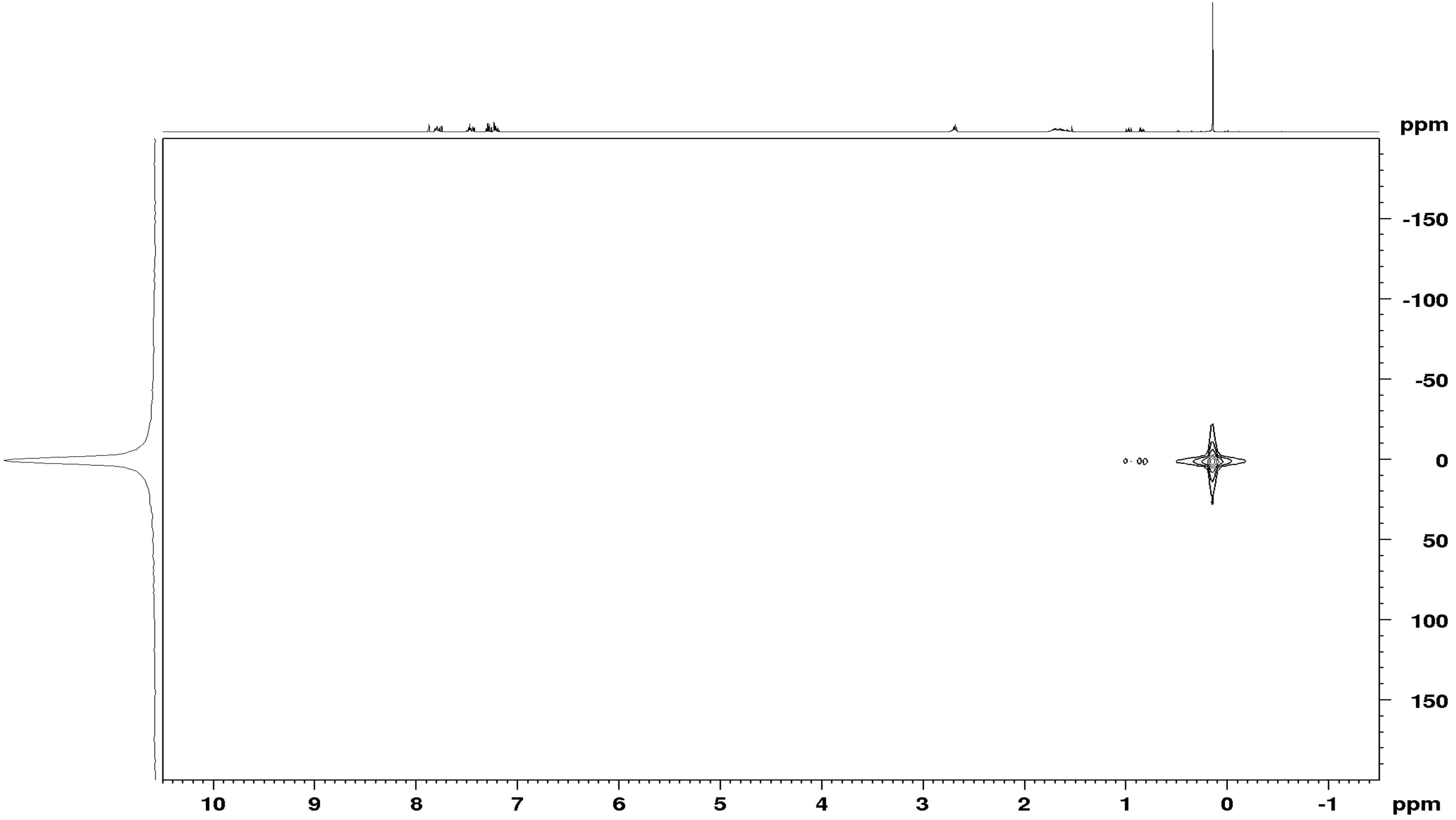

Figure S44. **^{1}H NMR** spectrum (500 MHz, $CDCl_3$, 298 K) of **3ba** from the reaction of alkene (**1b**) and alkynylsilane (**2a**).

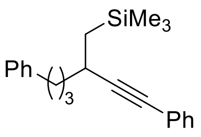

3ba

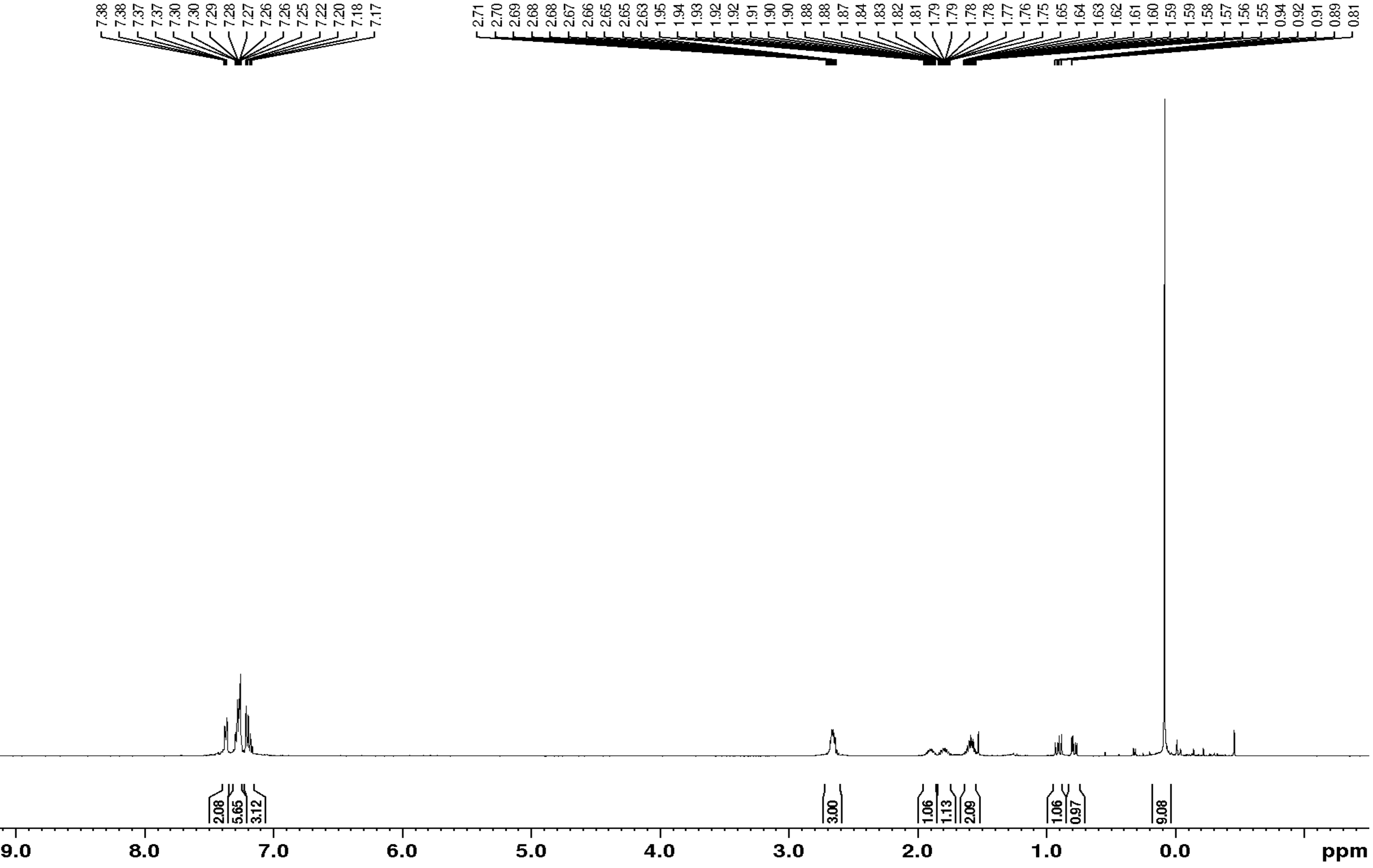

Figure S45. **$^{13}C\{^{1}H\}$ NMR** spectrum (126 MHz, $CDCl_3$, 298 K) of **3ba** from the reaction of alkene (**1b**) and alkynylsilane (**2a**).

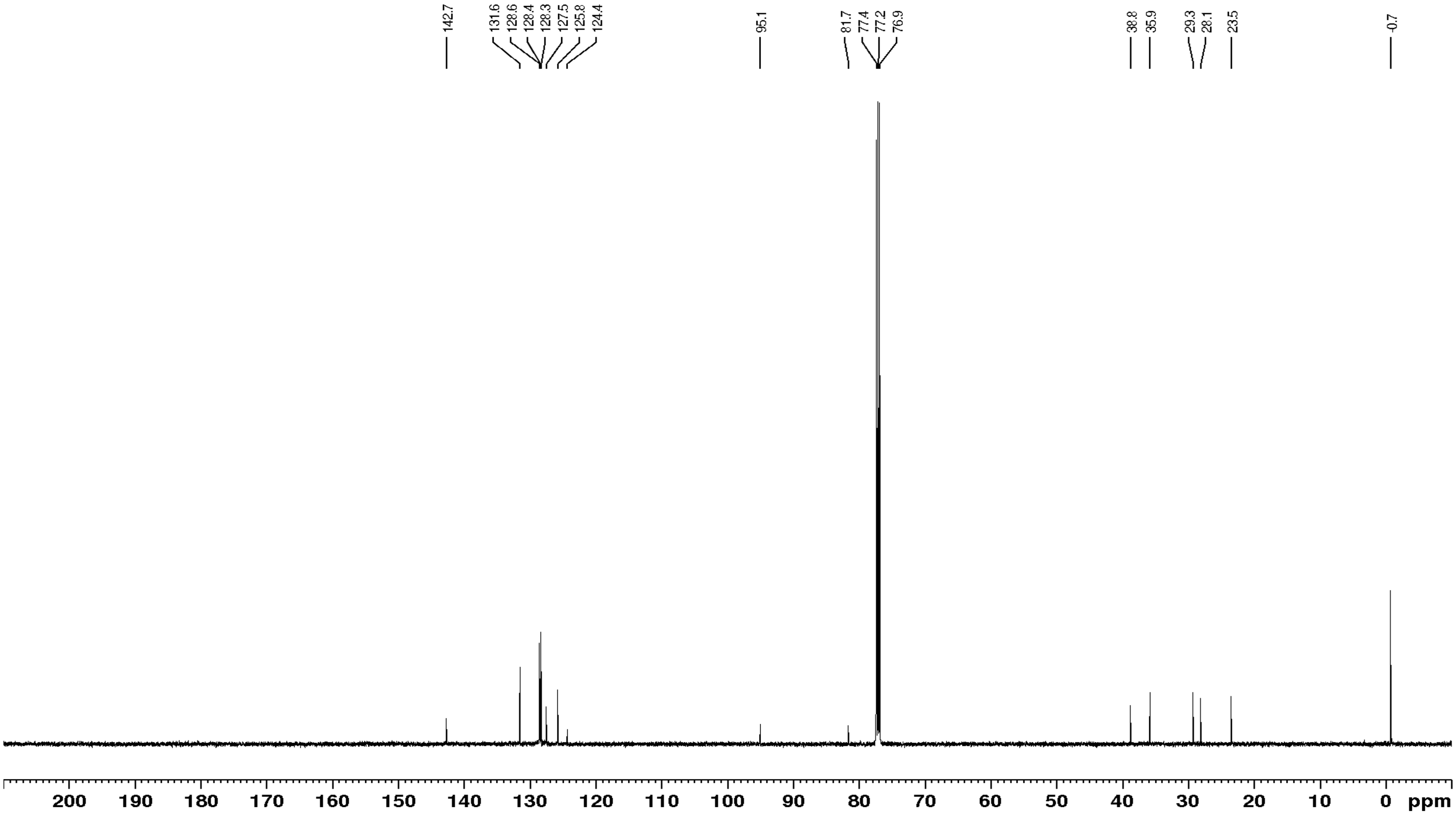

Figure S46. ^{1}**H/**^{29}**Si HMQC** NMR spectrum (500/99 MHz, $CDCl_3$, 298 K, optimized for J = 7 Hz) of **3ba** from the reaction of alkene (**1b**) and alkynylsilane (**2a**).

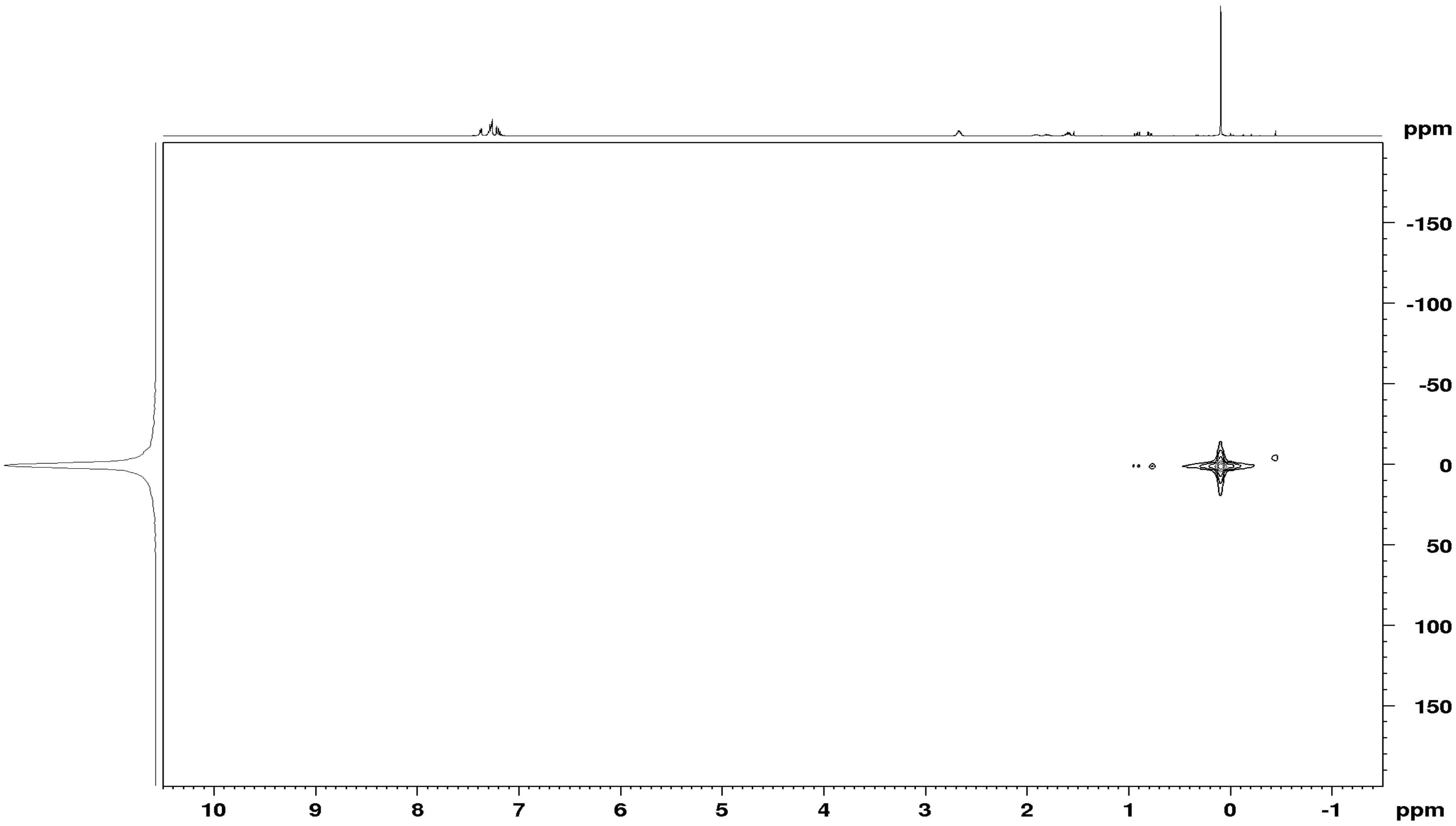

Figure S47. **^{1}H NMR** spectrum (500 MHz, $CDCl_3$, 298 K) of **3fa** from the reaction of alkene (**1f**) and alkynylsilane (**2a**).

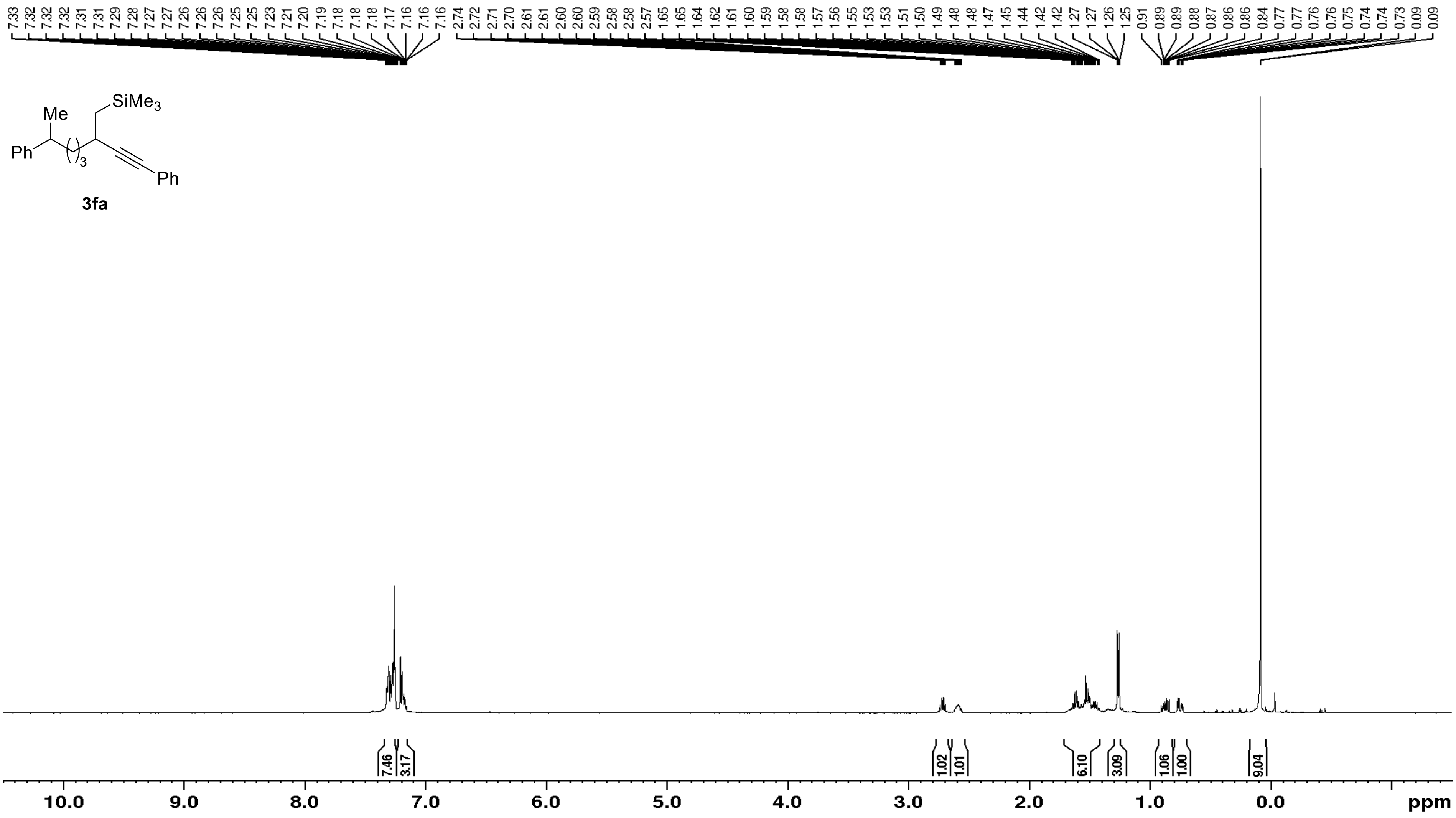

Figure S48. **$^{13}C\{^{1}H\}$ NMR** spectrum (126 MHz, $CDCl_3$, 298 K) of **3fa** from the reaction of alkene (**1f**) and alkynylsilane (**2a**).

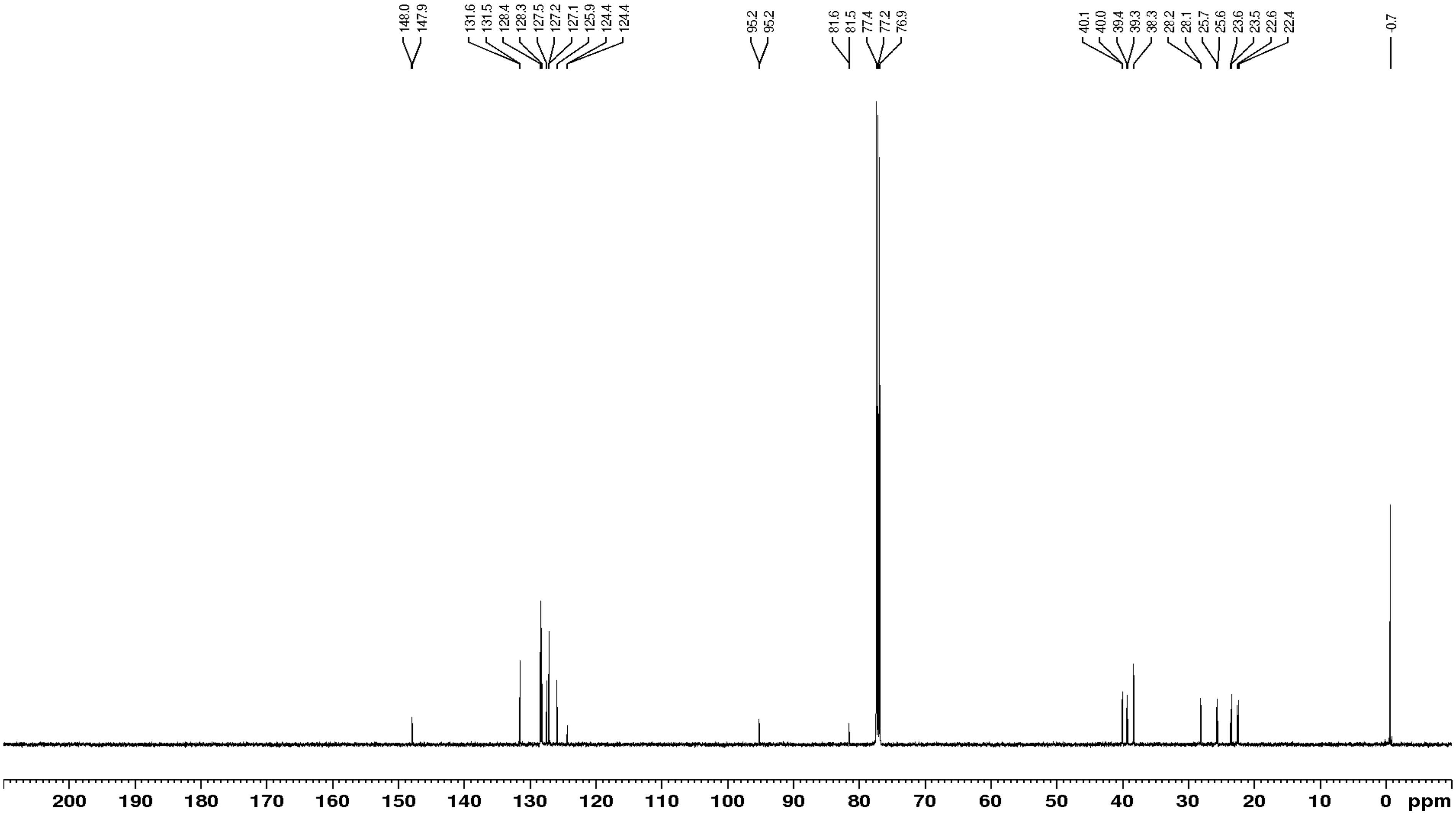

Figure S49. **$^{1}H/^{29}Si$ HMQC** NMR spectrum (500/99 MHz, $CDCl_3$, 298 K, optimized for *J* = 7 Hz) of **3fa** from the reaction of alkene (**1f**) and alkynylsilane (**2a**).

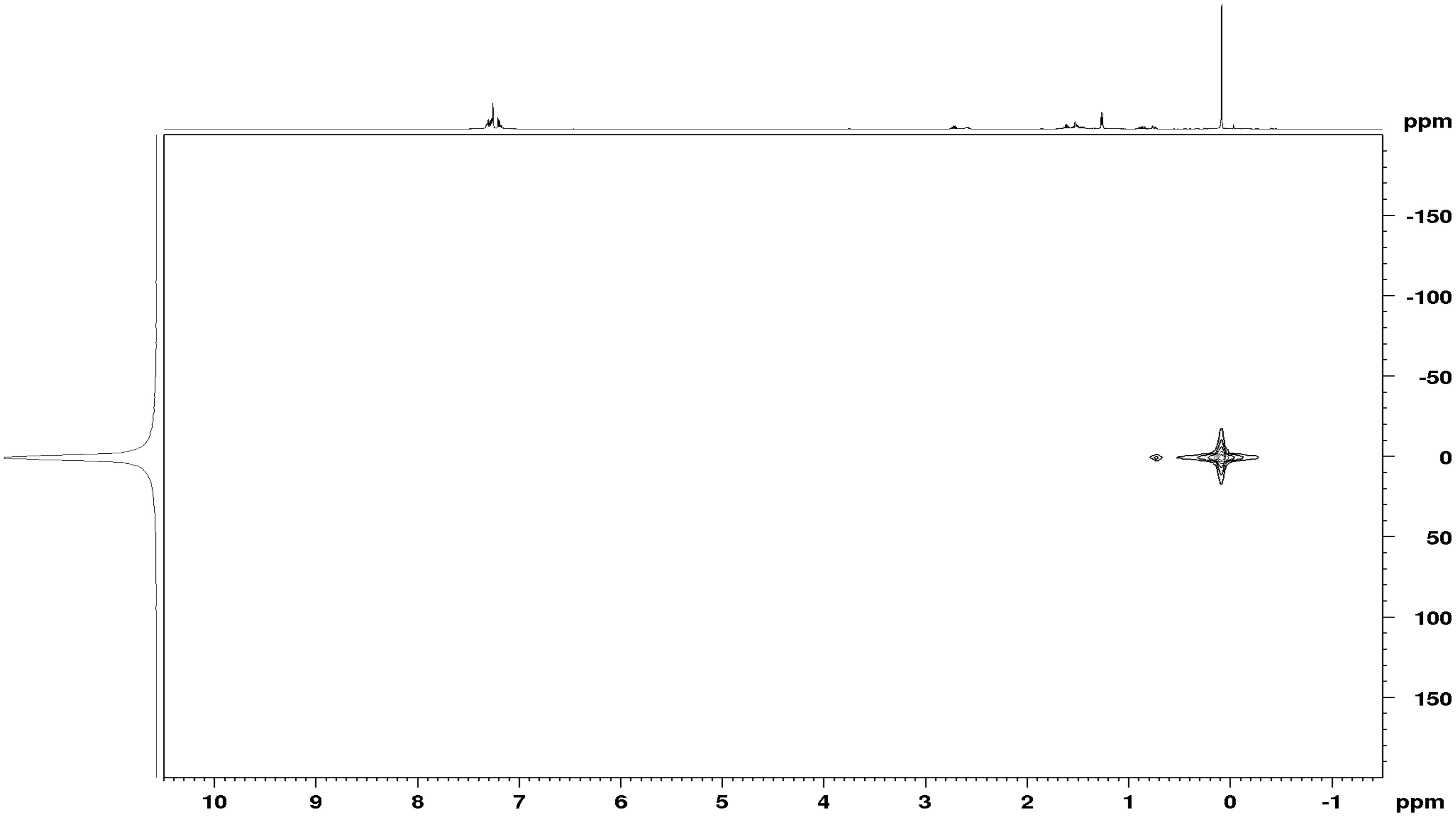

Figure S50. **^{1}H NMR** spectrum (500 MHz, $CDCl_3$, 298 K) of **3ga** from the reaction of alkene (**1g**) and alkynylsilane (**2a**).

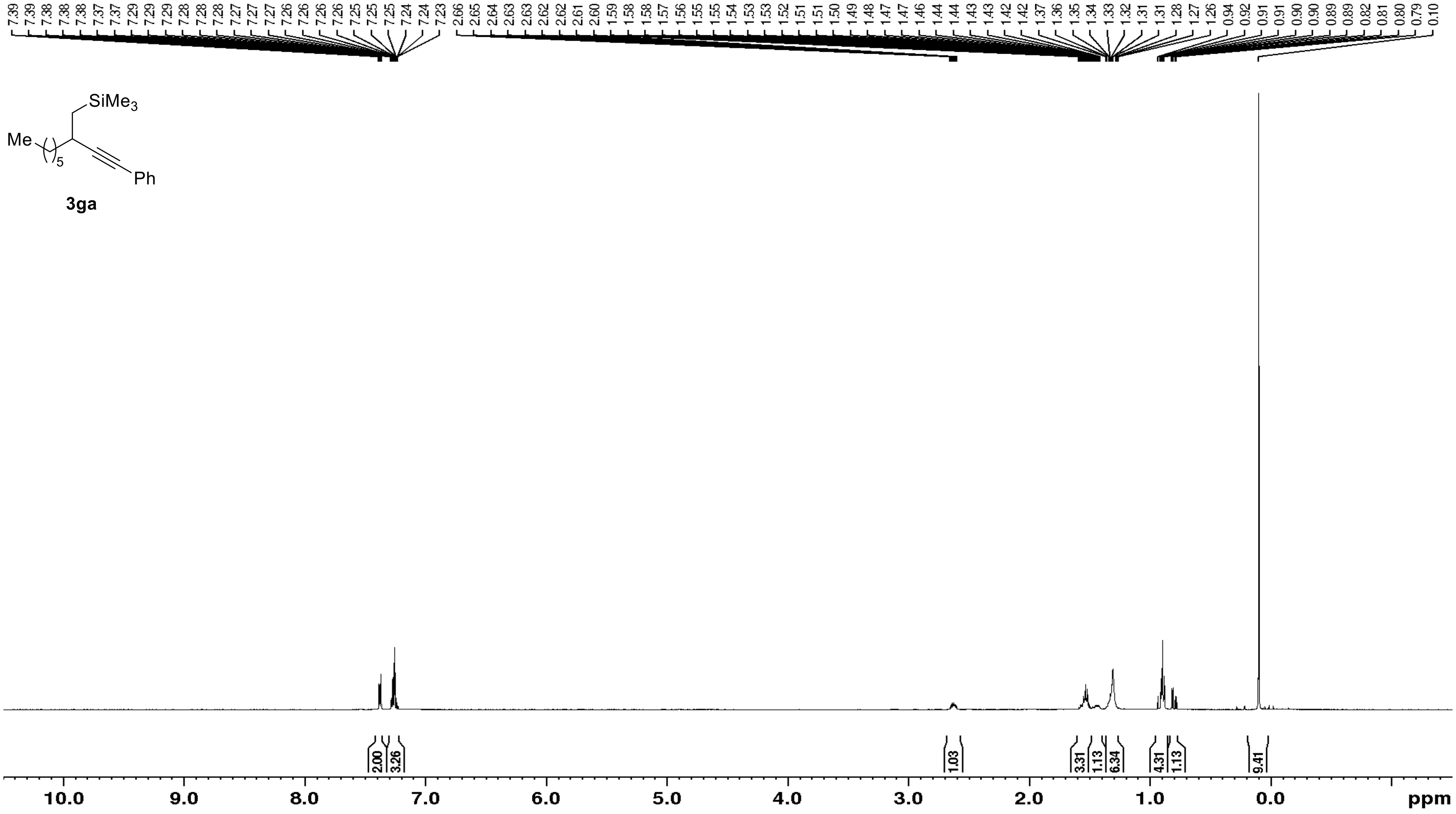

Figure S51. **$^{13}C\{^1H\}$ NMR** spectrum (126 MHz, $CDCl_3$, 298 K) of **3ga** from the reaction of alkene (**1g**) and alkynylsilane (**2a**).

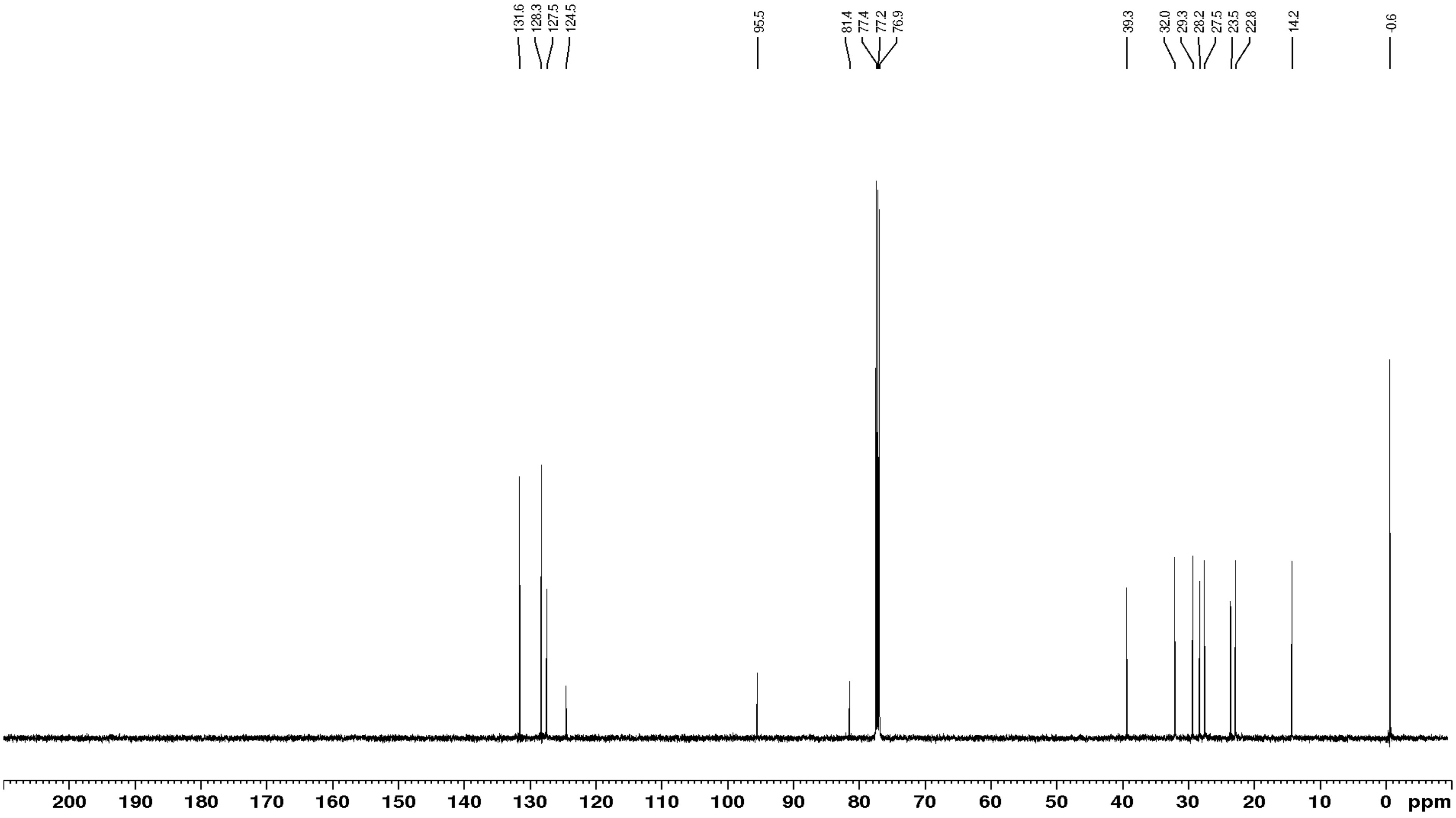

Figure S52. **$^{1}H/^{29}Si$ HMQC** NMR spectrum (500/99 MHz, $CDCl_3$, 298 K, optimized for *J* = 7 Hz) of **3ga** from the reaction of alkene (**1g**) and alkynylsilane (**2a**).

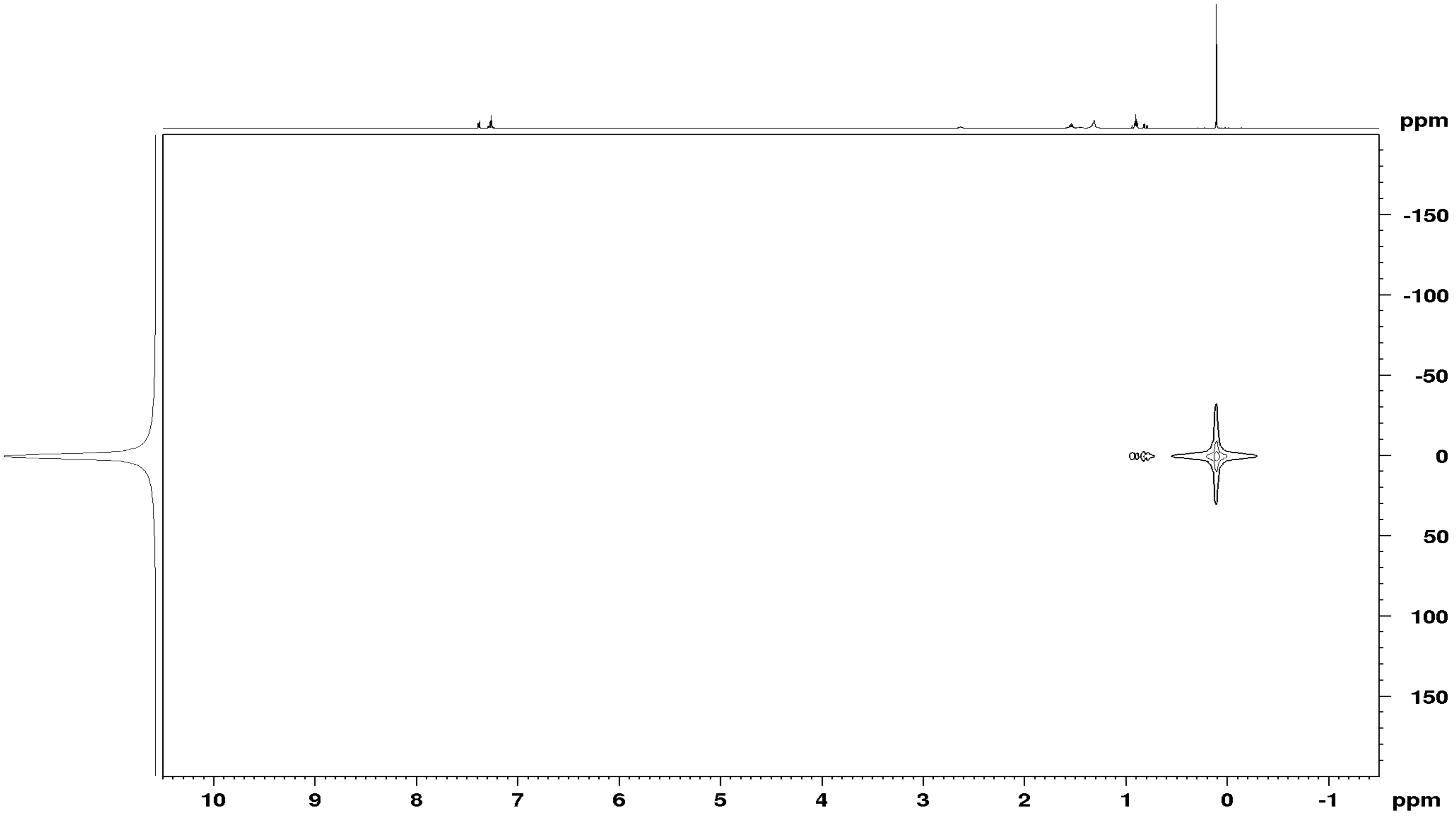

Figure S53. **^{1}H NMR** spectrum (500 MHz, $CDCl_3$, 298 K) of **3ha** from the reaction of alkene (**1h**) and alkynylsilane (**2a**).

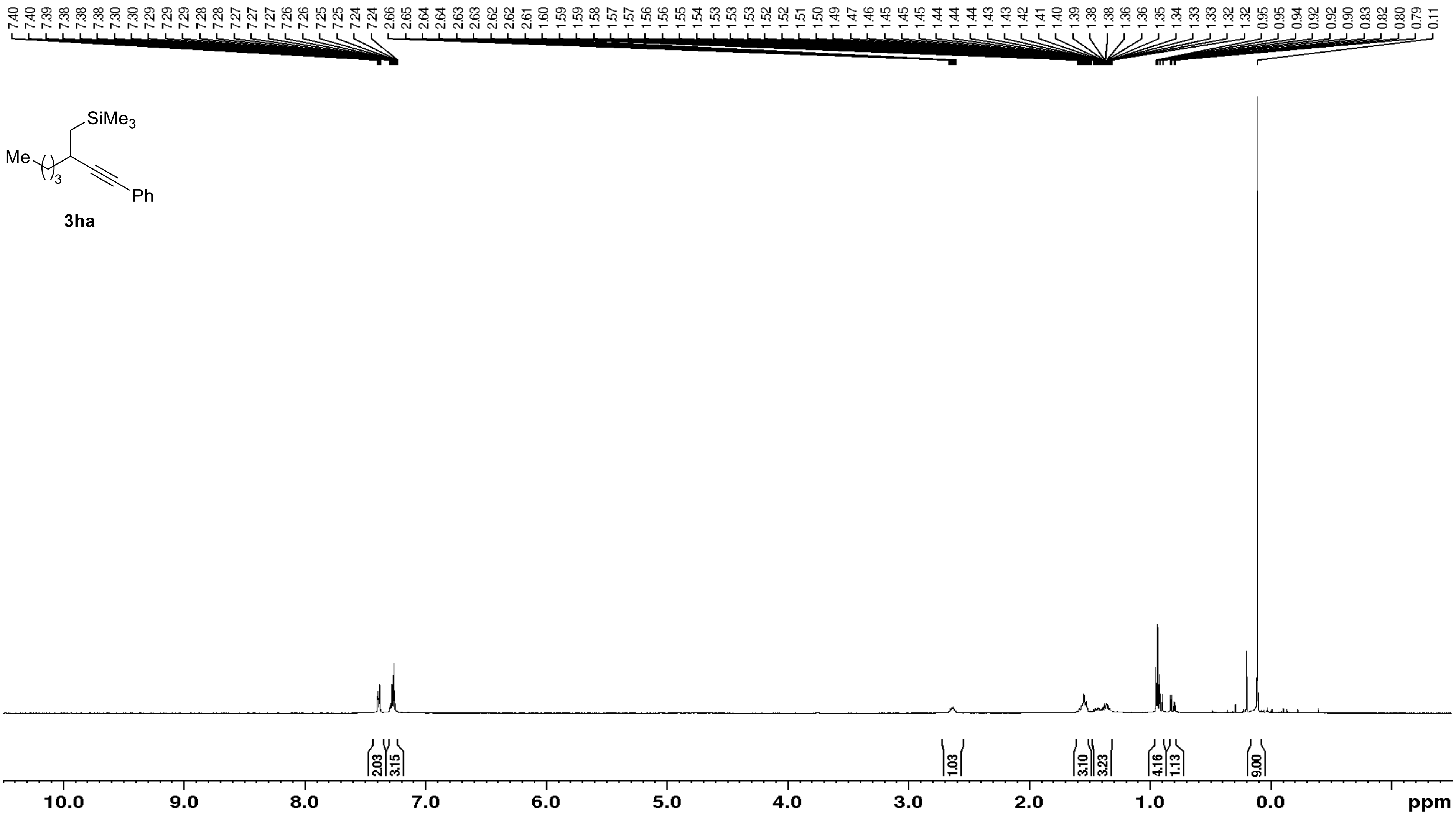

Figure S54. **$^{13}C\{^1H\}$ NMR** spectrum (126 MHz, $CDCl_3$, 298 K) of **3ha** from the reaction of alkene (**1h**) and alkynylsilane (**2a**).

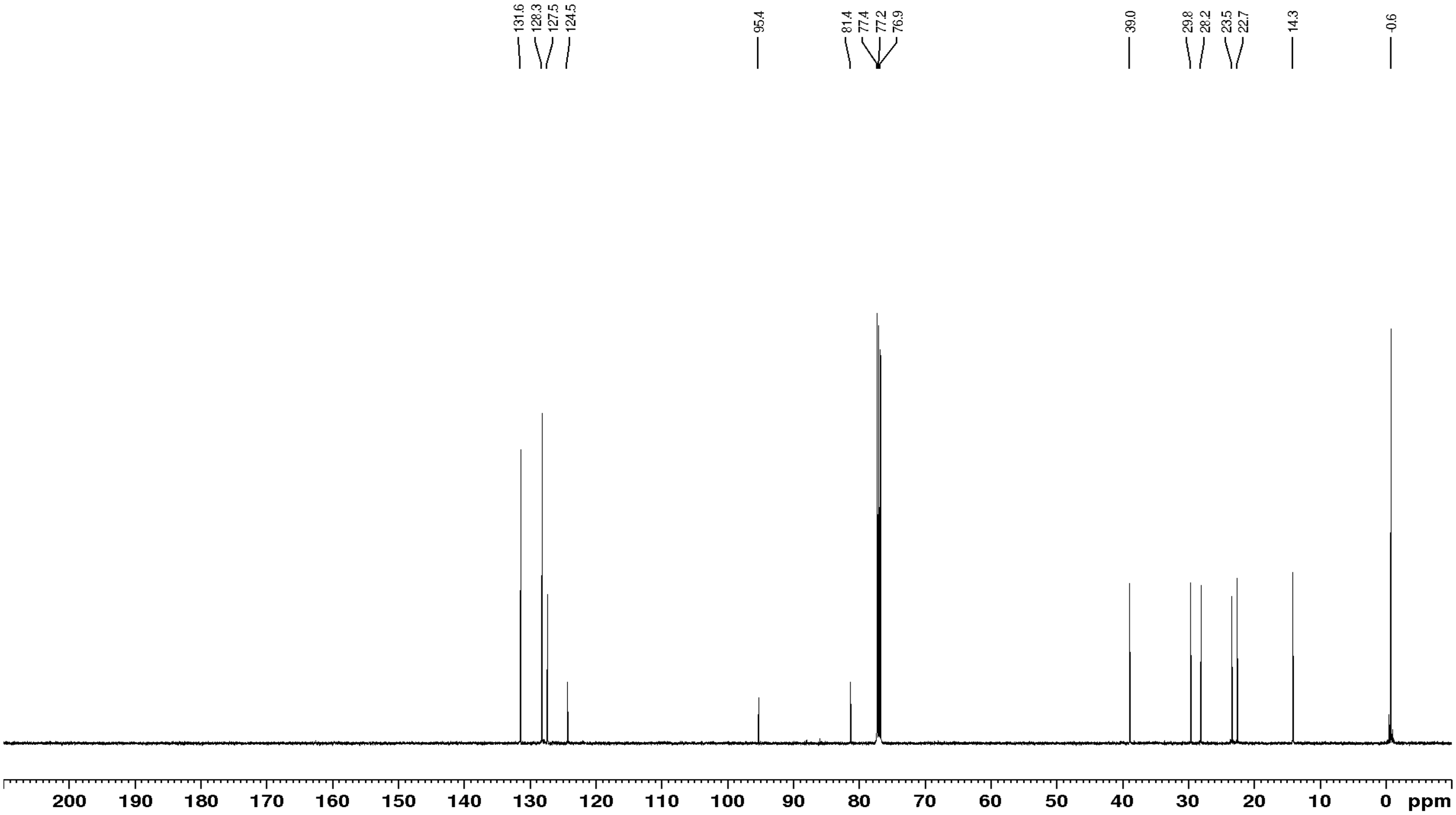

Figure S55. **$^{1}H/^{29}Si$ HMQC** NMR spectrum (500/99 MHz, $CDCl_3$, 298 K, optimized for *J* = 7 Hz) of **3ha** from the reaction of alkene (**1h**) and alkynylsilane (**2a**).

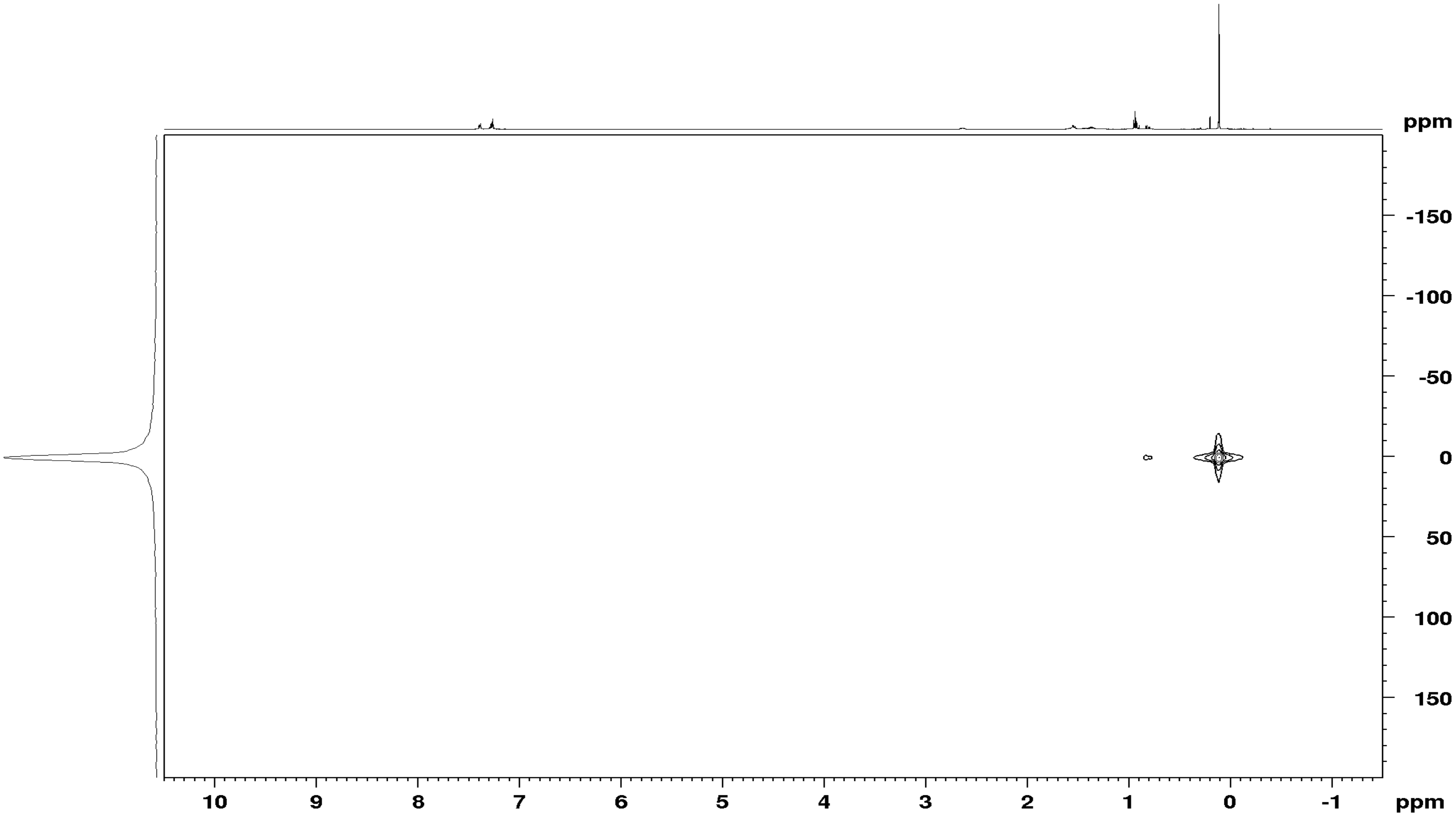

Figure S56. **^{1}H NMR** spectrum (500 MHz, $CDCl_3$, 298 K) of **3ao** from the reaction of alkene (**1a**) and alkynylsilane (**2o**).

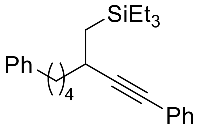

3ao

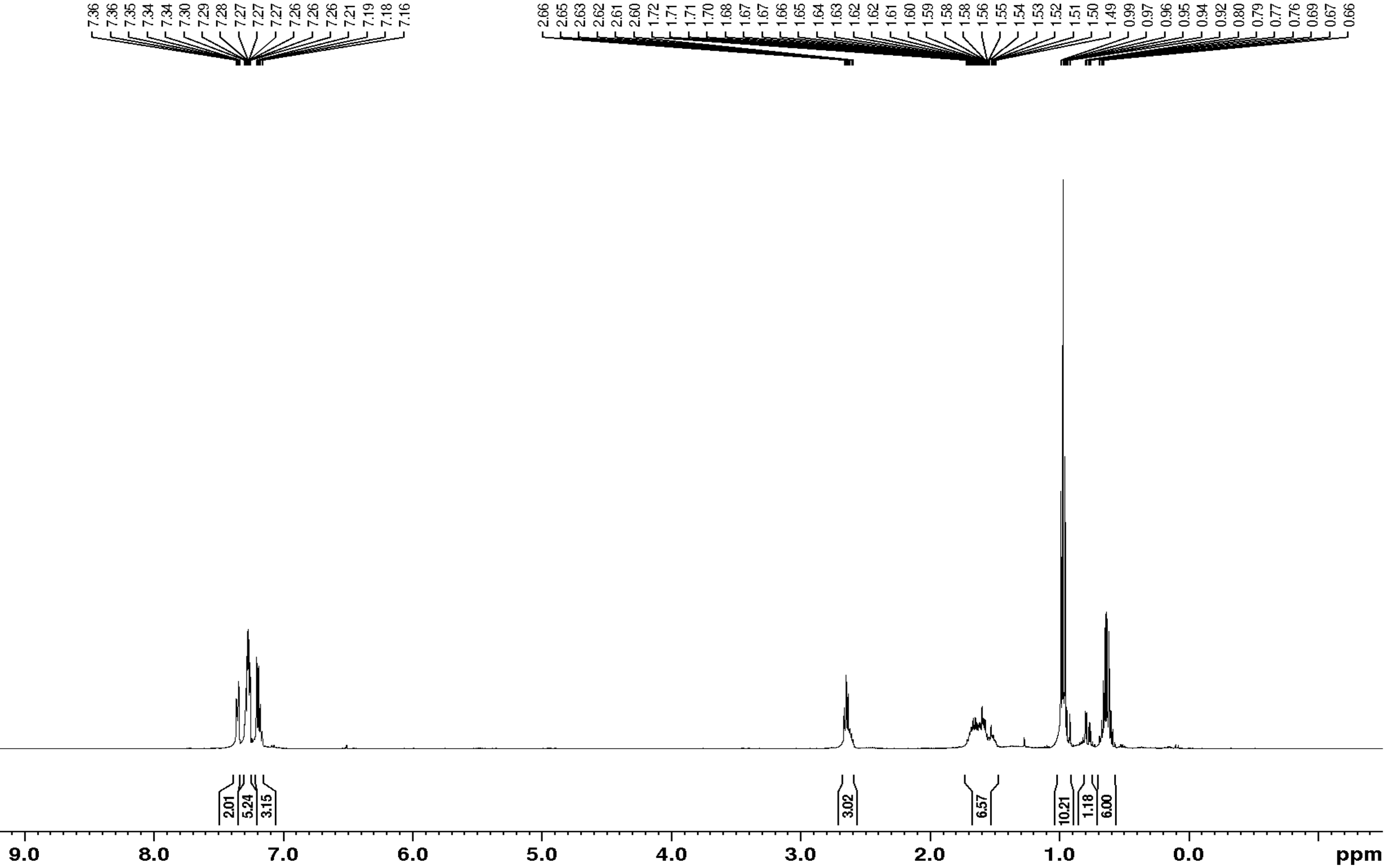

Figure S57. **$^{13}C\{^1H\}$ NMR** spectrum (126 MHz, $CDCl_3$, 298 K) of **3ao** from the reaction of alkene (**1a**) and alkynylsilane (**2o**).

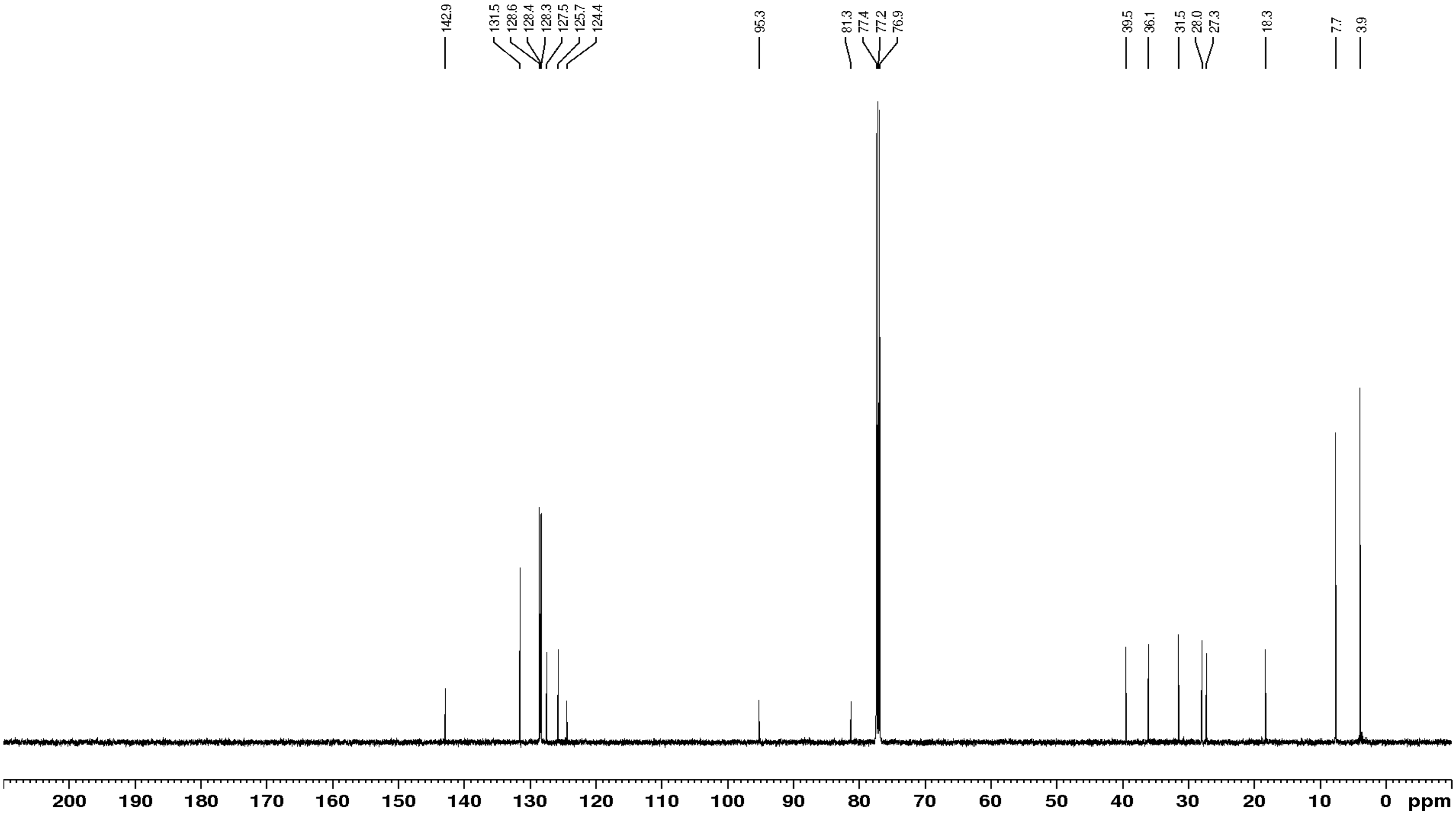

Figure S58. **$^{1}H/^{29}Si$ HMQC** NMR spectrum (500/99 MHz, $CDCl_3$, 298 K, optimized for J = 7 Hz) of **3ao** from the reaction of alkene (**1a**) and alkynylsilane (**2o**).

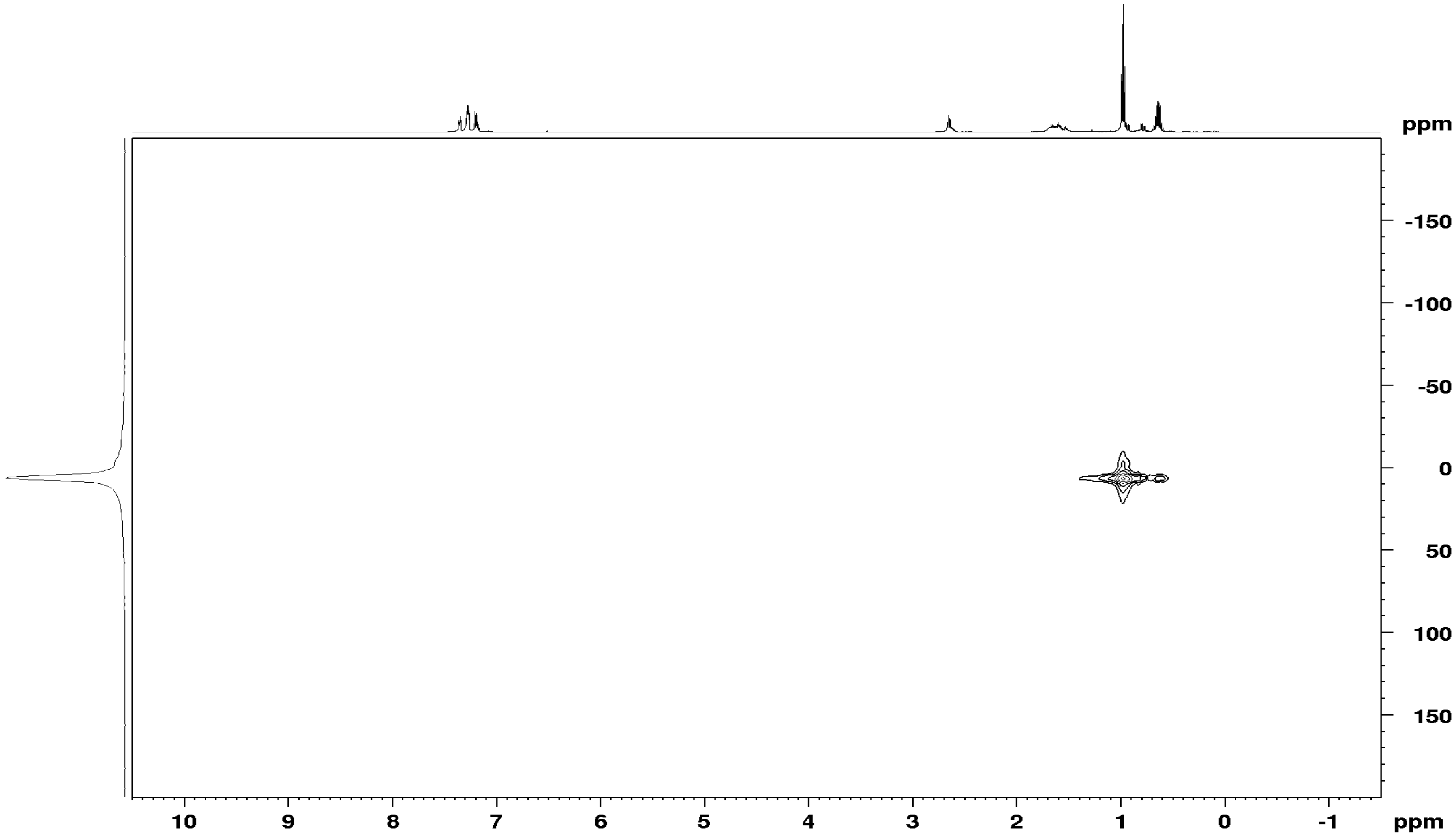

Figure S59. **^{1}H NMR** spectrum (500 MHz, $CDCl_3$, 298 K) of **3ap** from the reaction of alkene (**1a**) and alkynylsilane (**2p**).

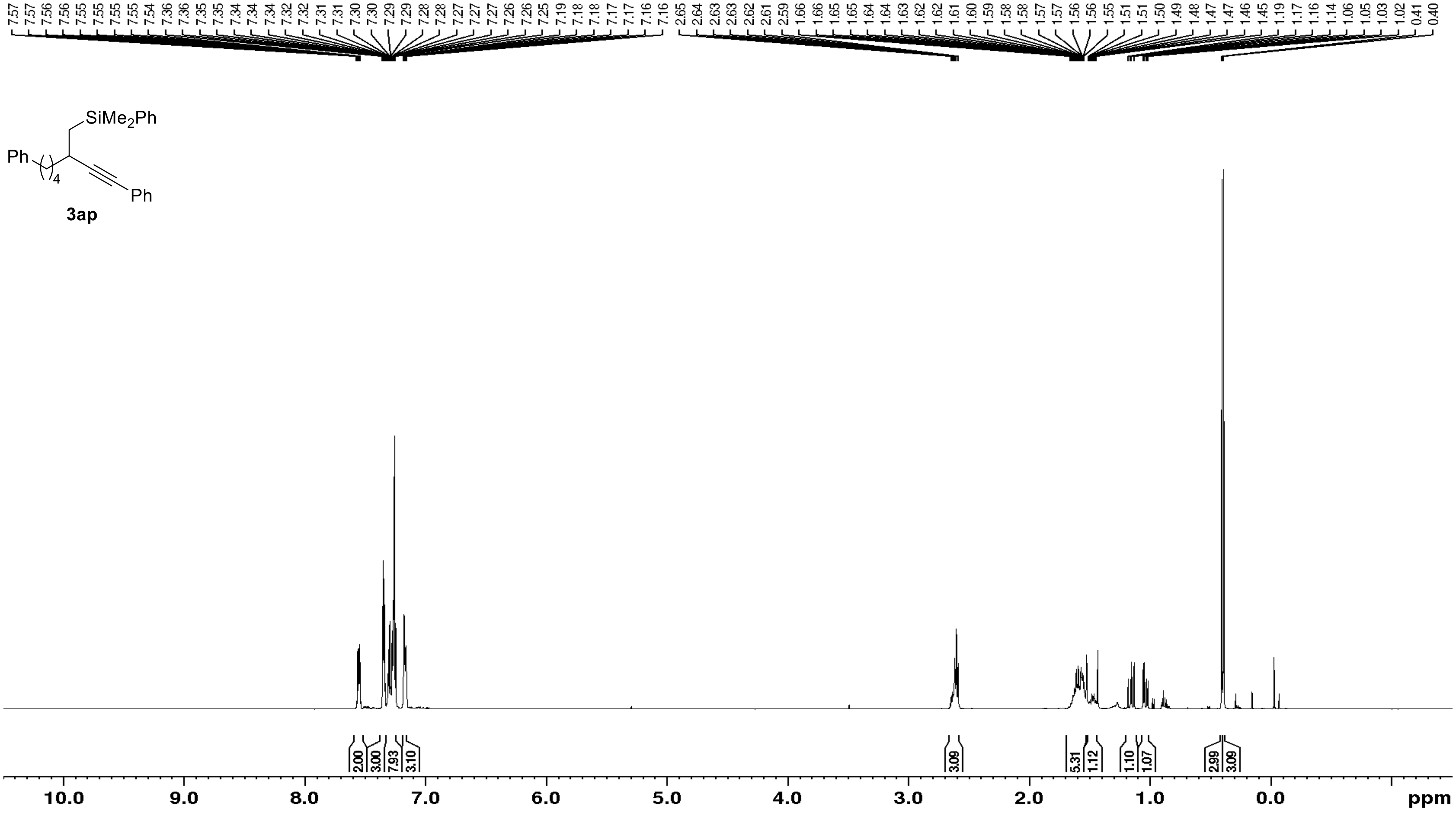

Figure S60. **$^{13}C\{^1H\}$ NMR** spectrum (126 MHz, $CDCl_3$, 298 K) of **3ap** from the reaction of alkene (**1a**) and alkynylsilane (**2p**).

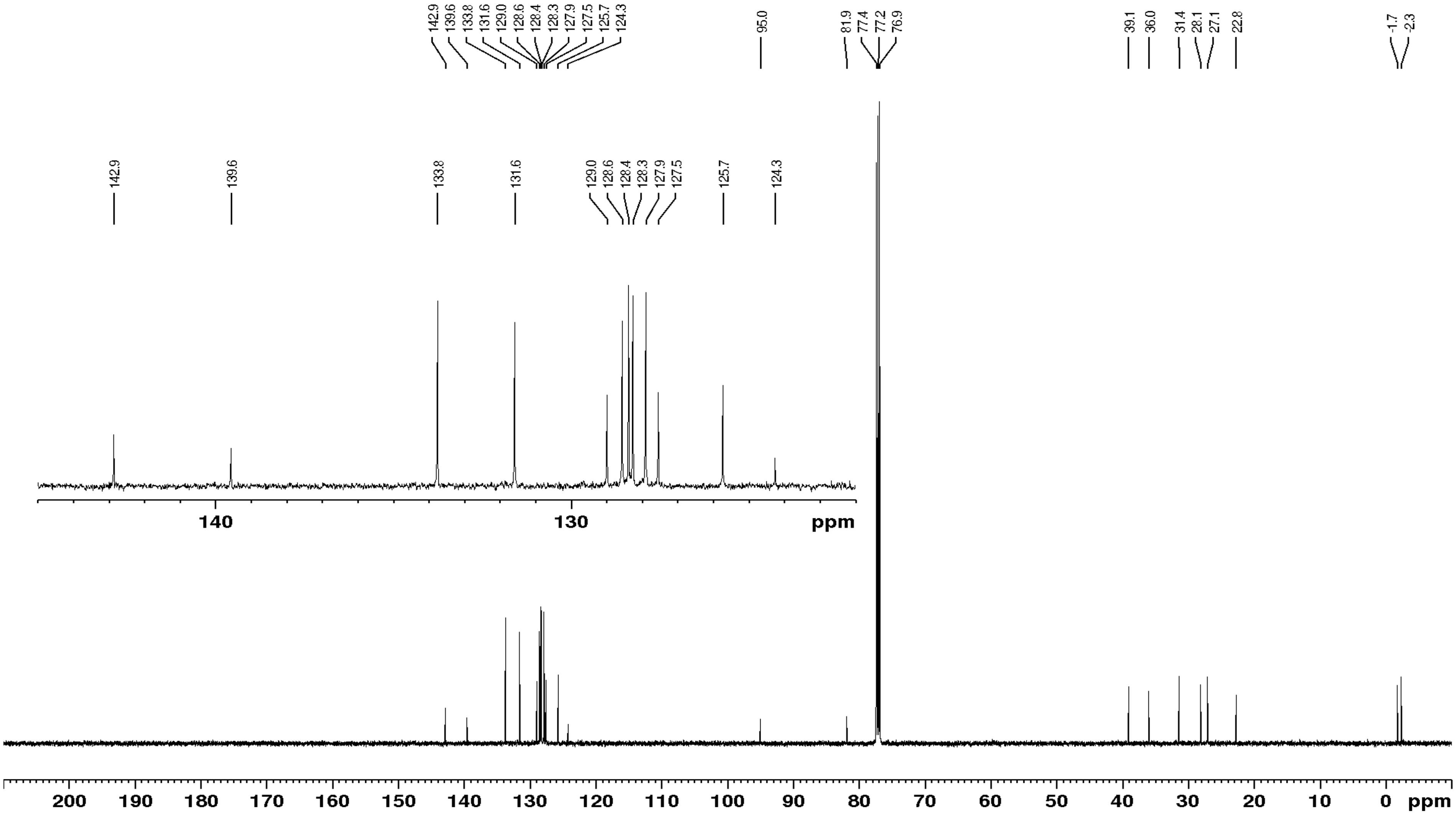

Figure S61. **$^{1}H/^{29}Si$ HMQC** NMR spectrum (500/99 MHz, $CDCl_3$, 298 K, optimized for J = 7 Hz) of **3ap** from the reaction of alkene (**1a**) and alkynylsilane (**2p**).

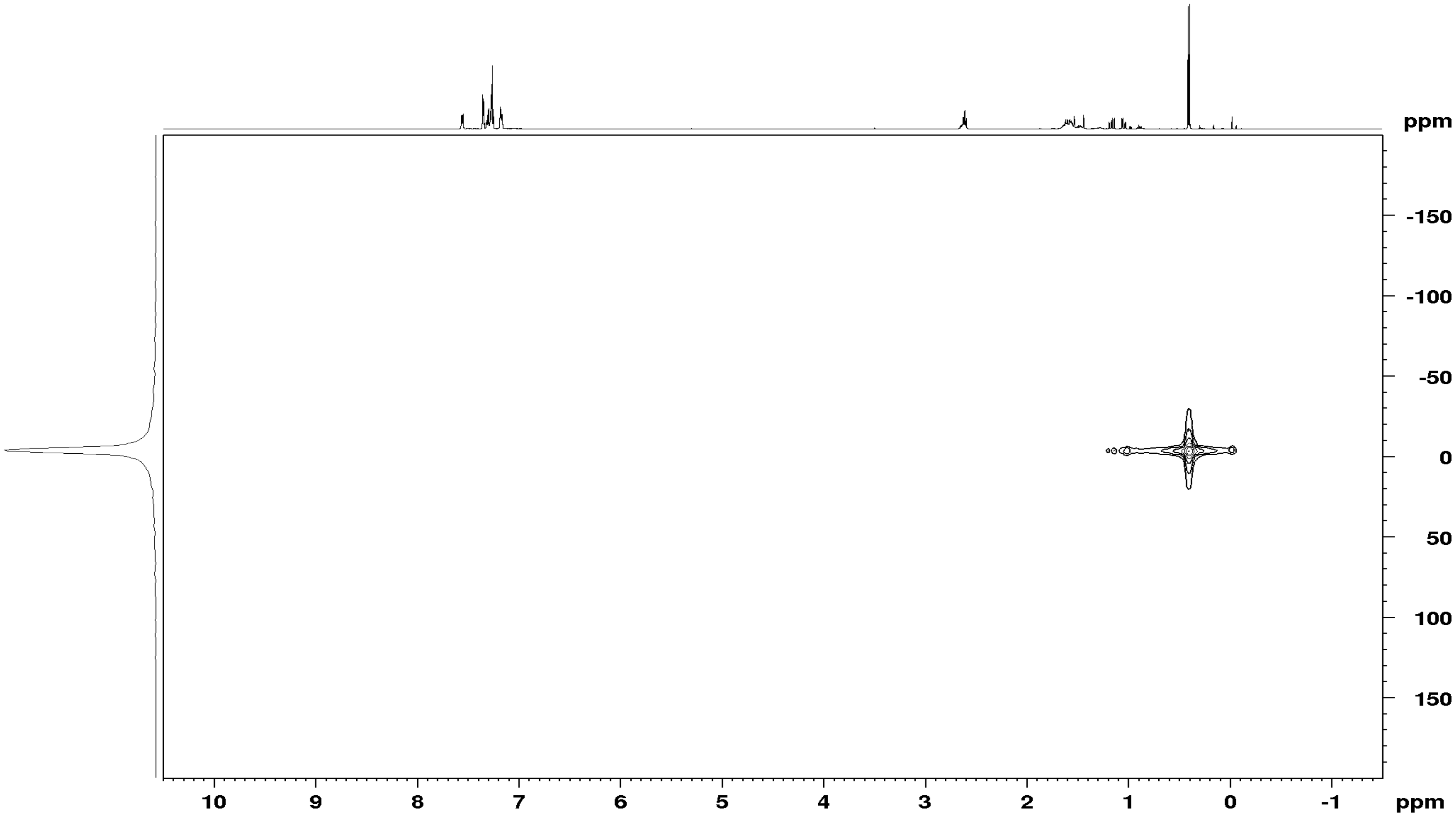

Figure S62. [1]**H NMR** spectrum (500 MHz, $CDCl_3$, 298 K) of **4ba** from the reaction of alkene (**1b**) and alkynylsilane (**2a**).

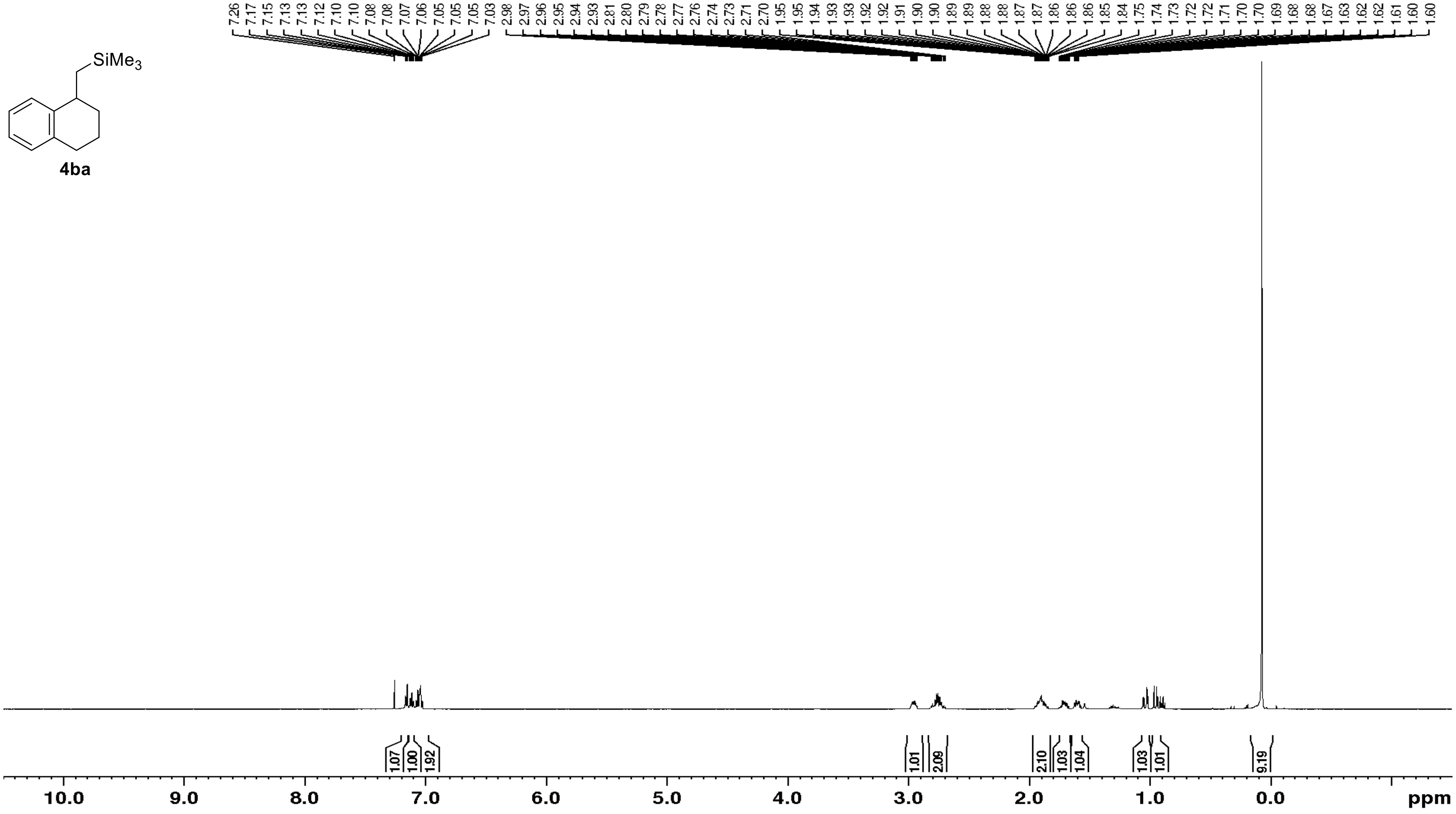

Figure S63. **$^{13}C\{^{1}H\}$ NMR** spectrum (126 MHz, $CDCl_3$, 298 K) of **4ba** from the reaction of alkene (**1b**) and alkynylsilane (**2a**).

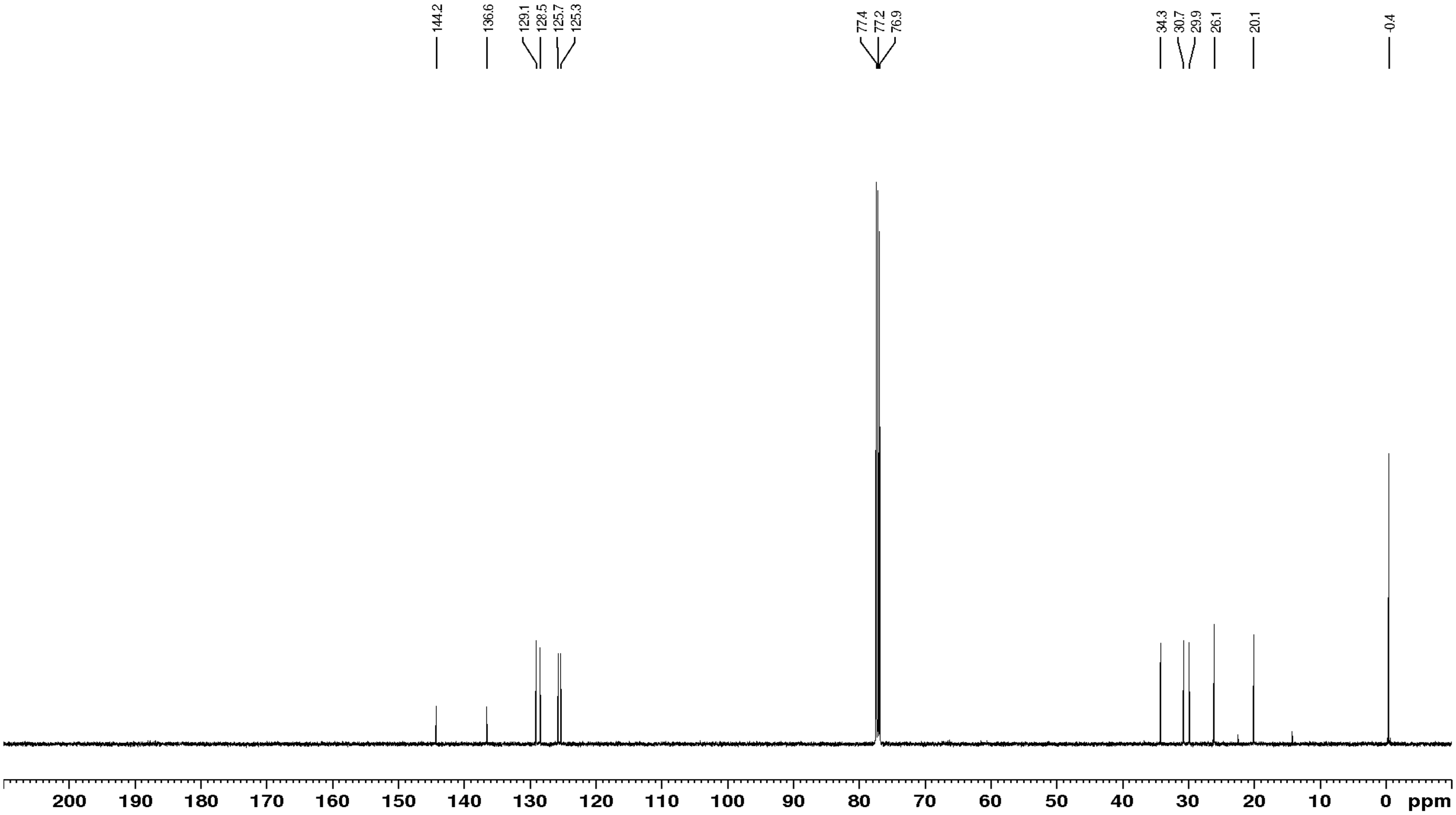

Figure S64. **$^{1}H/^{29}Si$ HMQC** NMR spectrum (500/99 MHz, $CDCl_3$, 298 K, optimized for *J* = 7 Hz) of **4ba** from the reaction of alkene (**1b**) and alkynylsilane (**2a**).

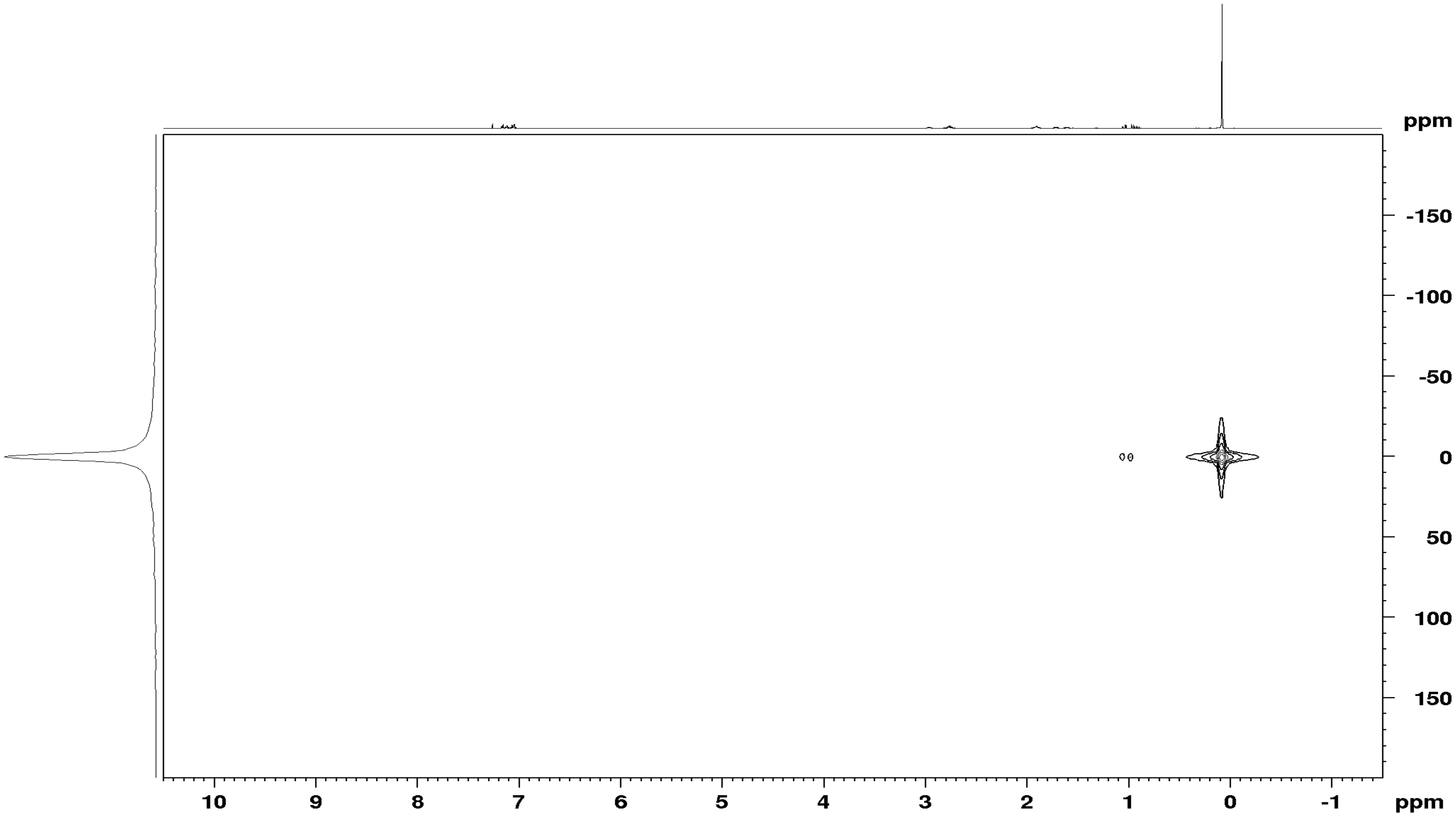

Figure S65. **^{1}H NMR** spectrum (500 MHz, $CDCl_3$, 298 K) of **5ca** from the reaction of alkene (**1c**) and alkynylsilane (**2a**).

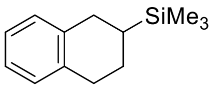

5ca

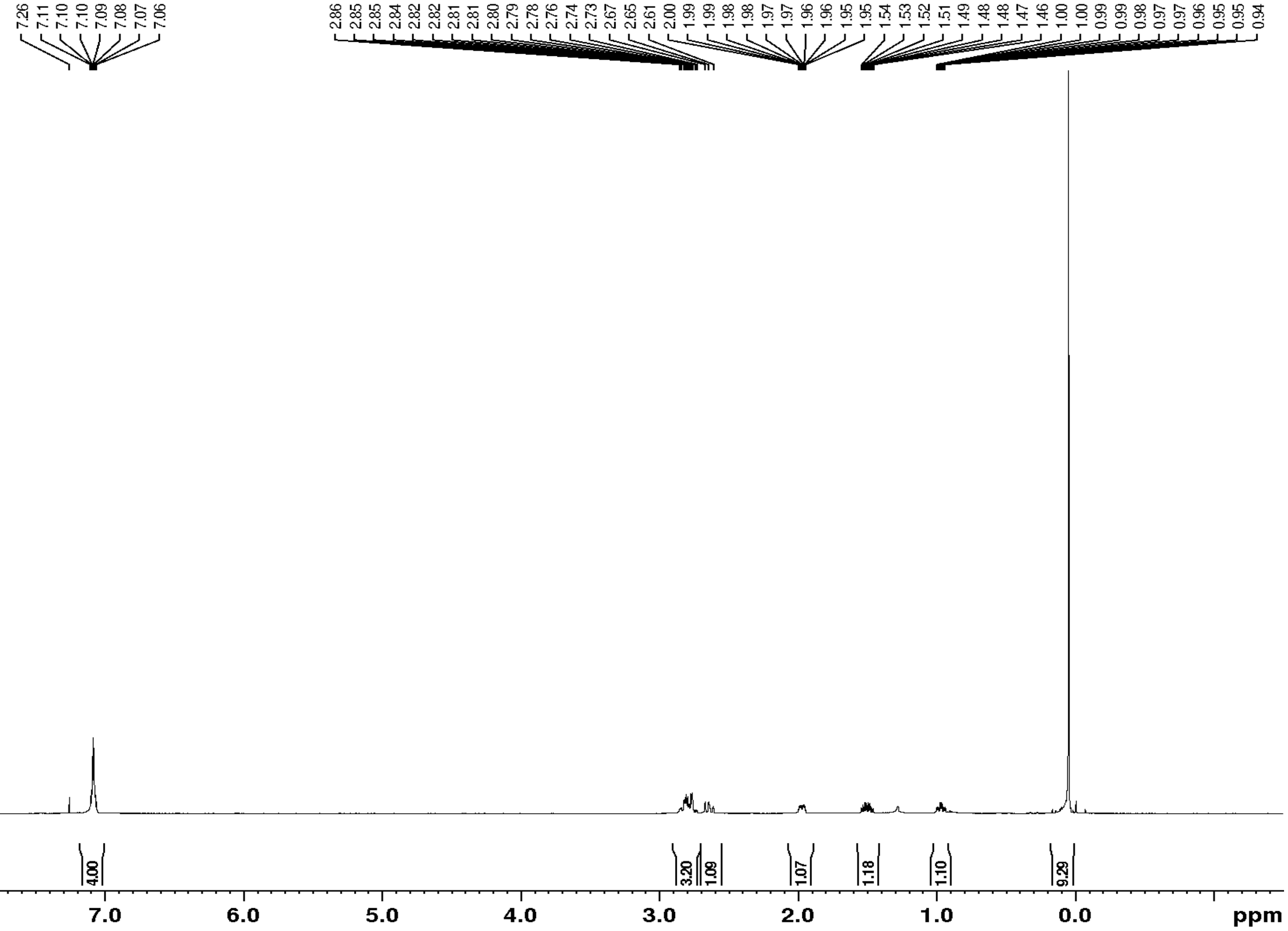

Figure S66. **$^{13}C\{^1H\}$ NMR** spectrum (126 MHz, $CDCl_3$, 298 K) of **5ca** from the reaction of alkene (**1c**) and alkynylsilane (**2a**).

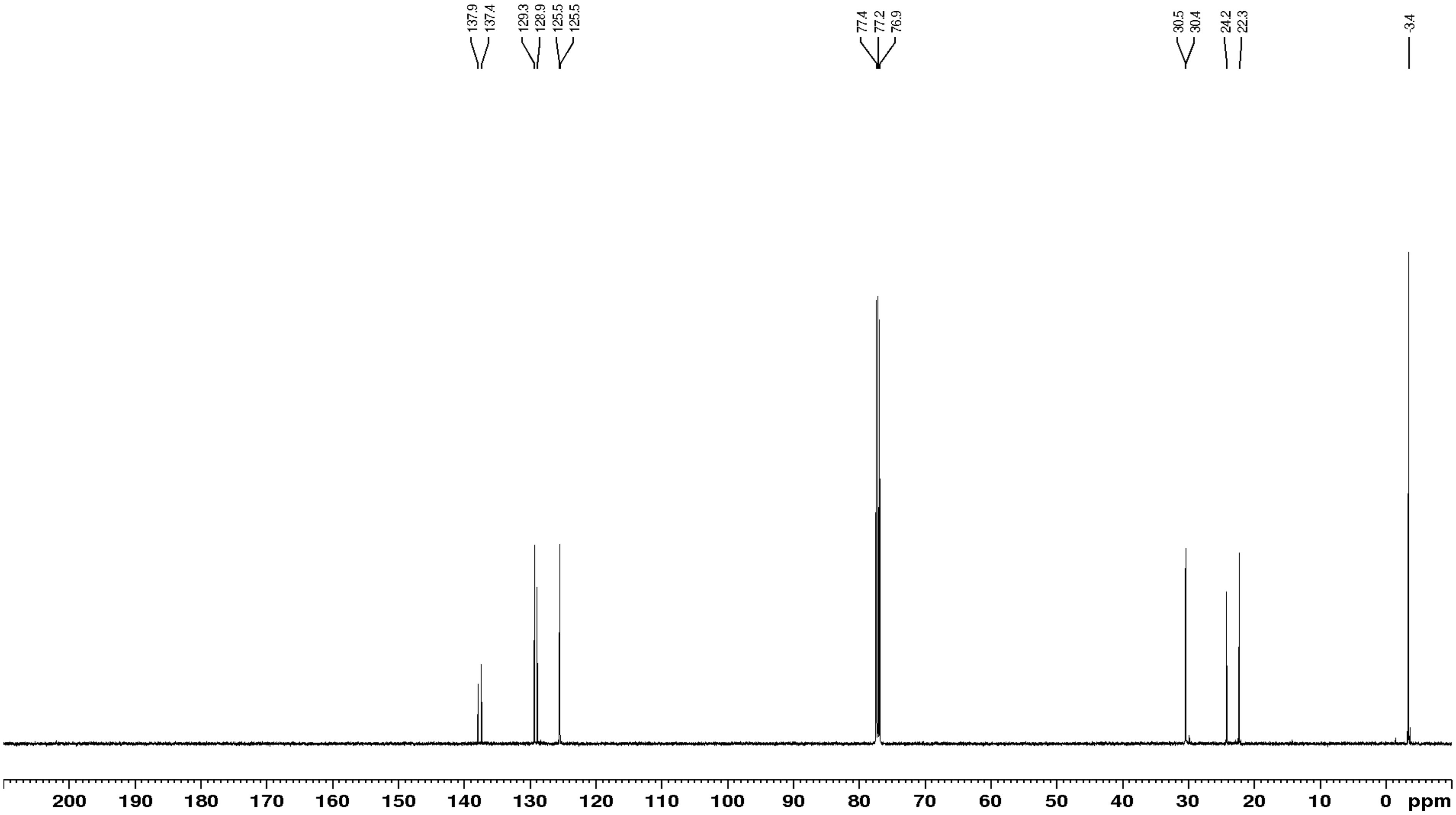

Figure S67. **$^{1}H/^{29}Si$ HMQC** NMR spectrum (500/99 MHz, $CDCl_3$, 298 K, optimized for *J* = 7 Hz) of **5ca** from the reaction of alkene (**1c**) and alkynylsilane (**2a**).

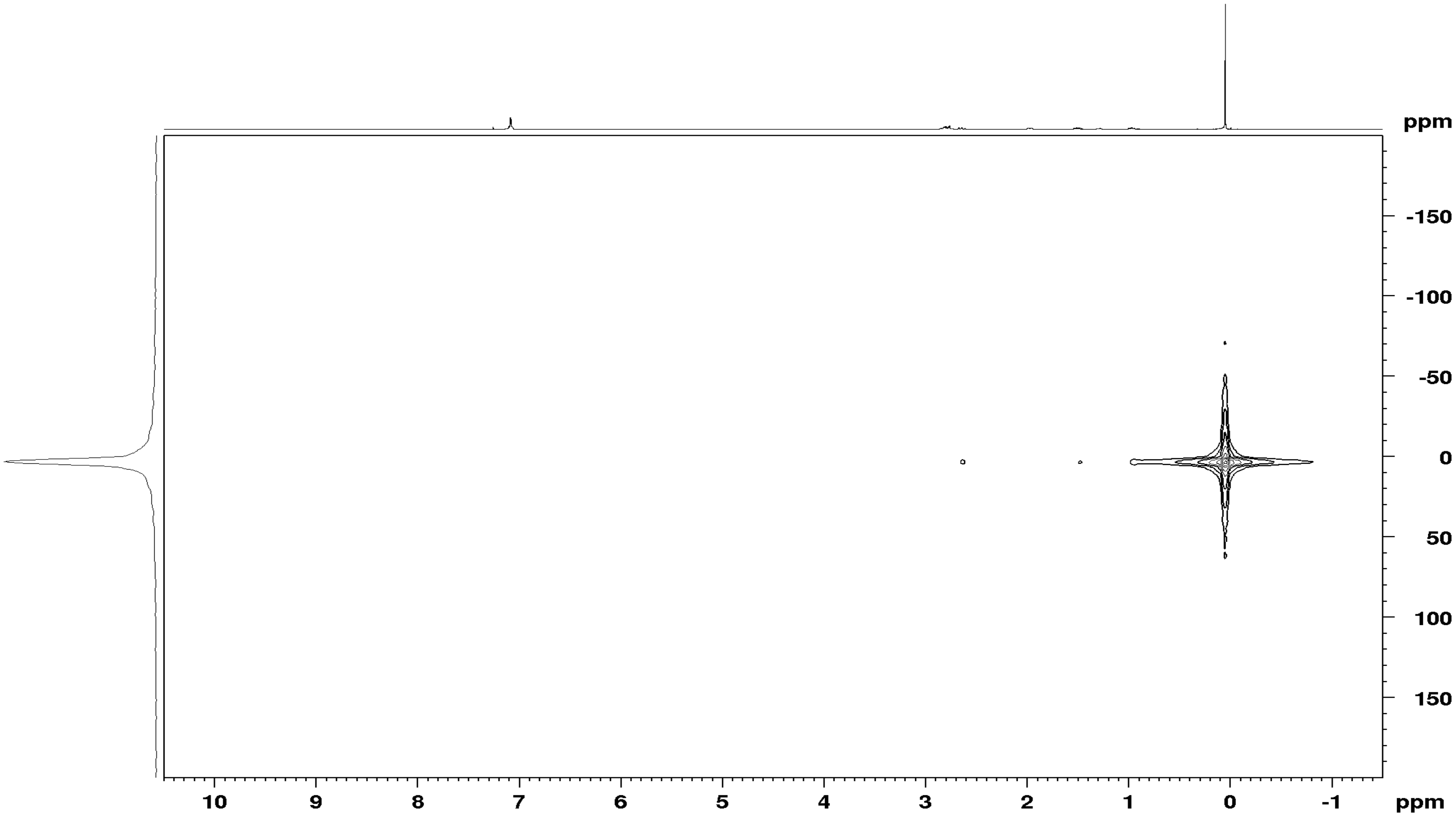

Figure S68. **^{1}H NMR** spectrum (500 MHz, $CDCl_3$, 298 K) of **6ea** from the reaction of alkene (**1e**) and alkynylsilane (**2a**).

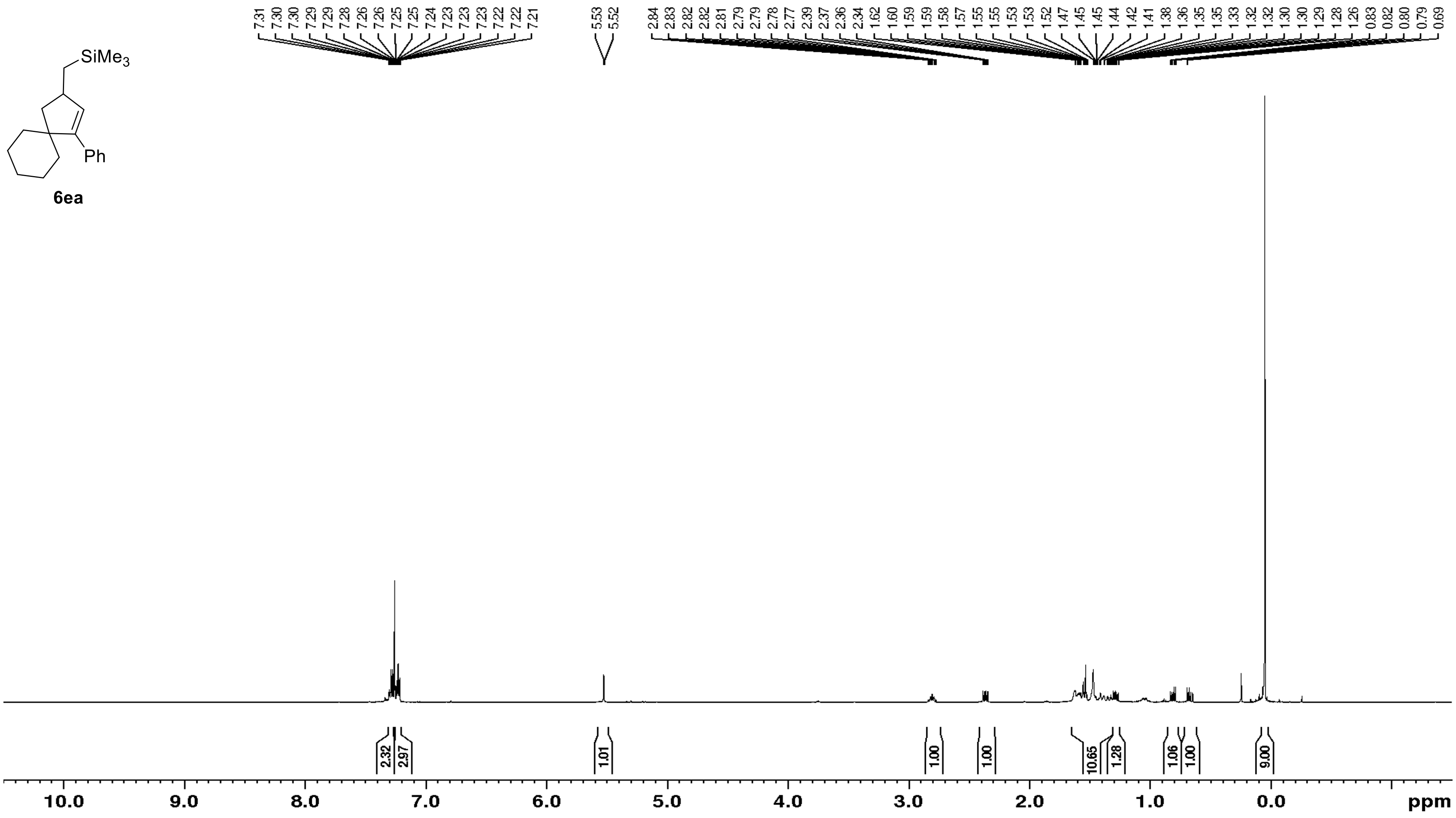

Figure S69. **$^{13}C\{^{1}H\}$ NMR** spectrum (126 MHz, $CDCl_3$, 298 K) of **6ea** from the reaction of alkene (**1e**) and alkynylsilane (**2a**).

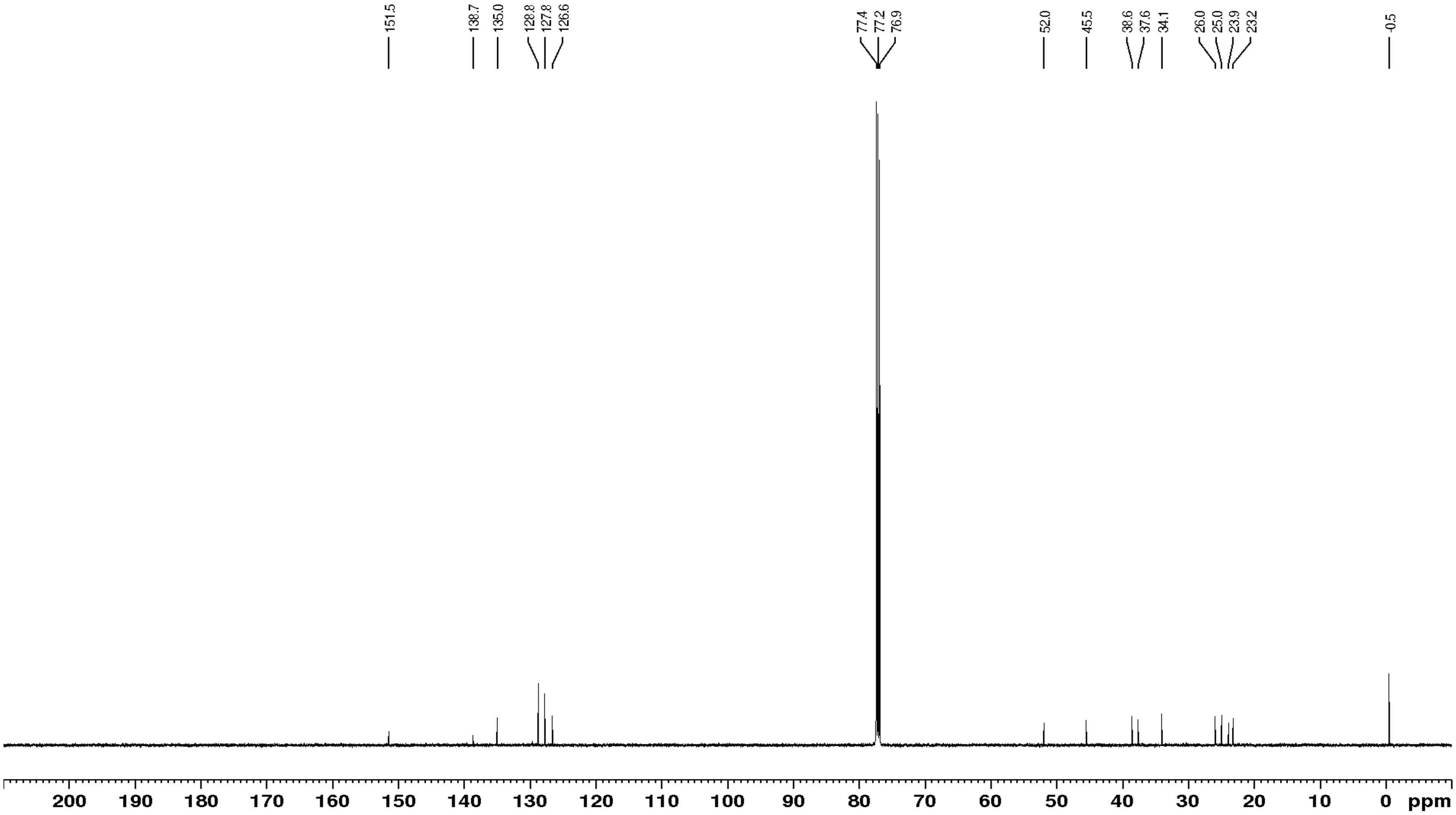

Figure S70. **$^{1}H/^{29}Si$ HMQC** NMR spectrum (500/99 MHz, $CDCl_3$, 298 K, optimized for *J* = 7 Hz) of **6ea** from the reaction of alkene (**1e**) and alkynylsilane (**2a**).

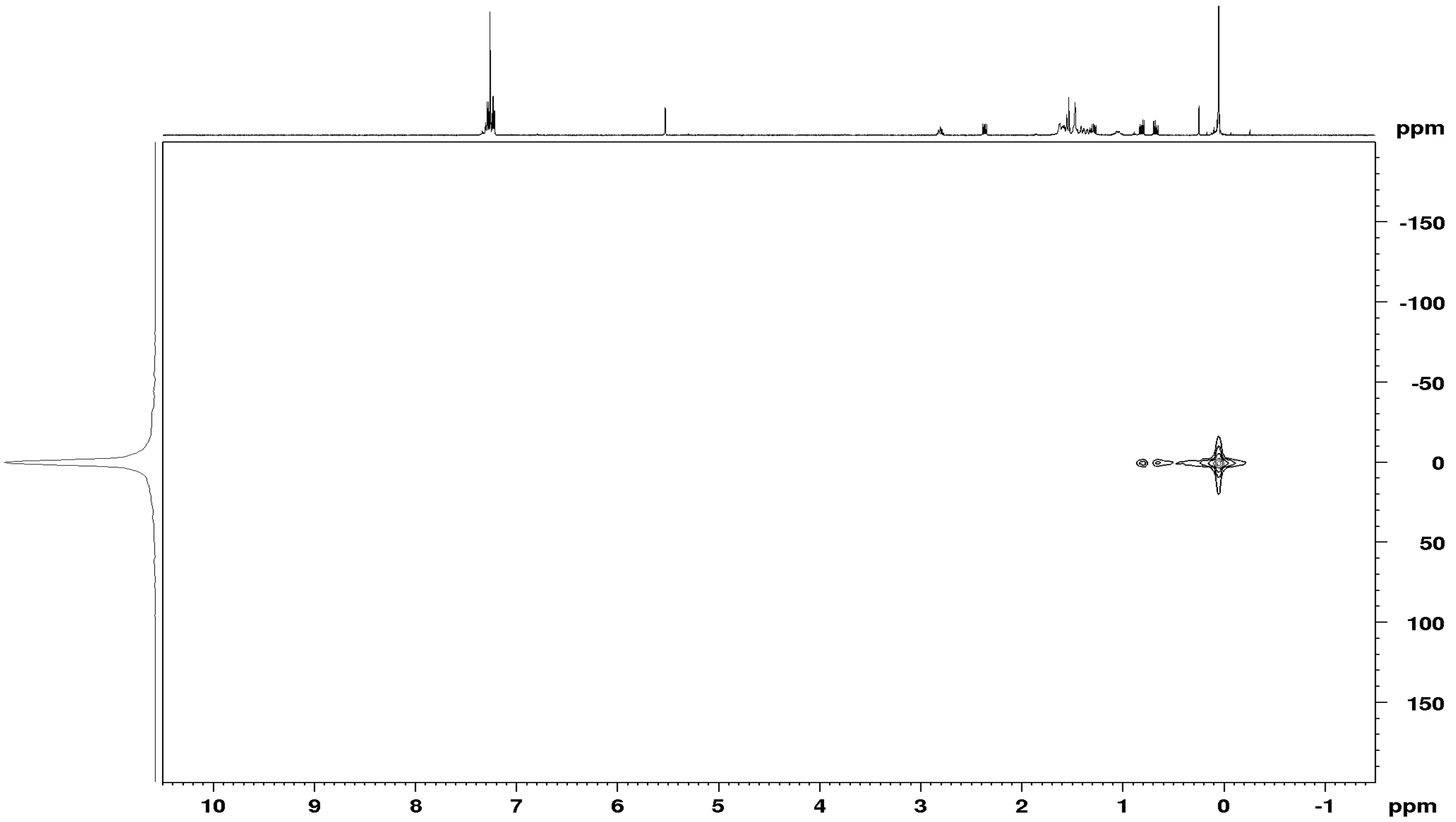

5 Computational Data

Computational Details: The quantum chemical DFT calculations have been performed with the TURBOMOLE 7.4 suite of programs[S23] The structures are fully optimized at the TPSS-D3/def2-TZVP + COSMO level of theory, which combines the TPSS meta-GGA density functional[S24] with the BJ-damped DFT-D3 dispersion correction[S25] and the def2-TZVP basis set,[S26] using the Conductor-like Screening Model (COSMO) continuum solvation model[S27] for PhCl solvent (dielectric constant ε = 5.70 and solvent diameter R_{solv} = 3.41 Å). The density-fitting RI-J approach[S26a, S28] is used to accelerate the geometry optimization and numerical harmonic frequency calculations[S29] in solution. The optimized structures are characterized by frequency analysis to identify the nature of located stationary points (no imaginary frequency for true minima and only one imaginary frequency for transition state) and to provide thermal corrections (at 298.15 K and 1 atm) according to the modified ideal gas-rigid rotor-harmonic oscillator model.[S30] This choice of dispersion-corrected meta-GGA functional makes the efficient exploration of all potential reaction paths possible.

The final solvation free energies in PhCl solution are computed with the COSMO-RS solvation model[S31] (parameter file: BP_TZVP_C30_1601.ctd) using the COSMOtherm program package[S32] on the above TPSS-D3 optimized structures, and corrected by +1.89 kcal·mol^{-1} to account for higher reference solute concentration of 1 mol·L^{-1} usually used in solution. To check the effects of the chosen DFT functional on the reaction energies and barriers, single-point calculations at the meta-GGA TPSS-D3[S24] and hybrid-meta-GGA PW6B95-D3[S33] levels are performed using a larger def2-QZVP basis set.[S26b, S34] The final reaction Gibbs free energies (ΔG) are determined from the electronic single-point energies plus TPSS-D3 thermal corrections and COSMO-RS solvation free energies. The computed relative free energies from both DFT functionals are mostly in very good mutal agreement for reaction energies of about 0.0 ± 1.3 kcal/mol (average ± standard deviations), though about 1.2 ± 1.6 kcal/mol (average ± standard deviations) higher reaction barriers are found at the PW6B95-D3 level as expected. In our discussion, higher-level PW6B95-D3 Gibbs free energies (in kcal/mol, at 298.15 K and 1 mol/L concentration) will be used in our discussion unless specified otherwise. The applied DFT methods in combination with the large AO basis set provide usually accurate electronic energies leading to errors for chemical energies (including barriers) on the order of typically 1-2 kcal/mol. This has been tested thoroughly for the huge data base GMTKN55[S35] which is the common standard in the field of DFT benchmarking.

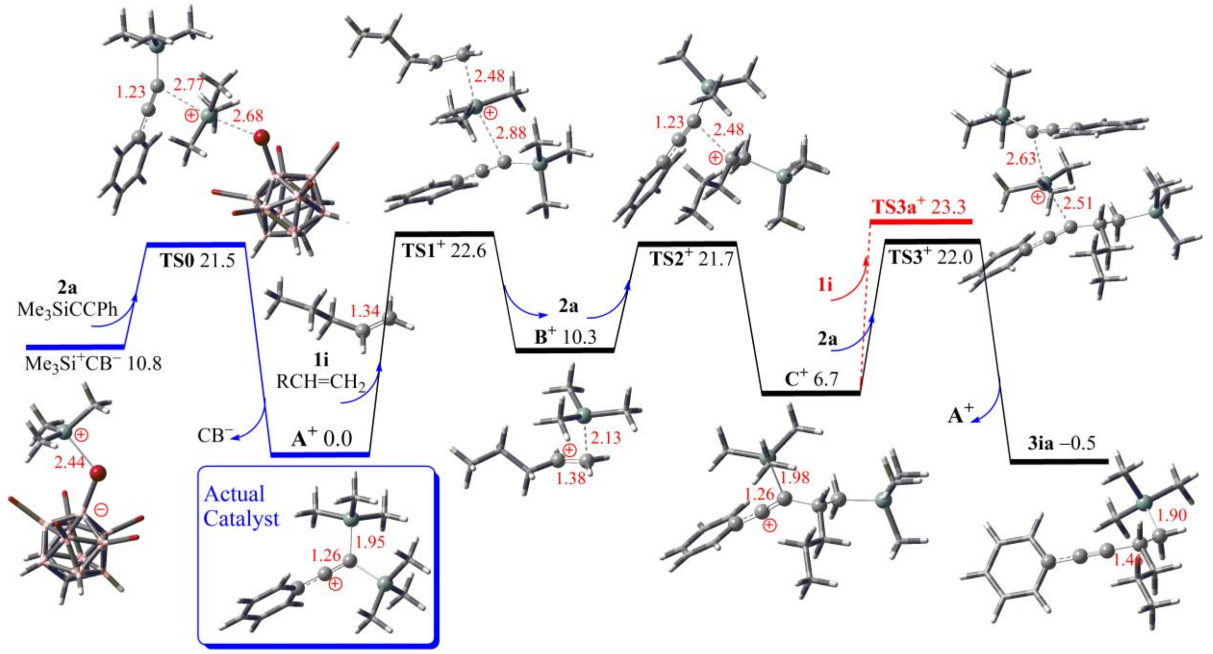

Figure S71. Gibbs free energy profile (in kcal/mol, at 298 K and 1 M concentration in PhCl solution) for the silylium Me_3Si^+ catalyzed silylalkynylation of alkene **1i** ($RCH{=}CH_2$ with R = *n*Pr) with **2a** $Me_3SiCCPh$. Crucial Br, Si and C atoms are highlighted as red, aqua and grey balls in ball-and-stick model, with selected bond lengths indicated by red numbers in Å.

Table S1. TPSS-D3/def2-TZVP + COSMO computed imaginary frequency (ImF), zero-point energies (ZPE), gas-phase enthalpic (Hc) and Gibbs free-energy (Gc) corrections; the COSMO-RS computed solvation enthalpic (Hsol) and Gibbs free-energy (Gsol) corrections in PhCl solution; TPSS-D3/def2-QZVP and PW6B95-D3/def2-QZVP single-point energies (TPSS-D3 and PW6B95-D3); the total PW6B95-D3 free energies G_P; the relative electronic energies (ΔE_T and ΔE_P) and Gibbs free-energies (ΔG_T and ΔG_P) at the TPSS-D3 and PW6B95-D3 levels.

Reactions in PhCl solution	Im cm^{-1}	ZPE kcal /mol	Hc kcal /mol	Gc kcal /mol	Hsol kcal /mol	Gsol kcal /mol	TPSS-D3 E_h	PW6B95-D3 E_h	G_P E_h	ΔE_T kcal /mol	ΔE_P kcal /mol	ΔG_P kcal /mol	ΔG_T kcal /mol
*In PhCl soöution, the Me_3Si^+ affinities decreases in the order **2a** $Me_3SiCCPh$ > **1i** $PrCH=CH_2$ > anion CB^- > PhCl , with Pr = propyl*													
Me_3Si^+	0.0	68.17	73.28	50.49	-54.50	-49.86	-409.11034	-409.47058	-409.46656				
CB^- or $[HCB_{11}H_5Br_6]^-$	0.0	76.33	88.22	47.30	-60.55	-51.73	-15760.64351	-15768.38386	-15768.38790				
**2a** + Me_3Si^+	0.0	199.81	214.19	157.73	-67.93	-58.41	-1126.53903	-1127.60072	-1127.43641	0.00	0.00	*0.00*	0.00
**A⁺** or $(Me_3Si)_2CCPh^+$	0.0	201.41	216.08	171.80	-46.53	-40.25	-1126.63063	-1127.68702	-1127.47437	-57.48	-54.15	*-23.82*	-27.15
**1i** + Me_3Si^+	0.0	152.83	162.62	116.68	-60.17	-52.89	-605.77648	-606.34401	-606.23632	0.00	0.00	*0.00*	0.00
**B⁺** or $PrCHCH_2SiMe_3^+$	0.0	155.29	164.78	131.21	-49.19	-44.10	-605.83518	-606.39964	-606.25781	-36.83	-34.91	*-13.49*	-15.41
Me_3Si^+ + CB^-	0.0	144.50	161.50	97.79	-115.06	-101.59	-16169.75385	-16177.85444	-16177.85446	0.00	0.00	*0.00*	0.00
$Me_3Si^+CB^-$	0.0	145.85	163.21	111.79	-29.81	-20.85	-16169.92568	-16178.02317	-16177.87523	-107.83	-105.88	*-13.03*	-14.98
PhCl + Me_3Si^+	0.0	124.55	133.71	88.59	-63.65	-55.69	-1101.16272	-1102.11216	-1102.05371	0.00	0.00	*0.00*	0.00
$PhClSiMe_3^+$	0.0	126.20	135.51	101.75	-49.55	-43.92	-1101.21729	-1102.16330	-1102.06813	-34.25	-32.09	*-9.05*	-11.20
*The overall silylalkynylation reaction of **1i** and **2a** is slightly exergonic by -0.49 kcal/mol*													
**1i** + **2a**	0.0	216.30	230.25	173.44	-19.09	-11.58	-914.09483	-915.00357	-914.73960	0.00	0.00	*0.00*	0.00
**3ia**	0.0	218.60	232.20	189.55	-16.18	-10.47	-914.11798	-915.02877	-914.74038	-14.53	-15.82	*-0.49*	0.80
*Very facile Me_3Si^+ transfer from the silylium salt $Me_3Si^+CB^-$ to **2a** $Me_3SiCCPh$ and **1i** $PrCH=CH_2$ over a low barrier of 10.7 kcal/mol*													
$Me_3Si^+CB^-$ + **2a**	0.0	277.49	304.12	219.04	-43.24	-29.41	-16887.35437	-16896.15331	-16895.84508	0.00	0.00	*0.00*	0.00
**TS0**	112.4i	277.90	305.00	234.26	-39.77	-28.38	-16887.36216	-16896.15910	-16895.82800	-4.89	-3.63	*10.72*	9.46
**A⁺** + CB^-	0.0	277.75	304.30	219.10	-107.08	-91.98	-16887.27414	-16896.07088	-16895.86227	50.34	51.72	*-10.79*	-12.17
*.. facile Me_3Si^+ transfer from the silylium salt $Me_3Si^+CB^-$ to alkene **1i** $PrCH=CH_2$ is only -0.45 kcal/mol exergonic and thus is thermodynamically less favorable.*													
$Me_3Si^+CB^-$ + **1i**	0.0	230.51	252.55	177.99	-35.48	-23.88	-16366.59183	-16374.89659	-16374.64499	0.00	0.00	*0.00*	0.00
**TS0a**	22.9i	232.03	253.94	193.37	-41.09	-30.32	-16366.58809	-16374.89107	-16374.62822	2.34	3.47	*10.52*	9.40
**B⁺** + CB^-	0.0	231.62	253.01	178.51	-109.74	-95.83	-16366.47869	-16374.78350	-16374.64571	70.99	70.97	*-0.45*	-0.43

*Full Catalytic cycle starting from the stable cation **A**$^+$ (or $(Me_3Si)_2CCPh^+$): Me_3Si^+ transfer to alkene **1i** at first*

**A**$^+$ + **1i**	0.0	286.07	305.42	237.99	-52.19	-43.28	-1323.29678	-1324.56045	-1324.24413	0.00	0.00	*0.00*	0.00
**TS1**$^+$	104.5i	287.54	306.83	252.77	-49.79	-42.26	-1323.28321	-1324.54664	-1324.20815	8.51	8.66	*22.57*	22.42
**B**$^+$ + **2a**	0.0	286.93	305.69	238.46	-62.61	-52.65	-1323.26387	-1324.52978	-1324.22766	20.65	19.24	*10.33*	11.74

*..alternative C-C bond formation between **A**$^+$ and **1i** is kinetically 2.3 kcal/mol less favorable via **TS1a**$^+$*

**A**$^+$ + **1i**	0.0	286.07	305.42	237.99	-52.19	-43.28	-1323.29678	-1324.56045	-1324.24413	0.00	0.00	*0.00*	0.00
**TS1a**$^+$	213.7i	287.74	306.22	254.51	-48.00	-41.09	-1323.29024	-1324.54760	-1324.20449	4.10	8.06	*24.87*	20.92
**Ba**$^+$	0.0	289.68	307.67	257.18	-48.23	-41.38	-1323.29574	-1324.55619	-1324.20928	0.65	2.67	*21.86*	19.84

*..Facile C-C bond formation between **2a** SimCCPh and carbocation **B**$^+$ $PrCHCH_2SiMe_3^+$ is -3.6 kcal/mol exergonic over a low barrier of 12.2 kcal/mol*

**2a** + **B**$^+$	0.0	286.93	305.69	238.46	-62.61	-52.65	-1323.26387	-1324.52978	-1324.22766	20.65	19.24	*10.33*	11.74
**TS2**$^+$	42.1i	287.78	306.40	254.20	-47.69	-40.56	-1323.29340	-1324.55298	-1324.20952	2.12	4.69	*21.72*	19.15
**TS2a**$^+$	60.0i	287.14	306.07	252.81	-48.16	-40.99	-1323.28873	-1324.54878	-1324.20822	5.05	7.32	*22.53*	20.26
**C**$^+$	0.0	289.17	307.78	255.48	-47.67	-40.65	-1323.31564	-1324.57883	-1324.23346	-11.84	-11.54	*6.69*	6.39

*..followed by direct Me_3Si^+ transfer from **C**$^+$ to **2a** $Me_3SiCCPh$ which encounters a 22.0 kcal/mol barrier via **TS3**$^+$ to form the desired product and regenerated **A**$^+$*

**A**$^+$ + **1i** + **2a**	0.0	417.71	446.33	345.24	-65.62	-51.83	-2040.72546	-2042.69059	-2042.21398	0.00	0.00	*0.00*	0.00
**C**$^+$ + **2a**	0.0	420.82	448.69	362.73	-61.10	-49.20	-2040.74432	-2042.70897	-2042.20331	-11.84	-11.54	*6.69*	6.39
**TS3**$^+$	158.5i	420.39	449.28	375.51	-51.05	-41.78	-2040.74894	-2042.71375	-2042.17890	-14.74	-14.53	*22.01*	21.81
**A**$^+$ + **3ia**	0.0	420.02	448.28	361.35	-62.70	-50.72	-2040.74861	-2042.71579	-2042.21475	-14.53	-15.82	*-0.49*	0.80

*The Me_3Si^+ transfer from **C+** to alkene **1i** $PrCH{=}CH_2$ is 3.2 kcal/mol endergonic over 1.3 kcal/mol higher barrier, and thus is less favorable than that to **2a***

**A**$^+$ + 2***1i**	0.0	370.73	394.76	304.19	-57.86	-46.31	-1519.96292	-1521.43388	-1521.01388	0.00	0.00	*0.00*	0.00
**C**$^+$ + **1i**	0.0	373.83	397.12	321.68	-53.34	-43.68	-1519.98178	-1521.45226	-1521.00321	-11.84	-11.54	*6.69*	6.39
**TS3a**$^+$	86.7i	374.65	398.26	335.64	-52.23	-43.72	-1519.97267	-1521.44494	-1520.97673	-6.12	-6.94	*23.31*	24.14
**B**$^+$ + **3ia**	0.0	373.89	396.98	320.76	-65.37	-54.57	-1519.95316	-1521.42841	-1520.99819	6.12	3.43	*9.85*	12.54

*..followed by faster Me_3Si^+ transfer from **B**$^+$ to **2a** $Me_3SiCCPh$*

**B**$^+$ + **2a**	0.0	286.93	305.69	238.46	-62.61	-52.65	-1323.26387	-1324.52978	-1324.22766	20.65	19.24	*9.85*	12.54
**TS1**$^+$	104.5i	287.54	306.83	252.77	-49.79	-42.26	-1323.28321	-1324.54664	-1324.20815	8.51	8.66	*22.09*	23.22
**A**$^+$ + **1i**	0.0	286.07	305.42	237.99	-52.19	-43.28	-1323.29678	-1324.56045	-1324.24413	0.00	0.00	*-0.48*	0.80

*Methyl shift within **A**$^+$ is unlikely due to a high barrier of 32.3 kcal/mol*

**A**$^+$	0.0	201.41	216.08	171.80	-46.53	-40.25	-1126.63063	-1127.68702	-1127.47437	0.00	0.00	*0.00*	0.00

**TSAm$^+$**	470.4i	201.16	215.16	172.08	-47.77	-41.44	-1126.57811	-1127.63410	-1127.42291	32.96	33.21	*32.29*	32.05
**Am$^+$**	0.0	203.56	217.21	175.09	-47.92	-41.77	-1126.63607	-1127.69405	-1127.47858	-3.41	-4.41	*-2.64*	-1.64

*Again, 1,3 or 1,5-methyl shifts within **C$^+$** is unlikely due to high barriers and endergonic reaction step*

**C$^+$**	0.0	289.17	307.78	255.48	-47.67	-40.65	-1323.31564	-1324.57883	-1324.23346	0.00	0.00	*6.69*	6.39
**TSCm$^+$**	420.0i	288.51	306.68	255.26	-50.52	-43.23	-1323.27031	-1324.53367	-1324.19276	28.44	28.34	*32.23*	32.03
**Cm$^+$**	0.0	290.56	308.66	257.46	-52.47	-44.90	-1323.30104	-1324.56675	-1324.22500	9.16	7.58	*12.00*	13.28
**TSCme$^+$**	409.2i	288.85	306.74	256.18	-48.12	-41.29	-1323.27764	-1324.53797	-1324.19251	23.84	25.64	*32.39*	30.29
**Cme$^+$**	0.0	290.21	308.16	257.42	-53.04	-45.59	-1323.29961	-1324.56498	-1324.22441	10.06	8.69	*12.37*	13.44

*The catalytic cation **A$^+$** cannot be trapped by the weakly coordinating CB$^-$ anion*

CB$^-$ + **A$^+$**	0.0	277.75	304.30	219.10	-107.08	-91.98	-16887.27414	-16896.07088	-16895.86227	0.00	0.00	*0.00*	0.00
**A$^+$**.CB$^-$	0.0	278.50	305.70	234.94	-42.11	-31.05	-16887.38190	-16896.17841	-16895.85049	-67.62	-67.48	*7.40*	7.26

*Competitive **A$^+$** catalyzed dimerization of **2a** is still possible, making its excess use necessary in experiment.*

*The **A$^+$** catalyzed dimerization of **2a** is -1.9 kcal/mol over a moderate barrier of 21.2 kcal/mol to form the neutral dimer **aP** $(Me_3Si)_2C$=CPhCCPh*

2***2a** + **A$^+$**	0.0	464.70	497.89	386.29	-73.38	-57.35	-2561.48800	-2563.94730	-2563.41407	0.00	0.00	*0.00*	0.00
**aTS1$^+$** + **2a**	185.2i	465.09	498.42	401.44	-61.62	-48.75	-2561.49600	-2563.94842	-2563.38036	-5.02	-0.71	*21.16*	16.85
**aA$^+$** + **2a**	0.0	467.12	499.79	404.81	-63.59	-50.50	-2561.51516	-2563.97158	-2563.40094	-17.04	-15.23	*8.24*	6.44
**aTS2$^+$**	122.9i	467.00	500.47	419.16	-54.28	-43.89	-2561.52771	-2563.98642	-2563.38537	-24.91	-24.55	*18.01*	17.65
**aP** + **A$^+$**	0.0	466.18	499.35	402.90	-67.30	-53.98	-2561.52168	-2563.97917	-2563.41711	-21.14	-20.00	*-1.91*	-3.04

Table S2. TPSS-D3/def2-TZVP + COSMO optimized Cartesian coordinates (in Å) in PhCl solution. Each structure is labeled by a specific name (See **Table S1**), followed by the number of atoms, the total energy (in hartrees), and the detailed atomic coordinates (in double-column text list).

1i : alkene $PrCH=CH_2$ with Pr = propyl

15

Energy = -196.6533479300

C	3.1477416	-1.0285840	-1.5531870
C	2.7306292	-0.7549538	-2.7917049
H	3.8451980	-1.8545583	-1.3995041
C	2.7530731	-0.2681345	-0.3205017
H	3.0624428	-1.3356940	-3.6486103
H	2.0382296	0.0618177	-2.9894746
C	3.9553182	0.3980581	0.3768390
H	2.2785985	-0.9565805	0.3949847
H	2.0064907	0.4942137	-0.5773828
H	4.7093767	-0.3675076	0.6024148
H	4.4239105	1.1044211	-0.3200265
C	3.5538239	1.1238484	1.6646057
H	3.1095644	0.4260872	2.3843898
H	2.8149777	1.9073018	1.4584086
H	4.4204953	1.5946925	2.1411979

2a : alkynylsilane $Me_3SiCCPh$

26

Energy = -717.3959015329

C	0.8833890	0.0049668	0.0011941
C	-0.3392871	0.0032325	0.0019433
C	-1.7625847	0.0018135	0.0019525
C	-2.4767964	-1.2134498	0.0092220
C	-2.4795351	1.2155193	-0.0066999
C	-3.8688869	-1.2096134	0.0069472
H	-1.9285487	-2.1503486	0.0160099
C	-3.8715856	1.2085813	-0.0083368
H	-1.9334244	2.1536864	-0.0125730
C	-4.5708999	-0.0013168	-0.0018118
H	-4.4081310	-2.1525637	0.0115471
H	-4.4129082	2.1503360	-0.0145710
H	-5.6570310	-0.0025205	-0.0031692
Si	2.7220564	0.0013352	0.0002339
C	3.3116606	-0.9132389	-1.5337096
C	3.3135472	-0.8735742	1.5564448
C	3.3153722	1.7854227	-0.0229662
H	2.9540149	-0.4229164	-2.4461858
H	4.4079701	-0.9375158	-1.5675247
H	2.9487708	-1.9473219	-1.5388059
H	2.9567575	-0.3602551	2.4565283
H	2.9510060	-1.9073058	1.5883714
H	4.4098977	-0.8966454	1.5896399
H	4.4116920	1.8253154	-0.0245765
H	2.9552428	2.3082572	-0.9162772
H	2.9569939	2.3306793	0.8575485

3ia : product $Me_3SiCH_2CHPrCCPh$

41

Energy = -914.0707644624

C	0.2036610	-0.7265129	-0.1586300
C	-0.9504722	-0.3521677	-0.1025441

C	-2.2912941	0.1232704	-0.0477467
C	-2.6345996	1.3558876	-0.6391357
C	-3.2969166	-0.6215218	0.5996321
C	-3.9440000	1.8251872	-0.5817264
H	-1.8651369	1.9346759	-1.1404210
C	-4.6039484	-0.1446400	0.6522277
H	-3.0396393	-1.5712795	1.0581218
C	-4.9335612	1.0786730	0.0629892
H	-4.1933733	2.7769315	-1.0420453
H	-5.3683126	-0.7294966	1.1558058
H	-5.9542086	1.4471603	0.1054094
Si	2.4116549	1.6569202	0.0878476
C	4.0717167	2.4560930	0.5005443
C	1.0561650	2.5408986	1.0551149
C	2.0853144	1.8033937	-1.7647555
H	4.0631781	3.5275294	0.2658641
H	4.3067137	2.3505928	1.5668787
H	4.8870129	1.9938531	-0.0691168
H	1.0135281	3.6024749	0.7817285
H	0.0745288	2.0976287	0.8609919
H	1.2452486	2.4810018	2.1339357
H	2.8635500	1.2976344	-2.3491925
H	1.1194602	1.3584939	-2.0301965
H	2.0673729	2.8557767	-2.0730777
C	1.6007238	-1.1569142	-0.2032007
C	2.5153889	-0.1788996	0.5812355
H	1.9138307	-1.1348252	-1.2578212
C	1.7557292	-2.6115027	0.2990947
H	3.5523979	-0.5179473	0.4485412
H	2.2953882	-0.2711282	1.6539213
C	0.9666238	-3.6361804	-0.5193699
H	1.4429722	-2.6522322	1.3507831
H	2.8251236	-2.8586594	0.2734317
H	-0.0953589	-3.3602209	-0.5093112
H	1.2919531	-3.5874301	-1.5675003
C	1.1362657	-5.0643794	0.0071028
H	0.7902213	-5.1425245	1.0445430
H	2.1888003	-5.3708047	-0.0165965
H	0.5640150	-5.7800101	-0.5930134

aA⁺ : cation $(Me_3Si)_2$CCPh$(SiMe_3)$CCPh

65

Energy = -1844.037277887

C	1.3910254	-0.8972033	0.0941307
C	0.7025750	0.2456812	-0.1152807
C	1.2353094	1.5386712	-0.6234618
C	0.6732802	2.1575902	-1.7487221
C	2.2750992	2.1781976	0.0648410
C	1.1689433	3.3777695	-2.1998089
H	-0.1412853	1.6710852	-2.2782622
C	2.7559251	3.4108047	-0.3746063
H	2.6963173	1.7077872	0.9476338
C	2.2073067	4.0098799	-1.5098505
H	0.7432108	3.8407188	-3.0849564
H	3.5577229	3.9020298	0.1684203
H	2.5834851	4.9684330	-1.8542515
Si	3.1174671	-1.1353775	-0.7021773
C	3.4729022	0.0170517	-2.1494400
C	3.1474669	-2.8601106	-1.4705276
C	4.4637781	-0.9610793	0.6023622
H	3.7172128	1.0404322	-1.8562071
H	4.3346893	-0.3966265	-2.6898122

H	2.6307408	0.0548419	-2.8489118
H	2.9593647	-3.6817292	-0.7755434
H	2.4151154	-2.9280692	-2.2836480
H	4.1405967	-3.0184793	-1.9098698
H	5.4408613	-1.1747794	0.1513408
H	4.4969112	0.0588314	1.0013932
H	4.3220553	-1.6483785	1.4419201
Si	0.6526167	-2.4013100	1.0019687
C	-0.2037626	-3.5045593	-0.2629382
H	-0.9691080	-2.9485267	-0.8170341
H	0.4995610	-3.9152769	-0.9933734
H	-0.6966131	-4.3437001	0.2434259
C	-0.5860366	-1.9659470	2.3597257
H	-0.7977286	-2.8934798	2.9076875
H	-0.1745321	-1.2537437	3.0818527
H	-1.5395307	-1.5759455	1.9989979
C	2.0168972	-3.3204771	1.9197868
H	2.8453716	-3.6585735	1.2934360
H	2.4303226	-2.6900522	2.7160937
H	1.5780944	-4.2053415	2.3975698
C	-0.7577286	0.3764497	0.2104326
C	-1.8621060	0.0052621	-0.2678407
C	-3.1402439	-0.3109346	-0.7014794
C	-3.7894806	-1.4803508	-0.2222338
C	-3.7998247	0.5429362	-1.6264469
C	-5.0678434	-1.7775022	-0.6609699
H	-3.2760056	-2.1246700	0.4825990
C	-5.0796569	0.2299704	-2.0499419
H	-3.2905164	1.4303337	-1.9866322
C	-5.7108282	-0.9250748	-1.5688497
H	-5.5728411	-2.6671087	-0.3004860
H	-5.5926874	0.8771138	-2.7531187
H	-6.7146447	-1.1643014	-1.9058407
Si	-1.2136239	1.6850238	1.6832933
C	-1.2826757	3.3547834	0.8523900
C	0.2020435	1.5182749	2.9045272
C	-2.8648260	1.2148980	2.4170970
H	-0.3138472	3.6564487	0.4464942
H	-2.0229119	3.3743812	0.0461451
H	-1.5850455	4.0923146	1.6078024
H	0.9740730	0.8258225	2.5595699
H	0.6672481	2.4993572	3.0500102
H	-0.1723514	1.1644505	3.8704035
H	-3.0650394	1.8884946	3.2598598
H	-3.6865448	1.3257210	1.7026844
H	-2.8688347	0.1907716	2.8031066

A$^+$.CB$^-$: contact ion pair of **A**$^+$ and anion CB$^-$

63

Energy = -16887.01027992

B	2.1553203	-1.4293765	-0.2660781
B	2.1788072	0.3377477	-0.5109105
B	2.2807505	-1.7452833	1.4881883
B	1.3299741	-0.3565489	0.8982383
B	3.7040962	-1.9008116	0.4480012
B	3.6410538	-0.6190777	-0.7826657
Br	1.1615288	-2.6193488	-1.4667511
B	2.3179692	1.1167130	1.0883966
B	3.7407171	0.9489016	0.0506619
Br	1.2157272	1.1858593	-1.9971114
B	2.3819520	-0.1692553	2.3279225
B	3.8436684	-1.1253808	2.0424557

Br	1.4239101	-3.3029934	2.3120086
Br	-0.6168911	-0.3127007	1.0580809
C	4.5349403	-0.4298102	0.6518365
H	4.2904623	-2.9015602	0.2257626
H	4.1832086	-0.7695929	-1.8210483
B	3.8662921	0.6358655	1.7970996
Br	1.5083129	2.8679696	1.4538837
H	4.3490280	1.8372185	-0.4351511
Br	1.6461708	0.0929691	4.1237514
H	4.5233121	-1.6122813	2.8765867
Si	-3.4313964	-1.5735434	-1.6580703
H	5.6136768	-0.4550267	0.5697310
H	4.5595873	1.3168720	2.4682726
C	-5.0999015	-1.9594028	-2.4187516
C	-2.7253456	-3.0345388	-0.7367242
C	-2.2277826	-0.8789919	-2.9042781
H	-5.5465008	-1.0723020	-2.8801954
H	-4.9676296	-2.7184307	-3.2002117
H	-5.8036241	-2.3577115	-1.6808625
H	-1.8566684	-2.7558167	-0.1337268
H	-3.4677440	-3.5214807	-0.0983747
H	-2.3918969	-3.7655045	-1.4846557
H	-2.6427668	-0.0132607	-3.4328485
H	-1.2919384	-0.5800060	-2.4204734
H	-1.9886807	-1.6457912	-3.6507566
C	-3.4323962	1.0257202	-0.7236353
C	-3.7974393	-0.1328619	-0.3927779
C	-3.0309735	2.3042618	-1.0855064
Si	-4.7092536	-0.2840448	1.3206586
C	-3.8941727	3.1175341	-1.8681615
C	-1.7630032	2.7896040	-0.6738979
C	-6.5226975	-0.4833291	0.9026560
C	-4.0222640	-1.7584559	2.2392878
C	-4.3631715	1.3079726	2.2402741
C	-3.4846631	4.3888913	-2.2286057
H	-4.8595673	2.7288960	-2.1745679
C	-1.3746514	4.0665464	-1.0444793
H	-1.1147111	2.1534817	-0.0817284
H	-6.8880431	0.3536560	0.2982860
H	-6.7148119	-1.4133717	0.3584298
H	-7.1043228	-0.5109310	1.8326780
H	-2.9287774	-1.7462174	2.2812542
H	-4.4005644	-1.7186397	3.2688654
H	-4.3449781	-2.7061306	1.7992909
H	-4.8547195	1.2821381	3.2199872
H	-3.2880922	1.4392315	2.4072169
H	-4.7389998	2.1836444	1.6995308
C	-2.2290415	4.8607246	-1.8169871
H	-4.1324297	5.0194425	-2.8283262
H	-0.4034699	4.4404184	-0.7386425
H	-1.9155388	5.8596890	-2.1049306

Am⁺ : cation after Si-to-C methyl shift of **A⁺**

39

Energy = -1126.630293060

C	0.6802419	-0.2910994	0.1072101
C	-0.2520524	-1.2898714	0.0231249
C	-1.6479291	-0.8315303	0.1793824
C	-2.7009974	-1.2148994	-0.6520175
C	-1.8562737	0.2628258	1.0961860
C	-3.8930138	-0.4882617	-0.6447547
H	-2.5736699	-2.0326384	-1.3534224

C	-3.0975476	0.9554805	1.1112289
H	-1.2908817	0.2406317	2.0319093
C	-4.0880636	0.6100106	0.2100876
H	-4.6838150	-0.7695239	-1.3337368
H	-3.2630864	1.7358893	1.8471854
H	-5.0288215	1.1491334	0.1915775
Si	-0.2279812	1.3241044	0.2741584
C	-0.8237283	2.0663754	-1.3254457
C	3.3114412	1.2371114	0.1092358
C	0.3038141	2.5815843	1.5361992
H	-1.5424836	2.8718413	-1.1428080
H	0.0344577	2.4945453	-1.8584071
H	-1.2838071	1.3135651	-1.9720556
H	4.3905149	1.1879654	-0.0792341
H	2.8931061	1.9600735	-0.6028649
H	3.1728524	1.6335278	1.1213019
H	1.1090861	3.2004478	1.1235364
H	-0.5272355	3.2501570	1.7871125
H	0.6650580	2.1085080	2.4543497
Si	2.5545156	-0.4733522	-0.0940354
C	2.9240922	-1.1026631	-1.8290848
H	2.5143760	-2.1016599	-2.0105112
H	2.5096336	-0.4256200	-2.5850816
H	4.0093263	-1.1526445	-1.9809463
C	0.0037711	-2.7390149	-0.2764021
H	1.0680165	-2.9412311	-0.3964044
H	-0.3890804	-3.3720493	0.5272779
H	-0.5069650	-3.0380276	-1.1996836
C	3.2331612	-1.6292592	1.2248598
H	4.3247215	-1.6947880	1.1384313
H	2.9975244	-1.2582289	2.2289998
H	2.8294476	-2.6435293	1.1370751

aP : neutral dimer $(Me_3Si)_2$CCPhCCPh

52

Energy = -1434.822929680

C	1.5206886	-0.9545249	-0.0514740
C	0.7179848	0.0743312	-0.4844375
C	1.2273798	1.4056185	-0.9253312
C	0.7668355	2.0201223	-2.0983931
C	2.1479973	2.0870867	-0.1175175
C	1.2570279	3.2668538	-2.4802555
H	0.0343020	1.5069735	-2.7153449
C	2.6241525	3.3439333	-0.4886800
H	2.4748149	1.6262977	0.8104340
C	2.1872594	3.9327379	-1.6765064
H	0.9116128	3.7234954	-3.4036847
H	3.3323087	3.8641932	0.1502875
H	2.5595256	4.9102203	-1.9698970
Si	3.3486746	-1.0833868	-0.5206751
C	3.9087541	0.0715035	-1.9058099
C	3.5754535	-2.8105283	-1.2664206
C	4.4950416	-0.8666047	0.9621744
H	4.0880406	1.0990454	-1.5810971
H	4.8476603	-0.3283451	-2.3121458
H	3.1771662	0.1003537	-2.7209385
H	3.2868193	-3.6329144	-0.6069906
H	2.9865503	-2.8981193	-2.1876995
H	4.6309829	-2.9500953	-1.5331852
H	5.5372869	-1.0258703	0.6575997
H	4.4182690	0.1472265	1.3723429
H	4.2669142	-1.5730048	1.7664851

Si	0.7117139	-2.4122186	0.8569550
C	-0.0581249	-3.6146720	-0.3761528
H	-0.7887666	-3.0987317	-1.0092348
H	0.7023497	-4.0553393	-1.0303735
H	-0.5724276	-4.4317856	0.1451863
C	-0.5846803	-1.8290187	2.1021236
H	-0.8553545	-2.6756259	2.7466287
H	-0.1733065	-1.0412116	2.7448039
H	-1.4962137	-1.4451926	1.6401222
C	1.9783398	-3.3173267	1.9313623
H	2.8427120	-3.7149015	1.3933514
H	2.3485815	-2.6583341	2.7258344
H	1.4735997	-4.1622981	2.4176813
C	-0.6971388	-0.0388785	-0.5404123
C	-1.9063508	-0.1190915	-0.6803895
C	-3.3106668	-0.2661508	-0.7986167
C	-3.9231261	-1.5056791	-0.5139891
C	-4.1197241	0.8191122	-1.1966468
C	-5.3027191	-1.6487637	-0.6224180
H	-3.3040815	-2.3439401	-0.2100456
C	-5.4979797	0.6640187	-1.3054681
H	-3.6529496	1.7743995	-1.4155794
C	-6.0946658	-0.5672434	-1.0184532
H	-5.7627891	-2.6069608	-0.3991631
H	-6.1104695	1.5066099	-1.6131656
H	-7.1711573	-0.6832843	-1.1031365

aTS1^{+} : TS addition of **2a** to **A**$^{+}$ via new C-C

65

Energy = -1844.015546403

C	1.3607672	-0.6293071	-0.2028101
C	0.6302051	0.4265705	-0.2136291
C	0.4244288	1.7774860	-0.6318319
C	-0.6351542	2.1382244	-1.4860166
C	1.3585822	2.7536267	-0.2269842
C	-0.7413337	3.4424841	-1.9442053
H	-1.3523024	1.3846728	-1.7887791
C	1.2422024	4.0592327	-0.6917540
H	2.1667571	2.4775388	0.4401557
C	0.1949479	4.4054297	-1.5478082
H	-1.5516364	3.7162776	-2.6120515
H	1.9660818	4.8065613	-0.3836125
H	0.1029877	5.4262964	-1.9048541
Si	2.7531286	-0.4616475	-1.5886785
C	2.1987552	0.6605461	-2.9833640
C	3.0402987	-2.1587816	-2.3348537
C	4.2767556	0.2177192	-0.7378583
H	2.1943659	1.7208337	-2.7195037
H	2.9074117	0.5243792	-3.8103703
H	1.2039755	0.3905251	-3.3539985
H	3.3964521	-2.9110997	-1.6277639
H	2.1370215	-2.5367004	-2.8245842
H	3.8103871	-2.0372009	-3.1078561
H	5.0961582	0.2734167	-1.4654778
H	4.0990898	1.2297223	-0.3592890
H	4.6071230	-0.4075924	0.0959805
Si	1.3847215	-2.2343134	0.8455424
C	0.6761006	-3.6085597	-0.2182531
H	-0.2623363	-3.3072587	-0.6938313
H	1.3672536	-3.9268701	-1.0028148
H	0.4689900	-4.4771633	0.4190004
C	0.4260379	-2.0872510	2.4499476

H	0.6462965	-2.9957991	3.0263285
H	0.7564646	-1.2329881	3.0475928
H	-0.6542861	-2.0187450	2.3190679
C	3.1706731	-2.5588922	1.3237353
H	3.8458512	-2.6906140	0.4745500
H	3.5627387	-1.7523369	1.9534379
H	3.2004885	-3.4823269	1.9156026
C	-1.1117537	0.3888839	1.1833245
C	-1.8993560	-0.2932837	0.5156557
C	-2.7640873	-1.0898794	-0.2615016
C	-2.8594644	-2.4792403	-0.0215510
C	-3.5626301	-0.5046533	-1.2701275
C	-3.7164771	-3.2596429	-0.7863848
H	-2.2659236	-2.9261199	0.7681454
C	-4.4157877	-1.2958256	-2.0279180
H	-3.5133497	0.5664121	-1.4356743
C	-4.4909269	-2.6723577	-1.7917717
H	-3.7867578	-4.3262473	-0.5983164
H	-5.0277128	-0.8423844	-2.8011095
H	-5.1598237	-3.2865595	-2.3866639
Si	-0.7540266	1.5143247	2.6365603
C	-1.3651937	3.2169699	2.1428532
C	1.0846173	1.5144055	3.0523236
C	-1.7600754	0.8306578	4.0637249
H	-0.8364142	3.6089499	1.2692964
H	-2.4359609	3.1907104	1.9138706
H	-1.2103869	3.9155016	2.9745131
H	1.6974725	1.0776334	2.2585889
H	1.4364356	2.5371008	3.2272385
H	1.2703169	0.9407601	3.9669265
H	-1.6215625	1.4613088	4.9506346
H	-2.8279793	0.8122681	3.8210988
H	-1.4473255	-0.1871011	4.3200757

aTS2$^+$: Me_3Si^+ transfer from cation **aA$^+$** to **2a**

91

Energy = -2561.437453696

C	0.2605944	-3.1804391	0.4524500
C	-0.0147187	-2.1630992	-0.4163512
C	0.8033432	-1.7848264	-1.6105745
C	0.2238935	-1.7025973	-2.8844719
C	2.1530030	-1.4478338	-1.4510368
C	0.9906117	-1.3196714	-3.9820159
H	-0.8253074	-1.9544317	-3.0129372
C	2.9157368	-1.0455922	-2.5464800
H	2.5985890	-1.4982390	-0.4631179
C	2.3371911	-0.9842755	-3.8145931
H	0.5385064	-1.2791028	-4.9685243
H	3.9599495	-0.7822191	-2.4083434
H	2.9305624	-0.6751822	-4.6698212
Si	1.5446185	-4.5278962	0.0121026
C	1.8642780	-4.7151399	-1.8360814
C	0.8375010	-6.2019617	0.5257000
C	3.1620728	-4.2065694	0.9256278
H	2.4995850	-3.9359184	-2.2627084
H	2.3636045	-5.6814915	-1.9857087
H	0.9257518	-4.7364997	-2.4011511
H	0.5760629	-6.2921904	1.5826641
H	-0.0534517	-6.4400833	-0.0669255
H	1.5922359	-6.9675102	0.3045573
H	3.8473540	-5.0480334	0.7642851
H	3.6566926	-3.3006215	0.5586941

H	3.0124666	-4.0972962	2.0043814
Si	-0.7229343	-3.4256552	2.0677280
C	-2.1435279	-4.6166142	1.7405481
H	-2.8198876	-4.2012326	0.9838152
H	-1.8009780	-5.5926727	1.3857131
H	-2.7204577	-4.7719297	2.6605482
C	-1.4578426	-1.8524784	2.8152875
H	-1.8282145	-2.1241780	3.8126818
H	-0.7213902	-1.0550798	2.9562966
H	-2.2996371	-1.4465347	2.2513541
C	0.4472907	-4.0590984	3.4040040
H	0.9704177	-4.9847734	3.1532692
H	1.2020181	-3.2989059	3.6395751
H	-0.1311794	-4.2401062	4.3184853
C	-1.1527694	-1.3018633	-0.2736788
C	-2.2275536	-0.7133782	-0.4050620
C	-3.4956661	-0.0928848	-0.5273571
C	-4.5034304	-0.3543162	0.4240913
C	-3.7513127	0.8015782	-1.5879311
C	-5.7372140	0.2758910	0.3156763
H	-4.3093814	-1.0489721	1.2344268
C	-4.9871609	1.4299494	-1.6803175
H	-2.9800371	0.9963820	-2.3249240
C	-5.9793138	1.1714124	-0.7299467
H	-6.5115014	0.0708686	1.0479036
H	-5.1800264	2.1192472	-2.4961452
H	-6.9432861	1.6646432	-0.8068506
Si	-0.4220225	1.0668369	0.2389953
C	-0.2290075	1.5407110	-1.5543197
C	1.0404756	0.4075186	1.1869058
C	-1.7517495	1.7062915	1.3755018
H	-0.6078261	0.7582638	-2.2154190
H	-0.7410967	2.4808428	-1.7678995
H	0.8353208	1.6702033	-1.7697927
H	0.8790442	-0.6229265	1.5117605
H	1.9502413	0.4413126	0.5859223
H	1.1811481	1.0306310	2.0760124
H	-1.2826421	2.3865622	2.0925399
H	-2.5568769	2.2291137	0.8579834
H	-2.1832100	0.8708368	1.9363574
C	0.3610240	3.9687848	0.4054000
C	1.4957593	3.5010991	0.4362251
C	2.8174140	2.9777794	0.4493233
C	3.5022557	2.8006019	1.6679318
C	3.4455465	2.6071886	-0.7566330
C	4.7800817	2.2515057	1.6754974
H	3.0207320	3.0911831	2.5961479
C	4.7230071	2.0565875	-0.7367627
H	2.9273306	2.7591080	-1.6978816
C	5.3907835	1.8731251	0.4766050
H	5.3016945	2.1170678	2.6181289
H	5.2015167	1.7751208	-1.6698014
H	6.3876357	1.4429955	0.4878819
Si	-1.0398421	5.1810851	0.2929044
C	-0.2456919	6.8085994	-0.2029965
C	-1.8704804	5.3426120	1.9679493
C	-2.2624983	4.6396168	-1.0286674
H	0.2748240	6.7175806	-1.1625618
H	-1.0123771	7.5869704	-0.3018165
H	0.4791796	7.1373147	0.5496320
H	-2.4263999	4.4443794	2.2537973
H	-1.1354336	5.5550260	2.7521488

H	-2.5809903	6.1778970	1.9380878
H	-3.0643881	5.3847270	-1.1003160
H	-1.7814742	4.5778062	-2.0116024
H	-2.7310856	3.6735686	-0.8163832

A⁺ : stable cation $(Me_3Si)_2CCPh^+$

39

Energy = -1126.621139008

C	0.7321589	-0.2092396	2.5106150
C	-0.4503565	-0.2751951	2.0875472
C	-1.7554984	-0.3476876	1.6202983
C	-2.4922770	-1.5524147	1.7676890
C	-2.3463626	0.7839818	0.9989073
C	-3.7937639	-1.6134906	1.2994754
H	-2.0252865	-2.4071212	2.2450009
C	-3.6492287	0.7019243	0.5378114
H	-1.7689168	1.6961736	0.8944906
C	-4.3684728	-0.4913286	0.6878316
H	-4.3671923	-2.5279596	1.4062042
H	-4.1120489	1.5595560	0.0616203
H	-5.3896500	-0.5474088	0.3235686
Si	2.1750394	-0.6628561	1.2770399
C	1.3944790	-1.6169433	-0.1279448
C	3.4042326	-1.7020497	2.2253023
C	2.8735188	0.9754060	0.7100957
H	0.6476832	-1.0151651	-0.6579028
H	2.1668030	-1.9034075	-0.8516818
H	0.9126897	-2.5338885	0.2287497
H	3.8584485	-1.1584823	3.0594909
H	2.9464574	-2.6189720	2.6104176
H	4.2098092	-1.9922426	1.5389294
H	3.6566900	0.7848675	-0.0346278
H	2.1012019	1.5918442	0.2379747
H	3.3196422	1.5444740	1.5305513
Si	1.0356304	0.3390680	4.3573448
C	1.3757047	-1.2430458	5.2926222
H	1.4961953	-1.0038992	6.3567848
H	0.5412718	-1.9458258	5.1966631
H	2.2892256	-1.7391470	4.9529413
C	-0.5391344	1.1688190	4.9278137
H	-0.4244954	1.5040459	5.9654943
H	-0.7733898	2.0467601	4.3157617
H	-1.3921397	0.4818930	4.8913189
C	2.4823690	1.5209199	4.3548094
H	3.4086765	1.0515271	4.0095275
H	2.2831724	2.4023615	3.7368964
H	2.6466800	1.8632859	5.3844435

Ba⁺ : carbocation $(Me_3Si)_2CC(Ph)CH_2CHPr^+$

54

Energy = -1323.272391423

C	-0.6899598	-0.7845109	0.0700803
C	0.5810181	-0.6471788	0.5465377
C	1.1380321	0.5375579	1.2418981
C	1.7463579	0.4347199	2.5012170
C	1.0570778	1.7958489	0.6253525
C	2.2381014	1.5711414	3.1395030
H	1.8080501	-0.5279172	3.0005063
C	1.5634935	2.9284303	1.2564285
H	0.6024706	1.8773638	-0.3565847
C	2.1515969	2.8188327	2.5179628
H	2.6895447	1.4822737	4.1227417

H	1.4980973	3.8945782	0.7658247
H	2.5431243	3.7014371	3.0142766
Si	-2.0642304	0.4108055	0.7480181
C	-1.6501376	1.1694909	2.4180731
C	-3.6349636	-0.5776282	1.0818092
C	-2.3873215	1.7402747	-0.5450312
H	-0.9293161	1.9882452	2.3766355
H	-2.5893248	1.5619059	2.8295753
H	-1.2753895	0.4150155	3.1181015
H	-4.0233777	-1.1666587	0.2493099
H	-3.4950223	-1.2432696	1.9408249
H	-4.4069464	0.1519757	1.3591866
H	-1.5607536	2.4545950	-0.6029827
H	-2.5544928	1.3259938	-1.5433194
H	-3.2888159	2.2929408	-0.2522790
Si	-1.3629317	-2.2087789	-1.0331077
C	-2.0462504	-3.5054757	0.1473085
H	-2.4872610	-4.3167250	-0.4452389
H	-1.2413646	-3.9376674	0.7533863
H	-2.8115646	-3.1218217	0.8242540
C	-0.1457929	-3.0949362	-2.1718539
H	0.6736970	-3.6204355	-1.6728205
H	-0.7463963	-3.8597578	-2.6828094
H	0.2640654	-2.4479536	-2.9556649
C	-2.6400386	-1.4870092	-2.2094105
H	-3.4601505	-0.9461542	-1.7344545
H	-2.1528950	-0.8107371	-2.9216945
H	-3.0732993	-2.3133076	-2.7864979
C	2.0904326	-1.3421251	-0.6878584
C	1.5851449	-1.9167198	0.5165440
H	1.6014083	-1.6155822	-1.6176079
C	3.2293804	-0.4151169	-0.7697518
H	1.0289746	-2.8448633	0.4381462
H	2.2309919	-1.8659797	1.3900692
C	3.1499666	0.6191548	-1.9038977
H	4.0862294	-1.0920157	-0.9626757
H	3.4381757	0.0404072	0.2047041
H	3.0646039	0.0910701	-2.8607927
H	2.2364690	1.2103707	-1.7832695
C	4.3683326	1.5435661	-1.9119980
H	5.2943118	0.9731808	-2.0442154
H	4.4425919	2.0966646	-0.9689173
H	4.2964233	2.2703603	-2.7263304

B^+ : cation $PrCHCH_2SiMe_3^+$

28

Energy = -605.8577983015

C	2.6224255	-1.3004338	-1.4493363
C	1.5675184	-1.7701101	-2.1965454
H	3.5530675	-1.8695151	-1.4727265
C	2.6155613	-0.0957277	-0.5984184
H	1.7630232	-2.5143241	-2.9667328
H	0.6851417	-1.1439581	-2.3312363
C	3.3079876	-0.2729578	0.7698528
H	1.6069429	0.3205005	-0.5057415
H	3.2032415	0.6400780	-1.1784604
H	2.7689202	-1.0248806	1.3572451
H	4.3198501	-0.6615064	0.6076305
C	3.3630862	1.0454787	1.5441328
H	2.3564198	1.4382224	1.7243399
H	3.9278683	1.8012044	0.9876990
H	3.8484750	0.8999467	2.5134871

Si	0.8151005	-3.0705518	-0.6838384
C	2.2149997	-4.0390462	0.0686090
H	2.8531433	-3.4296013	0.7156788
H	1.7844380	-4.8397701	0.6830596
H	2.8378422	-4.5086038	-0.7001256
C	-0.1232480	-1.9795269	0.4940136
H	0.5273648	-1.3779423	1.1343351
H	-0.8179173	-1.3167222	-0.0318659
H	-0.7170990	-2.6318069	1.1476254
C	-0.2811259	-4.0889515	-1.7956230
H	0.3001724	-4.6653755	-2.5212359
H	-0.8290501	-4.7936259	-1.1558060
H	-1.0101003	-3.4705961	-2.3275033

CB^- : anion $HCB_{11}H_5Br_6^-$

24

Energy = -15760.35350236

B	0.0000048	1.5177969	-0.7541088
B	1.4433476	0.4690192	-0.7539915
B	0.8921484	-1.2279881	-0.7541709
B	-0.8921567	-1.2279817	-0.7541732
B	-1.4433445	0.4690287	-0.7539958
B	-0.0000015	-0.0001122	0.1971138
B	-0.0000074	-1.5112901	-2.2560753
B	-1.4372403	-0.4670262	-2.2559996
B	-0.8882402	1.2224920	-2.2559933
B	0.8882600	1.2224793	-2.2559906
B	1.4372400	-0.4670451	-2.2559967
C	0.0000030	-0.0001846	-3.0357632
H	0.0000032	-0.0000823	-4.1178464
H	-0.0000205	-2.5147839	-2.8799294
H	-2.3914832	-0.7771194	-2.8798692
H	-1.4780490	2.0341042	-2.8799442
H	1.4780642	2.0340969	-2.8799390
H	2.3914752	-0.7771407	-2.8798735
Br	-0.0000123	3.2853859	0.0968242
Br	3.1234201	1.0160927	0.0978792
Br	1.9315167	-2.6576790	0.0964759
Br	-1.9314873	-2.6576838	0.0965091
Br	-3.1234349	1.0160548	0.0978717
Br	-0.0000055	-0.0004325	2.1454191

Cme^+ : $Me_2SiCH_2CHPrC(SiMe_3)=CMePh^+$

54

Energy = -1323.282867294

C	-1.7151848	0.9903052	0.3484839
C	-2.5536346	-0.0081853	1.2107560
H	-1.7408243	1.9295105	0.9118183
C	-2.4037324	1.3115510	-0.9947637
H	-2.2190097	0.0598130	2.2543112
H	-3.6118984	0.2799093	1.1870047
C	-1.6321941	2.3325438	-1.8371581
H	-3.4004365	1.7099962	-0.7638148
H	-2.5757137	0.4105605	-1.5968703
H	-1.4851658	3.2447349	-1.2449241
H	-0.6313876	1.9405686	-2.0566098
C	-2.3542717	2.6732191	-3.1435168
H	-3.3462774	3.0948819	-2.9453442
H	-2.4876103	1.7777816	-3.7620030
H	-1.7871388	3.4043790	-3.7283511
Si	-2.3982585	-1.7931613	0.7462631
C	-1.1113688	-2.8161221	1.5783286

H	-1.5297091	-3.1093927	2.5523760
H	-0.8842622	-3.7328348	1.0273599
H	-0.1973533	-2.2466903	1.7642152
C	-3.8513868	-2.6749504	0.0255570
H	-4.5100204	-2.9579964	0.8591063
H	-4.4235742	-2.0322304	-0.6496011
H	-3.5544429	-3.5902542	-0.4948600
C	-0.7708646	-1.3984227	-1.2743666
H	-0.4403239	-2.4388971	-1.2465922
H	-0.8879373	-1.1133496	-2.3282426
H	-1.8611692	-1.3457666	-0.9724386
C	-0.2450827	0.5645541	0.2527324
C	0.1511653	-0.4765899	-0.5167230
C	1.5809588	-0.7888593	-0.7786045
C	2.3933086	0.1934182	-1.3640793
C	2.1367796	-2.0459196	-0.4970292
C	3.7392222	-0.0587479	-1.6254339
H	1.9560414	1.1540842	-1.6188313
C	3.4848848	-2.2945837	-0.7453026
H	1.5240697	-2.8263582	-0.0533210
C	4.2904460	-1.3009054	-1.3076648
H	4.3553537	0.7126140	-2.0779929
H	3.9085189	-3.2637171	-0.4988695
H	5.3397345	-1.4986658	-1.5051038
Si	0.9209878	1.5268400	1.4158213
C	-0.0574880	1.8535655	3.0012701
C	1.3860754	3.1846981	0.6550222
C	2.4433321	0.5581941	1.9509622
H	-0.3271885	0.9204555	3.5104517
H	0.5806008	2.4229379	3.6887112
H	-0.9711874	2.4379804	2.8491450
H	2.0101472	3.0652124	-0.2366284
H	0.4953631	3.7559627	0.3692671
H	1.9480856	3.7840072	1.3818377
H	2.8872698	1.0718552	2.8134851
H	2.1738533	-0.4545996	2.2722274
H	3.2081518	0.4725894	1.1757475

Cm⁺ : $Me_3SiCH_2CHPrC(SiMe_2)=CMePh^+$

54

Energy = -1323.281834874

C	-0.8227448	-0.7915177	-0.2362425
C	-2.0086869	-0.2013583	-0.6284260
C	-2.2846184	1.2387170	-0.4568335
C	-2.2200887	1.8196467	0.8213543
C	-2.6686651	2.0367318	-1.5485214
C	-2.5055400	3.1701707	0.9982570
H	-1.9826664	1.1954854	1.6757277
C	-2.9165824	3.3953005	-1.3734346
H	-2.7242420	1.6047165	-2.5431319
C	-2.8365491	3.9655945	-0.1010135
H	-2.4678039	3.6029181	1.9933572
H	-3.1771315	4.0087599	-2.2304532
H	-3.0413961	5.0231508	0.0345976
Si	-0.7258593	-2.5818630	0.0132814
C	-3.1237663	-0.9545765	-1.2949318
C	-2.1478060	-3.7605079	0.0879207
C	0.8935842	-3.3514090	0.4544912
H	-3.3938183	-0.4305970	-2.2200475
H	-2.8585270	-1.9767637	-1.5661177
H	-4.0248338	-0.9476133	-0.6697250
H	-1.9109796	-4.5440202	0.8168546

H	-3.0897029	-3.2781828	0.3591138
H	-2.2759327	-4.2458927	-0.8890083
H	0.9632868	-4.3353184	-0.0245424
H	1.7622647	-2.7511558	0.1795215
H	0.9144067	-3.5237483	1.5399289
Si	2.4718715	0.5819182	2.1931219
C	3.7487612	-0.2442266	1.0781573
H	3.6620681	0.0776547	0.0335725
H	4.7622676	0.0058213	1.4144649
H	3.6578981	-1.3372493	1.1035500
C	2.6991871	2.4519226	2.1828473
H	1.9267500	2.9411316	2.7887852
H	3.6739375	2.7172814	2.6103205
H	2.6515471	2.8771252	1.1746294
C	2.6697678	-0.0504771	3.9559489
H	2.5702018	-1.1419613	4.0010971
H	3.6551424	0.2123715	4.3590884
H	1.9120467	0.3827531	4.6203632
C	0.4962345	-0.0625003	0.1005162
C	0.7163954	0.0962504	1.6237071
H	1.2785649	-0.7497283	-0.2517668
C	0.7485793	1.2387121	-0.6810072
H	0.4729329	-0.8554648	2.1230263
H	0.0012186	0.8236689	2.0258574
C	0.6785796	1.0805994	-2.2015674
H	1.7598666	1.5708799	-0.4141588
H	0.0745083	2.0336050	-0.3495802
H	1.4053857	0.3196489	-2.5173946
H	-0.3083088	0.7010874	-2.4931903
C	0.9512136	2.3983514	-2.9312284
H	1.9436955	2.7889824	-2.6784079
H	0.2109468	3.1560333	-2.6492264
H	0.9058717	2.2683687	-4.0173939

C⁺ : cation $Me_3SiCH_2CHPrC(SiMe_3)=CPh^+$

54

Energy = -1323.290049603

C	-0.5992266	0.0880469	-0.0773725
C	-1.5636820	0.8801924	-0.2456603
C	-2.6995363	1.6495045	-0.4545121
C	-3.5456036	1.9747312	0.6387619
C	-3.0098152	2.1137300	-1.7604319
C	-4.6760379	2.7432062	0.4200593
H	-3.2936585	1.6145980	1.6303347
C	-4.1432128	2.8829970	-1.9587422
H	-2.3536727	1.8577183	-2.5854760
C	-4.9728232	3.1946229	-0.8729499
H	-5.3306586	2.9954426	1.2472839
H	-4.3898064	3.2423998	-2.9519357
H	-5.8613195	3.7965851	-1.0366605
Si	-1.3179601	-1.7389275	-0.3696982
C	-3.1666050	-1.7010046	-0.6205210
C	-0.8349382	-2.6507791	1.1897282
C	-0.4042599	-2.3244734	-1.8905081
H	-3.4531730	-1.1345410	-1.5123938
H	-3.5163698	-2.7318522	-0.7554182
H	-3.6922588	-1.2828960	0.2442330
H	0.2486898	-2.6676938	1.3421119
H	-1.3109536	-2.2210018	2.0765506
H	-1.1749682	-3.6900369	1.0942206
H	-0.7095808	-3.3565965	-2.1042991
H	-0.6476302	-1.7104369	-2.7632595

H	0.6807417	-2.3165503	-1.7495230
Si	2.7502723	0.3304589	2.5452052
C	3.6686876	-1.0599876	1.6665120
H	3.8326148	-0.8406632	0.6045061
H	4.6534618	-1.2149880	2.1230725
H	3.1217100	-2.0085504	1.7349480
C	3.6905884	1.9528468	2.3831279
H	3.1279066	2.7781146	2.8356955
H	4.6523667	1.8796608	2.9057495
H	3.9007461	2.2214158	1.3421453
C	2.5317577	-0.0812201	4.3679559
H	2.0143815	-1.0388367	4.5014002
H	3.5036688	-0.1519882	4.8707556
H	1.9466807	0.6917759	4.8808018
C	0.8809432	0.1840479	0.2954802
C	0.9898472	0.4622360	1.8063789
H	1.3361823	-0.7886192	0.0790906
C	1.5720790	1.2484501	-0.5736112
H	0.3716596	-0.2622726	2.3519029
H	0.5640803	1.4509961	2.0213327
C	1.5390382	0.9648107	-2.0772416
H	2.6167290	1.3024862	-0.2436483
H	1.1204716	2.2256475	-0.3590541
H	2.0068732	-0.0090514	-2.2707940
H	0.4975257	0.8837078	-2.4138970
C	2.2548508	2.0512139	-2.8851225
H	3.3064754	2.1310224	-2.5876043
H	1.7862402	3.0292039	-2.7263296
H	2.2232138	1.8303236	-3.9565721

$Me_3Si^+CB^-$: contact ion pair

37

Energy = -16169.58060021

B	0.3490989	-2.4942354	1.6359211
B	1.3527043	-1.8079490	0.3125768
B	2.8035004	-2.8496642	0.1136420
B	2.6907720	-4.1859524	1.3063816
B	1.1738739	-3.9663339	2.2470338
B	2.1184065	-2.5539239	1.7214191
B	2.2465708	-4.4587147	-0.3821097
B	1.2513430	-5.1412228	0.9272875
B	-0.1844572	-4.1067164	1.1254875
B	-0.0745498	-2.7856241	-0.0576400
B	1.4280409	-3.0032681	-0.9895282
C	0.5504134	-4.3309321	-0.3920456
H	0.0148496	-4.9322113	-1.1152037
H	2.8084893	-5.1761459	-1.1318755
H	1.1562738	-6.3113166	1.0470644
H	-1.2314440	-4.5913916	1.3735147
H	-1.0456583	-2.3887607	-0.5981811
H	1.4437163	-2.7492662	-2.1418340
Br	-0.7141178	-1.3516734	2.8123915
Br	1.3699193	0.1038566	-0.1058046
Br	4.5258511	-2.1105997	-0.4403462
Br	4.2872114	-4.9719152	2.1158132
Br	1.0505478	-4.5028763	4.1226806
Br	3.1626769	-1.6574556	3.1976664
Si	3.5007213	0.7584226	3.1846224
C	4.4802208	0.7671859	4.7674711
H	4.7516833	1.8098026	4.9811869
H	3.8973953	0.3918178	5.6140526
H	5.4055923	0.1895267	4.6823733

C	4.5103643	1.1345996	1.6784324
H	3.9670250	0.9337366	0.7532239
H	4.7643373	2.2030427	1.7110398
H	5.4404984	0.5590415	1.6805322
C	1.8222031	1.5246078	3.3423122
H	1.9647073	2.6094740	3.4416982
H	1.1943242	1.3342979	2.4699435
H	1.3113860	1.1607413	4.2385018

Me_3Si^+ : silylium

13

Energy = -409.1603585195

Si	-0.0001944	0.0000235	0.0003575
C	-0.0178795	1.8305747	0.0001964
H	0.5302808	2.1919592	0.8809609
H	0.5279935	2.1915046	-0.8822047
C	-1.5764051	-0.9309536	0.0001751
H	-2.1639309	-0.6369400	0.8805820
H	-2.1617000	-0.6394694	-0.8826073
C	1.5941513	-0.8995414	0.0000052
H	1.6334385	-1.5549969	0.8807174
H	1.6340774	-1.5526624	-0.8824435
H	-1.4274837	-2.0127480	0.0017942
H	2.4566090	-0.2297702	0.0011301
H	-1.0289589	2.2430211	0.0013370

$PhClSiMe_3^+$: adduct of PhCl and Me_3Si^+ via Cl

25

Energy = -1101.224552342

C	-0.0772495	1.9602116	0.3216614
C	-1.4209476	1.6161343	0.3366539
C	-1.7271210	0.2653371	0.5151959
C	-0.7068230	-0.6756960	0.6660791
C	0.6324929	-0.2824008	0.6363526
C	0.9700163	1.0610664	0.4590946
Cl	0.3376387	3.6944068	0.0859093
H	-2.1975240	2.3612742	0.2103185
H	-2.7673014	-0.0431223	0.5310920
H	-0.9568389	-1.7229920	0.8010890
H	1.4242470	-1.0160172	0.7474952
H	2.0032883	1.3859772	0.4257390
Si	0.4242300	4.5328680	2.2421321
C	1.7463427	3.4925437	3.0113721
H	1.8952502	3.8476423	4.0397027
H	1.4571590	2.4385817	3.0623597
H	2.6980678	3.5853467	2.4799099
C	-1.2994523	4.2347406	2.8439351
H	-1.3751463	4.6456107	3.8594104
H	-2.0418218	4.7390626	2.2182816
H	-1.5324591	3.1671332	2.8992667
C	0.8726188	6.2772315	1.8166691
H	1.8357402	6.3340764	1.3001647
H	0.1025721	6.7564609	1.2043022
H	0.9583071	6.8415071	2.7544541

PhCl : chlorobenzene

12

Energy = -692.0290667825

C	-0.0000005	1.3211246	-0.0000177
C	-1.2177146	0.6431364	0.0000333
C	-1.2081838	-0.7527539	0.0000270
C	0.0000001	-1.4525622	-0.0000320

C	1.2081834	-0.7527519	0.0000157
C	1.2177133	0.6431378	-0.0000109
Cl	0.0000002	3.0763182	-0.0001001
H	-2.1505432	1.1965485	0.0001123
H	-2.1519401	-1.2902284	0.0000058
H	0.0000049	-2.5382810	-0.0001827
H	2.1519359	-1.2902338	0.0001592
H	2.1505446	1.1965457	-0.0000099

$SiMe_4$: tetramethylsilane

17

Energy = -449.3300364288

Si	0.0000020	-0.0000000	-0.0000000
C	1.0891168	-1.0891173	1.0891160
H	1.7350441	-0.4823466	1.7356290
H	0.4823457	-1.7356264	1.7350471
H	1.7356285	-1.7350435	0.4823436
C	-1.0891173	1.0891149	1.0891148
H	-0.4823482	1.7356256	1.7350460
H	-1.7356233	1.7350480	0.4823425
H	-1.7350476	0.4823432	1.7356231
C	1.0891169	1.0891173	-1.0891159
H	1.7350443	0.4823466	-1.7356288
H	0.4823458	1.7356263	-1.7350472
H	1.7356284	1.7350436	-0.4823435
C	-1.0891173	-1.0891149	-1.0891148
H	-0.4823482	-1.7356255	-1.7350461
H	-1.7356233	-1.7350480	-0.4823425
H	-1.7350477	-0.4823431	-1.7356231

TS0a : Me_3Si^+ transfer from $Me_3Si^+CB^-$ to **1i**

52

Energy = -16366.23845083

B	-1.4140571	-1.0741745	-1.2827832
B	0.3015317	-0.8365633	-1.7295163
B	1.2996509	-1.8659569	-0.6607455
B	0.2019963	-2.7443367	0.4452007
B	-1.4779361	-2.2546013	0.0596774
B	-0.1550538	-1.0596940	-0.0245943
B	0.8710860	-3.5546684	-0.9777509
B	-0.8348159	-3.7922061	-0.5340816
B	-1.8274040	-2.7675406	-1.5967188
B	-0.7354357	-1.8961125	-2.6950081
B	0.9323623	-2.3830624	-2.3124873
C	-0.3709051	-3.4603992	-2.1365810
H	-0.4428300	-4.2698398	-2.8512731
H	1.6187593	-4.4681751	-0.9613191
H	-1.2192409	-4.8638983	-0.2221114
H	-2.8693784	-3.1592186	-1.9899131
H	-1.0497025	-1.7073076	-3.8172760
H	1.7201972	-2.5154920	-3.1817925
Br	-2.7302101	0.3674875	-1.4516968
Br	0.9671511	0.8555804	-2.4655431
Br	3.1081160	-1.3365533	-0.1215389
Br	0.7442180	-3.2259632	2.2630599
Br	-2.8693563	-2.1734675	1.4339398
Br	-0.0664329	0.3370571	1.3744004
Si	0.8331870	3.0641549	0.9924740
C	0.5494403	3.1488706	2.8396838
H	1.3515060	3.7427475	3.2918055
H	-0.4116434	3.6175045	3.0717373
H	0.5719873	2.1613162	3.3089959

C	2.5193407	2.4969220	0.4326910
H	2.4972783	1.4315071	0.1865214
H	2.8567146	3.0454348	-0.4508911
H	3.2369040	2.6535889	1.2451575
C	-0.5506951	3.3550381	-0.2143200
H	-0.2324093	4.0686134	-0.9809497
H	-0.8446080	2.4310991	-0.7171331
H	-1.4255688	3.7604869	0.3033142
C	0.6893849	5.9144837	1.3395936
C	1.8105064	5.3320220	0.8542563
H	0.5471093	5.9485079	2.4198911
C	-0.3610738	6.5849808	0.5255783
H	2.6113844	5.0214969	1.5198119
H	2.0273259	5.3445598	-0.2113690
C	-0.3588129	8.1096524	0.8020021
H	-1.3490750	6.1910480	0.7972712
H	-0.2011504	6.4036932	-0.5421415
H	-0.4981248	8.2810086	1.8760284
H	0.6238575	8.5166483	0.5371039
C	-1.4565884	8.8237903	0.0090783
H	-2.4471299	8.4440913	0.2830878
H	-1.3218994	8.6745597	-1.0679319
H	-1.4372894	9.9001143	0.2078233

TS0 : Me_3Si^+ transfer from $Me_3Si^+CB^-$ to **2a**

63

Energy = -16886.98440535

B	-1.8937673	-1.8089056	0.4687352
B	-1.4897768	-1.0891353	-1.1224540
B	-0.6867783	-3.0839269	0.8201207
B	-0.1950971	-1.5843132	-0.0127917
B	-2.2879256	-3.5165359	0.2073043
B	-2.7802799	-2.2932077	-0.9833745
Br	-2.7366402	-0.7696323	1.8967682
B	-0.0310555	-1.9188697	-1.7529200
B	-1.6381730	-2.3605512	-2.3454422
Br	-1.9171926	0.7715500	-1.5687314
B	0.4642970	-3.1519252	-0.5530175
B	-0.8400066	-4.3394546	-0.4170004
Br	-0.1279920	-3.4917404	2.6503822
Br	1.0543845	-0.3892446	0.9806715
C	-2.0484810	-3.7522934	-1.4590421
H	-3.1072764	-4.1275234	0.7977611
H	-3.9252173	-2.0902450	-1.1866844
B	-0.4406898	-3.6256420	-1.9963050
Br	1.2745190	-1.0054929	-2.8896449
H	-2.0292859	-2.2023234	-3.4480337
Br	2.3418753	-3.6390538	-0.2964273
H	-0.6971720	-5.4977408	-0.2401809
Si	0.9858078	2.2862657	0.8034109
H	-2.6778500	-4.4827387	-1.9505234
H	-0.0339285	-4.3090604	-2.8685682
C	2.3246366	2.2804580	2.1062166
C	1.4931131	2.2314222	-0.9881229
C	-0.7191269	2.4241206	1.5325745
H	2.4460892	3.2791220	2.5320752
H	2.0350713	1.6101653	2.9226727
H	3.2859444	1.9487336	1.7050490
H	0.6528156	1.9381800	-1.6217980
H	1.8738278	3.2013480	-1.3135870
H	2.2808288	1.4816719	-1.1057097
H	-0.7957372	3.3610887	2.0925190

H	-1.5109795	2.3791016	0.7847397
H	-0.8603651	1.5915775	2.2278507
C	0.1629799	4.9846097	0.0994051
C	1.2956827	5.0246676	0.5783072
C	-1.1331918	4.9086086	-0.4733624
Si	2.9521423	5.7815992	0.9555923
C	-2.2707527	5.2453899	0.2873916
C	-1.2898726	4.4799409	-1.8068821
C	3.1335922	6.0127456	2.8094911
C	4.3069317	4.7020996	0.2351456
C	2.9313055	7.4529591	0.0986722
C	-3.5374192	5.1402830	-0.2755285
H	-2.1470123	5.5781376	1.3127458
C	-2.5622791	4.3783582	-2.3584923
H	-0.4130381	4.2279779	-2.3931521
H	2.3102972	6.6184701	3.2045507
H	3.1574892	5.0703539	3.3659887
H	4.0714296	6.5428697	3.0168755
H	4.1593139	4.5512751	-0.8400420
H	5.2757970	5.1975657	0.3728114
H	4.3647634	3.7205611	0.7137976
H	3.8779781	7.9785953	0.2748794
H	2.8004112	7.3377155	-0.9827701
H	2.1167771	8.0798933	0.4774079
C	-3.6861700	4.7027695	-1.5947683
H	-4.4114973	5.3960665	0.3152920
H	-2.6783657	4.0411903	-3.3837125
H	-4.6781499	4.6160869	-2.0275408

TS1a$^+$: alkene **1i** addition to carbocation **A$^+$**

54

Energy = -1323.265311877

C	-1.1525024	-0.1330437	0.1507768
C	0.1054539	-0.0910229	0.4850762
C	1.2717941	0.7536821	0.3785080
C	1.9306105	1.2772215	1.5051240
C	1.7229956	1.0954963	-0.9106445
C	3.0044342	2.1442139	1.3408301
H	1.5832155	1.0273564	2.5022690
C	2.8039073	1.9568677	-1.0661582
H	1.2153237	0.6846716	-1.7768803
C	3.4454981	2.4818343	0.0576826
H	3.4989736	2.5587756	2.2133086
H	3.1443701	2.2191461	-2.0627265
H	4.2894607	3.1534606	-0.0647056
Si	-1.8702453	1.6472729	-0.2449521
C	-0.8304620	3.0327057	0.4731197
C	-3.5498529	1.7783023	0.5867579
C	-1.9565622	1.8000189	-2.1121765
H	0.1138017	3.1963906	-0.0516226
H	-1.4257007	3.9512953	0.3867530
H	-0.6164503	2.8774678	1.5357095
H	-4.2791266	1.0246556	0.2827743
H	-3.4433744	1.7388567	1.6763809
H	-3.9628872	2.7627325	0.3314237
H	-2.3941131	2.7742608	-2.3645766
H	-0.9552141	1.7597510	-2.5539576
H	-2.5685217	1.0229657	-2.5771580
Si	-2.4180845	-1.5723447	0.0922039
C	-3.2866022	-1.6037124	1.7556031
H	-3.8307507	-0.6778384	1.9602577
H	-4.0069153	-2.4308985	1.7659067

H -2.5784175 -1.7679818 2.5755499

C -1.6287402 -3.2443534 -0.2474315

H -1.0706484 -3.6649018 0.5936548

H -2.4577625 -3.9331257 -0.4572027

H -0.9895013 -3.2320751 -1.1364992

C -3.5981896 -1.2839054 -1.3383985

H -4.1452046 -0.3394765 -1.3053775

H -3.0667907 -1.3410504 -2.2950381

H -4.3371424 -2.0950205 -1.3254984

C 1.4114854 -2.2613483 0.6464149

C 0.6985786 -1.6813986 1.6647447

H 0.9032467 -2.9778352 0.0053216

C 2.8316269 -1.9822084 0.3266020

H -0.2721395 -2.0715741 1.9456759

H 1.2043364 -1.0831713 2.4161682

C 3.1582479 -2.0619174 -1.1744106

H 3.4214327 -2.7495144 0.8579501

H 3.1421436 -1.0244661 0.7619330

H 2.9538585 -3.0798500 -1.5276064

H 2.4874302 -1.3953972 -1.7278741

C 4.6122079 -1.6833693 -1.4607233

H 5.3023474 -2.3462254 -0.9268587

H 4.8162165 -0.6552230 -1.1404963

H 4.8307981 -1.7543995 -2.5305350

TS1^{+} : Me_3Si^{+} transfer from **A^{+}** to **1i**

54

Energy = -1323.258570716

C 1.1082360 0.0623480 -1.7825054

C -0.1114178 -0.0850146 -1.8214037

C -1.5163736 -0.2840574 -1.8775627

C -2.0763514 -1.5001490 -1.4368381

C -2.3604144 0.7342318 -2.3631464

C -3.4544860 -1.6843050 -1.4746271

H -1.4239775 -2.2874136 -1.0726769

C -3.7368843 0.5369207 -2.3994379

H -1.9263195 1.6678287 -2.7065206

C -4.2863467 -0.6678549 -1.9527601

H -3.8816193 -2.6229385 -1.1352323

H -4.3830436 1.3237515 -2.7762012

H -5.3615149 -0.8163461 -1.9811307

Si 2.8743836 0.0191135 -2.3677771

C 2.8154490 -0.8651132 -4.0226252

C 3.9232710 -0.9552016 -1.1523089

C 3.5123089 1.7700145 -2.5835398

H 2.1930975 -0.3187582 -4.7395692

H 3.8266182 -0.9463355 -4.4402506

H 2.4079078 -1.8762486 -3.9156929

H 3.9485413 -0.5082851 -0.1533501

H 3.5684765 -1.9872409 -1.0558350

H 4.9551006 -0.9922594 -1.5226744

H 4.4825968 1.7359184 -3.0943879

H 2.8295331 2.3640549 -3.2010503

H 3.6572775 2.2913512 -1.6324232

Si 0.7032645 0.4180973 1.0501857

C 1.0807157 -1.4106683 1.0690437

H 0.2082122 -1.9678127 1.4258797

H 1.3328707 -1.7708883 0.0709692

H 1.9222418 -1.6197001 1.7375706

C -1.0314138 1.0879479 0.9770377

H -1.1938388 1.7649553 1.8219275

H -1.1633450 1.6694745 0.0602845

H	-1.7929743	0.3051193	0.9992592
C	2.0142908	1.7243102	0.8020547
H	3.0274217	1.3169765	0.8400882
H	1.8544590	2.1899957	-0.1747724
H	1.9232856	2.5062874	1.5615933
C	0.1689852	-0.2074194	3.7746053
C	1.0930123	0.7397407	3.4831158
H	0.4798040	-1.2519147	3.7707212
C	-1.2420429	0.0495285	4.1699662
H	2.1439734	0.4814295	3.3776749
H	0.8482563	1.7965568	3.5594716
C	-1.4708205	-0.3823019	5.6411506
H	-1.9173113	-0.5392303	3.5344886
H	-1.4940001	1.1083672	4.0527957
H	-1.1959143	-1.4376866	5.7528831
H	-0.7999249	0.1950015	6.2872946
C	-2.9257935	-0.1689338	6.0658627
H	-3.6063929	-0.7615301	5.4444521
H	-3.2108364	0.8847664	5.9735425
H	-3.0690603	-0.4693619	7.1084432

TS2$^+$: **2a** addition to carbocation **B$^+$**

54

Energy = -1323.265775391

C	-0.7382449	-1.5945811	0.3459421
C	-1.5279949	-0.6543640	0.4645002
C	-2.3556294	0.4815049	0.5703942
C	-2.2570530	1.3224394	1.7013740
C	-3.2538660	0.8085056	-0.4698639
C	-3.0331330	2.4729910	1.7761631
H	-1.5839466	1.0521896	2.5083113
C	-4.0250204	1.9600221	-0.3791751
H	-3.3349402	0.1523796	-1.3299820
C	-3.9132779	2.7936148	0.7384027
H	-2.9592192	3.1181624	2.6457869
H	-4.7162812	2.2103170	-1.1775017
H	-4.5191573	3.6920505	0.8035023
Si	0.1291562	-3.2430742	0.3991800
C	-1.0041312	-4.4060095	1.3301872
C	1.7572817	-2.9937255	1.3019324
C	0.4164516	-3.7914096	-1.3695794
H	-1.9660558	-4.5145009	0.8179380
H	-0.5416091	-5.3978239	1.4015662
H	-1.1934521	-4.0438062	2.3462916
H	2.4531643	-2.3543679	0.7467232
H	1.6051738	-2.5593481	2.2957562
H	2.2500803	-3.9645345	1.4330372
H	0.8810796	-4.7846387	-1.3759187
H	-0.5252294	-3.8537786	-1.9252372
H	1.0855558	-3.1110341	-1.9081058
Si	2.9549389	1.6332657	0.3903578
C	3.9983879	0.4904601	-0.6638884
H	3.5719486	0.3419136	-1.6625300
H	4.9971183	0.9231251	-0.7957932
H	4.1256839	-0.4912458	-0.1933750
C	2.5954327	3.2612593	-0.4582515
H	1.9075087	3.8759688	0.1326891
H	3.5340120	3.8194221	-0.5651775
H	2.1735330	3.1416261	-1.4615560
C	3.6799285	1.8796369	2.0968516
H	3.8591444	0.9214668	2.5967085
H	4.6399701	2.4034315	2.0138521

H	3.0205685	2.4822236	2.7311294
C	0.8088370	0.0464341	-0.4290824
C	1.2175824	0.7363070	0.7552659
H	1.3388313	-0.8739390	-0.6664799
C	0.1681200	0.7311724	-1.5812283
H	1.4646223	0.0703270	1.5854072
H	0.5424499	1.5392481	1.0664997
C	-0.3627598	-0.1774889	-2.6952337
H	0.9719041	1.3626616	-2.0029366
H	-0.5935169	1.4388858	-1.2309016
H	0.4502440	-0.8237089	-3.0473247
H	-1.1324086	-0.8365331	-2.2816468
C	-0.9303229	0.6325025	-3.8627121
H	-0.1624919	1.2745114	-4.3082317
H	-1.7535657	1.2748331	-3.5299356
H	-1.3130903	-0.0301934	-4.6442010

TS3a⁺ : Me_3Si^+ transfer from **C⁺** to **1i**

69

Energy = -1519.932283762

C	1.0416990	0.6640351	-0.0931670
C	0.2941855	1.6247934	0.0186646
C	-0.5283433	2.7825985	0.1106255
C	-0.5629529	3.5415350	1.2961331
C	-1.3184628	3.1766296	-0.9870215
C	-1.3767853	4.6673187	1.3787601
H	0.0501238	3.2401078	2.1396683
C	-2.1301171	4.3026696	-0.8932567
H	-1.2867367	2.5947269	-1.9030486
C	-2.1634078	5.0480277	0.2885413
H	-1.3979158	5.2491726	2.2951743
H	-2.7354274	4.6021066	-1.7433763
H	-2.7984152	5.9260202	0.3581300
Si	-1.3199129	-0.8334385	0.3863084
C	-2.4130406	0.4727199	1.1330421
C	-0.1966853	-1.7048854	1.5978430
C	-1.1820842	-1.1333733	-1.4507381
H	-2.8515463	1.1322350	0.3801857
H	-3.2204937	0.0041165	1.7027661
H	-1.8298828	1.0853483	1.8264414
H	0.4655331	-2.4221108	1.1052002
H	0.4096101	-0.9503322	2.1087228
H	-0.7751389	-2.2355856	2.3589468
H	-2.0160160	-0.6584180	-1.9774246
H	-0.2509996	-0.7307849	-1.8534191
H	-1.2060509	-2.2078076	-1.6595085
Si	4.4345203	-1.7116141	0.8111098
C	3.7584565	-3.1995447	-0.1313440
H	3.5408402	-2.9638415	-1.1800733
H	4.4877218	-4.0184961	-0.1252381
H	2.8365995	-3.5788185	0.3272733
C	6.0115778	-1.0795609	-0.0013022
H	6.3890429	-0.1928494	0.5222747
H	6.7926538	-1.8486573	0.0407593
H	5.8681841	-0.8102822	-1.0533996
C	4.8158675	-2.2050663	2.5875806
H	3.9299743	-2.6181797	3.0850054
H	5.6058813	-2.9647226	2.6236377
H	5.1566125	-1.3419221	3.1724681
C	2.1536519	-0.2631723	-0.3465189
C	3.0845606	-0.3651463	0.8888460
H	1.7385349	-1.2587198	-0.5576022

C	2.9327803	0.1923415	-1.6064333
H	2.4800452	-0.5814208	1.7782631
H	3.5407879	0.6173038	1.0664605
C	2.1003675	0.2714130	-2.8868507
H	3.7538834	-0.5194458	-1.7526782
H	3.3902002	1.1666678	-1.3955018
H	1.6562295	-0.7117037	-3.0956755
H	1.2688103	0.9715186	-2.7371157
C	2.9355452	0.7177191	-4.0911181
H	3.7556629	0.0159783	-4.2809986
H	3.3735579	1.7068040	-3.9155715
H	2.3231187	0.7744875	-4.9967389
C	-2.9114921	-2.7777738	0.7649566
H	-3.0963025	-2.6233822	1.8256827
H	-2.1383514	-3.4926417	0.4948423
C	-3.7642155	-2.2892463	-0.1648880
H	-3.5862301	-2.5341603	-1.2118544
C	-4.9818783	-1.4792036	0.1134840
C	-6.2558586	-2.2520010	-0.3092138
H	-4.9430046	-0.5460493	-0.4654998
H	-5.0428849	-1.2215738	1.1759971
C	-7.5183972	-1.4156504	-0.0864737
H	-6.1715492	-2.5329515	-1.3656738
H	-6.3119775	-3.1827105	0.2667211
H	-8.4096716	-1.9790814	-0.3795957
H	-7.6239017	-1.1376056	0.9679431
H	-7.4877642	-0.4947380	-0.6795207

TS3$^+$: Me_3Si^+ transfer from **C$^+$** to **2a**

80

Energy = -2040.680863963

C	1.5795680	1.1095356	-0.1576709
C	1.3535996	2.3168474	-0.1863486
C	1.0833517	3.7083510	-0.2062637
C	0.9034353	4.4114899	1.0022861
C	0.9935999	4.3957111	-1.4336614
C	0.6306957	5.7745149	0.9769215
H	0.9811037	3.8794513	1.9451199
C	0.7221567	5.7592106	-1.4462621
H	1.1376814	3.8511771	-2.3613824
C	0.5373009	6.4484876	-0.2443036
H	0.4923963	6.3140436	1.9086341
H	0.6541969	6.2863818	-2.3926733
H	0.3234723	7.5128585	-0.2590473
Si	-0.8776689	0.6137307	-0.1117572
C	-1.7193822	2.2682642	-0.2764427
C	-0.5207842	-0.0437569	1.6032240
C	-0.5811774	-0.1996385	-1.7661406
H	-1.3277861	2.7932892	-1.1527625
H	-2.7937783	2.1384034	-0.4227433
H	-1.5617295	2.8998511	0.6015363
H	-0.1157426	-1.0588157	1.5625319
H	0.2002181	0.6012246	2.1123998
H	-1.4436059	-0.0708995	2.1871359
H	-1.5416781	-0.3301916	-2.2735941
H	0.0407535	0.4637924	-2.3735900
H	-0.0920968	-1.1727734	-1.6989588
Si	3.8154911	-1.9874692	1.6796180
C	2.8192503	-3.3504129	0.8393876
H	2.8180853	-3.2536392	-0.2530070
H	3.2387051	-4.3348992	1.0786305
H	1.7755242	-3.3478483	1.1748387

C	5.6085977	-2.0336625	1.1064891
H	6.1747414	-1.1957535	1.5307369
H	6.0851167	-2.9624331	1.4440604
H	5.7098870	-1.9880065	0.0167797
C	3.7645557	-2.2175306	3.5472599
H	2.7333511	-2.2253178	3.9207770
H	4.2342562	-3.1645944	3.8387151
H	4.2998167	-1.4085915	4.0592155
C	2.3582826	-0.1490755	-0.0940302
C	2.9846716	-0.3080668	1.3149885
H	1.6883904	-0.9983293	-0.2765680
C	3.4411223	-0.1478716	-1.1990357
H	2.1993581	-0.1811766	2.0698684
H	3.6981763	0.5100621	1.4781400
C	2.9100606	-0.0796358	-2.6310231
H	4.0258440	-1.0670764	-1.0757450
H	4.1235190	0.6899818	-1.0096755
H	2.2651234	-0.9476100	-2.8208702
H	2.2790582	0.8109405	-2.7452571
C	4.0412350	-0.0439474	-3.6629852
H	4.6762616	-0.9334142	-3.5797524
H	4.6764797	0.8365762	-3.5140239
H	3.6442339	-0.0069122	-4.6824005
C	-3.1971835	-0.5919230	0.2128665
C	-2.6982252	-1.6608242	-0.1471949
C	-2.0737408	-2.8635284	-0.5576165
C	-1.3431177	-3.6327620	0.3721208
C	-2.1752485	-3.2984713	-1.8955937
C	-0.7169525	-4.8042806	-0.0372499
H	-1.2843185	-3.3053775	1.4050406
C	-1.5434776	-4.4710065	-2.2923775
H	-2.7457555	-2.7093677	-2.6059279
C	-0.8118693	-5.2220571	-1.3674717
H	-0.1558142	-5.3949855	0.6796462
H	-1.6213343	-4.8024706	-3.3229977
H	-0.3200494	-6.1370668	-1.6830463
Si	-4.6325107	0.3494702	0.9531010
C	-5.8195570	-0.9717031	1.5567301
C	-5.4368030	1.3878612	-0.3870152
C	-4.0288482	1.3906979	2.3921878
H	-5.3441734	-1.6156918	2.3044271
H	-6.6983854	-0.5040306	2.0173333
H	-6.1623179	-1.6014419	0.7286780
H	-4.8260665	2.2387841	-0.7057220
H	-5.6632176	0.7795030	-1.2693526
H	-6.3823947	1.7909064	-0.0035861
H	-4.8770047	1.9419134	2.8162788
H	-3.6118800	0.7629456	3.1876588
H	-3.2714192	2.1242851	2.1010899

TSAm$^+$: Si-to-C methyl shift in **A$^+$**

39

Energy = -1126.571560063

C	0.9094522	0.1154490	0.0149388
C	-0.1574167	-0.6553602	-0.0372977
C	-1.6084058	-0.5507666	-0.0331279
C	-2.3875870	-0.9503572	-1.1300515
C	-2.2168173	0.0422604	1.0865289
C	-3.7622168	-0.7392130	-1.1110289
H	-1.9170404	-1.4001534	-1.9990136
C	-3.5943738	0.2440287	1.0971700
H	-1.6073432	0.3398572	1.9340031

C	-4.3663824	-0.1453022	0.0010170
H	-4.3631597	-1.0345554	-1.9651572
H	-4.0637669	0.7028556	1.9615998
H	-5.4404066	0.0124610	0.0122673
Si	1.1295233	1.9941795	-0.0254357
C	-0.4270902	2.7856839	-0.7012086
C	2.6076648	2.3003157	-1.1377015
C	1.4737359	2.5043733	1.7466524
H	-1.2882634	2.6138044	-0.0473396
H	-0.2831980	3.8691858	-0.7905995
H	-0.6713614	2.3962253	-1.6958272
H	3.5068743	1.8069222	-0.7504569
H	2.4253386	1.9321637	-2.1537174
H	2.8180017	3.3743093	-1.2028194
H	1.6549738	3.5848056	1.7962702
H	0.6239262	2.2753570	2.3992875
H	2.3586749	1.9964307	2.1461155
Si	1.8494162	-1.4446650	0.0550598
C	2.7446248	-1.9030965	-1.5004946
H	3.0558527	-2.9531129	-1.4794654
H	2.1193598	-1.7331693	-2.3822040
H	3.6423146	-1.2802182	-1.5922037
C	0.1131935	-2.5799653	-0.0144509
H	0.9170473	-3.3473141	-0.0188635
H	-0.4713048	-2.7659486	0.8835675
H	-0.4482278	-2.7648482	-0.9274643
C	2.6172663	-1.9206193	1.6729473
H	3.4981378	-1.2909795	1.8465319
H	1.9204915	-1.7700182	2.5030974
H	2.9402170	-2.9671197	1.6624085

TSCme$^+$: Si-to-C 1,5-methyl shift in **C$^+$**

54

Energy = -1323.253303838

C	-0.4696774	0.6326574	0.3480068
C	0.1694933	-0.3210951	-0.2817878
C	1.5207509	-0.6789856	-0.6680301
C	2.4020114	-1.2740152	0.2503814
C	1.9797006	-0.3620774	-1.9600429
C	3.7244283	-1.5183439	-0.1105529
H	2.0591092	-1.5215555	1.2465754
C	3.2988005	-0.6130886	-2.3153747
H	1.3036841	0.0999305	-2.6738710
C	4.1740784	-1.1908862	-1.3904701
H	4.4030477	-1.9645672	0.6092197
H	3.6479046	-0.3529699	-3.3094331
H	5.2049042	-1.3857277	-1.6695267
Si	0.6318010	1.9095948	1.3054807
C	2.2282537	2.2819373	0.3983926
C	0.9285951	1.1308783	2.9878368
C	-0.3475226	3.4995091	1.4880359
H	2.0416870	2.5477466	-0.6478083
H	2.6981813	3.1475295	0.8821515
H	2.9435824	1.4559616	0.4208398
H	-0.0130405	0.8380275	3.4660669
H	1.5659969	0.2433261	2.9137190
H	1.4296948	1.8485038	3.6486732
H	0.2648308	4.1998345	2.0704942
H	-0.5567594	3.9668675	0.5209675
H	-1.2934065	3.3663631	2.0222128
Si	-1.6201599	-2.1185649	0.4833945
C	-0.3329401	-2.7517635	1.6765750

H	-0.8774148	-3.2843025	2.4675537
H	0.3545420	-3.4667718	1.2132640
H	0.2418729	-1.9546561	2.1523097
C	-2.7672895	-3.5237286	0.0264817
H	-3.5495478	-3.2008271	-0.6691178
H	-2.2195805	-4.3559844	-0.4300487
H	-3.2552164	-3.9026314	0.9325651
C	-0.8857647	-1.6511061	-1.3410125
H	-0.2213857	-2.4838730	-1.5813383
H	-0.6230251	-0.8275737	-2.0162600
H	-1.9153734	-1.8302879	-1.7033295
C	-1.9883288	0.7295574	0.5014071
C	-2.5290611	-0.5953172	1.0921569
H	-2.2035552	1.5385873	1.2042178
C	-2.6830577	1.0907965	-0.8282150
H	-2.4236774	-0.5775359	2.1843315
H	-3.6010047	-0.6817043	0.8790276
C	-2.1751252	2.3762293	-1.4860991
H	-3.7539711	1.1907980	-0.6108092
H	-2.5936694	0.2597403	-1.5372373
H	-2.3179615	3.2146985	-0.7945289
H	-1.0938735	2.2963230	-1.6645922
C	-2.8921461	2.6707965	-2.8065914
H	-3.9713047	2.7779760	-2.6492872
H	-2.7370592	1.8585978	-3.5264510
H	-2.5226180	3.5969147	-3.2581419

TSCm⁺ : Si-to-C 1,3-methyl shift in **C⁺**

54

Energy = -1323.247655717

C	-0.2168359	-1.0941307	-0.1758285
C	-1.4292854	-0.7052150	0.1620376
C	-2.2602626	0.4592658	0.3730407
C	-1.9626383	1.3083567	1.4547477
C	-3.2949277	0.7896799	-0.5192024
C	-2.6781663	2.4902191	1.6207720
H	-1.1706127	1.0383388	2.1457934
C	-4.0028536	1.9730669	-0.3442144
H	-3.5231561	0.1313925	-1.3518555
C	-3.6947530	2.8231671	0.7226037
H	-2.4442080	3.1500425	2.4500736
H	-4.7931088	2.2363732	-1.0398695
H	-4.2510189	3.7459833	0.8547486
Si	-0.7467595	-2.8031000	0.1875227
C	-2.6355724	-2.1799295	0.7237803
C	-0.0872760	-3.5771565	1.7392806
C	-1.0142759	-3.9055151	-1.2788178
H	-3.4070434	-1.8832989	0.0173143
H	-2.7286792	-3.2860195	0.7918332
H	-2.8094228	-1.7967492	1.7264320
H	0.9593947	-3.8630925	1.5827515
H	-0.1308880	-2.8788271	2.5805424
H	-0.6533337	-4.4794800	1.9949740
H	-1.5512379	-4.8156824	-0.9902396
H	-1.5787689	-3.3917197	-2.0629503
H	-0.0417158	-4.1974131	-1.6921270
Si	3.6065974	0.6712039	0.3915979
C	4.2977554	0.1135183	-1.2691144
H	3.6878657	0.4646708	-2.1099736
H	5.3115535	0.5065192	-1.4121084
H	4.3551578	-0.9802558	-1.3288831
C	3.4919872	2.5497503	0.4734767

H	3.0459091	2.8707543	1.4227397	H	1.3471824	0.4345224	1.4245112
H	4.4945601	2.9907050	0.4122384	C	-0.1570972	0.9020141	-2.5532441
H	2.8927394	2.9749960	-0.3386314	H	1.5684367	1.5787115	-1.4594251
C	4.7133969	0.0659290	1.7890150	H	0.0799916	1.6599786	-0.5347764
H	4.8280870	-1.0244237	1.7630748	H	0.4607645	0.3771216	-3.2935188
H	5.7133477	0.5101237	1.7154674	H	-1.0393611	0.2721334	-2.3856668
H	4.3000115	0.3376552	2.7680487	C	-0.6016872	2.2574034	-3.1092461
C	0.9980828	-0.2976350	-0.5911933	H	0.2599018	2.9099906	-3.2911259
C	1.8957180	-0.1365300	0.6624472	H	-1.2662400	2.7673847	-2.4020409
H	1.5427281	-0.9088753	-1.3226004	H	-1.1406194	2.1399752	-4.0549379
C	0.6366098	1.0412280	-1.2501768				
H	2.0779432	-1.1277986	1.1016023				

6 References

[S1] C. A. Reed, *Acc. Chem. Res.* **2010**, *43*, 121–128.

[S2] Q. Wu, A. Roy, E. Irran, Z. W. Qu, S. Grimme, H. F. T. Klare, M. Oestreich, *Angew. Chem. Int. Ed.* **2019**, *58*, 17307–17311.

[S3] a) Z. W. Xie, R. Bau, A. Benesi, C. A. Reed, *Organometallics* **1995**, *14*, 3933–3941; b) T. He, G. Q. Wang, P. W. Long, S. Kemper, E. Irran, H. F. T. Klare, M. Oestreich, *Chem. Sci.* **2021**, *12*, 569–575; c) L. Omann, B. Pudasaini, E. Irran, H. F. T. Klare, M. H. Baik, M. Oestreich, *Chem. Sci.* **2018**, *9*, 5600–5607.

[S4] L. Omann, Z. W. Qu, E. Irran, H. F. T. Klare, S. Grimme, M. Oestreich, *Angew. Chem. Int. Ed.* **2018**, *57*, 8301–8305.

[S5] R. K. Harris, E. D. Becker, S. M. C. De Menezes, R. Goodfellow, P. Granger, *Pure. Appl. Chem.* **2001**, *73*, 1795–1818.

[S6] C. M. Che, W. Y. Yu, P. M. Chan, W. C. Cheng, S. M. Peng, K. C. Lau, W. K. Li, *J. Am. Chem. Soc.* **2000**, *122*, 11380–11392.

[S7] A. Boelke, L. D. Caspers, B. J. Nachtsheim, *Org. Lett.* **2017**, *19*, 5344–5347.

[S8] C. X. Xu, W. Y. Du, Y. Zeng, B. Dai, H. Guo, *Org. Lett.* **2014**, *16*, 948–951.

[S9] K. Sugimoto, S. Kosuge, T. Sugita, Y. Miura, K. Tsuge, Y. Matsuya, *Org. Lett.* **2021**, *23*, 3981–3985.

[S10] B. Zhao, H. L. Ma, C. G. Wang, Z. K. Shang, Y. Ding, A. G. Hu, *Macromolecules* **2020**, *53*, 240–248.

[S11] E. S. Taher, P. Guest, A. Benton, X. H. Ma, M. G. Banwell, A. C. Wills, T. Seiser, T. W. Newton, J. Hutzler, *J. Org. Chem.* **2017**, *82*, 211–233.

[S12] X. S. Luo, P. Wang, *Org. Lett.* **2021**, *23*, 4960–4965.

[S13] C. Bayon, N. He, M. Deir-Kaspar, P. Blasco, S. Andre, H. J. Gabius, A. Rumbero, J. Jimenez-Barbero, W. D. Fessner, M. J. Hernaiz, *Chem. Eur. J.* **2017**, *23*, 1623–1633.

[S14] H. Ueda, M. Yamaguchi, H. Kameya, K. Sugimoto, H. Tokuyama, *Org. Lett.* **2014**, *16*, 4948–4951.

[S15] L. Liu, D. Zhou, M. Liu, Y. B. Zhou, T. Q. Chen, *Org. Lett.* **2018**, *20*, 2741–2744.

[S16] K. Yamaguchi, Y. Wang, T. Oishi, Y. Kuroda, N. Mizuno, *Angew. Chem. Int. Ed.* **2013**, *52*, 5627–5630.

[S17] C. W. Liu, M. Szostak, *Org. Lett.* **2021**, *23*, 4726–4730.

[S18] Y. Unoh, K. Hirano, T. Satoh, M. Miura, *Angew. Chem. Int. Ed.* **2013**, *52*, 12975–12979.

[S19] L. Zhao, R. I. Kaiser, J. Phys. Chem. Lett. 2018, 8, 2620–2626.

[S20] X. F. Liu, B. X. Liu, Q. Liu, *Angew. Chem. Int. Ed.* **2020**, *59*, 6750–6755.

[S21] K. Tamao, J. I. Yoshida, M. Akita, Y. Sugihara, T. Iwahara, M. Kumada, *Bull. Chem. Soc. Jpn.* **1982**, *55*, 255–260.

[S22] L. A. Paquette, J. P. Gilday, G. D. Maynard, *J. Org. Chem.* **1989**, *54*, 5044–5053.

[S23] TURBOMOLE V7.4 **2019**, TURBOMOLE GmbH; available from http://www.turbomole.com.

[S24] J. Tao, J. P. Perdew, V. N. Staroverov, G. E. Scuseria, *Phys. Rev. Lett.* **2003**, *91*, 146401.

[S25] a) S. Grimme, J. Antony, S. Ehrlich, H. Krieg, *J. Chem. Phys.* **2010**, *132*, 154104–154119; b) S. Grimme, S. Ehrlich, L. Goerigk, *J. Comput. Chem.* **2011**, *32*, 1456–1465.

[S26] a) F. Weigend, M. Häser, H. Patzelt, R. Ahlrichs, *Chem. Phys. Lett.* **1998**, *294*, 143–152; b) F. Weigend, R. Ahlrichs, *Phys. Chem. Chem. Phys.* **2005**, *7*, 3297–3305.

[S27] A. Klamt, G. Schüürmann, *J. Chem. Soc. Perkin Trans. 2* **1993**, 799–805.

[S28] a) K. Eichkorn, F. Weigend, O. Treutler, R. Ahlrichs, *Theor. Chem. Acc.* **1997**, *97*, 119–124; b) F. Weigend, *Phys. Chem. Chem. Phys.* **2006**, *8*, 1057–1065.

[S29] P. Deglmann, K. May, F. Furche, R. Ahlrichs, *Chem. Phys. Lett.* **2004**, *384*, 103–107.

[S30] S. Grimme, *Chem. Eur. J.* **2012**, *18*, 9955–9964.

[S31] F. Eckert, A. Klamt, *AIChE J.* **2002**, *48*, 369–385.

[S32] F. Eckert, A. Klamt, *COSMOtherm, Version C3.0, Release 16.01*, COSMOlogic GmbH & Co. KG, Leverkusen, Germany, **2015**.

[S33] Y. Zhao, D. G. Truhlar, *J. Phys. Chem. A* **2005**, *109*, 5656–5667.

[S34] F. Weigend, F. Furche, R. Ahlrichs, *J. Chem. Phys.* **2003**, *119*, 12753–12762.

[S35] L. Goerigk, A. Hansen, C. Bauer, S. Ehrlich, A. Najibi, S. Grimme, *Phys. Chem. Chem. Phys.* **2017**, *19*, 32184–32215.
